# Atrial Fibrillation Is an Independent Risk Factor for Hospital-Acquired Pneumonia

**DOI:** 10.1371/journal.pone.0131782

**Published:** 2015-07-23

**Authors:** Jinxiu Zhu, Xin Zhang, Ganggang Shi, Kaihong Yi, Xuerui Tan

**Affiliations:** 1 Department of Cardiology, the First Affiliated Hospital of Shantou University Medical College, Shantou, Guangdong, 515041, China; 2 Molecular Cardiology Laboratory, the First Affiliated Hospital of Shantou University Medical College, Shantou, Guangdong, 515041, China; 3 Department of Pharmacology, Shantou University Medical College, Shantou, Guangdong, 515032, China; Mayo Clinic, UNITED STATES

## Abstract

**Background:**

Patients who were hospitalized for community-based pneumonia frequently had pre-existing atrial fibrillation (AF) and had subsequent cardiovascular complications. Whether patients who had AF would be susceptible to the development of hospital-acquired pneumonia (HAP) is a serious concern but this has not been investigated. In our clinics, we have made empirical observation of such susceptibility.

**Objectives:**

To investigate the association between newly developed HAP and pre-existing AF, and to identify whether AF is an independent risk factor for HAP.

**Methods:**

Hospital data from 8657 sequentially admitted inpatients [1059 patients with AF and 7598 without AF (NAF)] were collected from the Department of Cardiology, First Affiliated Hospital of Shantou University Medical College, Shantou, China, from January 1, 2009 to December 31, 2011. Exclusion criteria were: having previous or current pneumonia, pacemakers, sick sinus syndrome and repeated hospitalization. The incidence of HAP (within 48 hours after hospitalization) was identified among all the patients.

**Results:**

Among the AF patients, 274 had HAP (adjusted rate 25.64%) which was significantly higher than the 276 NAF patients who had HAP (adjusted rate 3.66%; *P*<0.001). The increased risk was also associated with high blood pressure, heart failure and age, but not with gender, smoking, coronary heart disease, diabetes, congenital heart disease. In addition, our multiple regression analysis indicates that AF is an independent risk factor for HAP.

**Conclusion:**

We have identified, for the first time, that AF is an important risk factor for HAP. Although additional clinical confirmation is needed, our data provide valuable evidence for use in prevention of HAP which is the most common cause of death from nosocomial infection.

## Introduction

Pneumonia is not only a serious medical problem by itself but it can significantly increase mortality for other types of diseases [1,2]. Therefore, both types of pneumonia: community- and hospital-acquired pneumonia, are major medical problems. However, most investigations have been focused on the prevention and management of community-acquired pneumonia to reduce disease complications. Therefore, disease complications due to hospital-acquired pneumonia (HAP) may be underestimated.

HAP is classified as a primary pneumonia that arises ≥48 hours after admission into hospitals [3]. It is one of the leading nosocomial infections worldwide which accounts for 13–18% of all nosocomial infections that affect 0.5–2.0% of hospitalized patients [4]. In fact, HAP is as commonly found in Asian countries as in developed countries [5]. HAP is also associated with elevated morbidity and mortality and increased hospital costs [3].

Within hospitals, the incident rate of HAP is higher in intensive care units (ICU) than that in general wards due to the use of artificial airways (endotracheal tubes and tracheostomies [6]. In addition, mechanical ventilation is the most significant risk factor for developing HAP, with incidence of 8% to 28% among ventilated patients [7]. Other risk factors include advanced age, severe underlying illness, long duration of hospital stay, and antibiotics use. Outside ICUs, risk factors include malnutrition, chronic renal failure, anemia, depression of consciousness, comorbidity, recent hospitalization, and thoracic surgery [8].

As mentioned earlier, AF as a plausible cause for HAP has not been investigated. Therefore, we have made an internal audit of clinical data and made empirical observation that hospitalized patients with atrial fibrillation (AF) appeared to have high prevalence of HAP. Therefore, AF patients may be susceptible to the development of HAP. Based on the observation, we have conducted a case-control study to determine the incidence of and risk factors for HAP among patients with and without AF. Our results indicate that AF is a significant and independent risk factor for HAP among hospitalized AF patients.

## Methods

### Patient recruitment and organization

All patients who were hospitalized from January 1, 2009 to December 31, 2011 in the Division of Cardiology of the First Affiliated Hospital of Shantou University Medical College in Shantou, China, were recruited into our study. Patients who had the following conditions were excluded from the study: 1) previous surgery, or mechanical ventilation or other interventional procedures; 2) community-acquired pneumonia; 3) the presence of potential pneumonia symptoms such as fever, pulmonary shadows, and white blood cell abnormalities at admission; 4) pacemaker implant recipients; 5) sick sinus syndrome; and 6) repeat hospitalization for any reason, in the selected period. The study was approved by the First Affiliated Hospital of Shantou University Medical College and complied with the local laws. This study did not need to write the informed consent because this was a retrospective analysis. The information from patient records was anonymized. All patient data were de-identified prior to analysis in order to ensure that the patients did not any undergo surgery including interventional therapy and radiofrequency ablation, mechanical ventilation or other interventional procedures during their stay at hospital.

Routinely collected personal and medical information were retrieved. All the data were collected (see S1 Dataset). According to hospital information, patients were divided into AF and NAF groups, and each group was divided into two subgroups based on the presence or absence of HAP according to the following criteria. In addition, patients with AF were divided into paroxysmal AF (VPAF) and non-PAF (NPAF). PAF was lasting less than 7 days, and more than or equal to 7 days was NPAF [9].

### Diagnostic Criteria of AF

AF was defined by having cardiac arrhythmia with specific characteristics on electrocardiogram (ECG) or dynamic ECG according to the European Society of Cardiology Guidelines [10]: (1) surface ECG shows ‘absolutely’ irregular RR intervals; (2) there is no distinct P wave on the surface ECG; and (3) the atrial cycle length is about 200ms (300bpm).

### Diagnostic Criteria of HAP

HAP is recognized if patients developed pneumonia at least 48 hours after hospitalization. Their clinical diagnosis was based on guidelines of the American Thoracic Society [3]. The diagnostic criteria were: abnormal X-ray shadows in lungs and presence of at least two of the followings: (1) fever ≥38°C; (2) white blood cell abnormalities (increase or decrease); and (3) purulent secretions.

### Statistical Analysis

EPI Data 3.02 was used for data entry and SPSS (IBM SPSS Statistics, version 19.0) for data analysis. Discrete variables were expressed as counts (percentage) and continuous variables as $\bar{x}\pm SD$. The independent sample *t* test (2-sided) and *chi-square* test (*χ* ^*2*^ test) was used for comparison among groups. The logistic regression analysis was used to analyze risk factors for HAP. Significance was defined as *P<*0.05.

## Results

### Baseline Characteristics of Patients

During the 3-year study period, a total of 8657 patients met our inclusion-exclusion criteria. Among the patients, 1059 (12.23%) belonged to the AF group and 7598 (87.77%) to the NAF group. In 1059 cases of AF included 556 (52.5%) PAF and 523 (47.5%) NPAF, 274 (25.87%) cases had HAP. HAP incidence in PAF was 30.03% (167 cases) which was significantly higher than in NPAF (21.27%, 107 cases, *P* = 0.036). Their general information (age, sex and smoking habit) are shown in Table 1. The average age was 65.8±12.9 years in AF group and 60.0±14.5 years in NAF group (*P*<0.001). Gender distribution (*P* = 0.359) and smoking habit (*P* = 0.633) were not significantly different between the two groups. The combined diseases had some differences between AF group and NAF group. Therefore, further analyses of the data were adjusted accordingly.

**Table 1:** Characteristics of the study population. HBP = high blood pressure; CHD = coronary heart disease; DM = diabetes mellitus; RHD = rheumatic heart disease; NRVD = non rheumatic valvular disease; MM = myocardiopathy/myocardiotis; HLD = hyperlipidemia; ED = electrolyte disturbance; cHD = congenital heart disease; HF = heart failure, which diagnosed according to NYHA (Criteria of New York Heart Association). The overlap among the underlying disease, a patient may be accompanied by a variety of illness

Characteristics	AF Group [Total number = 1059, mean (SD) or n (%)]	NAF Group [Total number = 7598, mean (SD) or n (%)]	χ^2^ or *t*-value	*P*-value
Age, years	65.8 (13.0)	60.0 (14.5)	8.157	<0.001
Sex, *n* (%)			0.841	0.359
Males	575(54.3)	4239(55.8)		
Females	484(45.7)	3359(44.2)		
Smoker	246 (23.2)	1725 (22.7)	0.228	0.633
Combined diseases				
HBP	373(35.2)	3040(40.0)	8.925	0.003
CHD	330(31.2)	2591(34.1)	3.593	0.058
DM	141(13.3)	656(8.6)	24.360	<0.001
RHD	127(12.0)	69(0.9)	516.063	<0.001
NRVD	108(10.2)	168(2.2)	192.108	<0.001
MM	46(4.3)	256(3.4)	2.621	0.108
HLD	42(4.0)	245(3.2)	1.594	0.207
ED	17(1.6)	70(0.9)	4.371	0.037
cHD	12(1.1)	53(0.7)	2.367	0.124
HF	466(44.0)	1581(20.8)	276.986	<0.001

### HAP Prevalence in AF and NAF Groups

Among the 1059 AF patients, 274 (25.87%) had HAP. Among the 7598 NAF patients, 276 (3.63%) had HAP. After adjustment by age, the HAP incidence in the AF group was still significantly higher than that in the NAF group (25.64% *vs*. 3.66%, *P<*0.001; Table 2). Importantly, the in-hospital mortality was significantly increased in patients with HAP than those without HAP, irrespective of their AF status (6.57% *vs*. 2.42%, *P* = 0.003). In addition, both the average hospitalization stays (15.9±10.2 *days vs*. 9.1±5.9 days, *P<*0.001) and the average medical cost per patient (RMB 13845.4±5801.3 yuan *vs*. 10607.8±4537.9 yuan; about US$ 2233 and US$ 1710, respectively; *P* = 0.002) in AF patients with HAP were significantly higher than in AF patients without HAP (Table 3).

**Table 2:** Differences of HAP prevalence adjusted by age between AF and NAF groups.

Age stratification	Total of AF and NAF (n)	AF group (n)	AF with HAP (n)	HAP prevalence in AF group (%)	Expected number of HAP cases (n)	HAP prevalence adjusted by age in AF group (%)	NAF group (n)	NAF with HAP (n)	HAP prevalence in NAF group (%)	Expected number of HAP cases (n)	HAP prevalence adjusted by age in NAF group (%)
<30	239	5	2	40.00	96	40.17	234	6	2.56	6	2.51
30–39	401	33	4	12.12	49	12.22	368	13	3.53	14	3.49
40–49	896	94	21	22.34	200	22.32	802	20	2.49	22	2.46
50–59	1855	177	42	23.73	440	23.72	1678	48	2.86	53	2.86
60–69	2158	291	74	25.43	549	25.44	1867	49	2.62	57	2.64
70-	3108	459	131	28.54	887	28.54	2649	140	5.29	164	5.28
Total	8657	1059	274	25.87 ^a^	2220	25.64^a^	7598	276	3.63 ^a^	317	3.66 ^a^

**Table 3:** Mortality, length of hospital stay and hospital cost for AF patients.

Variables	HAP Subgroup Group [Total number = 275, mean (SD) or n (%)]	NHAP Subgroup Group [Total number = 787, mean (SD) or n (%)]	*P*-Value
Mortality	18 (6.57%)	19 (2.42%)	0.003
Hospital stay (day)	15.9±10.2	9.1±5.9	<0.001
Cost (RMB)	13845.4±5801.3	10607.8±4537.9	0.002

^a^ *t* = 5.935, *P*<0.001

### Multifactor Analysis on Risk Factor of HAP

Results from logistic regression analysis of risk factors for HAP are shown in Table 4. The results show that there were increased susceptibility to HAP in patients with high blood pressure (HBP; OR = 4.695, 95% CI = 3.812–5.782), heart failure (HF; OR = 2.854, 95% CI = 2.355–3.459) and AF (OR = 13.386, 95% CI = 8.591–20.858). As for age, the older the patients were the higher the risk they had. However, the gender, smoking habit, coronary heart disease (CHD), diabetes mellitus (DM), rheumatic heart disease (RHD), non-rheumatic valvular disease (NRVD), myocardiopathy/myocardiotis (MM), hyperlipidemia (HLD), electrolyte disturbance (ED) and congenital heart disease (cHD), were not significant risk factors for HAP. The analysis showed that AF was an independent risk factor of HAP.

**Table 4:** Results of Logistic Regression Analysis Based on HAP (n = 8657).

	β	SE	Wald	*P*-value	OR	95% CI
Intercept^a^	3.065	1.003	9.333	0.002		
Age<30Y	0.481	0.468	1.059	0.303	1.618	0.647–4.046
Age 30∼39Y^a^	0.686	0.338	4.109	0.043	1.985	1.023–3.853
Age 40∼49Y^a^	0.543	0.242	5.052	0.025	1.722	1.072–2.765
Age 50∼59Y^a^	0.555	0.150	13.709	0.000	1.742	1.298–2.337
Age 60∼69Y^a^	0.475	0.132	13.022	0.000	1.609	1.243–2.083
Age 70∼Y^a^	0.214	0.055	14.977	0.000	0.808	1.725–1.900
gender	0.074	0.099	0.569	0.451	1.077	0.888–1.307
smoking	0.066	0.239	0.076	0.783	1.068	0.668–1.707
HBP^a^	1.546	0.106	211.872	0.000	4.695	3.812–5.782
CHD	-0.111	0.309	0.128	0.720	0.895	0.489–1.640
DM	-0.012	0.158	0.006	0.939	0.988	0.726–1.345
RHD	-0.385	0.234	2.708	0.100	0.681	0.430–1.076
NRVD	0.099	0.206	0.230	0.631	1.104	0.737–1.654
MM	-0.024	0.270	0.008	0.930	0.977	0.575–1.658
HLD	-0.415	0.283	2.144	0.143	0.660	0.379–1.151
ED	0.267	0.406	0.433	0.511	1.306	0.589–2.894
cHD	-1.032	0.754	1.875	0.171	0.356	0.081–1.561
HF^a^	1.049	0.098	114.304	0.000	2.854	2.355–3.459
AF^a^	2.594	0.226	131.411	0.000	13.386	8.591–20.858

^a^ = statistical significance.

## Discussion

AF which is the one of the most common forms of arrhythmia in humans [11] can also cause a multitude of clinical complications [12,13]. For example, advanced age (58.1%), HBP (40.3%), CHD (34.8%), HF (33.1%), RVD (23.9%), MM (5.4%) and DM (4.1%) are the most common concurrent conditions. Consequently, AF is associated with increased stroke, other thromboembolic events and death. Our data show that AF is also a major risk factor for HAP. A report of Topel AE et al. showed that AF post cardiac surgery is a risk factor for the development of HAP [14]. Our results have also revealed the relationship between AF and HAP. Interestingly, the subjects in each study were both with AF, whereas the AF patients in our study did not undergo any surgery and other interventional procedures, suggests that AF alone could be the independent risk for HAP. We think the findings focus on the point is a landmark.

The main findings of our investigations are: 1) AF is an important and independent risk factor of HAP in cardiovascular inpatients in Shantou, China. 2) having HAP significantly increased morbidity and mortality, patients’ hospitalization stays and total medical costs. 3) HBP and HF are also the important risk factors of HAP in cardiovascular inpatients but not all cardiovascular problems, e.g. CHD, RHD, NRVD, MM, cHD were risk factors.

Consistent with previous reports, our study also found that high blood pressure and heart failure were significantly associated with HAP. The other cardiovascular problems, e.g. CHD, RHD, NRVD, MM, cHD had no correlation with HAP. Oliveira TF et al has reported that hypertension was statistically associated with nosocomial pneumonia, compared with controls, OR = 2.22 (95% CI: 1.05–4.72) [15]. A population-based case-control study, conducted by Mor A, et al showed that patients with heart failure had an almost doubled risk of nosocomial pneumonia, OR = 1.81 (95% CI: 1.76–1.86) [16]. The most important finding of our analysis is that AF was an independent risk factor for HAP, OR = 13.386 (95% CI = 8.591–20.858). Combination of heart failure in a patient with AF may lead to more serious alveoli flooding, reduced microbial clearance and symptomatic deterioration, thus favoring the respiratory infection. Therefore, we consider that combination of risk factors (ie heart failure, high blood pressure and AF) might increase the incidence of HAP. However, our work was a single center, retrospective study with a small sample size of AF patients. The inference of this point must be confirmed in further randomized, larger, prospective clinical study.

A previous study showed that the prevalence of stroke was significantly higher in patients with AF compared to those patients without AF (12.1% *vs* 2.1%, *P*<0.01) [17]. Our study showed that AF patients also had significantly higher HAP incident rate of 25.9% which was higher than that for stroke (12.1%).

In the present study, HAP incidence in PAF was 30.03% (167 cases) which was significantly higher than in NPAF (21.27%, 107 cases, *P* = 0.036). One possible explanation is that NPAF has set up cardiopulmonary balance because it relative long duration of AF, whereas PAF lack adaptive compensatory mechanism, which might lead to decreased cardiac output and pulmonary congestion that may thus favorate the respiratory infection.

With our novel association between AF and HAP, it is necessary to consider possible mechanisms for the association. On the one hand, the traditional risk factors for HAP, e.g. severe and lingering illnesses, tracheal intubations and/or mechanical ventilation were not involved in our case because these confounding factors had been eliminated as exclusion criteria. On the other hand, a significant number of cardiovascular diseases, including AF, developed following hospital admission for community-acquired pneumonia [18]. In addition, such hospitalized pneumonia patients were frequently found to have pre-existing community-acquired pneumonia [1]. Therefore, there are strong relationship between pneumonia and AF.

In our case, the higher incidence of HAP in the inpatients with AF may have been due to hemodynamic changes. During AF, atrial irregular activities can lead to decreased cardiac output and pulmonary congestion, which can promote pulmonary infection. Research has shown that up to one-half of patients who had acute stroke had clinical or video fluoroscopic evidence of aspiration [19–21]. AF carries three to four folds increased risk of stroke but seven folds increased risk of HAP (from our study).

Within the same context, our patients with AF were older and had more chronic conditions, e.g. HBP, DM, RHD, NRVD, ED as well as HF which could have compromised defense mechanism against infection and thus had a greater tendency to develop pneumonia. These and other potential confounding factors were considered in our multifactor analysis. The analysis showed that AF was an independent risk factor for HAP. In addition, our data show that the combination of AF and HAP increased hospital cost, hospital stay and morbidity, therefore, most probably mortality. Although additional investigations are needed to validate our novel observation, our investigation provides valuable information for use in prevention of HAP which is the most common cause of death from nosocomial infection.

## Supporting Information

S1 Dataset The raw data of the study. The supporting information file of this paper contains 1 file, named “S1 Dataset”. In this file, we listed all the raw data from 8657 sequentially admitted inpatients [1059 patients with atrial fibrillation (AF) and 7598 without AF]. The statistic items include gender (1 expresses male and 2 expresses female), age. Others are clinical situation, such as a patient with (marked as 1) or without (marked as 0) hospital acquired pneumonia, coronary heart disease, diabetes mellitus, non rheumatic valvular disease, hyperlipoidemia, congenital heart disease, electrolyte disturbance, myocardiopathy/myocarditis, rheumatic heart disease, high blood pressure and AF. For heart failure (HF), the number “0” represents the patient without HF, and the number “1, 2, 3 or 4” separately represents the patient with HF of “1, 2, 3 or 4” class, according to NYHA (Criteria of New York Heart Association). The overlap among the underlying disease, a patient may be accompanied by a variety of illness.
(DOCX)
