# Supplementary material for: Atrial Fibrillation Is an Independent Risk Factor for Hospital-Acquired Pneumonia
Source: PLoS One. 2015 Jul 23;10(7):e0131782. doi: 10.1371/journal.pone.0131782 (PMC4512692; doi:10.1371/journal.pone.0131782)
Supplement: S1 Dataset — The supporting information file of this paper contains 1 file, named “S1 Dataset”. In this file, we listed all the raw data from 8657 sequentially admitted inpatients [1059 patients with atrial fibrillation (AF) and 7598 without AF]. The statistic items include gender (1 expresses male and 2 expresses female), age. Others are clinical situation, such as a patient with (marked as 1) or without (marked as 0) hospital acquired pneumonia, coronary heart disease, diabetes mellitus, non rheumatic valvular disease, hyperlipoidemia, congenital heart disease, electrolyte disturbance, myocardiopathy/myocarditis, rheumatic heart disease, high blood pressure and AF. For heart failure (HF), the number “0” represents the patient without HF, and the number “1, 2, 3 or 4” separately represents the patient with HF of “1, 2, 3 or 4” class, according to NYHA (Criteria of New York Heart Association). The overlap among the underlying disease, a patient may be accompanied by a variety of illness. (DOCX) [file pone.0131782.s001.docx]

Pneumonia/ gender/ age/ CHD/ DM/ NRVD/ hyperlipoidemia/ CHD1/ ED/ MM/ RHD/ HBP/ HF/AF

0 1 63 1 0 0 0 0 1 1 0 1 0 1

0 1 84 0 0 1 0 0 1 0 0 1 0 1

0 2 63 0 0 1 0 0 1 0 0 1 0 1

0 2 71 0 0 1 0 0 1 0 0 1 0 1

1 1 53 0 0 0 0 0 1 0 0 1 3 1

1 1 65 1 0 0 0 0 1 0 0 1 0 1

1 2 76 0 0 0 0 0 1 0 0 1 0 1

0 1 64 1 0 0 0 0 1 0 0 1 2 1

0 1 65 0 0 0 0 0 1 0 0 1 0 1

0 1 70 1 0 0 0 0 1 0 0 1 0 1

0 2 65 0 0 0 0 0 1 0 0 1 0 1

0 2 72 1 1 0 0 0 1 0 0 1 0 0

0 2 59 1 1 0 0 0 1 0 0 1 0 0

0 2 53 1 1 0 0 0 1 0 0 1 0 0

0 1 53 1 1 0 0 0 1 0 0 1 3 0

0 1 84 1 1 0 0 0 1 0 0 1 0 0

0 2 87 1 1 0 0 0 1 0 0 1 0 0

0 2 73 1 1 0 0 0 1 0 0 1 0 0

0 2 65 1 1 0 0 0 1 0 0 1 0 0

1 1 46 0 1 0 0 0 1 0 0 1 2 0

1 1 46 0 1 0 0 0 1 0 0 1 2 0

1 2 80 0 1 0 0 0 1 0 0 1 2 0

1 2 98 0 1 0 0 0 1 0 0 1 2 0

0 1 39 0 1 0 0 0 1 0 0 1 0 0

0 1 36 0 1 0 0 0 1 0 0 1 0 0

0 1 51 0 1 0 0 0 1 0 0 1 0 0

0 1 48 0 1 0 0 0 1 0 0 1 0 0

0 1 62 0 1 0 0 0 1 0 0 1 0 0

0 2 37 0 1 0 0 0 1 0 0 1 0 0

0 2 38 0 1 0 0 0 1 0 0 1 0 0

0 2 23 0 1 0 0 0 1 0 0 1 0 0

0 1 53 0 1 1 0 0 1 0 0 1 0 0

0 1 67 0 1 0 0 0 1 0 0 1 0 0

0 1 31 0 1 0 0 0 1 0 0 1 0 0

0 1 17 0 1 0 0 0 1 0 0 1 0 0

0 1 31 0 1 0 0 0 1 0 0 1 0 0

0 1 31 0 1 0 0 0 1 0 0 1 0 0

0 1 56 0 1 0 0 0 1 0 0 1 0 0

0 1 36 0 1 0 0 0 1 0 0 1 0 0

0 1 24 0 1 0 0 0 1 0 0 1 0 0

0 1 69 0 1 0 0 0 1 0 0 1 0 0

0 1 81 0 1 0 0 0 1 0 0 1 0 0

0 1 54 0 1 0 0 0 1 0 0 1 0 0

0 1 86 0 1 0 0 0 1 0 0 1 0 0

0 1 91 0 1 0 0 0 1 0 0 1 0 0

0 1 70 0 1 0 0 0 1 0 0 1 0 0

0 1 70 0 1 0 0 0 1 0 0 1 0 0

0 1 37 0 1 0 0 0 1 0 0 1 0 0

0 1 61 0 1 0 1 0 1 0 0 1 0 0

0 2 57 0 1 0 0 0 1 0 0 1 0 0

0 2 69 0 1 0 0 0 1 0 0 1 0 0

0 2 59 0 1 0 0 0 1 0 0 1 0 0

0 2 55 0 1 0 0 0 1 0 0 1 0 0

0 2 32 0 1 0 0 0 1 0 0 1 0 0

0 2 63 0 1 0 0 0 1 0 0 1 0 0

0 2 66 0 1 0 0 0 1 0 0 1 0 0

0 2 79 0 1 0 0 0 1 0 0 1 0 0

0 2 70 0 1 0 0 0 1 0 0 1 0 0

0 2 60 0 1 0 0 0 1 0 0 1 0 0

0 2 47 0 1 0 0 0 1 0 0 1 0 0

0 2 72 0 1 0 0 0 1 0 0 1 0 0

0 2 64 0 1 0 0 0 1 0 0 1 0 0

0 2 73 0 1 0 0 0 1 0 0 1 0 0

0 2 76 0 1 0 0 0 1 0 0 1 0 0

0 2 64 0 1 0 0 0 1 0 0 1 3 0

0 2 83 0 0 0 0 0 1 0 0 1 3 0

0 2 53 0 0 0 0 0 1 0 0 1 3 0

0 2 75 0 0 0 0 0 1 0 0 1 3 0

0 2 52 0 0 0 0 0 1 0 0 1 0 0

0 2 39 0 0 0 0 0 1 0 0 1 0 0

0 2 81 0 0 0 0 0 1 0 0 1 0 0

0 2 66 0 0 0 0 0 1 0 0 1 0 0

0 1 61 0 1 0 0 0 1 1 0 1 0 0

0 1 66 0 1 0 0 0 1 1 0 1 0 0

0 1 65 0 1 0 0 0 1 1 0 1 0 0

0 1 65 0 1 0 0 0 1 1 0 1 0 0

0 1 65 0 1 0 0 0 1 1 0 1 0 0

0 1 65 0 1 0 0 0 1 1 0 1 0 0

0 1 65 0 1 0 0 0 1 1 0 1 0 0

0 1 61 0 1 0 0 0 1 1 0 1 0 0

0 1 62 0 1 0 0 0 1 1 0 1 0 0

0 1 68 0 1 0 0 0 1 1 0 1 0 0

0 2 33 1 0 0 0 0 1 1 0 1 3 1

0 2 66 1 0 1 0 0 1 0 0 1 0 1

0 2 52 0 0 1 0 0 1 0 0 1 3 1

1 1 44 0 0 0 0 0 1 0 0 1 0 1

0 2 72 1 0 0 0 0 1 0 0 1 3 1

0 2 89 0 0 0 0 0 1 0 0 1 0 1

1 1 62 1 1 0 0 0 0 1 1 1 0 0

1 1 58 1 0 0 0 0 0 0 1 1 0 0

1 1 63 1 0 0 0 0 0 0 1 1 0 0

1 1 68 1 0 0 0 0 0 0 1 1 0 0

1 1 89 1 0 0 0 0 0 0 1 1 0 0

1 1 76 1 0 0 0 0 0 0 1 1 0 0

1 1 79 1 0 0 0 0 0 0 1 1 0 0

1 1 88 1 0 0 0 0 0 0 1 1 2 0

1 1 71 1 0 0 0 0 0 0 0 1 0 0

1 1 77 1 0 0 0 0 0 0 0 1 0 0

1 1 76 1 0 0 0 0 0 0 0 1 0 0

1 1 90 1 0 0 0 0 0 0 0 1 0 0

1 1 52 1 0 0 0 0 0 0 0 1 0 0

1 1 50 1 0 0 0 0 0 0 0 1 0 0

1 1 58 1 0 0 0 0 0 0 0 1 0 0

1 1 79 1 0 0 0 0 0 0 0 1 0 0

1 1 80 1 0 0 0 0 0 0 0 1 0 0

1 1 82 1 0 0 0 0 0 0 0 1 0 0

1 1 69 1 0 0 0 0 0 0 0 1 0 0

1 1 65 1 0 0 0 0 0 0 0 1 0 0

1 1 53 1 0 0 0 0 0 0 0 1 0 0

1 1 75 1 0 0 0 0 0 0 0 1 0 0

1 1 75 1 0 0 0 0 0 0 0 1 0 0

1 1 85 1 0 0 0 0 0 0 0 1 0 0

1 1 49 1 0 0 0 0 0 0 0 1 0 0

1 1 78 1 0 0 0 0 0 0 0 1 0 0

1 1 59 1 0 0 0 0 0 0 0 1 0 0

1 1 74 1 0 0 0 0 0 0 0 1 0 0

1 1 79 1 0 0 0 0 0 0 0 1 0 0

1 1 54 1 0 0 0 0 0 0 0 1 0 0

1 1 66 1 0 0 0 0 0 0 0 1 0 0

1 1 75 1 0 0 0 0 0 0 0 1 0 0

1 1 68 1 0 0 0 0 0 0 0 1 0 0

1 1 79 1 0 0 0 0 0 0 0 1 3 0

1 1 87 1 0 0 0 0 0 0 0 1 0 0

1 1 79 1 0 0 0 0 0 0 0 1 0 0

1 1 76 1 0 0 0 0 0 0 0 1 0 0

1 2 78 1 0 0 0 0 0 0 0 1 0 0

1 2 78 1 0 0 0 0 0 0 0 1 0 0

1 2 80 1 0 0 0 0 0 0 0 1 0 0

1 2 80 1 0 0 0 0 0 0 0 1 0 0

1 2 92 1 0 0 0 0 0 0 0 1 0 0

1 2 84 1 0 0 0 0 0 0 0 1 0 0

1 2 87 1 0 0 0 0 0 0 0 1 0 0

1 2 84 1 0 0 0 0 0 0 0 1 0 0

1 2 71 1 0 0 0 0 0 0 0 1 3 0

1 2 79 1 0 0 0 0 0 0 0 1 0 0

1 2 60 1 0 0 0 0 0 0 0 1 0 0

1 2 71 1 0 0 0 0 0 0 0 1 0 0

1 2 71 1 0 0 0 0 0 0 0 1 0 0

1 2 83 1 0 0 0 0 0 0 0 1 0 0

1 2 58 1 0 0 0 0 0 0 0 1 0 0

0 1 79 1 1 0 0 0 0 1 0 1 0 0

0 1 84 1 0 0 0 0 0 0 0 1 0 0

0 1 57 1 0 0 0 0 0 0 0 1 0 0

0 1 60 1 0 0 0 0 0 0 0 1 0 0

0 1 80 1 0 0 0 0 0 0 0 1 0 0

0 1 55 1 0 0 1 0 0 0 0 1 0 0

0 1 73 1 0 0 0 0 0 0 0 1 0 0

0 1 55 1 0 0 0 0 0 0 0 1 0 0

0 1 74 1 0 0 0 0 0 0 0 1 0 0

0 1 70 1 0 0 0 0 0 0 0 1 0 0

0 1 61 1 0 0 0 0 0 0 0 1 0 0

0 1 82 1 0 0 0 0 0 0 0 1 0 0

0 1 87 1 0 0 0 0 0 0 0 1 0 0

0 2 73 1 0 0 0 0 0 0 0 1 0 0

0 2 85 1 0 0 0 0 0 0 0 1 0 0

0 2 69 1 0 0 0 0 0 0 0 1 0 0

0 2 69 1 0 0 0 0 0 0 0 1 0 0

0 2 80 1 0 0 0 0 0 0 0 1 0 0

0 2 78 1 0 0 0 0 0 0 0 1 0 0

0 2 83 1 0 0 0 0 0 0 0 1 0 0

0 2 65 1 0 0 0 0 0 0 0 1 0 0

0 2 91 1 0 0 0 0 0 0 0 1 0 0

0 2 81 1 0 0 0 0 0 0 0 1 0 0

0 2 79 1 0 0 0 0 0 0 0 1 0 0

0 2 70 1 0 0 0 0 0 0 0 1 0 0

0 2 75 1 0 0 0 0 0 0 0 1 0 0

0 2 54 1 0 0 0 0 0 0 0 1 0 0

0 2 53 1 0 0 0 0 0 0 0 1 0 0

0 2 55 1 0 0 0 0 0 0 0 1 0 0

0 2 66 1 0 0 0 0 0 0 0 1 0 0

0 1 45 1 1 0 0 0 0 1 0 1 0 0

0 1 59 1 1 0 0 0 0 1 0 1 0 0

0 1 53 1 1 0 0 0 0 1 0 1 0 0

0 1 43 1 1 0 0 0 0 1 0 1 0 0

0 2 70 1 1 0 0 0 0 1 0 1 0 0

0 2 58 1 1 0 0 0 0 1 0 1 0 0

0 2 76 1 1 0 0 0 0 1 0 1 0 0

0 2 59 1 1 0 0 0 0 1 0 1 0 0

0 1 74 1 1 0 0 0 0 1 0 1 0 0

0 1 49 1 1 0 0 0 0 1 0 1 0 0

0 1 52 1 1 0 0 0 0 1 0 1 0 0

0 1 53 1 1 0 0 0 0 1 0 1 0 0

0 1 61 1 1 0 0 0 0 1 0 1 0 0

0 1 73 1 1 0 0 0 0 1 0 1 0 0

0 1 67 1 1 0 0 0 0 1 0 1 0 0

0 1 71 1 1 0 0 0 0 1 0 1 0 0

0 1 82 1 1 0 0 0 0 1 0 1 0 0

0 1 61 1 1 0 0 0 0 1 0 1 0 0

0 1 75 1 1 0 0 0 0 1 0 1 0 0

0 1 52 1 1 0 0 0 0 1 0 1 4 0

0 1 68 1 1 0 0 0 0 1 0 1 0 0

0 1 47 1 1 0 0 0 0 1 0 1 0 0

0 1 58 1 1 0 0 0 0 1 0 1 3 0

0 1 68 1 1 0 0 0 0 1 0 1 3 0

0 1 80 1 1 0 0 0 0 1 0 1 4 0

0 1 73 1 1 0 0 0 0 1 0 1 4 0

0 1 58 1 1 0 0 0 0 1 0 1 4 0

0 1 73 1 1 0 0 0 0 1 0 1 4 0

0 1 68 1 1 0 0 0 0 1 0 1 0 0

0 1 78 1 1 0 0 0 0 1 0 1 0 0

0 1 77 1 1 0 0 0 0 1 0 1 4 0

0 1 71 1 1 0 0 0 0 1 0 1 0 0

0 1 62 1 1 0 0 0 0 1 0 1 0 0

0 1 73 1 1 0 0 0 0 1 0 1 0 0

0 1 75 1 1 0 0 0 0 1 0 1 0 0

0 1 75 1 1 0 0 0 0 1 0 1 0 0

0 1 74 1 1 0 0 0 0 1 0 1 0 0

0 1 71 1 1 0 0 0 0 1 0 1 0 0

0 1 52 1 1 0 0 0 0 1 0 1 0 0

0 1 58 1 1 0 0 0 0 1 0 1 0 0

0 1 76 1 1 0 0 0 0 1 0 1 0 0

0 1 62 1 1 0 0 0 0 1 0 1 0 0

0 1 62 1 1 0 0 0 0 1 0 1 0 0

0 1 74 1 1 0 0 0 0 1 0 1 0 0

0 1 74 1 1 0 0 0 0 1 0 1 0 0

0 1 71 1 1 0 0 0 0 1 0 1 0 0

0 1 75 1 1 0 0 0 0 1 0 1 0 0

0 1 68 1 1 0 0 0 0 1 0 1 0 0

0 1 47 1 1 0 0 0 0 1 0 1 0 0

0 1 58 1 1 0 0 0 0 1 0 1 0 0

0 1 41 1 1 0 0 0 0 1 0 1 0 0

0 1 77 1 1 0 0 0 0 1 0 1 0 0

0 1 51 1 1 0 0 0 0 1 0 1 3 0

0 1 52 1 1 0 0 0 0 1 0 1 3 0

0 1 57 1 1 0 0 0 0 1 0 1 3 0

0 1 62 1 1 0 0 0 0 1 0 1 0 0

0 1 74 1 1 0 0 0 0 1 0 1 0 0

0 1 50 1 1 0 0 0 0 1 0 1 0 0

0 1 70 1 1 0 0 0 0 1 0 1 0 0

0 1 59 1 1 0 0 0 0 1 0 1 0 0

0 1 74 1 1 0 0 0 0 1 0 1 0 0

0 1 63 1 1 0 0 0 0 1 0 1 0 0

0 1 48 1 1 0 0 0 0 1 0 1 0 0

0 1 64 1 1 0 0 0 0 1 0 1 0 0

0 1 50 1 1 0 0 0 0 1 0 1 0 0

0 1 57 1 1 0 0 0 0 1 0 1 0 0

0 1 71 1 1 0 0 0 0 1 0 1 0 0

0 1 74 1 1 0 0 0 0 1 0 1 0 0

0 1 62 1 1 0 0 0 0 1 0 1 0 0

0 1 57 1 1 0 0 0 0 1 0 1 0 0

0 1 68 1 1 0 0 0 0 1 0 1 0 0

0 1 46 1 1 0 0 0 0 1 0 1 0 0

0 1 70 1 1 0 0 0 0 1 0 1 0 0

0 1 74 1 1 0 0 0 0 1 0 1 2 0

0 1 69 1 1 0 0 0 0 1 0 1 2 0

0 1 61 1 1 0 0 0 0 1 0 1 2 0

0 1 79 1 1 0 0 0 0 1 0 1 2 0

0 1 60 1 1 0 0 0 0 1 0 1 2 0

0 1 77 1 1 0 0 0 0 1 0 1 2 0

0 1 75 1 1 0 0 0 0 1 0 1 2 0

0 1 44 1 1 0 0 0 0 1 0 1 2 0

0 1 59 1 1 0 0 0 0 1 0 1 3 0

0 1 59 1 1 0 0 0 0 1 0 1 3 0

0 1 64 1 1 0 0 0 0 1 0 1 3 0

0 1 70 1 1 0 0 0 0 1 0 1 3 0

0 1 76 1 1 0 0 0 0 1 0 1 3 0

0 1 52 1 1 0 0 0 0 1 0 1 3 0

0 1 77 1 1 0 0 0 0 1 0 1 0 0

0 1 52 1 1 0 0 0 0 1 0 1 3 0

0 1 66 1 1 0 0 0 0 1 0 1 3 0

0 1 58 1 1 0 0 0 0 1 0 1 0 0

0 1 61 1 1 0 0 0 0 1 0 1 4 0

0 1 62 1 1 0 0 0 0 1 0 1 0 0

0 1 49 1 1 0 0 0 0 1 0 1 0 0

0 1 56 1 1 0 0 0 0 1 0 1 0 0

0 1 66 1 1 0 0 0 0 1 0 1 0 0

0 1 64 1 1 0 0 0 0 1 0 1 0 0

0 1 68 1 1 0 0 0 0 1 0 1 0 0

0 1 63 1 1 0 0 0 0 1 0 1 0 0

0 1 79 1 1 0 0 0 0 1 0 1 0 0

0 1 62 1 1 0 0 0 0 1 0 1 0 0

0 1 70 1 1 0 0 0 0 1 0 1 0 0

0 1 59 1 1 0 0 0 0 1 0 1 0 0

0 2 77 1 1 0 0 0 0 1 0 1 0 0

0 2 78 1 1 0 0 0 0 1 0 1 0 0

0 2 77 1 1 0 0 0 0 1 0 1 0 0

0 2 73 1 1 0 0 0 0 1 0 1 0 0

0 2 68 1 1 0 0 0 0 1 0 1 0 0

0 2 69 1 1 0 0 0 0 1 0 1 0 0

0 2 67 1 1 0 0 0 0 1 0 1 0 0

0 2 74 1 1 0 0 0 0 1 0 1 0 0

0 2 71 1 1 0 0 0 0 1 0 1 0 0

0 2 76 1 1 0 0 0 0 1 0 1 0 0

0 2 80 1 1 0 0 0 0 1 0 1 0 0

0 2 76 1 1 0 0 0 0 1 0 1 0 0

0 2 55 1 1 0 0 0 0 1 0 1 0 0

0 2 64 1 1 0 0 0 0 1 0 1 0 0

0 2 77 1 1 0 0 0 0 1 0 1 0 0

0 2 68 1 1 0 0 0 0 1 0 1 0 0

0 2 68 1 1 0 0 0 0 1 0 1 0 0

0 2 77 1 1 0 0 0 0 1 0 1 0 0

0 2 87 1 1 0 0 0 0 1 0 1 0 0

0 2 64 1 1 0 0 0 0 1 0 1 0 0

0 2 60 1 1 0 0 0 0 1 0 1 0 0

0 2 77 1 1 0 0 0 0 1 0 1 0 0

0 2 85 1 1 0 0 0 0 1 0 1 0 0

0 2 75 1 1 0 0 0 0 1 0 1 0 0

0 2 67 1 1 0 0 0 0 1 0 1 0 0

0 2 67 1 1 0 0 0 0 1 0 1 0 0

0 2 57 1 1 0 0 0 0 1 0 1 0 0

0 2 71 1 1 0 0 0 0 1 0 1 0 0

0 2 68 1 1 0 0 0 0 1 0 1 0 0

0 2 82 1 1 0 0 0 0 1 0 1 0 0

0 2 62 1 1 0 0 0 0 1 0 1 0 0

0 2 60 1 1 0 0 0 0 1 0 1 0 0

0 2 65 1 1 0 0 0 0 1 0 1 0 0

0 2 72 1 1 0 0 0 0 1 0 1 0 0

0 2 72 1 1 0 0 0 0 1 0 1 0 0

0 2 60 1 1 0 0 0 0 1 0 1 0 0

0 2 60 1 1 0 0 0 0 1 0 1 0 0

0 2 81 1 1 0 0 0 0 1 0 1 0 0

0 2 72 1 1 0 0 0 0 1 0 1 0 0

0 2 69 1 1 0 0 0 0 1 0 1 0 0

0 2 70 1 1 0 0 0 0 1 0 1 0 0

0 2 74 1 1 0 0 0 0 1 0 1 0 0

0 2 66 1 1 0 0 0 0 1 0 1 0 0

0 2 59 1 1 0 0 0 0 1 0 1 0 0

0 2 70 1 1 0 0 0 0 1 0 1 0 0

0 2 75 1 1 0 0 0 0 1 0 1 0 0

0 2 75 1 1 0 0 0 0 1 0 1 0 0

0 2 73 1 1 0 0 0 0 1 0 1 0 0

0 2 81 1 1 0 0 0 0 1 0 1 0 0

0 2 62 1 1 0 0 0 0 1 0 1 0 0

0 2 54 1 1 0 0 0 0 1 0 1 0 0

0 2 72 1 1 0 0 0 0 1 0 1 0 0

0 1 77 1 0 0 0 0 0 0 0 1 0 0

0 1 53 1 0 0 0 0 0 0 0 1 0 0

0 1 92 1 0 0 0 0 0 0 0 1 0 0

0 1 65 1 0 0 0 0 0 0 0 1 0 0

0 2 65 1 0 0 0 0 0 0 0 1 0 0

0 2 74 1 0 0 0 0 0 0 0 1 0 0

0 1 76 1 0 1 0 0 0 0 0 1 0 0

0 2 67 1 0 1 0 0 0 0 0 1 0 0

0 2 72 1 0 1 0 0 0 0 0 1 0 0

0 1 48 1 0 0 0 0 0 0 0 1 0 0

0 1 47 1 0 0 0 0 0 0 0 1 0 0

0 1 75 1 0 0 0 0 0 0 0 1 0 0

0 1 60 1 0 0 0 0 0 0 0 1 0 0

0 1 71 1 0 0 0 0 0 0 0 1 0 0

0 1 67 1 0 0 0 0 0 0 0 1 0 0

0 1 51 1 0 0 0 0 0 0 0 1 0 0

0 1 71 1 0 0 0 0 0 0 0 1 0 0

0 1 57 1 0 0 0 0 0 0 0 1 0 0

0 1 66 1 0 0 0 0 0 0 0 1 0 0

0 1 45 1 0 0 0 0 0 0 0 1 0 0

0 1 58 1 0 0 0 0 0 0 0 1 0 0

0 1 61 1 0 0 0 0 0 0 0 1 0 0

0 1 63 1 0 0 0 0 0 0 0 1 0 0

0 1 51 1 0 0 0 0 0 0 0 1 0 0

0 1 72 1 0 0 0 0 0 0 0 1 0 0

0 1 76 1 0 0 0 0 0 0 0 1 0 0

0 1 62 1 0 0 0 0 0 0 0 1 0 0

0 1 66 1 0 0 0 0 0 0 0 1 0 0

0 1 48 1 0 0 0 0 0 0 0 1 0 0

0 1 67 1 0 0 0 0 0 0 0 1 0 0

0 1 66 1 0 0 0 0 0 0 0 1 0 0

0 1 70 1 0 0 0 0 0 0 0 1 0 0

0 1 59 1 0 0 0 0 0 0 0 1 0 0

0 1 82 1 0 0 0 0 0 0 0 1 0 0

0 1 62 1 0 0 0 0 0 0 0 1 0 0

0 1 88 1 0 0 0 0 0 0 0 1 0 0

0 1 69 1 0 0 0 0 0 0 0 1 0 0

0 1 58 1 0 0 0 0 0 0 0 1 0 0

0 1 63 1 0 0 0 0 0 0 0 1 0 0

0 1 58 1 0 0 0 0 0 0 0 1 0 0

0 1 70 1 0 0 0 0 0 0 0 1 0 0

0 1 58 1 0 0 0 0 0 0 0 1 0 0

0 1 58 1 0 0 0 0 0 0 0 1 0 0

0 1 58 1 0 0 0 0 0 0 0 1 0 0

0 1 74 1 0 0 0 0 0 0 0 1 0 0

0 1 45 1 0 0 0 0 0 0 0 1 0 0

0 1 35 1 0 0 0 0 0 0 0 1 0 0

0 1 36 1 0 0 0 0 0 0 0 1 0 0

0 1 35 1 0 0 0 0 0 0 0 1 0 0

0 1 35 1 0 0 0 0 0 0 0 1 0 0

0 1 53 1 0 0 0 0 0 0 0 1 0 0

0 1 67 1 0 0 0 0 0 0 0 1 0 0

0 1 54 1 0 0 0 0 0 0 0 1 0 0

0 1 78 1 0 0 0 0 0 0 0 1 0 0

0 1 68 1 0 0 0 0 0 0 0 1 0 0

0 1 59 1 0 0 0 0 0 0 0 1 0 0

0 1 64 1 0 0 0 0 0 0 0 1 0 0

0 1 56 1 0 0 0 0 0 0 0 1 0 0

0 1 68 1 0 0 0 0 0 0 0 1 0 0

0 1 62 1 0 0 0 0 0 0 0 1 0 0

0 1 73 1 0 0 0 0 0 0 0 1 0 0

0 1 73 1 0 0 0 0 0 0 0 1 0 0

0 1 73 1 0 0 0 0 0 0 0 1 0 0

0 1 56 1 0 0 0 0 0 0 0 1 0 0

0 1 77 1 0 0 0 0 0 0 0 1 0 0

0 1 59 1 0 0 0 0 0 0 0 1 0 0

0 1 70 1 0 0 0 0 0 0 0 1 0 0

0 1 35 1 0 0 0 0 0 0 0 1 0 0

0 1 35 1 0 0 0 0 0 0 0 1 0 0

0 1 67 1 0 0 0 0 0 0 0 1 0 0

0 1 78 1 0 0 0 0 0 0 0 1 0 0

0 1 54 1 0 0 0 0 0 0 0 1 0 0

0 1 46 1 0 0 0 0 0 0 0 1 0 0

0 1 65 1 0 0 0 0 0 0 0 1 0 0

0 1 64 1 0 0 0 0 0 0 0 1 0 0

0 1 64 1 0 0 0 0 0 0 0 1 0 0

0 1 43 1 0 0 0 0 0 0 0 1 0 0

0 1 51 1 0 0 0 0 0 0 0 1 0 0

0 1 60 1 0 0 0 0 0 0 0 1 0 0

0 1 61 1 0 0 0 0 0 0 0 1 0 0

0 1 71 1 0 0 0 0 0 0 0 1 0 0

0 1 81 1 0 0 0 0 0 0 0 1 0 0

0 1 62 1 0 0 0 0 0 0 0 1 0 0

0 1 45 1 0 0 0 0 0 0 0 1 0 0

0 1 56 1 0 0 0 0 0 0 0 1 0 0

0 1 57 1 0 0 0 0 0 0 0 1 0 0

0 1 75 1 0 0 0 0 0 0 0 1 0 0

0 1 51 1 0 0 0 0 0 0 0 1 0 0

0 1 57 1 0 0 0 0 0 0 0 1 0 0

0 1 51 1 0 0 0 0 0 0 0 1 0 0

0 1 54 1 0 0 0 0 0 0 0 1 0 0

0 1 60 1 0 0 0 0 0 0 0 1 0 0

0 1 53 1 0 0 0 0 0 0 0 1 0 0

0 1 58 1 0 0 0 0 0 0 0 1 0 0

0 1 46 1 0 0 0 0 0 0 0 1 0 0

0 1 80 1 0 0 0 0 0 0 0 1 0 0

0 1 59 1 0 0 0 0 0 0 0 1 0 0

0 1 80 1 0 0 0 0 0 0 0 1 0 0

0 1 81 1 0 0 0 0 0 0 0 1 0 0

0 1 66 1 0 0 0 0 0 0 0 1 0 0

0 1 75 1 0 0 0 0 0 0 0 1 0 0

0 1 76 1 0 0 0 0 0 0 0 1 0 0

0 1 67 1 0 0 0 0 0 0 0 1 0 0

0 1 53 1 0 0 0 0 0 0 0 1 0 0

0 1 74 1 0 0 0 0 0 0 0 1 0 0

0 1 79 1 0 0 0 0 0 0 0 1 0 0

0 1 79 1 0 0 0 0 0 0 0 1 0 0

0 1 62 1 0 0 0 0 0 0 0 1 0 0

0 1 67 1 0 0 0 0 0 0 0 1 0 0

0 1 76 1 0 0 0 0 0 0 0 1 0 0

0 1 67 1 0 0 0 0 0 0 0 1 0 0

0 1 78 1 0 0 0 0 0 0 0 1 0 0

0 1 74 1 0 0 0 0 0 0 0 1 0 0

0 1 59 1 0 0 0 0 0 0 0 1 0 0

0 1 68 1 0 0 0 0 0 0 0 1 0 0

0 1 57 1 0 0 0 0 0 0 0 1 0 0

0 1 54 1 0 0 0 0 0 0 0 1 0 0

0 1 44 1 0 0 0 0 0 0 0 1 0 0

0 1 77 1 0 0 0 0 0 0 0 1 0 0

0 1 55 1 0 0 0 0 0 0 0 1 0 0

0 1 62 1 0 0 0 0 0 0 0 1 0 0

0 1 72 1 0 0 0 0 0 0 0 1 0 0

0 1 69 1 0 0 0 0 0 0 0 1 0 0

0 1 83 1 0 0 0 0 0 0 0 1 0 0

0 1 55 1 0 0 0 0 0 0 0 1 0 0

0 1 79 1 0 0 0 0 0 0 0 1 0 0

0 1 70 1 0 0 0 0 0 0 0 1 0 0

0 1 47 1 0 0 0 0 0 0 0 1 0 0

0 1 54 1 0 0 0 0 0 0 0 1 0 0

0 1 61 1 0 0 0 0 0 0 0 1 0 0

0 1 55 1 0 0 0 0 0 0 0 1 0 0

0 1 76 1 0 0 0 0 0 0 0 1 0 0

0 1 68 1 0 0 0 0 0 0 0 1 0 0

0 1 59 1 0 0 0 0 0 0 0 1 0 0

0 1 37 1 0 0 0 0 0 0 0 1 0 0

0 1 65 1 0 0 0 0 0 0 0 1 0 0

0 1 76 1 0 0 0 0 0 0 0 1 0 0

0 1 49 1 0 0 0 0 0 0 0 1 0 0

0 1 70 1 0 0 0 0 0 0 0 1 0 0

0 1 61 1 0 0 0 0 0 0 0 1 0 0

0 1 65 1 0 0 0 0 0 0 0 1 0 0

0 1 63 1 0 0 0 0 0 0 0 1 0 0

0 1 79 1 0 0 0 0 0 0 0 1 0 0

0 1 56 1 0 0 0 0 0 0 0 1 0 0

0 1 77 1 0 0 0 0 0 0 0 1 0 0

0 1 72 1 0 0 0 0 0 0 0 1 0 0

0 1 52 1 0 0 0 0 0 0 0 1 0 0

0 1 71 1 0 0 0 0 0 0 0 1 0 0

0 1 72 1 0 0 0 0 0 0 0 1 0 0

0 1 72 1 0 0 0 0 0 0 0 1 0 0

0 1 76 1 0 0 0 0 0 0 0 1 0 0

0 1 72 1 0 0 0 0 0 0 0 1 0 0

0 1 76 1 0 0 0 0 0 0 0 1 0 0

0 1 70 1 0 0 0 0 0 0 0 1 0 0

0 1 67 1 0 0 0 0 0 0 0 1 0 0

0 1 62 1 0 0 0 0 0 0 0 1 0 0

0 1 87 1 0 0 0 0 0 0 0 1 0 0

0 1 78 1 0 0 0 0 0 0 0 1 0 0

0 1 67 1 0 0 0 0 0 0 0 1 0 0

0 1 46 1 0 0 0 0 0 0 0 1 0 0

0 1 59 1 0 0 0 0 0 0 0 1 0 0

0 1 51 1 0 0 0 0 0 0 0 1 0 0

0 1 60 1 0 0 0 0 0 0 0 1 0 0

0 1 68 1 0 0 0 0 0 0 0 1 0 0

0 1 58 1 0 0 0 0 0 0 0 1 0 0

0 1 73 1 0 0 0 0 0 0 0 1 0 0

0 1 53 1 0 0 0 0 0 0 0 1 0 0

0 1 65 1 0 0 0 0 0 0 0 1 0 0

0 1 78 1 0 0 0 0 0 0 0 1 0 0

0 1 70 1 0 0 0 0 0 0 0 1 0 0

0 1 74 1 0 0 0 0 0 0 0 1 0 0

0 1 82 1 0 0 0 0 0 0 0 1 0 0

0 1 64 1 0 0 0 0 0 0 0 1 0 0

0 1 60 1 0 0 0 0 0 0 0 1 0 0

0 1 73 1 0 0 0 0 0 0 0 1 0 0

0 1 41 1 0 0 0 0 0 0 0 1 0 0

0 1 56 1 0 0 0 0 0 0 0 1 0 0

0 1 70 1 0 0 0 0 0 0 0 1 0 0

0 1 65 1 0 0 0 0 0 0 0 1 0 0

0 1 64 1 0 0 0 0 0 0 0 1 0 0

0 1 52 1 0 0 0 0 0 0 0 1 0 0

0 1 52 1 0 0 0 0 0 0 0 1 0 0

0 1 75 1 0 0 0 0 0 0 0 1 0 0

0 1 81 1 0 0 0 0 0 0 0 1 0 0

0 1 76 1 0 0 0 0 0 0 0 1 0 0

0 1 79 1 0 0 0 0 0 0 0 1 0 0

0 1 72 1 0 0 0 0 0 0 0 1 0 0

0 1 72 1 0 0 0 0 0 0 0 1 0 0

0 1 58 1 0 0 0 0 0 0 0 1 0 0

0 1 65 1 0 0 0 0 0 0 0 1 0 0

0 1 67 1 0 0 0 0 0 0 0 1 0 0

0 1 68 1 0 0 0 0 0 0 0 1 0 0

0 1 80 1 0 0 0 0 0 0 0 1 0 0

0 1 72 1 0 0 0 0 0 0 0 1 0 0

0 1 54 1 0 0 0 0 0 0 0 1 0 0

0 1 48 1 0 0 0 0 0 0 0 1 0 0

0 1 82 1 0 0 0 0 0 0 0 1 0 0

0 1 62 1 0 0 0 0 0 0 0 1 0 0

0 1 54 1 0 0 0 0 0 0 0 1 0 0

0 1 72 1 0 0 0 0 0 0 0 1 0 0

0 1 68 1 0 0 0 0 0 0 0 1 0 0

0 1 80 1 0 0 0 0 0 0 0 1 0 0

0 1 83 1 0 0 0 0 0 0 0 1 0 0

0 1 83 1 0 0 0 0 0 0 0 1 0 0

0 1 88 1 0 0 0 0 0 0 0 1 0 0

0 1 65 1 0 0 0 0 0 0 0 1 0 0

0 1 88 1 0 0 0 0 0 0 0 1 0 0

0 1 58 1 0 0 0 0 0 0 0 1 0 0

0 1 59 1 0 0 0 0 0 0 0 1 0 0

0 1 74 1 0 0 0 0 0 0 0 1 0 0

0 1 73 1 0 0 0 0 0 0 0 1 0 0

0 1 52 1 0 0 0 0 0 0 0 1 0 0

0 1 48 1 0 0 0 0 0 0 0 1 0 0

0 1 77 1 0 0 0 0 0 0 0 1 0 0

0 1 78 1 0 0 0 0 0 0 0 1 0 0

0 1 66 1 0 0 0 0 0 0 0 1 0 0

0 1 58 1 0 0 0 0 0 0 0 1 0 0

0 1 57 1 0 0 0 0 0 0 0 1 0 0

0 1 80 1 0 0 0 0 0 0 0 1 0 0

0 1 72 1 0 0 0 0 0 0 0 1 0 0

0 1 55 1 0 0 0 0 0 0 0 1 0 0

0 1 56 1 0 0 0 0 0 0 0 1 0 0

0 1 65 1 0 0 0 0 0 0 0 1 0 0

0 1 82 1 0 0 0 0 0 0 0 1 0 0

0 1 69 1 0 0 0 0 0 0 0 1 0 0

0 1 59 1 0 0 0 0 0 0 0 1 0 0

0 1 66 1 0 0 0 0 0 0 0 1 0 0

0 1 64 1 0 0 0 0 0 0 0 1 0 0

0 1 63 1 0 0 0 0 0 0 0 1 0 0

0 1 75 1 0 0 0 0 0 0 0 1 0 0

0 1 80 1 0 0 0 0 0 0 0 1 0 0

0 1 82 1 0 0 0 0 0 0 0 1 2 0

0 1 80 1 0 0 0 0 0 0 0 1 2 0

0 1 75 1 0 0 0 0 0 0 0 1 2 0

0 1 69 1 0 0 0 0 0 0 0 1 2 0

0 1 79 1 0 0 0 0 0 0 0 1 2 0

0 1 77 1 0 0 0 0 0 0 0 1 3 0

0 1 71 1 0 0 0 0 0 0 0 1 0 0

0 1 79 1 0 0 0 0 0 0 0 1 0 0

0 1 60 1 0 0 0 0 0 0 0 1 0 0

0 1 79 1 0 0 0 0 0 0 0 1 4 0

0 1 77 1 0 0 0 0 0 0 0 1 4 0

0 1 67 1 0 0 0 0 0 0 0 1 4 0

0 1 69 1 0 0 0 0 0 0 0 1 4 0

0 1 84 1 0 0 0 0 0 0 0 1 4 0

0 1 81 1 0 0 0 0 0 0 0 1 4 0

0 1 52 1 0 0 0 0 0 0 0 1 0 0

0 1 78 1 0 0 0 0 0 0 0 1 0 0

0 1 72 1 0 0 0 0 0 0 0 1 0 0

0 1 68 1 0 0 0 0 0 0 0 1 2 0

0 1 66 1 0 0 0 0 0 0 0 1 2 0

0 1 81 1 0 0 0 0 0 0 0 1 2 0

0 1 59 1 0 0 0 0 0 0 0 1 2 0

0 1 58 1 0 0 0 0 0 0 0 1 2 0

0 1 79 1 0 0 0 0 0 0 0 1 2 0

0 1 67 1 0 0 0 0 0 0 0 1 2 0

0 1 67 1 0 0 0 0 0 0 0 1 2 0

0 1 71 1 0 0 0 0 0 0 0 1 2 0

0 1 52 1 0 0 0 0 0 0 0 1 2 0

0 1 75 1 0 0 0 0 0 0 0 1 2 0

0 1 58 1 0 0 0 0 0 0 0 1 2 0

0 1 72 1 0 0 0 0 0 0 0 1 2 0

0 1 84 1 0 0 0 0 0 0 0 1 2 0

0 1 65 1 0 0 0 0 0 0 0 1 2 0

0 1 66 1 0 0 0 0 0 0 0 1 2 0

0 1 63 1 0 0 0 0 0 0 0 1 2 0

0 1 57 1 0 0 0 0 0 0 0 1 2 0

0 1 50 1 0 0 0 0 0 0 0 1 2 0

0 1 55 1 0 0 0 0 0 0 0 1 2 0

0 1 52 1 0 0 0 0 0 0 0 1 2 0

0 1 73 1 0 0 0 0 0 0 0 1 3 0

0 1 60 1 0 0 0 0 0 0 0 1 3 0

0 1 80 1 0 0 0 0 0 0 0 1 3 0

0 1 66 1 0 0 0 0 0 0 0 1 3 0

0 1 71 1 0 0 0 0 0 0 0 1 3 0

0 1 74 1 0 0 0 0 0 0 0 1 3 0

0 1 59 1 0 0 0 0 0 0 0 1 3 0

0 1 71 1 0 0 0 0 0 0 0 1 3 0

0 1 83 1 0 0 0 0 0 0 0 1 2 0

0 1 44 1 0 0 0 0 0 0 0 1 2 0

0 1 83 1 0 0 0 0 0 0 0 1 2 0

0 1 79 1 0 0 0 0 0 0 0 1 2 0

0 1 58 1 0 0 0 0 0 0 0 1 2 0

0 1 57 1 0 0 0 0 0 0 0 1 2 0

0 1 76 1 0 0 0 0 0 0 0 1 2 0

0 1 75 1 0 0 0 0 0 0 0 1 2 0

0 1 55 1 0 0 0 0 0 0 0 1 2 0

0 1 84 1 0 0 0 0 0 0 0 1 2 0

0 1 76 1 0 0 0 0 0 0 0 1 2 0

0 1 66 1 0 0 0 0 0 0 0 1 2 0

0 1 49 1 0 0 0 0 0 0 0 1 2 0

0 1 77 1 0 0 0 0 0 0 0 1 2 0

0 1 78 1 0 0 0 0 0 0 0 1 2 0

0 1 71 1 0 0 0 0 0 0 0 1 2 0

0 1 64 1 0 0 0 0 0 0 0 1 2 0

0 1 50 1 0 0 0 0 0 0 0 1 2 0

0 1 55 1 0 0 0 0 0 0 0 1 2 0

0 1 71 1 0 0 0 0 0 0 0 1 2 0

0 1 62 1 0 0 0 0 0 0 0 1 2 0

0 1 52 1 0 0 0 0 0 0 0 1 2 0

0 1 77 1 0 0 0 0 0 0 0 1 2 0

0 1 60 1 0 0 0 0 0 0 0 1 2 0

0 1 65 1 0 0 0 0 0 0 0 1 2 0

0 1 76 1 0 0 0 0 0 0 0 1 2 0

0 1 73 1 0 0 0 0 0 0 0 1 2 0

0 1 75 1 0 0 0 0 0 0 0 1 2 0

0 1 56 1 0 0 0 0 0 0 0 1 2 0

0 1 65 1 0 0 0 0 0 0 0 1 2 0

0 1 72 1 0 0 0 0 0 0 0 1 2 0

0 1 49 1 0 0 0 0 0 0 0 1 2 0

0 1 71 1 0 0 0 0 0 0 0 1 2 0

0 1 59 1 0 0 0 0 0 0 0 1 2 0

0 1 63 1 0 0 0 0 0 0 0 1 2 0

0 1 62 1 0 0 0 0 0 0 0 1 2 0

0 1 83 1 0 0 0 0 0 0 0 1 2 0

0 1 79 1 0 0 0 0 0 0 0 1 2 0

0 1 86 1 0 0 0 0 0 0 0 1 2 0

0 1 66 1 0 0 0 0 0 0 0 1 2 0

0 1 80 1 0 0 0 0 0 0 0 1 2 0

0 1 61 1 0 0 0 0 0 0 0 1 2 0

0 1 82 1 0 0 0 0 0 0 0 1 2 0

0 1 81 1 0 0 0 0 0 0 0 1 2 0

0 1 74 1 0 0 0 0 0 0 0 1 2 0

0 1 92 1 0 0 0 0 0 0 0 1 2 0

0 1 62 1 0 0 0 0 0 0 0 1 2 0

0 1 79 1 0 0 0 0 0 0 0 1 2 0

0 1 77 1 0 0 0 0 0 0 0 1 2 0

0 1 75 1 0 0 0 0 0 0 0 1 2 0

0 1 63 1 0 0 0 0 0 0 0 1 2 0

0 1 61 1 0 0 0 0 0 0 0 1 2 0

0 1 63 1 0 0 0 0 0 0 0 1 2 0

0 1 79 1 0 0 0 0 0 0 0 1 2 0

0 1 74 1 0 0 0 0 0 0 0 1 2 0

0 1 65 1 0 0 0 0 0 0 0 1 2 0

0 1 73 1 0 0 0 0 0 0 0 1 2 0

0 1 70 1 0 0 0 0 0 0 0 1 0 0

0 1 73 1 0 0 0 0 0 0 0 1 0 0

0 1 86 1 0 0 0 0 0 0 0 1 0 0

0 1 54 1 0 0 0 0 0 0 0 1 0 0

0 1 59 1 0 0 0 0 0 0 0 1 0 0

0 1 56 1 0 0 0 0 0 0 0 1 0 0

0 1 47 1 0 0 0 0 0 0 0 1 0 0

0 1 78 1 0 0 0 0 0 0 0 1 0 0

0 1 63 1 0 0 0 0 0 0 0 1 0 0

0 1 53 1 0 0 0 0 0 0 0 1 0 0

0 1 70 1 0 0 0 0 0 0 0 1 0 0

0 1 53 1 0 0 0 0 0 0 0 1 0 0

0 1 78 1 0 0 0 0 0 0 0 1 0 0

0 1 78 1 0 0 0 0 0 0 0 1 0 0

0 1 65 1 0 0 0 0 0 0 0 1 0 0

0 1 58 1 0 0 0 0 0 0 0 1 0 0

0 1 70 1 0 0 0 0 0 0 0 1 2 0

0 1 59 1 0 0 0 0 0 0 0 1 2 0

0 1 52 1 0 0 0 0 0 0 0 1 2 0

0 1 59 1 0 0 0 0 0 0 0 1 2 0

0 1 69 1 0 0 0 0 0 0 0 1 2 0

0 1 68 1 0 0 0 0 0 0 0 1 2 0

0 1 72 1 0 0 0 0 0 0 0 1 2 0

0 1 72 1 0 0 0 0 0 0 0 1 2 0

0 1 43 1 0 0 0 0 0 0 0 1 0 0

0 1 79 1 0 0 0 0 0 0 0 1 0 0

0 1 44 1 0 0 0 0 0 0 0 1 0 0

0 1 73 1 0 0 0 0 0 0 0 1 0 0

0 1 76 1 0 0 0 0 0 0 0 1 0 0

0 1 41 1 0 0 0 0 0 0 0 1 0 0

0 1 85 1 0 0 0 0 0 0 0 1 0 0

0 1 79 1 0 0 0 0 0 0 0 1 0 0

0 1 54 1 0 0 0 0 0 0 0 1 0 0

0 1 69 1 0 0 0 0 0 0 0 1 0 0

0 1 69 1 0 0 0 0 0 0 0 1 0 0

0 1 67 1 0 0 0 0 0 0 0 1 0 0

0 1 60 1 0 0 0 0 0 0 0 1 0 0

0 1 48 1 0 0 0 0 0 0 0 1 0 0

0 1 76 1 0 0 0 0 0 0 0 1 0 0

0 1 86 1 0 0 0 0 0 0 0 1 0 0

0 1 84 1 0 0 0 0 0 0 0 1 0 0

0 1 51 1 0 0 0 0 0 0 0 1 0 0

0 1 60 1 0 0 0 0 0 0 0 1 0 0

0 1 70 1 0 0 0 0 0 0 0 1 0 0

0 1 63 1 0 0 0 0 0 0 0 1 0 0

0 1 63 1 0 0 0 0 0 0 0 1 0 0

0 1 69 1 0 0 0 0 0 0 0 1 0 0

0 1 70 1 0 0 0 0 0 0 0 1 0 0

0 1 80 1 0 0 0 0 0 0 0 1 0 0

0 1 69 1 0 0 0 0 0 0 0 1 0 0

0 1 67 1 0 0 0 0 0 0 0 1 0 0

0 1 70 1 0 0 0 0 0 0 0 1 0 0

0 1 63 1 0 0 0 0 0 0 0 1 0 0

0 1 79 1 0 0 0 0 0 0 0 1 0 0

0 1 79 1 0 0 0 0 0 0 0 1 0 0

0 1 79 1 0 0 0 0 0 0 0 1 0 0

0 1 70 1 0 0 0 0 0 0 0 1 0 0

0 1 60 1 0 0 0 0 0 0 0 1 0 0

0 1 69 1 0 0 0 0 0 0 0 1 0 0

0 1 55 1 0 0 0 0 0 0 0 1 0 0

0 1 81 1 0 0 0 0 0 0 0 1 0 0

0 1 61 1 0 0 1 0 0 0 0 1 0 0

0 1 61 1 0 0 1 0 0 0 0 1 0 0

0 1 79 1 0 0 0 0 0 0 0 1 0 0

0 1 46 1 0 0 0 0 0 0 0 1 0 0

0 1 62 1 0 0 1 0 0 0 0 1 0 0

0 1 79 1 0 0 1 0 0 0 0 1 0 0

0 1 75 1 0 0 0 0 0 0 0 1 0 0

0 1 60 1 0 0 1 0 0 0 0 1 0 0

0 1 52 1 0 0 1 0 0 0 0 1 0 0

0 1 63 1 0 0 1 0 0 0 0 1 0 0

0 1 67 1 0 0 1 0 0 0 0 1 4 0

0 1 67 1 0 0 1 0 0 0 0 1 0 0

0 1 57 1 0 0 1 0 0 0 0 1 0 0

0 1 57 1 0 0 1 0 0 0 0 1 0 0

0 1 65 1 0 0 1 0 0 0 0 1 0 0

0 1 49 1 0 0 1 0 0 0 0 1 0 0

0 1 54 1 0 0 1 0 0 0 0 1 0 0

0 1 62 1 0 0 1 0 0 0 0 1 0 0

0 1 54 1 0 0 1 0 0 0 0 1 0 0

0 1 52 1 0 0 1 0 0 0 0 1 0 0

0 1 50 1 0 0 1 0 0 0 0 1 0 0

0 1 77 1 0 0 1 0 0 0 0 1 0 0

0 1 75 1 0 0 1 0 0 0 0 1 0 0

0 1 45 1 0 0 1 0 0 0 0 1 0 0

0 1 45 1 0 0 1 0 0 0 0 1 0 0

0 1 55 1 0 0 1 0 0 0 0 1 0 0

0 1 38 1 0 0 1 0 0 0 0 1 0 0

0 1 42 1 0 0 1 0 0 0 0 1 0 0

0 1 53 1 0 0 1 0 0 0 0 1 0 0

0 1 67 1 0 0 0 0 0 0 0 1 0 0

0 1 56 1 0 0 0 0 0 0 0 1 0 0

0 1 72 1 0 0 0 0 0 0 0 1 0 0

0 1 74 1 0 0 0 0 0 0 0 1 0 0

0 1 49 1 0 0 0 0 0 0 0 1 0 0

0 1 60 1 0 0 0 0 0 0 0 1 0 0

0 1 67 1 0 0 0 0 0 0 0 1 0 0

0 1 76 1 0 0 0 0 0 0 0 1 0 0

0 1 54 1 0 0 0 0 0 0 0 1 0 0

0 1 76 1 0 0 0 0 0 0 0 1 0 0

0 1 61 1 0 0 0 0 0 0 0 1 0 0

0 1 69 1 0 0 0 0 0 0 0 1 0 0

0 1 60 1 0 0 0 0 0 0 0 1 0 0

0 1 84 1 0 0 0 0 0 0 0 1 0 0

0 1 55 1 0 0 0 0 0 0 0 1 0 0

0 1 52 1 0 0 0 0 0 0 0 1 0 0

0 1 47 1 0 0 0 0 0 0 0 1 0 0

0 1 65 1 0 0 0 0 0 0 0 1 0 0

0 1 55 1 0 0 0 0 0 0 0 1 0 0

0 1 58 1 0 0 0 0 0 0 0 1 0 0

0 1 50 1 0 0 0 0 0 0 0 1 0 0

0 1 63 1 0 0 0 0 0 0 0 1 0 0

0 1 57 1 0 0 0 0 0 0 0 1 0 0

0 1 50 1 0 0 0 0 0 0 0 1 0 0

0 1 65 1 0 0 0 0 0 0 0 1 0 0

0 1 57 1 0 0 0 0 0 0 0 1 0 0

0 1 76 1 0 0 0 0 0 0 0 1 0 0

0 1 53 1 0 0 0 0 0 0 0 1 0 0

0 1 54 1 0 0 0 0 0 0 0 1 0 0

0 1 52 1 0 0 0 0 0 0 0 1 0 0

0 1 53 1 0 0 0 0 0 0 0 1 0 0

0 1 75 1 0 0 0 0 0 0 0 1 0 0

0 1 49 1 0 0 0 0 0 0 0 1 0 0

0 1 44 1 0 0 0 0 0 0 0 1 0 0

0 1 63 1 0 0 0 0 0 0 0 1 0 0

0 1 61 1 0 0 0 0 0 0 0 1 0 0

0 1 55 1 0 0 0 0 0 0 0 1 0 0

0 1 77 1 0 0 0 0 0 0 0 1 0 0

0 1 61 1 0 0 0 0 0 0 0 1 0 0

0 1 69 1 0 0 0 0 0 0 0 1 0 0

0 1 45 1 0 0 0 0 0 0 0 1 0 0

0 1 49 1 0 0 0 0 0 0 0 1 0 0

0 1 76 1 0 0 0 0 0 0 0 1 0 0

0 1 70 1 0 0 0 0 0 0 0 1 0 0

0 1 57 1 0 0 0 0 0 0 0 1 0 0

0 1 68 1 0 0 0 0 0 0 0 1 0 0

0 1 60 1 0 0 0 0 0 0 0 1 0 0

0 1 54 1 0 0 0 0 0 0 0 1 0 0

0 1 58 1 0 0 0 0 0 0 0 1 0 0

0 1 43 1 0 0 0 0 0 0 0 1 0 0

0 1 68 1 0 0 0 0 0 0 0 1 3 0

0 1 70 1 0 0 0 0 0 0 0 1 3 0

0 1 56 1 0 0 0 0 0 0 0 1 3 0

0 1 74 1 0 0 0 0 0 0 0 1 3 0

0 1 57 1 0 0 0 0 0 0 0 1 3 0

0 1 55 1 0 0 0 0 0 0 0 1 3 0

0 1 52 1 0 0 0 0 0 0 0 1 3 0

0 1 62 1 0 0 0 0 0 0 0 1 3 0

0 1 50 1 0 0 0 0 0 0 0 1 4 0

0 1 63 1 0 0 0 0 0 0 0 1 4 0

0 1 53 1 0 0 0 0 0 0 0 1 4 0

0 1 45 1 0 0 0 0 0 0 0 1 4 0

0 1 74 1 0 0 0 0 0 0 0 1 0 0

0 1 45 1 0 0 0 0 0 0 0 1 0 0

0 1 46 1 0 0 0 0 0 0 0 1 4 0

0 1 54 1 0 0 0 0 0 0 0 1 4 0

0 1 68 1 0 0 0 0 0 0 0 1 0 0

0 1 71 1 0 0 0 0 0 0 0 1 0 0

0 1 60 1 0 0 0 0 0 0 0 1 0 0

0 1 70 1 0 0 0 0 0 0 0 1 0 0

0 1 55 1 0 0 0 0 0 0 0 1 0 0

0 1 56 1 0 0 0 0 0 0 0 1 0 0

0 1 59 1 0 0 0 0 0 0 0 1 0 0

0 1 58 1 0 0 0 0 0 0 0 1 0 0

0 1 57 1 0 0 0 0 0 0 0 1 0 0

0 1 52 1 0 0 0 0 0 0 0 1 0 0

0 1 57 1 0 0 0 0 0 0 0 1 0 0

0 1 54 1 0 0 0 0 0 0 0 1 0 0

0 1 46 1 0 0 0 0 0 0 0 1 0 0

0 1 83 1 0 0 0 0 0 0 0 1 0 0

0 1 79 1 0 0 0 0 0 0 0 1 0 0

0 1 59 1 0 0 0 0 0 0 0 1 0 0

0 1 78 1 0 0 0 0 0 0 0 1 0 0

0 1 84 1 0 0 0 0 0 0 0 1 0 0

0 1 58 1 0 0 0 0 0 0 0 1 0 0

0 1 55 1 0 0 0 0 0 0 0 1 0 0

0 1 75 1 0 0 0 0 0 0 0 1 0 0

0 1 66 1 0 0 0 0 0 0 0 1 0 0

0 1 58 1 0 0 0 0 0 0 0 1 0 0

0 1 71 1 0 0 0 0 0 0 0 1 0 0

0 1 40 1 0 0 0 0 0 0 0 1 0 0

0 1 61 1 0 0 0 0 0 0 0 1 0 0

0 1 63 1 0 0 0 0 0 0 0 1 0 0

0 1 44 1 0 0 0 0 0 0 0 1 0 0

0 1 53 1 0 0 0 0 0 0 0 1 0 0

0 1 50 1 0 0 0 0 0 0 0 1 0 0

0 1 48 1 0 0 0 0 0 0 0 1 0 0

0 1 69 1 0 0 0 0 0 0 0 1 0 0

0 1 48 1 0 0 0 0 0 0 0 1 0 0

0 1 62 1 0 0 0 0 0 0 0 1 0 0

0 1 52 1 0 0 0 0 0 0 0 1 0 0

0 1 57 1 0 0 0 0 0 0 0 1 0 0

0 1 63 1 0 0 0 0 0 0 0 1 0 0

0 1 67 1 0 0 0 0 0 0 0 1 0 0

0 1 49 1 0 0 0 0 0 0 0 1 0 0

0 1 82 1 0 0 0 0 0 0 0 1 0 0

0 1 63 1 0 0 0 0 0 0 0 1 0 0

0 1 55 1 0 0 0 0 0 0 0 1 0 0

0 1 63 1 0 0 0 0 0 0 0 1 0 0

0 1 73 1 0 0 0 0 0 0 0 1 0 0

0 1 76 1 0 0 0 0 0 0 0 1 0 0

0 1 57 1 0 0 0 0 0 0 0 1 0 0

0 1 60 1 0 0 0 0 0 0 0 1 0 0

0 1 73 1 0 0 0 0 0 0 0 1 0 0

0 1 66 1 0 0 0 0 0 0 0 1 0 0

0 1 73 1 0 0 0 0 0 0 0 1 0 0

0 1 62 1 0 0 0 0 0 0 0 1 0 0

0 1 62 1 0 0 0 0 0 0 0 1 0 0

0 1 43 1 0 0 0 0 0 0 0 1 0 0

0 1 52 1 0 0 0 0 0 0 0 1 0 0

0 1 51 1 0 0 0 0 0 0 0 1 0 0

0 1 60 1 0 0 0 0 0 0 0 1 0 0

0 1 58 1 0 0 0 0 0 0 0 1 0 0

0 1 75 1 0 0 0 0 0 0 0 1 0 0

0 1 65 1 0 0 0 0 0 0 0 1 0 0

0 1 66 1 0 0 0 0 0 0 0 1 0 0

0 1 57 1 0 0 0 0 0 0 0 1 0 0

0 1 71 1 0 0 0 0 0 0 0 1 0 0

0 1 54 1 0 0 0 0 0 0 0 1 0 0

0 1 67 1 0 0 0 0 0 0 0 1 0 0

0 1 67 1 0 0 0 0 0 0 0 1 0 0

0 1 84 1 0 0 0 0 0 0 0 1 0 0

0 1 57 1 0 0 0 0 0 0 0 1 0 0

0 1 77 1 0 0 0 0 0 0 0 1 0 0

0 1 60 1 0 0 0 0 0 0 0 1 0 0

0 1 84 1 0 0 0 0 0 0 0 1 0 0

0 1 69 1 0 0 0 0 0 0 0 1 0 0

0 1 67 1 0 0 0 0 0 0 0 1 0 0

0 1 46 1 0 0 0 0 0 0 0 1 0 0

0 1 50 1 0 0 0 0 0 0 0 1 0 0

0 1 53 1 0 0 0 0 0 0 0 1 0 0

0 1 67 1 0 0 0 0 0 0 0 1 0 0

0 1 65 1 0 0 0 0 0 0 0 1 0 0

0 1 69 1 0 0 0 0 0 0 0 1 2 0

0 1 78 1 0 0 1 0 0 0 0 1 0 0

0 1 65 1 0 0 0 0 0 0 0 1 0 0

0 1 45 1 0 0 0 0 0 0 0 1 0 0

0 1 76 1 0 0 0 0 0 0 0 1 0 0

0 1 48 1 0 0 0 0 0 0 0 1 0 0

0 1 52 1 0 0 0 0 0 0 0 1 0 0

0 1 40 1 0 0 0 0 0 0 0 1 0 0

0 1 80 1 0 0 0 0 0 0 0 1 0 0

0 1 56 1 0 0 0 0 0 0 0 1 0 0

0 1 53 1 0 0 0 0 0 0 0 1 0 0

0 1 55 1 0 0 0 0 0 0 0 1 0 0

0 1 72 1 0 0 0 0 0 0 0 1 0 0

0 1 59 1 0 0 0 0 0 0 0 1 0 0

0 1 77 1 0 0 0 0 0 0 0 1 0 0

0 1 59 1 0 0 0 0 0 0 0 1 0 0

0 1 76 1 0 0 0 0 0 0 0 1 0 0

0 1 56 1 0 0 0 0 0 0 0 1 0 0

0 1 55 1 0 0 0 0 0 0 0 1 0 0

0 1 75 1 0 0 1 0 0 0 0 1 0 0

0 1 45 1 0 0 1 0 0 0 0 1 0 0

0 1 56 1 0 0 1 0 0 0 0 1 0 0

0 1 58 1 0 0 1 0 0 0 0 1 0 0

0 1 74 1 0 0 1 0 0 0 0 1 0 0

0 1 66 1 0 0 1 0 0 0 0 1 0 0

0 1 60 1 0 0 0 0 0 0 0 1 0 0

0 1 49 1 0 0 0 0 0 0 0 1 0 0

0 1 83 1 0 0 0 0 0 0 0 1 0 0

0 1 64 1 0 0 0 0 0 0 0 1 0 0

0 1 61 1 0 0 0 0 0 0 0 1 0 0

0 1 77 1 0 0 0 0 0 0 0 1 0 0

0 1 64 1 0 0 0 0 0 0 0 1 0 0

0 1 46 1 0 0 0 0 0 0 0 1 0 0

0 1 63 1 0 0 0 0 0 0 0 1 0 0

0 1 62 1 0 0 0 0 0 0 0 1 0 0

0 1 74 1 0 0 0 0 0 0 0 1 0 0

0 1 72 1 0 0 0 0 0 0 0 1 0 0

0 1 54 1 0 0 0 0 0 0 0 1 0 0

0 1 58 1 0 0 0 0 0 0 0 1 0 0

0 1 87 1 0 0 0 0 0 0 0 1 0 0

0 1 66 1 0 0 0 0 0 0 0 1 0 0

0 1 77 1 0 0 0 0 0 0 0 1 0 0

0 1 59 1 0 0 0 0 0 0 0 1 0 0

0 1 55 1 0 0 0 0 0 0 0 1 0 0

0 1 59 1 0 0 0 0 0 0 0 1 0 0

0 1 59 1 0 0 0 0 0 0 0 1 0 0

0 1 66 1 0 0 0 0 0 0 0 1 0 0

0 1 56 1 0 0 0 0 0 0 0 1 0 0

0 1 61 1 0 0 0 0 0 0 0 1 0 0

0 1 47 1 0 0 0 0 0 0 0 1 0 0

0 1 65 1 0 0 0 0 0 0 0 1 0 0

0 1 70 1 0 0 0 0 0 0 0 1 0 0

0 1 60 1 0 0 0 0 0 0 0 1 0 0

0 1 44 1 0 0 0 0 0 0 0 1 0 0

0 1 74 1 0 0 0 0 0 0 0 1 0 0

0 1 59 1 0 0 0 0 0 0 0 1 0 0

0 1 62 1 0 0 0 0 0 0 0 1 0 0

0 1 37 1 0 0 0 0 0 0 0 1 0 0

0 1 72 1 0 0 0 0 0 0 0 1 0 0

0 1 88 1 0 0 0 0 0 0 0 1 0 0

0 1 56 1 0 0 0 0 0 0 0 1 0 0

0 1 71 1 0 0 0 0 0 0 0 1 0 0

0 1 79 1 0 0 0 0 0 0 0 1 0 0

0 1 69 1 0 0 0 0 0 0 0 1 0 0

0 1 59 1 0 0 0 0 0 0 0 1 0 0

0 1 71 1 0 0 0 0 0 0 0 1 0 0

0 1 63 1 0 0 0 0 0 0 0 1 0 0

0 1 52 1 0 0 0 0 0 0 0 1 0 0

0 1 48 1 0 0 0 0 0 0 0 1 0 0

0 1 52 1 0 0 0 0 0 0 0 1 0 0

0 1 75 1 0 0 0 0 0 0 0 1 0 0

0 1 29 1 0 0 0 0 0 0 0 1 0 0

0 1 81 1 0 0 0 0 0 0 0 1 0 0

0 1 47 1 0 0 0 0 0 0 0 1 0 0

0 1 60 1 0 0 0 0 0 0 0 1 0 0

0 1 45 1 0 0 0 0 0 0 0 1 0 0

0 1 55 1 0 0 0 0 0 0 0 1 0 0

0 1 59 1 0 0 0 0 0 0 0 1 0 0

0 1 40 1 0 0 0 0 0 0 0 1 0 0

0 1 80 1 0 0 0 0 0 0 0 1 0 0

0 1 39 1 0 0 0 0 0 0 0 1 0 0

0 1 86 1 0 0 0 0 0 0 0 1 0 0

0 1 58 1 0 0 0 0 0 0 0 1 0 0

0 1 60 1 0 0 0 0 0 0 0 1 0 0

0 1 67 1 0 0 0 0 0 0 0 1 0 0

0 1 73 1 0 0 0 0 0 0 0 1 0 0

0 1 75 1 0 0 0 0 0 0 0 1 0 0

0 1 61 1 0 0 0 0 0 0 0 1 0 0

0 1 50 1 0 0 0 0 0 0 0 1 0 0

0 1 86 1 0 0 0 0 0 0 0 1 0 0

0 1 64 1 0 0 0 0 0 0 0 1 0 0

0 1 76 1 0 0 0 0 0 0 0 1 0 0

0 1 72 1 0 0 0 0 0 0 0 1 0 0

0 1 56 1 0 0 0 0 0 0 0 1 0 0

0 1 58 1 0 0 0 0 0 0 0 1 0 0

0 1 64 1 0 0 0 0 0 0 0 1 0 0

0 1 35 1 0 0 0 0 0 0 0 1 0 0

0 1 43 1 0 0 0 0 0 0 0 1 0 0

0 1 43 1 0 0 0 0 0 0 0 1 0 0

0 1 53 1 0 0 0 0 0 0 0 1 0 0

0 1 75 1 0 0 0 0 0 0 0 1 0 0

0 1 49 1 0 0 0 0 0 0 0 1 0 0

0 1 49 1 0 0 0 0 0 0 0 1 0 0

0 1 40 1 0 0 0 0 0 0 0 1 0 0

0 1 48 1 0 0 0 0 0 0 0 1 0 0

0 1 59 1 0 0 0 0 0 0 0 1 0 0

0 1 78 1 0 0 0 0 0 0 0 1 0 0

0 1 46 1 0 0 0 0 0 0 0 1 0 0

0 1 70 1 0 0 0 0 0 0 0 1 0 0

0 1 49 1 0 0 0 0 0 0 0 1 0 0

0 1 56 1 0 0 0 0 0 0 0 1 0 0

0 1 58 1 0 0 0 0 0 0 0 1 0 0

0 1 64 1 0 0 0 0 0 0 0 1 0 0

0 1 52 1 0 0 0 0 0 0 0 1 0 0

0 1 48 1 0 0 0 0 0 0 0 1 0 0

0 1 54 1 0 0 0 0 0 0 0 1 0 0

0 1 55 1 0 0 0 0 0 0 0 1 0 0

0 1 47 1 0 0 0 0 0 0 0 1 0 0

0 1 76 1 0 0 0 0 0 0 0 1 0 0

0 1 70 1 0 0 0 0 0 0 0 1 0 0

0 1 72 1 0 0 0 0 0 0 0 1 0 0

0 1 82 1 0 0 0 0 0 0 0 1 0 0

0 1 55 1 0 0 0 0 0 0 0 1 0 0

0 1 48 1 0 0 0 0 0 0 0 1 0 0

0 1 68 1 0 0 0 0 0 0 0 1 0 0

0 1 50 1 0 0 0 0 0 0 0 1 0 0

0 1 59 1 0 0 0 0 0 0 0 1 0 0

0 1 66 1 0 0 0 0 0 0 0 1 0 0

0 1 58 1 0 0 0 0 0 0 0 1 0 0

0 1 59 1 0 0 0 0 0 0 0 1 0 0

0 1 60 1 0 0 0 0 0 0 0 1 0 0

0 1 60 1 0 0 0 0 0 0 0 1 0 0

0 1 58 1 0 0 0 0 0 0 0 1 0 0

0 1 42 1 0 0 0 0 0 0 0 1 0 0

0 1 48 1 0 0 0 0 0 0 0 1 0 0

0 1 49 1 0 0 0 0 0 0 0 1 0 0

0 1 43 1 0 0 0 0 0 0 0 1 0 0

0 1 51 1 0 0 0 0 0 0 0 1 0 0

0 1 69 1 0 0 0 0 0 0 0 1 0 0

0 1 55 1 0 0 0 0 0 0 0 1 0 0

0 1 86 1 0 0 0 0 0 0 0 1 0 0

0 1 73 1 0 0 0 0 0 0 0 1 0 0

0 1 49 1 0 0 0 0 0 0 0 1 0 0

0 1 45 1 0 0 0 0 0 0 0 1 0 0

0 1 47 1 0 0 0 0 0 0 0 1 0 0

0 1 46 1 0 0 0 0 0 0 0 1 0 0

0 1 67 1 0 0 0 0 0 0 0 1 0 0

0 1 57 1 0 0 0 0 0 0 0 1 0 0

0 1 57 1 0 0 0 0 0 0 0 1 0 0

0 1 66 1 0 0 0 0 0 0 0 1 0 0

0 1 57 1 0 0 0 0 0 0 0 1 0 0

0 1 64 1 0 0 0 0 0 0 0 1 0 0

0 1 65 1 0 0 0 0 0 0 0 1 0 0

0 1 64 1 0 0 0 0 0 0 0 1 0 0

0 1 35 1 0 0 0 0 0 0 0 1 0 0

0 1 54 1 0 0 0 0 0 0 0 1 0 0

0 1 55 1 0 0 0 0 0 0 0 1 0 0

0 1 56 1 0 0 0 0 0 0 0 1 0 0

0 1 81 1 0 0 0 0 0 0 0 1 0 0

0 1 77 1 0 0 0 0 0 0 0 1 0 0

0 1 60 1 0 0 0 0 0 0 0 1 0 0

0 1 66 1 0 0 0 0 0 0 0 1 0 0

0 1 74 1 0 0 0 0 0 0 0 1 0 0

0 1 79 1 0 0 0 0 0 0 0 1 0 0

0 1 53 1 0 0 0 0 0 0 0 1 0 0

0 1 50 1 0 0 0 0 0 0 0 1 0 0

0 1 54 1 0 0 0 0 0 0 0 1 0 0

0 1 41 1 0 0 0 0 0 0 0 1 0 0

0 1 82 1 0 0 0 0 0 0 0 1 0 0

0 1 62 1 0 0 0 0 0 0 0 1 0 0

0 1 65 1 0 0 0 0 0 0 0 1 0 0

0 1 50 1 0 0 0 0 0 0 0 1 0 0

0 1 57 1 0 0 0 0 0 0 0 1 0 0

0 1 62 1 0 0 0 0 0 0 0 1 0 0

0 1 48 1 0 0 0 0 0 0 0 1 0 0

0 1 52 1 0 0 0 0 0 0 0 1 0 0

0 1 59 1 0 0 0 0 0 0 0 1 0 0

0 1 64 1 0 0 0 0 0 0 0 1 0 0

0 1 48 1 0 0 0 0 0 0 0 1 0 0

0 1 51 1 0 0 0 0 0 0 0 1 0 0

0 1 67 1 0 0 0 0 0 0 0 1 0 0

0 1 54 1 0 0 0 0 0 0 0 1 0 0

0 1 60 1 0 0 0 0 0 0 0 1 0 0

0 1 70 1 0 0 0 0 0 0 0 1 0 0

0 1 66 1 0 0 0 0 0 0 0 1 0 0

0 1 52 1 0 0 0 0 0 0 0 1 0 0

0 1 58 1 0 0 0 0 0 0 0 1 0 0

0 1 68 1 0 0 0 0 0 0 0 1 0 0

0 1 69 1 0 0 0 0 0 0 0 1 0 0

0 1 78 1 0 0 0 0 0 0 0 1 0 0

0 1 58 1 0 0 0 0 0 0 0 1 0 0

0 1 69 1 0 0 0 0 0 0 0 1 0 0

0 1 69 1 0 0 0 0 0 0 0 1 0 0

0 1 60 1 0 0 0 0 0 0 0 1 0 0

0 1 64 1 0 0 0 0 0 0 0 1 0 0

0 1 57 1 0 0 0 0 0 0 0 1 0 0

0 1 68 1 0 0 0 0 0 0 0 1 0 0

0 1 45 1 0 0 0 0 0 0 0 1 0 0

0 1 63 1 0 0 0 0 0 0 0 1 0 0

0 1 61 1 0 0 0 0 0 0 0 1 0 0

0 1 63 1 0 0 0 0 0 0 0 1 0 0

0 1 56 1 0 0 0 0 0 0 0 1 0 0

0 1 64 1 0 0 0 0 0 0 0 1 0 0

0 1 79 1 0 0 0 0 0 0 0 1 0 0

0 1 75 1 0 0 0 0 0 0 0 1 0 0

0 1 82 1 0 0 0 0 0 0 0 1 0 0

0 1 71 1 0 0 0 0 0 0 0 1 0 0

0 1 83 1 0 0 0 0 0 0 0 1 1 0

0 1 68 1 0 0 0 0 0 0 0 1 1 0

0 1 39 1 0 0 0 0 0 0 0 1 1 0

0 1 68 1 0 0 0 0 0 0 0 1 2 0

0 1 51 1 0 0 0 0 0 0 0 1 2 0

0 1 70 1 0 0 0 0 0 0 0 1 3 0

0 1 57 1 0 0 0 0 0 0 0 1 4 0

0 1 60 1 0 0 0 0 0 0 0 1 0 0

0 1 68 1 0 0 0 0 0 0 0 1 0 0

0 1 68 1 0 0 0 0 0 0 0 1 0 0

0 1 60 1 0 0 0 0 0 0 0 1 2 0

0 1 55 1 0 0 0 0 0 0 0 1 0 0

0 1 47 1 0 0 0 0 0 0 0 1 0 0

0 1 51 1 0 0 0 0 0 0 0 1 0 0

0 1 53 1 0 0 0 0 0 0 0 1 0 0

0 1 66 1 0 0 0 0 0 0 0 1 0 0

0 1 79 1 0 0 0 0 0 0 0 1 0 0

0 1 77 1 0 0 0 0 0 0 0 1 0 0

0 1 76 1 0 0 0 0 0 0 0 1 0 0

0 1 53 1 0 0 0 0 0 0 0 1 0 0

0 1 68 1 0 0 0 0 0 0 0 1 0 0

0 1 54 1 0 0 0 0 0 0 0 1 0 0

0 1 58 1 0 0 0 0 0 0 0 1 0 0

0 1 66 1 0 0 0 0 0 0 0 1 0 0

0 1 60 1 0 0 0 0 0 0 0 1 0 0

0 1 66 1 0 0 0 0 0 0 0 1 0 0

0 1 73 1 0 0 0 0 0 0 0 1 0 0

0 1 58 1 0 0 0 0 0 0 0 1 0 0

0 1 72 1 0 0 0 0 0 0 0 1 0 0

0 1 79 1 0 0 0 0 0 0 0 1 0 0

0 1 55 1 0 0 0 0 0 0 0 1 0 0

0 1 64 1 0 0 0 0 0 0 0 1 0 0

0 1 72 1 0 0 0 0 0 0 0 1 0 0

0 1 75 1 0 0 0 0 0 0 0 1 0 0

0 1 50 1 0 0 0 0 0 0 0 1 0 0

0 1 52 1 0 0 0 0 0 0 0 1 0 0

0 1 48 1 0 0 0 0 0 0 0 1 0 0

0 1 67 1 0 0 0 0 0 0 0 1 0 0

0 1 63 1 0 0 0 0 0 0 0 1 0 0

0 1 72 1 0 0 0 0 0 0 0 1 0 0

0 1 71 1 0 0 0 0 0 0 0 1 0 0

0 1 54 1 0 0 0 0 0 0 0 1 0 0

0 1 65 1 0 0 0 0 0 0 0 1 0 0

0 1 54 1 0 0 0 0 0 0 0 1 0 0

0 1 76 1 0 0 0 0 0 0 0 1 0 0

0 1 77 1 0 0 0 0 0 0 0 1 0 0

0 1 63 1 0 0 0 0 0 0 0 1 0 0

0 1 75 1 0 0 0 0 0 0 0 1 0 0

0 1 77 1 0 0 0 0 0 0 0 1 0 0

0 1 82 1 0 0 0 0 0 0 0 1 0 0

0 1 57 1 0 0 0 0 0 0 0 1 0 0

0 1 68 1 0 0 0 0 0 0 0 1 0 0

0 1 79 1 0 0 0 0 0 0 0 1 0 0

0 1 59 1 0 0 0 0 0 0 0 1 0 0

0 1 52 1 0 0 0 0 0 0 0 1 0 0

0 1 66 1 0 0 0 0 0 0 0 1 0 0

0 1 65 1 0 0 0 0 0 0 0 1 0 0

0 1 61 1 0 0 0 0 0 0 0 1 0 0

0 1 74 1 0 0 0 0 0 0 0 1 0 0

0 1 66 1 0 0 0 0 0 0 0 1 0 0

0 1 67 1 0 0 0 0 0 0 0 1 0 0

0 1 67 1 0 0 0 0 0 0 0 1 0 0

0 1 37 1 0 0 0 0 0 0 0 1 0 0

0 1 71 1 0 0 0 0 0 0 0 1 0 0

0 1 82 1 0 0 0 0 0 0 0 1 0 0

0 1 79 1 0 0 0 0 0 0 0 1 0 0

0 1 75 1 0 0 0 0 0 0 0 1 0 0

0 1 74 1 0 0 0 0 0 0 0 1 0 0

0 1 70 1 0 0 0 0 0 0 0 1 0 0

0 1 66 1 0 0 0 0 0 0 0 1 0 0

0 1 57 1 0 0 0 0 0 0 0 1 0 0

0 1 78 1 0 0 0 0 0 0 0 1 0 0

0 1 74 1 0 0 0 0 0 0 0 1 0 0

0 1 63 1 0 0 0 0 0 0 0 1 0 0

0 1 93 1 0 0 0 0 0 0 0 1 0 0

0 1 66 1 0 0 0 0 0 0 0 1 0 0

0 1 77 1 0 0 0 0 0 0 0 1 0 0

0 1 79 1 0 0 0 0 0 0 0 1 0 0

0 1 71 1 0 0 0 0 0 0 0 1 0 0

0 1 75 1 0 0 0 0 0 0 0 1 0 0

0 1 72 1 0 0 0 0 0 0 0 1 0 0

0 1 57 1 0 0 0 0 0 0 0 1 0 0

0 1 85 1 0 0 0 0 0 0 0 1 0 0

0 1 82 1 0 0 0 0 0 0 0 1 0 0

0 1 63 1 0 0 0 0 0 0 0 1 0 0

0 1 73 1 0 0 0 0 0 0 0 1 0 0

0 1 66 1 0 0 0 0 0 0 0 1 0 0

0 1 57 1 0 0 0 0 0 0 0 1 0 0

0 1 67 1 0 0 0 0 0 0 0 1 0 0

0 1 74 1 0 0 0 0 0 0 0 1 0 0

0 1 62 1 0 0 0 0 0 0 0 1 0 0

0 1 76 1 0 0 0 0 0 0 0 1 0 0

0 1 78 1 0 0 0 0 0 0 0 1 0 0

0 1 70 1 0 0 0 0 0 0 0 1 0 0

0 1 43 1 0 0 0 0 0 0 0 1 0 0

0 1 73 1 0 0 0 0 0 0 0 1 0 0

0 1 66 1 0 0 0 0 0 0 0 1 0 0

0 1 67 1 0 0 0 0 0 0 0 1 0 0

0 1 66 1 0 0 0 0 0 0 0 1 0 0

0 1 50 1 0 0 0 0 0 0 0 1 0 0

0 1 50 1 0 0 0 0 0 0 0 1 0 0

0 1 55 1 0 0 0 0 0 0 0 1 0 0

0 1 51 1 0 0 0 0 0 0 0 1 0 0

0 1 67 1 0 0 0 0 0 0 0 1 0 0

0 1 41 1 0 0 0 0 0 0 0 1 0 0

0 1 75 1 0 0 0 0 0 0 0 1 0 0

0 1 53 1 0 0 0 0 0 0 0 1 0 0

0 1 67 1 0 0 0 0 0 0 0 1 0 0

0 1 58 1 0 0 0 0 0 0 0 1 0 0

0 1 73 1 0 0 0 0 0 0 0 1 0 0

0 1 60 1 0 0 0 0 0 0 0 1 0 0

0 1 47 1 0 0 0 0 0 0 0 1 0 0

0 1 62 1 0 0 0 0 0 0 0 1 0 0

0 1 64 1 0 0 0 0 0 0 0 1 0 0

0 1 50 1 0 0 0 0 0 0 0 1 0 0

0 1 74 1 0 0 0 0 0 0 0 1 0 0

0 1 51 1 0 0 0 0 0 0 0 1 2 0

0 1 66 1 0 0 0 0 0 0 0 1 3 0

0 1 71 1 0 0 0 0 0 0 0 1 3 0

0 1 54 1 0 0 0 0 0 0 0 1 3 0

0 1 49 1 0 0 0 0 0 0 0 1 0 0

0 1 61 1 0 0 0 0 0 0 0 1 0 0

0 1 68 1 0 0 0 0 0 0 0 1 0 0

0 1 62 1 0 0 0 0 0 0 0 1 0 0

0 1 72 1 0 0 0 0 0 0 0 1 0 0

0 1 48 1 0 0 0 0 0 0 0 1 0 0

0 1 59 1 0 0 0 0 0 0 0 1 0 0

0 1 69 1 0 0 0 0 0 0 0 1 0 0

0 1 70 1 0 0 0 0 0 0 0 1 0 0

0 1 74 1 0 0 0 0 0 0 0 1 0 0

0 1 50 1 0 0 0 0 0 0 0 1 0 0

0 1 70 1 0 0 0 0 0 0 0 1 0 0

0 1 67 1 0 0 0 0 0 0 0 1 0 0

0 1 69 1 0 0 0 0 0 0 0 1 0 0

0 1 61 1 0 0 0 0 0 0 0 1 0 0

0 1 74 1 0 0 0 0 0 0 0 1 0 0

0 1 74 1 0 0 0 0 0 0 0 1 0 0

0 1 77 1 0 0 0 0 0 0 0 1 0 0

0 1 74 1 0 0 0 0 0 0 0 1 0 0

0 1 72 1 0 0 0 0 0 0 0 1 0 0

0 1 29 1 0 0 0 0 0 0 0 1 0 0

0 1 69 1 0 0 0 0 0 0 0 1 0 0

0 1 42 1 0 0 0 0 0 0 0 1 0 0

0 1 42 1 0 0 0 0 0 0 0 1 0 0

0 1 51 1 0 0 0 0 0 0 0 1 0 0

0 1 57 1 0 0 0 0 0 0 0 1 0 0

0 1 52 1 0 0 0 0 0 0 0 1 0 0

0 1 50 1 0 0 0 0 0 0 0 1 0 0

0 1 70 1 0 0 0 0 0 0 0 1 0 0

0 1 70 1 0 0 0 0 0 0 0 1 0 0

0 1 44 1 0 0 0 0 0 0 0 1 0 0

0 1 38 1 0 0 0 0 0 0 0 1 0 0

0 1 84 1 0 0 0 0 0 0 0 1 0 0

0 1 68 1 0 0 0 0 0 0 0 1 0 0

0 1 59 1 0 0 0 0 0 0 0 1 0 0

0 1 54 1 0 0 0 0 0 0 0 1 0 0

0 1 51 1 0 0 0 0 0 0 0 1 0 0

0 1 61 1 0 0 0 0 0 0 0 1 0 0

0 1 63 1 0 0 0 0 0 0 0 1 0 0

0 1 49 1 0 0 0 0 0 0 0 1 0 0

0 1 76 1 0 0 0 0 0 0 0 1 0 0

0 1 53 1 0 0 0 0 0 0 0 1 0 0

0 1 57 1 0 0 0 0 0 0 0 1 0 0

0 1 39 1 0 0 0 0 0 0 0 1 0 0

0 1 62 1 0 0 0 0 0 0 0 1 0 0

0 1 51 1 0 0 0 0 0 0 0 1 0 0

0 1 78 1 0 0 0 0 0 0 0 1 0 0

0 1 54 1 0 0 0 0 0 0 0 1 0 0

0 1 57 1 0 0 0 0 0 0 0 1 0 0

0 1 80 1 0 0 0 0 0 0 0 1 0 0

0 1 42 1 0 0 0 0 0 0 0 1 0 0

0 1 51 1 0 0 0 0 0 0 0 1 0 0

0 1 70 1 0 0 0 0 0 0 0 1 0 0

0 1 72 1 0 0 0 0 0 0 0 1 0 0

0 1 58 1 0 0 0 0 0 0 0 1 0 0

0 1 51 1 0 0 0 0 0 0 0 1 0 0

0 1 51 1 0 0 0 0 0 0 0 1 0 0

0 1 50 1 0 0 0 0 0 0 0 1 0 0

0 1 64 1 0 0 0 0 0 0 0 1 0 0

0 1 77 1 0 0 0 0 0 0 0 1 0 0

0 1 52 1 0 0 0 0 0 0 0 1 0 0

0 1 63 1 0 0 0 0 0 0 0 1 0 0

0 1 55 1 0 0 0 0 0 0 0 1 0 0

0 1 79 1 0 0 0 0 0 0 0 1 0 0

0 1 74 1 0 0 0 0 0 0 0 1 0 0

0 1 55 1 0 0 0 0 0 0 0 1 0 0

0 1 71 1 0 0 0 0 0 0 0 1 0 0

0 1 33 1 0 0 0 0 0 0 0 1 0 0

0 1 45 1 0 0 0 0 0 0 0 1 0 0

0 1 71 1 0 0 0 0 0 0 0 1 0 0

0 1 55 1 0 0 0 0 0 0 0 1 0 0

0 1 37 1 0 0 0 0 0 0 0 1 0 0

0 1 77 1 0 0 0 0 0 0 0 1 0 0

0 1 68 1 0 0 0 0 0 0 0 1 0 0

0 1 64 1 0 0 0 0 0 0 0 1 0 0

0 1 44 1 0 0 0 0 0 0 0 1 0 0

0 1 71 1 0 0 0 0 0 0 0 1 0 0

0 1 71 1 0 0 0 0 0 0 0 1 0 0

0 1 71 1 0 0 0 0 0 0 0 1 0 0

0 1 64 1 0 0 0 0 0 0 0 1 0 0

0 1 62 1 0 0 0 0 0 0 0 1 0 0

0 1 60 1 0 0 0 0 0 0 0 1 0 0

0 1 80 1 0 0 0 0 0 0 0 1 0 0

0 1 75 1 0 0 0 0 0 0 0 1 0 0

0 1 58 1 0 0 0 0 0 0 0 1 0 0

0 1 61 1 0 0 0 0 0 0 0 1 0 0

0 1 66 1 0 0 0 0 0 0 0 1 0 0

0 1 53 1 0 0 0 0 0 0 0 1 0 0

0 1 54 1 0 0 0 0 0 0 0 1 0 0

0 1 61 1 0 0 0 0 0 0 0 1 0 0

0 1 62 1 0 0 0 0 0 0 0 1 0 0

0 1 73 1 0 0 0 0 0 0 0 1 0 0

0 1 65 1 0 0 0 0 0 0 0 1 0 0

0 1 69 1 0 0 0 0 0 0 0 1 0 0

0 1 50 1 0 0 0 0 0 0 0 1 0 0

0 1 36 1 0 0 0 0 0 0 0 1 0 0

0 1 68 1 0 0 0 0 0 0 0 1 0 0

0 1 35 1 0 0 0 0 0 0 0 1 0 0

0 1 72 1 0 0 0 0 0 0 0 1 0 0

0 1 46 1 0 0 0 0 0 0 0 1 0 0

0 1 48 1 0 0 0 0 0 0 0 1 0 0

0 1 84 1 0 0 0 0 0 0 0 1 0 0

0 1 68 1 0 0 0 0 0 0 0 1 0 0

0 1 54 1 0 0 0 0 0 0 0 1 0 0

0 1 60 1 0 0 0 0 0 0 0 1 0 0

0 1 73 1 0 0 0 0 0 0 0 1 0 0

0 1 58 1 0 0 0 0 0 0 0 1 0 0

0 1 74 1 0 0 0 0 0 0 0 1 0 0

0 1 81 1 0 0 0 0 0 0 0 1 0 0

0 1 75 1 0 0 0 0 0 0 0 1 0 0

0 1 79 1 0 0 0 0 0 0 0 1 0 0

0 1 72 1 0 0 0 0 0 0 0 1 0 0

0 1 85 1 0 0 0 0 0 0 0 1 0 0

0 1 79 1 0 0 0 0 0 0 0 1 0 0

0 1 79 1 0 0 0 0 0 0 0 1 0 0

0 1 68 1 0 0 0 0 0 0 0 1 0 0

0 1 73 1 0 0 0 0 0 0 0 1 0 0

0 1 77 1 0 0 0 0 0 0 0 1 0 0

0 1 76 1 0 0 0 0 0 0 0 1 0 0

0 1 60 1 0 0 0 0 0 0 0 1 0 0

0 1 73 1 0 0 0 0 0 0 0 1 0 0

0 1 68 1 0 0 0 0 0 0 0 1 0 0

0 1 68 1 0 0 0 0 0 0 0 1 0 0

0 1 68 1 0 0 0 0 0 0 0 1 0 0

0 1 68 1 0 0 0 0 0 0 0 1 0 0

0 1 69 1 0 0 0 0 0 0 0 1 0 0

0 1 66 1 0 0 0 0 0 0 0 1 0 0

0 1 82 1 0 0 0 0 0 0 0 1 0 0

0 1 66 1 0 0 0 0 0 0 0 1 0 0

0 1 86 1 0 0 0 0 0 0 0 1 0 0

0 1 82 1 0 0 0 0 0 0 0 1 0 0

0 1 65 1 0 0 0 0 0 0 0 1 0 0

0 1 75 1 0 0 0 0 0 0 0 1 0 0

0 1 72 1 0 0 0 0 0 0 0 1 0 0

0 1 75 1 0 0 0 0 0 0 0 1 0 0

0 1 63 1 0 0 0 0 0 0 0 1 0 0

0 1 55 1 0 0 0 0 0 0 0 1 0 0

0 1 54 1 0 0 0 0 0 0 0 1 0 0

0 1 79 1 0 0 0 0 0 0 0 1 0 0

0 1 61 1 0 0 0 0 0 0 0 1 0 0

0 1 74 1 0 0 0 0 0 0 0 1 0 0

0 1 63 1 0 0 0 0 0 0 0 1 0 0

0 1 70 1 0 0 0 0 0 0 0 1 0 0

0 1 52 1 0 0 0 0 0 0 0 1 0 0

0 1 54 1 0 0 0 0 0 0 0 1 0 0

0 1 73 1 0 0 0 0 0 0 0 1 0 0

0 1 58 1 0 0 0 0 0 0 0 1 0 0

0 1 53 1 0 0 0 0 0 0 0 1 0 0

0 1 63 1 0 0 0 0 0 0 0 1 0 0

0 1 91 1 0 0 0 0 0 0 0 1 0 0

0 1 72 1 0 0 0 0 0 0 0 1 0 0

0 1 83 1 0 0 0 0 0 0 0 1 0 0

0 1 44 1 0 0 0 0 0 0 0 1 0 0

0 1 69 1 0 0 0 0 0 0 0 1 0 0

0 1 32 1 0 0 0 0 0 0 0 1 0 0

0 1 49 1 0 0 0 0 0 0 0 1 0 0

0 1 63 1 0 0 0 0 0 0 0 1 0 0

0 1 66 1 0 0 0 0 0 0 0 1 0 0

0 1 49 1 0 0 0 0 0 0 0 1 0 0

0 1 40 1 0 0 0 0 0 0 0 1 0 0

0 1 74 1 0 0 0 0 0 0 0 1 0 0

0 1 68 1 0 0 0 0 0 0 0 1 0 0

0 1 54 1 0 0 0 0 0 0 0 1 0 0

0 1 65 1 0 0 0 0 0 0 0 1 0 0

0 1 68 1 0 0 0 0 0 0 0 1 0 0

0 1 85 1 0 0 0 0 0 0 0 1 0 0

0 1 62 1 0 0 0 0 0 0 0 1 0 0

0 1 82 1 0 0 0 0 0 0 0 1 0 0

0 1 79 1 0 0 0 0 0 0 0 1 0 0

0 1 73 1 0 0 0 0 0 0 0 1 0 0

0 1 60 1 0 0 0 0 0 0 0 1 0 0

0 1 73 1 0 0 0 0 0 0 0 1 0 0

0 1 74 1 0 0 0 0 0 0 0 1 0 0

0 1 72 1 0 0 0 0 0 0 0 1 0 0

0 1 62 1 0 0 0 0 0 0 0 1 0 0

0 1 67 1 0 0 0 0 0 0 0 1 0 0

0 1 80 1 0 0 0 0 0 0 0 1 0 0

0 1 46 1 0 0 0 0 0 0 0 1 0 0

0 1 59 1 0 0 0 0 0 0 0 1 0 0

0 1 54 1 0 0 0 0 0 0 0 1 0 0

0 1 63 1 0 0 0 0 0 0 0 1 0 0

0 1 58 1 0 0 0 0 0 0 0 1 0 0

0 1 57 1 0 0 0 0 0 0 0 1 0 0

0 1 83 1 0 0 0 0 0 0 0 1 0 0

0 1 73 1 0 0 0 0 0 0 0 1 0 0

0 1 51 1 0 0 0 0 0 0 0 1 0 0

0 1 73 1 0 0 0 0 0 0 0 1 0 0

0 1 28 1 0 0 0 0 0 0 0 1 0 0

0 1 76 1 0 0 0 0 0 0 0 1 0 0

0 1 69 1 0 0 0 0 0 0 0 1 0 0

0 1 76 1 0 0 0 0 0 0 0 1 0 0

0 1 66 1 0 0 0 0 0 0 0 1 0 0

0 1 59 1 0 0 0 0 0 0 0 1 0 0

0 1 66 1 0 0 0 0 0 0 0 1 0 0

0 1 75 1 0 0 0 0 0 0 0 1 0 0

0 1 58 1 0 0 0 0 0 0 0 1 0 0

0 1 58 1 0 0 0 0 0 0 0 1 0 0

0 1 75 1 0 0 0 0 0 0 0 1 0 0

0 1 52 1 0 0 0 0 0 0 0 1 0 0

0 1 72 1 0 0 0 0 0 0 0 1 0 0

0 1 57 1 0 0 0 0 0 0 0 1 0 0

0 1 71 1 0 0 0 0 0 0 0 1 0 0

0 1 97 1 0 0 0 0 0 0 0 1 0 0

0 1 58 1 0 0 0 0 0 0 0 1 0 0

0 1 69 1 0 0 0 0 0 0 0 1 0 0

0 1 56 1 0 0 0 0 0 0 0 1 0 0

0 1 77 1 0 0 0 0 0 0 0 1 0 0

0 1 68 1 0 0 0 0 0 0 0 1 0 0

0 1 56 1 0 0 0 0 0 0 0 1 0 0

0 1 58 1 0 0 0 0 0 0 0 1 0 0

0 1 75 1 0 0 0 0 0 0 0 1 0 0

0 1 53 1 0 0 0 0 0 0 0 1 0 0

0 1 83 1 0 0 0 0 0 0 0 1 0 0

0 1 64 1 0 0 0 0 0 0 0 1 0 0

0 1 69 1 0 0 0 0 0 0 0 1 0 0

0 1 52 1 0 0 0 0 0 0 0 1 0 0

0 1 55 1 0 0 0 0 0 0 0 1 0 0

0 1 68 1 0 0 0 0 0 0 0 1 0 0

0 1 62 1 0 0 0 0 0 0 0 1 0 0

0 1 81 1 0 0 0 0 0 0 0 1 0 0

0 1 54 1 0 0 0 0 0 0 0 1 0 0

0 1 73 1 0 0 0 0 0 0 0 1 0 0

0 1 59 1 0 0 0 0 0 0 0 1 0 0

0 1 77 1 0 0 0 0 0 0 0 1 0 0

0 1 79 1 0 0 0 0 0 0 0 1 0 0

0 1 68 1 0 0 0 0 0 0 0 1 0 0

0 1 88 1 0 0 0 0 0 0 0 1 0 0

0 1 66 1 0 0 0 0 0 0 0 1 0 0

0 1 85 1 0 0 0 0 0 0 0 1 0 0

0 1 71 1 0 0 0 0 0 0 0 1 0 0

0 1 60 1 0 0 0 0 0 0 0 1 0 0

0 1 58 1 0 0 0 0 0 0 0 1 0 0

0 1 61 1 0 0 0 0 0 0 0 1 0 0

0 1 70 1 0 0 0 0 0 0 0 1 0 0

0 1 65 1 0 0 0 0 0 0 0 1 0 0

0 1 51 1 0 0 0 0 0 0 0 1 0 0

0 1 59 1 0 0 0 0 0 0 0 1 0 0

0 1 71 1 0 0 0 0 0 0 0 1 0 0

0 1 69 1 0 0 0 0 0 0 0 1 0 0

0 1 75 1 0 0 0 0 0 0 0 1 0 0

0 1 69 1 0 0 0 0 0 0 0 1 0 0

0 1 50 1 0 0 0 0 0 0 0 1 0 0

0 1 79 1 0 0 0 0 0 0 0 1 0 0

0 1 79 1 0 0 0 0 0 0 0 1 0 0

0 1 54 1 0 0 0 0 0 0 0 1 0 0

0 1 56 1 0 0 0 0 0 0 0 1 0 0

0 1 68 1 0 0 0 0 0 0 0 1 0 0

0 1 71 1 0 0 0 0 0 0 0 1 0 0

0 1 54 1 0 0 0 0 0 0 0 1 0 0

0 1 60 1 0 0 0 0 0 0 0 1 0 0

0 1 60 1 0 0 0 0 0 0 0 1 0 0

0 1 73 1 0 0 0 0 0 0 0 1 0 0

0 1 67 1 0 0 0 0 0 0 0 1 0 0

0 1 73 1 0 0 0 0 0 0 0 1 0 0

0 1 60 1 0 0 0 0 0 0 0 1 0 0

0 1 74 1 0 0 0 0 0 0 0 1 0 0

0 1 51 1 0 0 0 0 0 0 0 1 0 0

0 1 77 1 0 0 0 0 0 0 0 1 0 0

0 1 64 1 0 0 0 0 0 0 0 1 0 0

0 1 85 1 0 0 0 0 0 0 0 1 0 0

0 1 59 1 0 0 0 0 0 0 0 1 0 0

0 1 52 1 0 0 0 0 0 0 0 1 0 0

0 1 52 1 0 0 0 0 0 0 0 1 0 0

0 1 69 1 0 0 0 0 0 0 0 1 0 0

0 1 76 1 0 0 0 0 0 0 0 1 0 0

0 1 69 1 0 0 0 0 0 0 0 1 0 0

0 1 68 1 0 0 0 0 0 0 0 1 0 0

0 1 62 1 0 0 0 0 0 0 0 1 0 0

0 1 72 1 0 0 0 0 0 0 0 1 0 0

0 1 64 1 0 0 0 0 0 0 0 1 0 0

0 1 61 1 0 0 0 0 0 0 0 1 0 0

0 1 52 1 0 0 0 0 0 0 0 1 0 0

0 1 54 1 0 0 0 0 0 0 0 1 0 0

0 1 58 1 0 0 0 0 0 0 0 1 0 0

0 1 66 1 0 0 0 0 0 0 0 1 0 0

0 1 68 1 0 0 0 0 0 0 0 1 0 0

0 1 73 1 0 0 0 0 0 0 0 1 0 0

0 1 73 1 0 0 0 0 0 0 0 1 0 0

0 1 72 1 0 0 0 0 0 0 0 1 0 0

0 1 69 1 0 0 0 0 0 0 0 1 0 0

0 1 76 1 0 0 0 0 0 0 0 1 0 0

0 1 76 1 0 0 0 0 0 0 0 1 0 0

0 1 89 1 0 0 0 0 0 0 0 1 0 0

0 1 89 1 0 0 0 0 0 0 0 1 0 0

0 1 83 1 0 0 0 0 0 0 0 1 0 0

0 1 73 1 0 0 0 0 0 0 0 1 0 0

0 1 71 1 0 0 0 0 0 0 0 1 0 0

0 1 75 1 0 0 0 0 0 0 0 1 0 0

0 1 78 1 0 0 0 0 0 0 0 1 0 0

0 1 52 1 0 0 0 0 0 0 0 1 0 0

0 1 67 1 0 0 0 0 0 0 0 1 0 0

0 1 64 1 0 0 0 0 0 0 0 1 0 0

0 1 32 1 0 0 0 0 0 0 0 1 0 0

0 1 61 1 0 0 0 0 0 0 0 1 0 0

0 1 58 1 0 0 0 0 0 0 0 1 0 0

0 1 63 1 0 0 0 0 0 0 0 1 0 0

0 1 72 1 0 0 0 0 0 0 0 1 0 0

0 1 74 1 0 0 0 0 0 0 0 1 0 0

0 1 70 1 0 0 0 0 0 0 0 1 0 0

0 1 53 1 0 0 0 0 0 0 0 1 0 0

0 1 65 1 0 0 0 0 0 0 0 1 0 0

0 1 60 1 0 0 0 0 0 0 0 1 0 0

0 1 50 1 0 0 0 0 0 0 0 1 0 0

0 1 72 1 0 0 0 0 0 0 0 1 0 0

0 1 70 1 0 0 0 0 0 0 0 1 0 0

0 1 74 1 0 0 0 0 0 0 0 1 0 0

0 1 46 1 0 0 0 0 0 0 0 1 0 0

0 1 55 1 0 0 0 0 0 0 0 1 0 0

0 1 71 1 0 0 0 0 0 0 0 1 0 0

0 1 71 1 0 0 0 0 0 0 0 1 0 0

0 1 59 1 0 0 0 0 0 0 0 1 0 0

0 1 61 1 0 0 0 0 0 0 0 1 0 0

0 1 58 1 0 0 0 0 0 0 0 1 0 0

0 1 60 1 0 0 0 0 0 0 0 1 0 0

0 1 58 1 0 0 0 0 0 0 0 1 0 0

0 1 48 1 0 0 0 0 0 0 0 1 0 0

0 1 46 1 0 0 0 0 0 0 0 1 0 0

0 1 69 1 0 0 0 0 0 0 0 1 0 0

0 1 68 1 0 0 0 0 0 0 0 1 0 0

0 1 68 1 0 0 0 0 0 0 0 1 0 0

0 1 59 1 0 0 0 0 0 0 0 1 0 0

0 1 49 1 0 0 0 0 0 0 0 1 0 0

0 1 43 1 0 0 0 0 0 0 0 1 0 0

0 1 67 1 0 0 0 0 0 0 0 1 0 0

0 1 56 1 0 0 0 0 0 0 0 1 0 0

0 1 48 1 0 0 0 0 0 0 0 1 0 0

0 1 62 1 0 0 0 0 0 0 0 1 0 0

0 1 52 1 0 0 0 0 0 0 0 1 0 0

0 1 89 1 0 0 0 0 0 0 0 1 0 0

0 1 67 1 0 0 0 0 0 0 0 1 0 0

0 1 66 1 0 0 0 0 0 0 0 1 0 0

0 1 61 1 0 0 0 0 0 0 0 1 0 0

0 1 56 1 0 0 0 0 0 0 0 1 0 0

0 1 85 1 0 0 0 0 0 0 0 1 0 0

0 1 51 1 0 0 0 0 0 0 0 1 0 0

0 1 78 1 0 0 0 0 0 0 0 1 0 0

0 1 59 1 0 0 0 0 0 0 0 1 0 0

0 1 57 1 0 0 0 0 0 0 0 1 0 0

0 1 48 1 0 0 0 0 0 0 0 1 0 0

0 1 67 1 0 0 0 0 0 0 0 1 0 0

0 1 61 1 0 0 0 0 0 0 0 1 0 0

0 1 59 1 0 0 0 0 0 0 0 1 0 0

0 1 50 1 0 0 0 0 0 0 0 1 0 0

0 1 46 1 0 0 0 0 0 0 0 1 0 0

0 1 56 1 0 0 0 0 0 0 0 1 0 0

0 1 59 1 0 0 0 0 0 0 0 1 0 0

0 1 72 1 0 0 0 0 0 0 0 1 0 0

0 1 79 1 0 0 0 0 0 0 0 1 0 0

0 1 65 1 0 0 0 0 0 0 0 1 0 0

0 1 64 1 0 0 0 0 0 0 0 1 0 0

0 1 75 1 0 0 0 0 0 0 0 1 0 0

0 1 84 1 0 0 0 0 0 0 0 1 0 0

0 1 52 1 0 0 0 0 0 0 0 1 0 0

0 1 68 1 0 0 0 0 0 0 0 1 0 0

0 1 84 1 0 0 0 0 0 0 0 1 0 0

0 1 88 1 0 0 0 0 0 0 0 1 0 0

0 1 67 1 0 0 0 0 0 0 0 1 0 0

0 1 54 1 0 0 0 0 0 0 0 1 0 0

0 1 62 1 0 0 0 0 0 0 0 1 0 0

0 1 67 1 0 0 0 0 0 0 0 1 0 0

0 1 66 1 0 0 0 0 0 0 0 1 0 0

0 1 55 1 0 0 0 0 0 0 0 1 0 0

0 1 70 1 0 0 0 0 0 0 0 1 0 0

0 1 55 1 0 0 0 0 0 0 0 1 0 0

0 1 63 1 0 0 0 0 0 0 0 1 0 0

0 1 71 1 0 0 0 0 0 0 0 1 0 0

0 1 70 1 0 0 0 0 0 0 0 1 0 0

0 1 64 1 0 0 0 0 0 0 0 1 0 0

0 1 75 1 0 0 0 0 0 0 0 1 0 0

0 1 78 1 0 0 0 0 0 0 0 1 0 0

0 1 64 1 0 0 0 0 0 0 0 1 0 0

0 1 61 1 0 0 0 0 0 0 0 1 0 0

0 1 72 1 0 0 1 0 0 0 0 1 0 0

0 1 59 1 0 0 0 0 0 0 0 1 0 0

0 1 67 1 0 0 0 0 0 0 0 1 0 0

0 1 57 1 0 0 0 0 0 0 0 1 0 0

0 1 89 1 0 0 0 0 0 0 0 1 0 0

0 1 65 1 0 0 0 0 0 0 0 1 0 0

0 1 71 1 0 0 0 0 0 0 0 1 0 0

0 1 58 1 0 0 0 0 0 0 0 1 0 0

0 1 64 1 0 0 0 0 0 0 0 1 0 0

0 1 57 1 0 0 0 0 0 0 0 1 0 0

0 1 73 1 0 0 0 0 0 0 0 1 0 0

0 1 49 1 0 0 0 0 0 0 0 1 0 0

0 1 66 1 0 0 0 0 0 0 0 1 0 0

0 1 63 1 0 0 0 0 0 0 0 1 0 0

0 1 51 1 0 0 0 0 0 0 0 1 0 0

0 1 75 1 0 0 0 0 0 0 0 1 0 0

0 1 62 1 0 0 0 0 0 0 0 1 0 0

0 1 78 1 0 0 0 0 0 0 0 1 0 0

0 1 57 1 0 0 0 0 0 0 0 1 0 0

0 1 52 1 0 0 0 0 0 0 0 1 0 0

0 1 77 1 0 0 0 0 0 0 0 1 0 0

0 1 60 1 0 0 0 0 0 0 0 1 0 0

0 1 60 1 0 0 0 0 0 0 0 1 0 0

0 1 72 1 0 0 0 0 0 0 0 1 0 0

0 1 74 1 0 0 0 0 0 0 0 1 0 0

0 1 59 1 0 0 0 0 0 0 0 1 0 0

0 1 69 1 0 0 0 0 0 0 0 1 0 0

0 1 57 1 0 0 0 0 0 0 0 1 0 0

0 1 73 1 0 0 0 0 0 0 0 1 0 0

0 1 52 1 0 0 0 0 0 0 0 1 0 0

0 1 59 1 0 0 0 0 0 0 0 1 0 0

0 1 47 1 0 0 0 0 0 0 0 1 0 0

0 1 59 1 0 0 0 0 0 0 0 1 0 0

0 1 46 1 0 0 0 0 0 0 0 1 0 0

0 1 63 1 0 0 0 0 0 0 0 1 0 0

0 1 72 1 0 0 0 0 0 0 0 1 0 0

0 1 86 1 0 0 0 0 0 0 0 1 0 0

0 1 61 1 0 0 0 0 0 0 0 1 0 0

0 1 89 1 0 0 0 0 0 0 0 1 0 0

0 1 63 1 0 0 0 0 0 0 0 1 0 0

0 1 77 1 0 0 0 0 0 0 0 1 0 0

0 1 71 1 0 0 0 0 0 0 0 1 0 0

0 1 76 1 0 0 0 0 0 0 0 1 0 0

0 1 66 1 0 0 0 0 0 0 0 1 0 0

0 1 46 1 0 0 0 0 0 0 0 1 0 0

0 1 65 1 0 0 0 0 0 0 0 1 0 0

0 1 60 1 0 0 0 0 0 0 0 1 0 0

0 1 59 1 0 0 0 0 0 0 0 1 0 0

0 1 74 1 0 0 0 0 0 0 0 1 0 0

0 1 73 1 0 0 0 0 0 0 0 1 0 0

0 1 74 1 0 0 0 0 0 0 0 1 0 0

0 1 81 1 0 0 0 0 0 0 0 1 0 0

0 1 69 1 0 0 0 0 0 0 0 1 0 0

0 1 66 1 0 0 0 0 0 0 0 1 0 0

0 1 74 1 0 0 0 0 0 0 0 1 0 0

0 1 74 1 0 0 0 0 0 0 0 1 0 0

0 1 80 1 0 0 0 0 0 0 0 1 0 0

0 1 72 1 0 0 0 0 0 0 0 1 0 0

0 1 47 1 0 0 0 0 0 0 0 1 0 0

0 1 71 1 0 0 0 0 0 0 0 1 0 0

0 1 66 1 0 0 0 0 0 0 0 1 0 0

0 1 75 1 0 0 0 0 0 0 0 1 0 0

0 1 74 1 0 0 0 0 0 0 0 1 0 0

0 1 65 1 0 0 0 0 0 0 0 1 0 0

0 1 67 1 0 0 0 0 0 0 0 1 0 0

0 1 57 1 0 0 0 0 0 0 0 1 0 0

0 1 74 1 0 0 0 0 0 0 0 1 0 0

0 1 79 1 0 0 0 0 0 0 0 1 0 0

0 1 75 1 0 0 0 0 0 0 0 1 0 0

0 1 76 1 0 0 0 0 0 0 0 1 0 0

0 1 63 1 0 0 0 0 0 0 0 1 0 0

0 1 75 1 0 0 0 0 0 0 0 1 0 0

0 1 75 1 0 0 0 0 0 0 0 1 0 0

0 1 73 1 0 0 0 0 0 0 0 1 0 0

0 1 81 1 0 0 0 0 0 0 0 1 0 0

0 1 76 1 0 0 0 0 0 0 0 1 0 0

0 1 77 1 0 0 0 0 0 0 0 1 0 0

0 1 56 1 0 0 0 0 0 0 0 1 0 0

0 1 74 1 0 0 0 0 0 0 0 1 0 0

0 1 76 1 0 0 0 0 0 0 0 1 0 0

0 1 76 1 0 0 0 0 0 0 0 1 0 0

0 1 67 1 0 0 0 0 0 0 0 1 0 0

0 1 69 1 0 0 0 0 0 0 0 1 0 0

0 1 64 1 0 0 0 0 0 0 0 1 0 0

0 1 59 1 0 0 0 0 0 0 0 1 0 0

0 1 63 1 0 0 0 0 0 0 0 1 0 0

0 1 72 1 0 0 0 0 0 0 0 1 0 0

0 1 57 1 0 0 0 0 0 0 0 1 0 0

0 1 74 1 0 0 0 0 0 0 0 1 0 0

0 1 81 1 0 0 0 0 0 0 0 1 0 0

0 1 73 1 0 0 0 0 0 0 0 1 0 0

0 1 63 1 0 0 0 0 0 0 0 1 0 0

0 1 71 1 0 0 0 0 0 0 0 1 0 0

0 1 80 1 0 0 0 0 0 0 0 1 0 0

0 1 77 1 0 0 0 0 0 0 0 1 0 0

0 1 79 1 0 0 0 0 0 0 0 1 0 0

0 1 71 1 0 0 0 0 0 0 0 1 0 0

0 1 68 1 0 0 0 0 0 0 0 1 0 0

0 1 65 1 0 0 0 0 0 0 0 1 0 0

0 1 78 1 0 0 0 0 0 0 0 1 0 0

0 1 50 1 0 0 0 0 0 0 0 1 0 0

0 1 59 1 0 0 0 0 0 0 0 1 0 0

0 1 45 1 0 0 0 0 0 0 0 1 0 0

0 1 64 1 0 0 0 0 0 0 0 1 0 0

0 1 58 1 0 0 0 0 0 0 0 1 0 0

0 1 63 1 0 0 0 0 0 0 0 1 0 0

0 1 78 1 0 0 0 0 0 0 0 1 0 0

0 1 78 1 0 0 0 0 0 0 0 1 0 0

0 1 81 1 0 0 0 0 0 0 0 1 0 0

0 1 83 1 0 0 0 0 0 0 0 1 0 0

0 1 78 1 0 0 0 0 0 0 0 1 0 0

0 1 73 1 0 0 0 0 0 0 0 1 0 0

0 1 59 1 0 0 0 0 0 0 0 1 2 0

0 1 65 1 0 0 0 0 0 0 0 1 1 0

0 1 58 1 0 0 0 0 0 0 0 1 1 0

0 1 65 1 0 0 0 0 0 0 0 1 2 0

0 1 29 1 0 0 0 0 0 0 0 1 2 0

0 1 70 1 0 0 0 0 0 0 0 1 2 0

0 1 59 1 0 0 0 0 0 0 0 1 2 0

0 1 47 1 0 0 0 0 0 0 0 1 3 0

0 1 81 1 0 0 0 0 0 0 0 1 3 0

0 1 63 1 0 0 0 0 0 0 0 1 4 0

0 1 58 1 0 0 0 0 0 0 0 1 4 0

0 1 78 1 0 0 0 0 0 0 0 1 0 0

0 1 46 1 0 0 0 0 0 0 0 1 2 0

0 1 61 1 0 0 0 0 0 0 0 1 0 0

0 1 79 1 0 0 0 0 0 0 0 1 0 0

0 1 61 1 0 0 0 0 0 0 0 1 0 0

0 1 64 1 0 0 0 0 0 0 0 1 0 0

0 1 74 1 0 0 0 0 0 0 0 1 0 0

0 1 63 1 0 0 0 0 0 0 0 1 0 0

0 1 63 1 0 0 0 0 0 0 0 1 0 0

0 1 68 1 0 0 0 0 0 0 0 1 0 0

0 1 66 1 0 0 0 0 0 0 0 1 0 0

0 1 69 1 0 0 0 0 0 0 0 1 0 0

0 1 62 1 0 0 0 0 0 0 0 1 0 0

0 1 55 1 0 0 0 0 0 0 0 1 0 0

0 1 84 1 0 0 0 0 0 0 0 1 0 0

0 1 76 1 0 0 0 0 0 0 0 1 0 0

0 1 71 1 0 0 0 0 0 0 0 1 0 0

0 1 81 1 0 0 0 0 0 0 0 1 0 0

0 1 50 1 0 0 0 0 0 0 0 1 0 0

0 1 38 1 0 0 0 0 0 0 0 1 0 0

0 1 77 1 0 0 0 0 0 0 0 1 0 0

0 1 64 1 0 0 0 0 0 0 0 1 0 0

0 1 73 1 0 0 0 0 0 0 0 1 0 0

0 1 78 1 0 0 0 0 0 0 0 1 0 0

0 1 71 1 0 0 0 0 0 0 0 1 0 0

0 1 51 1 0 0 0 0 0 0 0 1 0 0

0 1 73 1 0 0 0 0 0 0 0 1 0 0

0 1 85 1 0 0 0 0 0 0 0 1 0 0

0 1 56 1 0 0 0 0 0 0 0 1 0 0

0 1 77 1 0 0 0 0 0 0 0 1 0 0

0 1 39 1 0 0 0 0 0 0 0 1 0 0

0 1 57 1 0 0 0 0 0 0 0 1 0 0

0 1 57 1 0 0 0 0 0 0 0 1 0 0

0 1 71 1 0 0 0 0 0 0 0 1 0 0

0 1 70 1 0 0 0 0 0 0 0 1 0 0

0 1 78 1 0 0 0 0 0 0 0 1 0 0

0 1 57 1 0 0 0 0 0 0 0 1 0 0

0 1 78 1 0 0 0 0 0 0 0 1 0 0

0 1 81 1 0 0 0 0 0 0 0 1 0 0

0 1 61 1 0 0 0 0 0 0 0 1 0 0

0 1 61 1 0 0 0 0 0 0 0 1 0 0

0 1 51 1 0 0 0 0 0 0 0 1 0 0

0 1 64 1 0 0 0 0 0 0 0 1 0 0

0 1 59 1 0 0 0 0 0 0 0 1 0 0

0 1 59 1 0 0 0 0 0 0 0 1 0 0

0 1 75 1 0 0 0 0 0 0 0 1 0 0

0 1 55 1 0 0 0 0 0 0 0 1 0 0

0 1 71 1 0 0 0 0 0 0 0 1 0 0

0 1 71 1 0 0 0 0 0 0 0 1 0 0

0 1 71 1 0 0 0 0 0 0 0 1 0 0

0 1 86 1 0 0 0 0 0 0 0 1 0 0

0 1 63 1 0 0 0 0 0 0 0 1 0 0

0 1 68 1 0 0 0 0 0 0 0 1 0 0

0 1 70 1 0 0 0 0 0 0 0 1 0 0

0 1 49 1 0 0 0 0 0 0 0 1 0 0

0 1 64 1 0 0 0 0 0 0 0 1 0 0

0 1 39 1 0 0 0 0 0 0 0 1 0 0

0 1 59 1 0 0 0 0 0 0 0 1 0 0

0 1 61 1 0 0 0 0 0 0 0 1 0 0

0 1 61 1 0 0 0 0 0 0 0 1 0 0

0 1 22 1 0 0 0 0 0 0 0 1 0 0

0 1 69 1 0 0 0 0 0 0 0 1 0 0

0 1 36 1 0 0 0 0 0 0 0 1 0 0

0 1 47 1 0 0 0 0 0 0 0 1 0 0

0 1 69 1 0 0 0 0 0 0 0 1 0 0

0 1 72 1 0 0 0 0 0 0 0 1 0 0

0 1 64 1 0 0 0 0 0 0 0 1 0 0

0 1 56 1 0 0 0 0 0 0 0 1 0 0

0 1 49 1 0 0 0 0 0 0 0 1 0 0

0 1 37 1 0 0 0 0 0 0 0 1 0 0

0 1 58 1 0 0 0 0 0 0 0 1 0 0

0 1 40 1 0 0 0 0 0 0 0 1 0 0

0 1 38 1 0 0 0 0 0 0 0 1 0 0

0 1 38 1 0 0 0 0 0 0 0 1 0 0

0 1 62 1 0 0 0 0 0 0 0 1 0 0

0 1 53 1 0 0 0 0 0 0 0 1 0 0

0 1 80 1 0 0 0 0 0 0 0 1 0 0

0 1 56 1 0 0 0 0 0 0 0 1 0 0

0 1 39 1 0 0 0 0 0 0 0 1 0 0

0 1 70 1 0 0 0 0 0 0 0 1 0 0

0 1 75 1 0 0 0 0 0 0 0 1 0 0

0 1 49 1 0 0 0 0 0 0 0 1 0 0

0 1 38 1 0 0 0 0 0 0 0 1 0 0

0 1 59 1 0 0 0 0 0 0 0 1 0 0

0 1 48 1 0 0 0 0 0 0 0 1 0 0

0 1 43 1 0 0 0 0 0 0 0 1 0 0

0 1 42 1 0 0 0 0 0 0 0 1 0 0

0 1 65 1 0 0 0 0 0 0 0 1 0 0

0 1 67 1 0 0 0 0 0 0 0 1 0 0

0 1 20 1 0 0 0 0 0 0 0 1 0 0

0 1 37 1 0 0 0 0 0 0 0 1 0 0

0 1 65 1 0 0 0 0 0 0 0 1 0 0

0 1 72 1 0 0 0 0 0 0 0 1 0 0

0 1 73 1 0 0 0 0 0 0 0 1 0 0

0 1 69 1 0 0 0 0 0 0 0 1 0 0

0 1 67 1 0 0 0 0 0 0 0 1 0 0

0 1 49 1 0 0 0 0 0 0 0 1 0 0

0 1 45 1 0 0 0 0 0 0 0 1 0 0

0 1 75 1 0 0 0 0 0 0 0 1 0 0

0 1 78 1 0 0 0 0 0 0 0 1 0 0

0 1 43 1 0 0 0 0 0 0 0 1 0 0

0 1 84 1 0 0 0 0 0 0 0 1 0 0

0 1 67 1 0 0 0 0 0 0 0 1 0 0

0 1 71 1 0 0 0 0 0 0 0 1 0 0

0 1 79 1 0 0 0 0 0 0 0 1 0 0

0 1 64 1 0 0 0 0 0 0 0 1 0 0

0 1 54 1 0 0 0 0 0 0 0 1 0 0

0 1 52 1 0 0 0 0 0 0 0 1 0 0

0 1 52 1 0 0 0 0 0 0 0 1 0 0

0 1 59 1 0 0 0 0 0 0 0 1 0 0

0 1 54 1 0 0 0 0 0 0 0 1 0 0

0 1 62 1 0 0 0 0 0 0 0 1 0 0

0 1 44 1 0 0 0 0 0 0 0 1 0 0

0 1 56 1 0 0 0 0 0 0 0 1 0 0

0 1 69 1 0 0 0 0 0 0 0 1 0 0

0 1 44 1 0 0 0 0 0 0 0 1 0 0

0 1 40 1 0 0 0 0 0 0 0 1 0 0

0 1 65 1 0 0 0 0 0 0 0 1 0 0

0 1 86 1 0 0 0 0 0 0 0 1 0 0

0 1 84 1 0 0 0 0 0 0 0 1 0 0

0 1 74 1 0 0 0 0 0 0 0 1 0 0

0 1 59 1 0 0 0 0 0 0 0 1 0 0

0 1 83 1 0 0 0 0 0 0 0 1 0 0

0 1 74 1 0 0 0 0 0 0 0 1 0 0

0 1 78 1 0 0 0 0 0 0 0 1 0 0

0 1 61 1 0 0 0 0 0 0 0 1 0 0

0 1 63 1 0 0 0 0 0 0 0 1 0 0

0 1 47 1 0 0 0 0 0 0 0 1 0 0

0 1 79 1 0 0 0 0 0 0 0 1 0 0

0 1 74 1 0 0 0 0 0 0 0 1 0 0

0 1 50 1 0 0 0 0 0 0 0 1 0 0

0 1 61 1 0 0 0 0 0 0 0 1 0 0

0 1 71 1 0 0 0 0 0 0 0 1 0 0

0 1 65 1 0 0 0 0 0 0 0 1 0 0

0 1 47 1 0 0 0 0 0 0 0 1 0 0

0 1 83 1 0 0 0 0 0 0 0 1 0 0

0 1 69 1 0 0 0 0 0 0 0 1 0 0

0 1 73 1 0 0 0 0 0 0 0 1 0 0

0 1 58 1 0 0 0 0 0 0 0 1 0 0

0 1 62 1 0 0 0 0 0 0 0 1 0 0

0 1 65 1 0 0 0 0 0 0 0 1 0 0

0 1 42 1 0 0 0 0 0 0 0 1 0 0

0 1 65 1 0 0 0 0 0 0 0 1 0 0

0 1 29 1 0 0 0 0 0 0 0 1 0 0

0 1 78 1 0 0 0 0 0 0 0 1 0 0

0 1 82 1 0 0 0 0 0 0 0 1 0 0

0 1 87 1 0 0 0 0 0 0 0 1 0 0

0 1 62 1 0 0 0 0 0 0 0 1 0 0

0 1 65 1 0 0 0 0 0 0 0 1 0 0

0 1 70 1 0 0 0 0 0 0 0 1 0 0

0 1 72 1 0 0 0 0 0 0 0 1 0 0

0 1 62 1 0 0 0 0 0 0 0 1 0 0

0 1 58 1 0 0 0 0 0 0 0 1 0 0

0 1 85 1 0 0 0 0 0 0 0 1 0 0

0 1 82 1 0 0 0 0 0 0 0 1 0 0

0 1 73 1 0 0 0 0 0 0 0 1 0 0

0 1 49 1 0 0 0 0 0 0 0 1 0 0

0 1 30 1 0 0 0 0 0 0 0 1 0 0

0 1 59 1 0 0 0 0 0 0 0 1 0 0

0 1 76 1 0 0 0 0 0 0 0 1 0 0

0 1 77 1 0 0 0 0 0 0 0 1 0 0

0 1 52 1 0 0 0 0 0 0 0 1 0 0

0 1 47 1 0 0 0 0 0 0 0 1 0 0

0 1 49 1 0 0 0 0 0 0 0 1 0 0

0 1 66 1 0 0 1 0 0 0 0 1 0 0

0 1 57 1 0 0 0 0 0 0 0 1 0 0

0 1 83 1 0 0 0 0 0 0 0 1 0 0

0 1 85 1 0 0 0 0 0 0 0 1 0 0

0 1 71 1 0 0 0 0 0 0 0 1 0 0

0 1 70 1 0 0 0 0 0 0 0 1 0 0

0 1 64 1 0 0 0 0 0 0 0 1 0 0

0 1 63 1 0 0 0 0 0 0 0 1 0 0

0 1 78 1 0 0 0 0 0 0 0 1 0 0

0 1 62 1 0 0 0 0 0 0 0 1 0 0

0 1 62 1 0 0 0 0 0 0 0 1 0 0

0 1 80 1 0 0 0 0 0 0 0 1 0 0

0 1 65 1 0 0 0 0 0 0 0 1 0 0

0 1 78 1 0 0 0 0 0 0 0 1 0 0

0 1 70 1 0 0 0 0 0 0 0 1 0 0

0 1 47 1 0 0 0 0 0 0 0 1 0 0

0 1 70 1 0 0 0 0 0 0 0 1 0 0

0 1 51 1 0 0 0 0 0 0 0 1 0 0

0 1 62 1 0 0 0 0 0 0 0 1 0 0

0 1 74 1 0 0 0 0 0 0 0 1 0 0

0 1 79 1 0 0 0 0 0 0 0 1 0 0

0 1 66 1 0 0 0 0 0 0 0 1 0 0

0 1 61 1 0 0 0 0 0 0 0 1 0 0

0 1 77 1 0 0 0 0 0 0 0 1 0 0

0 1 54 1 0 0 0 0 0 0 0 1 0 0

0 1 64 1 0 0 0 0 0 0 0 1 0 0

0 1 45 1 0 0 0 0 0 0 0 1 0 0

0 1 74 1 0 0 0 0 0 0 0 1 0 0

0 1 65 1 0 0 0 0 0 0 0 1 0 0

0 1 64 1 0 0 0 0 0 0 0 1 0 0

0 1 83 1 0 0 0 0 0 0 0 1 0 0

0 1 64 1 0 0 0 0 0 0 0 1 0 0

0 1 54 1 0 0 0 0 0 0 0 1 0 0

0 1 59 1 0 0 0 0 0 0 0 1 0 0

0 1 71 1 0 0 0 0 0 0 0 1 0 0

0 1 88 1 0 0 0 0 0 0 0 1 0 0

0 1 48 1 0 0 0 0 0 0 0 1 0 0

0 1 14 1 0 0 0 0 0 0 0 1 0 0

0 1 61 1 0 0 0 0 0 0 0 1 0 0

0 1 61 1 0 0 0 0 0 0 0 1 0 0

0 1 80 1 0 0 0 0 0 0 0 1 0 0

0 1 35 1 0 0 0 0 0 0 0 1 0 0

0 1 64 1 0 0 0 0 0 0 0 1 0 0

0 1 52 1 0 0 0 0 0 0 0 1 0 0

0 1 54 1 0 0 0 0 0 0 0 1 0 0

0 1 52 1 0 0 0 0 0 0 0 1 0 0

0 1 70 1 0 0 0 0 0 0 0 1 0 0

0 1 62 1 0 0 0 0 0 0 0 1 0 0

0 1 60 1 0 0 0 0 0 0 0 1 0 0

0 1 57 1 0 0 0 0 0 0 0 1 0 0

0 1 65 1 0 0 0 0 0 0 0 1 0 0

0 1 58 1 0 0 0 0 0 0 0 1 0 0

0 1 83 1 0 0 0 0 0 0 0 1 0 0

0 1 71 1 0 0 0 0 0 0 0 1 0 0

0 1 68 1 0 0 0 0 0 0 0 1 0 0

0 1 78 1 0 0 1 0 0 0 0 1 0 0

0 1 53 1 0 0 0 0 0 0 0 1 0 0

0 1 84 1 0 0 0 0 0 0 0 1 0 0

0 1 45 1 0 0 0 0 0 0 0 1 0 0

0 1 55 1 0 0 0 0 0 0 0 1 0 0

0 1 50 1 0 0 0 0 0 0 0 1 0 0

0 1 65 1 0 0 0 0 0 0 0 1 0 0

0 1 62 1 0 0 0 0 0 0 0 1 0 0

0 1 57 1 0 0 0 0 0 0 0 1 0 0

0 1 73 1 0 0 0 0 0 0 0 1 0 0

0 1 73 1 0 0 0 0 0 0 0 1 0 0

0 1 57 1 0 0 0 0 0 0 0 1 0 0

0 1 43 1 0 0 0 0 0 0 0 1 0 0

0 1 61 1 0 0 0 0 0 0 0 1 0 0

0 1 82 1 0 0 0 0 0 0 0 1 0 0

0 1 57 1 0 0 0 0 0 0 0 1 0 0

0 1 80 1 0 0 0 0 0 0 0 1 0 0

0 1 74 1 0 0 0 0 0 0 0 1 0 0

0 1 71 1 0 0 0 0 0 0 0 1 0 0

0 1 71 1 0 0 0 0 0 0 0 1 0 0

0 1 69 1 0 0 0 0 0 0 0 1 0 0

0 1 54 1 0 0 0 0 0 0 0 1 0 0

0 1 56 1 0 0 0 0 0 0 0 1 0 0

0 1 74 1 0 0 0 0 0 0 0 1 0 0

0 1 64 1 0 0 0 0 0 0 0 1 0 0

0 1 64 1 0 0 0 0 0 0 0 1 0 0

0 1 79 1 0 0 0 0 0 0 0 1 0 0

0 1 51 1 0 0 0 0 0 0 0 1 0 0

0 1 77 1 0 0 0 0 0 0 0 1 0 0

0 1 51 1 0 0 0 0 0 0 0 1 0 0

0 1 51 1 0 0 0 0 0 0 0 1 0 0

0 1 64 1 0 0 0 0 0 0 0 1 0 0

0 1 53 1 0 0 0 0 0 0 0 1 0 0

0 1 62 1 0 0 0 0 0 0 0 1 0 0

0 1 70 1 0 0 0 0 0 0 0 1 0 0

0 1 79 1 0 0 0 0 0 0 0 1 0 0

0 1 66 1 0 0 0 0 0 0 0 1 0 0

0 1 61 1 0 0 0 0 0 0 0 1 0 0

0 1 45 1 0 0 0 0 0 0 0 1 0 0

0 1 71 1 0 0 0 0 0 0 0 1 0 0

0 1 60 1 0 0 0 0 0 0 0 1 0 0

0 1 65 1 0 0 0 0 0 0 0 1 0 0

0 1 67 1 0 0 0 0 0 0 0 1 0 0

0 1 81 1 0 0 0 0 0 0 0 1 0 0

0 1 67 1 0 0 0 0 0 0 0 1 0 0

0 1 64 1 0 0 0 0 0 0 0 1 0 0

0 1 71 1 0 0 0 0 0 0 0 1 0 0

0 1 53 1 0 0 0 0 0 0 0 1 0 0

0 1 69 1 0 0 0 0 0 0 0 1 0 0

0 1 55 1 0 0 0 0 0 0 0 1 0 0

0 1 74 1 0 0 0 0 0 0 0 1 0 0

0 1 76 1 0 0 0 0 0 0 0 1 1 0

0 1 86 1 0 0 0 0 0 0 0 1 1 0

0 1 66 1 0 0 0 0 0 0 0 1 1 0

0 1 64 1 0 0 0 0 0 0 0 1 1 0

0 1 81 1 0 0 0 0 0 0 0 1 2 0

0 1 63 1 0 0 0 0 0 0 0 1 2 0

0 1 59 1 0 0 0 0 0 0 0 1 2 0

0 1 72 1 0 0 0 0 0 0 0 1 2 0

0 1 50 1 0 0 0 0 0 0 0 1 2 0

0 1 79 1 0 0 0 0 0 0 0 1 2 0

0 1 59 1 0 0 0 0 0 0 0 1 2 0

0 1 75 1 0 0 0 0 0 0 0 1 2 0

0 2 70 1 0 0 0 0 0 0 0 1 3 0

0 2 70 1 0 0 0 0 0 0 0 1 3 0

0 2 68 1 0 0 0 0 0 0 0 1 3 0

0 2 75 1 0 0 0 0 0 0 0 1 3 0

0 2 73 1 0 0 0 0 0 0 0 1 4 0

0 2 73 1 0 0 0 0 0 0 0 1 4 0

0 2 73 1 0 0 0 0 0 0 0 1 4 0

0 2 81 1 0 0 0 0 0 0 0 1 0 0

0 2 70 1 0 0 0 0 0 0 0 1 0 0

0 2 70 1 0 0 0 0 0 0 0 1 0 0

0 2 72 1 0 0 0 0 0 0 0 1 0 0

0 2 58 1 0 0 0 0 0 0 0 1 0 0

0 2 58 1 0 0 0 0 0 0 0 1 0 0

0 2 75 1 0 0 0 0 0 0 0 1 0 0

0 2 61 1 0 0 0 0 0 0 0 1 0 0

0 2 75 1 0 0 0 0 0 0 0 1 0 0

0 2 68 1 0 0 0 0 0 0 0 1 0 0

0 2 66 1 0 0 0 0 0 0 0 1 0 0

0 2 73 1 0 0 0 0 0 0 0 1 0 0

0 2 57 1 0 0 0 0 0 0 0 1 0 0

0 2 58 1 0 0 0 0 0 0 0 1 0 0

0 2 86 1 0 0 0 0 0 0 0 1 0 0

0 2 74 1 0 0 0 0 0 0 0 1 0 0

0 2 75 1 0 0 0 0 0 0 0 1 0 0

0 2 78 1 0 0 0 0 0 0 0 1 0 0

0 2 80 1 0 0 0 0 0 0 0 1 0 0

0 2 63 1 0 0 0 0 0 0 0 1 0 0

0 2 60 1 0 0 0 0 0 0 0 1 0 0

0 2 78 1 0 0 0 0 0 0 0 1 0 0

0 2 45 1 0 0 0 0 0 0 0 1 0 0

0 2 52 1 0 0 0 0 0 0 0 1 0 0

0 2 62 1 0 0 0 0 0 0 0 1 0 0

0 2 66 1 0 0 0 0 0 0 0 1 0 0

0 2 80 1 0 0 0 0 0 0 0 1 0 0

0 2 75 1 0 0 0 0 0 0 0 1 0 0

0 2 60 1 0 0 0 0 0 0 0 1 0 0

0 2 64 1 0 0 0 0 0 0 0 1 0 0

0 2 54 1 0 0 0 0 0 0 0 1 0 0

0 2 74 1 0 0 0 0 0 0 0 1 0 0

0 2 63 1 0 0 0 0 0 0 0 1 0 0

0 2 54 1 0 0 0 0 0 0 0 1 0 0

0 2 88 1 0 0 0 0 0 0 0 1 0 0

0 2 75 1 0 0 0 0 0 0 0 1 0 0

0 2 66 1 0 0 0 0 0 0 0 1 0 0

0 2 62 1 0 0 0 0 0 0 0 1 0 0

0 2 75 1 0 0 0 0 0 0 0 1 0 0

0 2 71 1 0 0 0 0 0 0 0 1 0 0

0 2 58 1 0 0 0 0 0 0 0 1 0 0

0 2 44 1 0 0 0 0 0 0 0 1 0 0

0 2 66 1 0 0 0 0 0 0 0 1 0 0

0 2 66 1 0 0 0 0 0 0 0 1 0 0

0 2 66 1 0 0 0 0 0 0 0 1 0 0

0 2 76 1 0 0 0 0 0 0 0 1 0 0

0 2 77 1 0 0 0 0 0 0 0 1 0 0

0 2 71 1 0 0 0 0 0 0 0 1 0 0

0 2 68 1 0 0 0 0 0 0 0 1 0 0

0 2 75 1 0 0 0 0 0 0 0 1 0 0

0 2 78 1 0 0 0 0 0 0 0 1 0 0

0 2 65 1 0 0 0 0 0 0 0 1 0 0

0 2 76 1 0 0 0 0 0 0 0 1 0 0

0 2 61 1 0 0 0 0 0 0 0 1 0 0

0 2 75 1 0 0 0 0 0 0 0 1 0 0

0 2 53 1 0 0 0 0 0 0 0 1 0 0

0 2 81 1 0 0 0 0 0 0 0 1 0 0

0 2 71 1 0 0 0 0 0 0 0 1 0 0

0 2 52 1 0 0 0 0 0 0 0 1 0 0

0 2 72 1 0 0 0 0 0 0 0 1 0 0

0 2 58 1 0 0 0 0 0 0 0 1 0 0

0 2 56 1 0 0 0 0 0 0 0 1 0 0

0 2 72 1 0 0 0 0 0 0 0 1 0 0

0 2 60 1 0 0 0 0 0 0 0 1 0 0

0 2 49 1 0 0 0 0 0 0 0 1 0 0

0 2 61 1 0 0 0 0 0 0 0 1 0 0

0 2 71 1 0 0 0 0 0 0 0 1 0 0

0 2 63 1 0 0 0 0 0 0 0 1 0 0

0 2 67 1 0 0 0 0 0 0 0 1 0 0

0 2 72 1 0 0 0 0 0 0 0 1 0 0

0 2 78 1 0 0 0 0 0 0 0 1 0 0

0 2 73 1 0 0 0 0 0 0 0 1 0 0

0 2 63 1 0 0 0 0 0 0 0 1 0 0

0 2 64 1 0 0 0 0 0 0 0 1 0 0

0 2 62 1 0 0 0 0 0 0 0 1 0 0

0 2 68 1 0 0 0 0 0 0 0 1 0 0

0 2 70 1 0 0 0 0 0 0 0 1 0 0

0 2 76 1 0 0 0 0 0 0 0 1 0 0

0 2 72 1 0 0 0 0 0 0 0 1 0 0

0 2 78 1 0 0 0 0 0 0 0 1 0 0

0 2 75 1 0 0 0 0 0 0 0 1 0 0

0 2 75 1 0 0 0 0 0 0 0 1 0 0

0 2 58 1 0 0 0 0 0 0 0 1 0 0

0 2 64 1 0 0 0 0 0 0 0 1 0 0

0 2 65 1 0 0 0 0 0 0 0 1 0 0

0 2 43 1 0 0 0 0 0 0 0 1 0 0

0 2 74 1 0 0 0 0 0 0 0 1 0 0

0 2 62 1 0 0 0 0 0 0 0 1 0 0

0 2 45 1 0 0 0 0 0 0 0 1 0 0

0 2 85 1 0 0 0 0 0 0 0 1 0 0

0 2 84 1 0 0 0 0 0 0 0 1 0 0

0 2 81 1 0 0 0 0 0 0 0 1 0 0

0 2 76 1 0 0 0 0 0 0 0 1 0 0

0 2 68 1 0 0 0 0 0 0 0 1 0 0

0 2 83 1 0 0 0 0 0 0 0 1 0 0

0 2 52 1 0 0 0 0 0 0 0 1 0 0

0 2 65 1 0 0 0 0 0 0 0 1 0 0

0 2 52 1 0 0 0 0 0 0 0 1 0 0

0 2 66 1 0 0 0 0 0 0 0 1 0 0

0 2 85 1 0 0 0 0 0 0 0 1 0 0

0 2 80 1 0 0 0 0 0 0 0 1 0 0

0 2 82 1 0 0 0 0 0 0 0 1 0 0

0 2 69 1 0 0 0 0 0 0 0 1 0 0

0 2 58 1 0 0 0 0 0 0 0 1 0 0

0 2 71 1 0 0 0 0 0 0 0 1 0 0

0 2 84 1 0 0 0 0 0 0 0 1 0 0

0 2 63 1 0 0 0 0 0 0 0 1 0 0

0 2 51 1 0 0 0 0 0 0 0 1 0 0

0 2 60 1 0 0 1 0 0 0 0 1 0 0

0 2 60 1 0 0 1 0 0 0 0 1 0 0

0 2 69 1 0 0 1 0 0 0 0 1 0 0

0 2 51 1 0 0 1 0 0 0 0 1 0 0

0 2 63 1 0 0 1 0 0 0 0 1 0 0

0 2 77 1 0 0 1 0 0 0 0 1 0 0

0 2 64 1 0 0 1 0 0 0 0 1 0 0

0 2 78 1 0 0 1 0 0 0 0 1 0 0

0 2 47 1 0 0 1 0 0 0 0 1 0 0

0 2 51 1 0 0 1 0 0 0 0 1 0 0

0 2 70 1 0 0 1 0 0 0 0 1 0 0

0 2 53 1 0 0 0 0 0 0 0 1 0 0

0 2 76 1 0 0 0 0 0 0 0 1 0 0

0 2 62 1 0 0 0 0 0 0 0 1 0 0

0 2 69 1 0 0 0 0 0 0 0 1 0 0

0 2 59 1 0 0 0 0 0 0 0 1 0 0

0 2 63 1 0 0 0 0 0 0 0 1 0 0

0 2 52 1 0 0 0 0 0 0 0 1 0 0

0 2 59 1 0 0 0 0 0 0 0 1 0 0

0 2 69 1 0 0 0 0 0 0 0 1 0 0

0 2 71 1 0 0 0 0 0 0 0 1 0 0

0 2 56 1 0 0 0 0 0 0 0 1 0 0

0 2 73 1 0 0 0 0 0 0 0 1 0 0

0 2 77 1 0 0 0 0 0 0 0 1 0 0

0 2 79 1 0 0 0 0 0 0 0 1 0 0

0 2 84 1 0 0 0 0 0 0 0 1 0 0

0 2 54 1 0 0 0 0 0 0 0 1 0 0

0 2 68 1 0 0 0 0 0 0 0 1 0 0

0 2 48 1 0 0 0 0 0 0 0 1 0 0

0 2 62 1 0 0 0 0 0 0 0 1 0 0

0 2 57 1 0 0 0 0 0 0 0 1 0 0

0 2 45 1 0 0 0 0 0 0 0 1 0 0

0 2 88 1 0 0 0 0 0 0 0 1 0 0

0 2 60 1 0 0 0 0 0 0 0 1 0 0

0 2 67 1 0 0 0 0 0 0 0 1 0 0

0 2 78 1 0 0 0 0 0 0 0 1 0 0

0 2 69 1 0 0 0 0 0 0 0 1 0 0

0 2 59 1 0 0 0 0 0 0 0 1 0 0

0 2 72 1 0 0 0 0 0 0 0 1 0 0

0 2 60 1 0 0 0 0 0 0 0 1 0 0

0 2 64 1 0 0 0 0 0 0 0 1 0 0

0 2 69 1 0 0 0 0 0 0 0 1 0 0

0 2 58 1 0 0 0 0 0 0 0 1 0 0

0 2 64 1 0 0 0 0 0 0 0 1 0 0

0 2 73 1 0 0 0 0 0 0 0 1 0 0

0 2 72 1 0 0 0 0 0 0 0 1 0 0

0 2 58 1 0 0 0 0 0 0 0 1 0 0

0 2 77 1 0 0 0 0 0 0 0 1 0 0

0 2 62 1 0 0 0 0 0 0 0 1 0 0

0 2 44 1 0 0 0 0 0 0 0 1 0 0

0 2 71 1 0 0 0 0 0 0 0 1 0 0

0 2 71 1 0 0 0 0 0 0 0 1 0 0

0 2 75 1 0 0 0 0 0 0 0 1 0 0

0 2 52 1 0 0 0 0 0 0 0 1 0 0

0 2 74 1 0 0 0 0 0 0 0 1 0 0

0 2 74 1 0 0 0 0 0 0 0 1 0 0

0 2 86 1 0 0 0 0 0 0 0 1 0 0

0 2 83 1 0 0 0 0 0 0 0 1 0 0

0 2 66 1 0 0 0 0 0 0 0 1 0 0

0 2 82 1 0 0 0 0 0 0 0 1 0 0

0 2 53 1 0 0 0 0 0 0 0 1 0 0

0 2 74 1 0 0 0 0 0 0 0 1 0 0

0 2 82 1 0 0 0 0 0 0 0 1 0 0

0 2 77 1 0 0 0 0 0 0 0 1 0 0

0 2 52 1 0 0 0 0 0 0 0 1 0 0

0 2 62 1 0 0 0 0 0 0 0 1 0 0

0 2 77 1 0 0 0 0 0 0 0 1 0 0

0 2 91 1 0 0 0 0 0 0 0 1 0 0

0 2 73 1 0 0 0 0 0 0 0 1 0 0

0 2 69 1 0 0 0 0 0 0 0 1 0 0

0 2 49 1 0 0 0 0 0 0 0 1 0 0

0 2 79 1 0 0 0 0 0 0 0 1 0 0

0 2 75 1 0 0 0 0 0 0 0 1 0 0

0 2 48 1 0 0 0 0 0 0 0 1 0 0

0 2 58 1 0 0 0 0 0 0 0 1 0 0

0 2 86 1 0 0 0 0 0 0 0 1 0 0

0 2 75 1 0 0 0 0 0 0 0 1 3 0

0 2 61 1 0 0 0 0 0 0 0 1 0 0

0 2 59 1 0 0 0 0 0 0 0 1 0 0

0 2 58 1 0 0 0 0 0 0 0 1 0 0

0 2 45 1 0 0 0 0 0 0 0 1 0 0

0 2 72 1 0 0 0 0 0 0 0 1 0 0

0 2 84 1 0 0 0 0 0 0 0 1 0 0

0 2 76 1 0 0 0 0 0 0 0 1 0 0

0 2 52 1 0 0 0 0 0 0 0 1 0 0

0 2 77 1 0 0 0 0 0 0 0 1 0 0

0 2 64 1 0 0 0 0 0 0 0 1 0 0

0 2 69 1 0 0 0 0 0 0 0 1 0 0

0 2 55 1 0 0 0 0 0 0 0 1 0 0

0 2 77 1 0 0 0 0 0 0 0 1 0 0

0 2 57 1 0 0 0 0 0 0 0 1 0 0

0 2 53 1 0 0 0 0 0 0 0 1 4 0

0 2 76 1 0 0 0 0 0 0 0 1 4 0

0 2 74 1 0 0 0 0 0 0 0 1 0 0

0 2 56 1 0 0 0 0 0 0 0 1 0 0

0 2 64 1 0 0 0 0 0 0 0 1 0 0

0 2 57 1 0 0 0 0 0 0 0 1 0 0

0 2 71 1 0 0 0 0 0 0 0 1 0 0

0 2 61 1 0 0 0 0 0 0 0 1 0 0

0 2 64 1 0 0 0 0 0 0 0 1 0 0

0 2 57 1 0 0 0 0 0 0 0 1 0 0

0 2 81 1 0 0 0 0 0 0 0 1 0 0

0 2 76 1 0 0 0 0 0 0 0 1 0 0

0 2 62 1 0 0 0 0 0 0 0 1 0 0

0 2 89 1 0 0 0 0 0 0 0 1 0 0

0 2 62 1 0 0 0 0 0 0 0 1 0 0

0 2 62 1 0 0 0 0 0 0 0 1 0 0

0 2 73 1 0 0 0 0 0 0 0 1 0 0

0 2 72 1 0 0 0 0 0 0 0 1 0 0

0 2 78 1 0 0 0 0 0 0 0 1 0 0

0 2 66 1 0 0 0 0 0 0 0 1 0 0

0 2 78 1 0 0 0 0 0 0 0 1 0 0

0 2 84 1 0 0 0 0 0 0 0 1 0 0

0 2 64 1 0 0 0 0 0 0 0 1 0 0

0 2 63 1 0 0 0 0 0 0 0 1 0 0

0 2 81 1 0 0 0 0 0 0 0 1 0 0

0 2 77 1 0 0 0 0 0 0 0 1 0 0

0 2 61 1 0 0 0 0 0 0 0 1 0 0

0 2 67 1 0 0 0 0 0 0 0 1 0 0

0 2 77 1 0 0 0 0 0 0 0 1 0 0

0 2 59 1 0 0 0 0 0 0 0 1 0 0

0 2 65 1 0 0 0 0 0 0 0 1 0 0

0 2 47 1 0 0 0 0 0 0 0 1 0 0

0 2 78 1 0 0 0 0 0 0 0 1 0 0

0 2 64 1 0 0 0 0 0 0 0 1 0 0

0 2 79 1 0 0 0 0 0 0 0 1 0 0

0 2 76 1 0 0 0 0 0 0 0 1 0 0

0 2 82 1 0 0 0 0 0 0 0 1 0 0

0 2 84 1 0 0 0 0 0 0 0 1 0 0

0 2 74 1 0 0 0 0 0 0 0 1 0 0

0 2 85 1 0 0 0 0 0 0 0 1 0 0

0 2 79 1 0 0 0 0 0 0 0 1 0 0

0 2 74 1 0 0 0 0 0 0 0 1 0 0

0 2 76 1 0 0 0 0 0 0 0 1 0 0

0 2 76 1 0 0 0 0 0 0 0 1 0 0

0 2 72 1 0 0 0 0 0 0 0 1 0 0

0 2 70 1 0 0 0 0 0 0 0 1 0 0

0 2 57 1 0 0 0 0 0 0 0 1 0 0

0 2 75 1 0 0 0 0 0 0 0 1 0 0

0 2 61 1 0 0 0 0 0 0 0 1 0 0

0 2 66 1 0 0 0 0 0 0 0 1 0 0

0 2 75 1 0 0 0 0 0 0 0 1 0 0

0 2 76 1 0 0 0 0 0 0 0 1 0 0

0 2 57 1 0 0 0 0 0 0 0 1 0 0

0 2 73 1 0 0 0 0 0 0 0 1 0 0

0 2 82 1 0 0 0 0 0 0 0 1 0 0

0 2 85 1 0 0 0 0 0 0 0 1 0 0

0 2 78 1 0 0 0 0 0 0 0 1 0 0

0 2 72 1 0 0 0 0 0 0 0 1 0 0

0 2 74 1 0 0 0 0 0 0 0 1 0 0

0 2 70 1 0 0 0 0 0 0 0 1 0 0

0 2 81 1 0 0 0 0 0 0 0 1 0 0

0 2 69 1 0 0 0 0 0 0 0 1 0 0

0 2 64 1 0 0 0 0 0 0 0 1 0 0

0 2 77 1 0 0 0 0 0 0 0 1 0 0

0 2 76 1 0 0 0 0 0 0 0 1 0 0

0 2 62 1 0 0 0 0 0 0 0 1 0 0

0 2 48 1 0 0 0 0 0 0 0 1 0 0

0 2 77 1 0 0 0 0 0 0 0 1 0 0

0 2 56 1 0 0 0 0 0 0 0 1 0 0

0 2 78 1 0 0 0 0 0 0 0 1 0 0

0 2 72 1 0 0 0 0 0 0 0 1 0 0

0 2 68 1 0 0 0 0 0 0 0 1 0 0

0 2 55 1 0 0 0 0 0 0 0 1 0 0

0 2 50 1 0 0 0 0 0 0 0 1 3 0

0 2 50 1 0 0 0 0 0 0 0 1 2 0

0 2 49 1 0 0 0 0 0 0 0 1 4 0

0 2 68 1 0 0 0 0 0 0 0 1 0 0

0 2 68 1 0 0 0 0 0 0 0 1 0 0

0 2 47 1 0 0 0 0 0 0 0 1 0 0

0 2 74 1 0 0 0 0 0 0 0 1 0 0

0 2 66 1 0 0 0 0 0 0 0 1 0 0

0 2 76 1 0 0 0 0 0 0 0 1 0 0

0 2 60 1 0 0 0 0 0 0 0 1 0 0

0 2 83 1 0 0 0 0 0 0 0 1 0 0

0 2 56 1 0 0 0 0 0 0 0 1 0 0

0 2 73 1 0 0 0 0 0 0 0 1 0 0

0 2 88 1 0 0 0 0 0 0 0 1 0 0

0 2 77 1 0 0 0 0 0 0 0 1 0 0

0 2 61 1 0 0 0 0 0 0 0 1 0 0

0 2 79 1 0 0 0 0 0 0 0 1 0 0

0 2 79 1 0 0 0 0 0 0 0 1 0 0

0 2 79 1 0 0 0 0 0 0 0 1 0 0

0 2 78 1 0 0 0 0 0 0 0 1 0 0

0 2 84 1 0 0 0 0 0 0 0 1 0 0

0 2 75 1 0 0 0 0 0 0 0 1 0 0

0 2 72 1 0 0 0 0 0 0 0 1 0 0

0 2 75 1 0 0 0 0 0 0 0 1 0 0

0 2 77 1 0 0 0 0 0 0 0 1 0 0

0 2 67 1 0 0 0 0 0 0 0 1 0 0

0 2 75 1 0 0 0 0 0 0 0 1 0 0

0 2 72 1 0 0 0 0 0 0 0 1 0 0

0 2 84 1 0 0 0 0 0 0 0 1 0 0

0 2 79 1 0 0 0 0 0 0 0 1 0 0

0 2 70 1 0 0 0 0 0 0 0 1 0 0

0 2 76 1 0 0 0 0 0 0 0 1 0 0

0 2 77 1 0 0 0 0 0 0 0 1 0 0

0 2 76 1 0 0 0 0 0 0 0 1 0 0

0 2 77 1 0 0 0 0 0 0 0 1 0 0

0 2 84 1 0 0 0 0 0 0 0 1 0 0

0 2 92 1 0 0 0 0 0 0 0 1 0 0

0 2 75 1 0 0 0 0 0 0 0 1 0 0

0 2 87 1 0 0 0 0 0 0 0 1 0 0

0 2 69 1 0 0 0 0 0 0 0 1 0 0

0 2 79 1 0 0 0 0 0 0 0 1 0 0

0 2 61 1 0 0 0 0 0 0 0 1 0 0

0 2 84 1 0 0 0 0 0 0 0 1 0 0

0 2 74 1 0 0 0 0 0 0 0 1 0 0

0 2 55 1 0 0 0 0 0 0 0 1 0 0

0 2 75 1 0 0 0 0 0 0 0 1 0 0

0 2 67 1 0 0 0 0 0 0 0 1 0 0

0 2 63 1 0 0 0 0 0 0 0 1 0 0

0 2 58 1 0 0 0 0 0 0 0 1 0 0

0 2 58 1 0 0 0 0 0 0 0 1 0 0

0 2 60 1 0 0 0 0 0 0 0 1 0 0

0 2 61 1 0 0 0 0 0 0 0 1 0 0

0 2 57 1 0 0 0 0 0 0 0 1 0 0

0 2 43 1 0 0 0 0 0 0 0 1 0 0

0 2 61 1 0 0 0 0 0 0 0 1 0 0

0 2 58 1 0 0 0 0 0 0 0 1 0 0

0 2 73 1 0 0 0 0 0 0 0 1 0 0

0 2 65 1 0 0 0 0 0 0 0 1 0 0

0 2 57 1 0 0 0 0 0 0 0 1 0 0

0 2 64 1 0 0 0 0 0 0 0 1 0 0

0 2 74 1 0 0 0 0 0 0 0 1 0 0

0 2 84 1 0 0 0 0 0 0 0 1 0 0

0 2 77 1 0 0 0 0 0 0 0 1 0 0

0 2 67 1 0 0 0 0 0 0 0 1 0 0

0 2 79 1 0 0 0 0 0 0 0 1 0 0

0 2 60 1 0 0 0 0 0 0 0 1 0 0

0 2 59 1 0 0 0 0 0 0 0 1 0 0

0 2 75 1 0 0 0 0 0 0 0 1 0 0

0 2 74 1 0 0 0 0 0 0 0 1 0 0

0 2 76 1 0 0 0 0 0 0 0 1 0 0

0 2 76 1 0 0 0 0 0 0 0 1 0 0

0 2 82 1 0 0 0 0 0 0 0 1 0 0

0 2 66 1 0 0 0 0 0 0 0 1 0 0

0 2 72 1 0 0 0 0 0 0 0 1 0 0

0 2 67 1 0 0 0 0 0 0 0 1 0 0

0 2 77 1 0 0 0 0 0 0 0 1 0 0

0 2 73 1 0 0 0 0 0 0 0 1 0 0

0 2 71 1 0 0 0 0 0 0 0 1 0 0

0 2 57 1 0 0 0 0 0 0 0 1 0 0

0 2 77 1 0 0 0 0 0 0 0 1 0 0

0 2 71 1 0 0 0 0 0 0 0 1 0 0

0 2 34 1 0 0 0 0 0 0 0 1 0 0

0 2 62 1 0 0 0 0 0 0 0 1 0 0

0 2 75 1 0 0 0 0 0 0 0 1 0 0

0 2 60 1 0 0 0 0 0 0 0 1 0 0

0 2 77 1 0 0 0 0 0 0 0 1 0 0

0 2 62 1 0 0 0 0 0 0 0 1 0 0

0 2 70 1 0 0 0 0 0 0 0 1 0 0

0 2 70 1 0 0 0 0 0 0 0 1 0 0

0 2 68 1 0 0 0 0 0 0 0 1 0 0

0 2 54 1 0 0 0 0 0 0 0 1 0 0

0 2 81 1 0 0 0 0 0 0 0 1 0 0

0 2 55 1 0 0 0 0 0 0 0 1 0 0

0 2 58 1 0 0 0 0 0 0 0 1 0 0

0 2 61 1 0 0 0 0 0 0 0 1 0 0

0 2 52 1 0 0 0 0 0 0 0 1 0 0

0 2 66 1 0 0 0 0 0 0 0 1 0 0

0 2 75 1 0 0 0 0 0 0 0 1 0 0

0 2 79 1 0 0 0 0 0 0 0 1 0 0

0 2 72 1 0 0 0 0 0 0 0 1 0 0

0 2 53 1 0 0 0 0 0 0 0 1 0 0

0 2 58 1 0 0 0 0 0 0 0 1 0 0

0 2 59 1 0 0 0 0 0 0 0 1 0 0

0 2 59 1 0 0 0 0 0 0 0 1 0 0

0 2 83 1 0 0 0 0 0 0 0 1 0 0

0 2 74 1 0 0 0 0 0 0 0 1 0 0

0 2 74 1 0 0 0 0 0 0 0 1 0 0

0 2 62 1 0 0 0 0 0 0 0 1 0 0

0 2 46 1 0 0 0 0 0 0 0 1 0 0

0 2 74 1 0 0 0 0 0 0 0 1 0 0

0 2 69 1 0 0 0 0 0 0 0 1 0 0

0 2 63 1 0 0 0 0 0 0 0 1 0 0

0 2 78 1 0 0 0 0 0 0 0 1 0 0

0 2 62 1 0 0 0 0 0 0 0 1 0 0

0 2 77 1 0 0 0 0 0 0 0 1 0 0

0 2 70 1 0 0 0 0 0 0 0 1 0 0

0 2 60 1 0 0 0 0 0 0 0 1 0 0

0 2 67 1 0 0 0 0 0 0 0 1 0 0

0 2 76 1 0 0 0 0 0 0 0 1 0 0

0 2 78 1 0 0 0 0 0 0 0 1 0 0

0 2 67 1 0 0 0 0 0 0 0 1 0 0

0 2 65 1 0 0 0 0 0 0 0 1 0 0

0 2 56 1 0 0 0 0 0 0 0 1 0 0

0 2 76 1 0 0 0 0 0 0 0 1 0 0

0 2 61 1 0 0 0 0 0 0 0 1 0 0

0 2 77 1 0 0 0 0 0 0 0 1 0 0

0 2 77 1 0 0 0 0 0 0 0 1 0 0

0 2 51 1 0 0 0 0 0 0 0 1 0 0

0 2 67 1 0 0 0 0 0 0 0 1 0 0

0 2 54 1 0 0 0 0 0 0 0 1 0 0

0 2 45 1 0 0 0 0 0 0 0 1 0 0

0 2 59 1 0 0 0 0 0 0 0 1 0 0

0 2 72 1 0 0 0 0 0 0 0 1 0 0

0 2 72 1 0 0 0 0 0 0 0 1 0 0

0 2 48 1 0 0 0 0 0 0 0 1 0 0

0 2 57 1 0 0 0 0 0 0 0 1 0 0

0 2 71 1 0 0 0 0 0 0 0 1 0 0

0 2 60 1 0 0 0 0 0 0 0 1 0 0

0 2 55 1 0 0 0 0 0 0 0 1 0 0

0 2 77 1 0 0 0 0 0 0 0 1 0 0

0 2 77 1 0 0 0 0 0 0 0 1 0 0

0 2 54 1 0 0 0 0 0 0 0 1 0 0

0 2 60 1 0 0 0 0 0 0 0 1 0 0

0 2 50 1 0 0 0 0 0 0 0 1 0 0

0 2 69 1 0 0 0 0 0 0 0 1 0 0

0 2 49 1 0 0 0 0 0 0 0 1 0 0

0 2 48 1 0 0 0 0 0 0 0 1 0 0

0 2 59 1 0 0 0 0 0 0 0 1 0 0

0 2 81 1 0 0 0 0 0 0 0 1 0 0

0 2 73 1 0 0 0 0 0 0 0 1 0 0

0 2 79 1 0 0 0 0 0 0 0 1 0 0

0 2 78 1 0 0 0 0 0 0 0 1 0 0

0 2 70 1 0 0 0 0 0 0 0 1 0 0

0 2 56 1 0 0 0 0 0 0 0 1 0 0

0 2 65 1 0 0 0 0 0 0 0 1 0 0

0 2 53 1 0 0 0 0 0 0 0 1 0 0

0 2 74 1 0 0 0 0 0 0 0 1 0 0

0 2 81 1 0 0 0 0 0 0 0 1 0 0

0 2 81 1 0 0 0 0 0 0 0 1 0 0

0 2 61 1 0 0 0 0 0 0 0 1 0 0

0 2 72 1 0 0 0 0 0 0 0 1 0 0

0 2 69 1 0 0 0 0 0 0 0 1 0 0

0 2 59 1 0 0 0 0 0 0 0 1 0 0

0 2 71 1 0 0 0 0 0 0 0 1 0 0

0 2 60 1 0 0 0 0 0 0 0 1 0 0

0 2 58 1 0 0 0 0 0 0 0 1 0 0

0 2 71 1 0 0 0 0 0 0 0 1 0 0

0 2 76 1 0 0 0 0 0 0 0 1 0 0

0 2 58 1 0 0 0 0 0 0 0 1 0 0

0 2 55 1 0 0 0 0 0 0 0 1 0 0

0 2 67 1 0 0 0 0 0 0 0 1 0 0

0 2 74 1 0 0 0 0 0 0 0 1 0 0

0 2 72 1 0 0 0 0 0 0 0 1 0 0

0 2 79 1 0 0 0 0 0 0 0 1 0 0

0 2 74 1 0 0 0 0 0 0 0 1 0 0

0 2 68 1 0 0 0 0 0 0 0 1 0 0

0 2 69 1 0 0 0 0 0 0 0 1 0 0

0 2 75 1 0 0 0 0 0 0 0 1 0 0

0 2 50 1 0 0 0 0 0 0 0 1 0 0

0 2 76 1 0 0 0 0 0 0 0 1 0 0

0 2 76 1 0 0 0 0 0 0 0 1 0 0

0 2 75 1 0 0 0 0 0 0 0 1 0 0

0 2 64 1 0 0 0 0 0 0 0 1 0 0

0 2 76 1 0 0 0 0 0 0 0 1 0 0

0 2 64 1 0 0 0 0 0 0 0 1 0 0

0 2 69 1 0 0 0 0 0 0 0 1 0 0

0 2 85 1 0 0 0 0 0 0 0 1 0 0

0 2 83 1 0 0 0 0 0 0 0 1 0 0

0 2 61 1 0 0 0 0 0 0 0 1 0 0

0 2 64 1 0 0 0 0 0 0 0 1 0 0

0 2 79 1 0 0 0 0 0 0 0 1 0 0

0 2 66 1 0 0 0 0 0 0 0 1 0 0

0 2 50 1 0 0 0 0 0 0 0 1 0 0

0 2 73 1 0 0 0 0 0 0 0 1 0 0

0 2 64 1 0 0 0 0 0 0 0 1 0 0

0 2 59 1 0 0 0 0 0 0 0 1 0 0

0 2 72 1 0 0 0 0 0 0 0 1 0 0

0 2 71 1 0 0 0 0 0 0 0 1 0 0

0 2 63 1 0 0 0 0 0 0 0 1 0 0

0 2 62 1 0 0 0 0 0 0 0 1 0 0

0 2 50 1 0 0 0 0 0 0 0 1 0 0

0 2 79 1 0 0 0 0 0 0 0 1 0 0

0 2 76 1 0 0 0 0 0 0 0 1 0 0

0 2 62 1 0 0 0 0 0 0 0 1 0 0

0 2 56 1 0 0 0 0 0 0 0 1 0 0

0 2 52 1 0 0 0 0 0 0 0 1 0 0

0 2 64 1 0 0 0 0 0 0 0 1 0 0

0 2 75 1 0 0 0 0 0 0 0 1 0 0

0 2 85 1 0 0 0 0 0 0 0 1 0 0

0 2 69 1 0 0 0 0 0 0 0 1 0 0

0 2 73 1 0 0 0 0 0 0 0 1 0 0

0 2 72 1 0 0 0 0 0 0 0 1 0 0

0 2 76 1 0 0 0 0 0 0 0 1 0 0

0 2 89 1 0 0 0 0 0 0 0 1 0 0

0 2 65 1 0 0 0 0 0 0 0 1 0 0

0 2 60 1 0 0 0 0 0 0 0 1 0 0

0 2 83 1 0 0 0 0 0 0 0 1 0 0

0 2 80 1 0 0 0 0 0 0 0 1 0 0

0 2 77 1 0 0 0 0 0 0 0 1 0 0

0 2 85 1 0 0 0 0 0 0 0 1 0 0

0 2 79 1 0 0 0 0 0 0 0 1 0 0

0 2 76 1 0 0 0 0 0 0 0 1 0 0

0 2 56 1 0 0 0 0 0 0 0 1 0 0

0 2 85 1 0 0 0 0 0 0 0 1 0 0

0 2 72 1 0 0 0 0 0 0 0 1 0 0

0 2 56 1 0 0 0 0 0 0 0 1 0 0

0 2 77 1 0 0 0 0 0 0 0 1 0 0

0 2 45 1 0 0 0 0 0 0 0 1 0 0

0 2 50 1 0 0 0 0 0 0 0 1 0 0

0 2 70 1 0 0 0 0 0 0 0 1 0 0

0 2 73 1 0 0 0 0 0 0 0 1 0 0

0 2 87 1 0 0 0 0 0 0 0 1 0 0

0 2 60 1 0 0 0 0 0 0 0 1 0 0

0 2 60 1 0 0 0 0 0 0 0 1 0 0

0 2 80 1 0 0 0 0 0 0 0 1 0 0

0 2 85 1 0 0 0 0 0 0 0 1 0 0

0 2 82 1 0 0 0 0 0 0 0 1 0 0

0 2 77 1 0 0 0 0 0 0 0 1 0 0

0 2 87 1 0 0 0 0 0 0 0 1 0 0

0 2 77 1 0 0 0 0 0 0 0 1 0 0

0 2 62 1 0 0 0 0 0 0 0 1 0 0

0 2 57 1 0 0 0 0 0 0 0 1 0 0

0 2 79 1 0 0 0 0 0 0 0 1 0 0

0 2 71 1 0 0 0 0 0 0 0 1 0 0

0 2 70 1 0 0 0 0 0 0 0 1 0 0

0 2 92 1 0 0 0 0 0 0 0 1 0 0

0 2 83 1 0 0 0 0 0 0 0 1 0 0

0 2 81 1 0 0 0 0 0 0 0 1 0 0

0 2 66 1 0 0 0 0 0 0 0 1 0 0

0 2 56 1 0 0 0 0 0 0 0 1 0 0

0 2 52 1 0 0 0 0 0 0 0 1 0 0

0 2 76 1 0 0 0 0 0 0 0 1 0 0

0 2 56 1 0 0 0 0 0 0 0 1 0 0

0 2 55 1 0 0 0 0 0 0 0 1 0 0

0 2 60 1 0 0 0 0 0 0 0 1 0 0

0 2 58 1 0 0 0 0 0 0 0 1 0 0

0 2 57 1 0 0 0 0 0 0 0 1 0 0

0 2 62 1 0 0 0 0 0 0 0 1 0 0

0 2 62 1 0 0 0 0 0 0 0 1 0 0

0 2 60 1 0 0 0 0 0 0 0 1 0 0

0 2 77 1 0 0 0 0 0 0 0 1 0 0

0 2 65 1 0 0 0 0 0 0 0 1 0 0

0 2 49 1 0 0 0 0 0 0 0 1 0 0

0 2 54 1 0 0 0 0 0 0 0 1 0 0

0 2 72 1 0 0 0 0 0 0 0 1 0 0

0 2 53 1 0 0 0 0 0 0 0 1 0 0

0 2 48 1 0 0 0 0 0 0 0 1 0 0

0 2 77 1 0 0 0 0 0 0 0 1 0 0

0 2 76 1 0 0 0 0 0 0 0 1 0 0

0 2 84 1 0 0 0 0 0 0 0 1 0 0

0 2 73 1 0 0 0 0 0 0 0 1 0 0

0 2 71 1 0 0 0 0 0 0 0 1 0 0

0 2 81 1 0 0 0 0 0 0 0 1 0 0

0 2 61 1 0 0 0 0 0 0 0 1 0 0

0 2 70 1 0 0 0 0 0 0 0 1 0 0

0 2 56 1 0 0 0 0 0 0 0 1 0 0

0 2 73 1 0 0 0 0 0 0 0 1 0 0

0 2 56 1 0 0 0 0 0 0 0 1 0 0

0 2 65 1 0 0 0 0 0 0 0 1 0 0

0 2 62 1 0 0 0 0 0 0 0 1 0 0

0 2 74 1 0 0 0 0 0 0 0 1 0 0

0 2 69 1 0 0 0 0 0 0 0 1 0 0

0 2 56 1 0 0 0 0 0 0 0 1 0 0

0 2 53 1 0 0 0 0 0 0 0 1 0 0

0 2 49 1 0 0 0 0 0 0 0 1 0 0

0 2 59 1 0 0 0 0 0 0 0 1 0 0

0 2 83 1 0 0 0 0 0 0 0 1 0 0

0 2 76 1 0 0 0 0 0 0 0 1 0 0

0 2 65 1 0 0 0 0 0 0 0 1 0 0

0 2 53 1 0 0 0 0 0 0 0 1 0 0

0 2 75 1 0 0 0 0 0 0 0 1 0 0

0 2 90 1 0 0 0 0 0 0 0 1 0 0

0 2 70 1 0 0 0 0 0 0 0 1 0 0

0 2 76 1 0 0 0 0 0 0 0 1 0 0

0 2 60 1 0 0 0 0 0 0 0 1 0 0

0 2 70 1 0 0 0 0 0 0 0 1 0 0

0 2 61 1 0 0 0 0 0 0 0 1 0 0

0 2 73 1 0 0 0 0 0 0 0 1 0 0

0 2 74 1 0 0 0 0 0 0 0 1 0 0

0 2 56 1 0 0 0 0 0 0 0 1 0 0

0 2 64 1 0 0 0 0 0 0 0 1 0 0

0 2 44 1 0 0 0 0 0 0 0 1 0 0

0 2 65 1 0 0 0 0 0 0 0 1 0 0

0 2 70 1 0 0 0 0 0 0 0 1 0 0

0 2 55 1 0 0 0 0 0 0 0 1 0 0

0 2 75 1 0 0 0 0 0 0 0 1 0 0

0 2 59 1 0 0 0 0 0 0 0 1 0 0

0 2 63 1 0 0 0 0 0 0 0 1 0 0

0 2 53 1 0 0 0 0 0 0 0 1 0 0

0 2 60 1 0 0 0 0 0 0 0 1 0 0

0 2 70 1 0 0 0 0 0 0 0 1 0 0

0 2 75 1 0 0 0 0 0 0 0 1 0 0

0 2 65 1 0 0 0 0 0 0 0 1 0 0

0 2 60 1 0 0 0 0 0 0 0 1 0 0

0 2 71 1 0 0 0 0 0 0 0 1 0 0

0 2 71 1 0 0 0 0 0 0 0 1 0 0

0 2 73 1 0 0 0 0 0 0 0 1 0 0

0 2 71 1 0 0 0 0 0 0 0 1 0 0

0 2 78 1 0 0 0 0 0 0 0 1 0 0

0 2 84 1 0 0 0 0 0 0 0 1 0 0

0 2 73 1 0 0 0 0 0 0 0 1 0 0

0 2 70 1 0 0 0 0 0 0 0 1 0 0

0 2 63 1 0 0 0 0 0 0 0 1 0 0

0 2 54 1 0 0 0 0 0 0 0 1 0 0

0 2 64 1 0 0 0 0 0 0 0 1 0 0

0 2 52 1 0 0 0 0 0 0 0 1 0 0

0 2 71 1 0 0 0 0 0 0 0 1 0 0

0 2 77 1 0 0 0 0 0 0 0 1 0 0

0 2 59 1 0 0 0 0 0 0 0 1 0 0

0 2 60 1 0 0 0 0 0 0 0 1 0 0

0 2 19 1 0 0 0 0 0 0 0 1 0 0

0 2 55 1 0 0 1 0 0 0 0 1 0 0

0 2 60 1 0 0 0 0 0 0 0 1 0 0

0 2 58 1 0 0 0 0 0 0 0 1 0 0

0 2 51 1 0 0 0 0 0 0 0 1 0 0

0 2 79 1 0 0 0 0 0 0 0 1 0 0

0 2 77 1 0 0 0 0 0 0 0 1 0 0

0 2 71 1 0 0 0 0 0 0 0 1 0 0

0 2 57 1 0 0 0 0 0 0 0 1 0 0

0 2 59 1 0 0 0 0 0 0 0 1 0 0

0 2 77 1 0 0 0 0 0 0 0 1 0 0

0 2 77 1 0 0 0 0 0 0 0 1 0 0

0 2 82 1 0 0 0 0 0 0 0 1 0 0

0 2 77 1 0 0 0 0 0 0 0 1 0 0

0 2 54 1 0 0 0 0 0 0 0 1 4 0

0 2 59 1 0 0 0 0 0 0 0 1 4 0

0 2 58 1 0 0 0 0 0 0 0 1 4 0

0 2 59 1 0 0 0 0 0 0 0 1 4 0

0 2 79 1 0 0 0 0 0 0 0 1 4 0

0 2 67 1 0 0 0 0 0 0 0 1 4 0

0 2 60 1 0 0 0 0 0 0 0 1 4 0

0 2 48 1 0 0 0 0 0 0 0 1 4 0

0 2 73 1 0 0 0 0 0 0 0 1 0 0

0 2 70 1 0 0 0 0 0 0 0 1 0 0

0 2 71 1 0 0 0 0 0 0 0 1 0 0

0 2 55 1 0 0 0 0 0 0 0 1 3 0

0 2 55 1 0 0 0 0 0 0 0 1 4 0

0 2 52 1 0 0 0 0 0 0 0 1 4 0

0 2 56 1 0 0 0 0 0 0 0 1 4 0

0 2 51 1 0 0 0 0 0 0 0 1 4 0

0 2 79 1 0 0 0 0 0 0 0 1 0 0

0 2 73 1 0 0 0 0 0 0 0 1 0 0

0 2 48 1 0 0 0 0 0 0 0 1 0 0

0 2 51 1 0 0 0 0 0 0 0 1 0 0

0 2 57 1 0 0 0 0 0 0 0 1 0 0

0 2 61 1 0 0 0 0 0 0 0 1 0 0

0 2 79 1 0 0 0 0 0 0 0 1 0 0

0 2 69 1 0 0 0 0 0 0 0 1 0 0

0 2 67 1 0 0 0 0 0 0 0 1 0 0

0 2 53 1 0 0 0 0 0 0 0 1 0 0

0 2 76 1 0 0 0 0 0 0 0 1 0 0

0 2 81 1 0 0 0 0 0 0 0 1 0 0

0 2 77 1 0 0 0 0 0 0 0 1 0 0

0 2 76 1 0 0 0 0 0 0 0 1 0 0

0 2 57 1 0 0 0 0 0 0 0 1 0 0

0 2 74 1 0 0 0 0 0 0 0 1 0 0

0 2 67 1 0 0 0 0 0 0 0 1 0 0

0 2 74 1 0 0 0 0 0 0 0 1 0 0

0 2 82 1 0 0 0 0 0 0 0 1 0 0

0 2 76 1 0 0 0 0 0 0 0 1 0 0

0 2 74 1 0 0 0 0 0 0 0 1 0 0

0 2 65 1 0 0 0 0 0 0 0 1 0 0

0 2 76 1 0 0 0 0 0 0 0 1 0 0

0 2 89 1 0 0 0 0 0 0 0 1 0 0

0 2 56 1 0 0 0 0 0 0 0 1 0 0

0 2 64 1 0 0 0 0 0 0 0 1 0 0

0 2 59 1 0 0 0 0 0 0 0 1 0 0

0 2 64 1 0 0 0 0 0 0 0 1 0 0

0 2 59 1 0 0 0 0 0 0 0 1 2 0

0 2 78 1 0 0 0 0 0 0 0 1 2 0

0 2 59 1 0 0 0 0 0 0 0 1 2 0

0 2 60 1 0 0 0 0 0 0 0 1 2 0

0 2 59 1 0 0 0 0 0 0 0 1 2 0

0 2 76 1 0 0 0 0 0 0 0 1 2 0

0 2 59 1 0 0 0 0 0 0 0 1 2 0

0 2 60 1 0 0 0 0 0 0 0 1 2 0

1 2 69 0 1 0 0 0 0 1 0 1 2 0

1 2 69 0 1 0 0 0 0 1 0 1 2 0

1 1 68 0 1 0 0 0 0 1 0 1 2 0

1 1 75 0 1 0 0 0 0 1 0 1 2 0

1 1 61 0 1 0 0 0 0 1 0 1 2 0

1 2 79 0 1 0 0 0 0 0 0 1 2 0

1 2 68 0 1 0 1 0 0 0 0 1 2 0

1 2 79 0 1 0 0 0 0 0 0 1 2 0

1 2 62 0 1 0 0 0 0 0 0 1 2 0

1 2 62 0 1 0 0 0 0 0 0 1 2 0

1 2 68 0 1 0 0 0 0 0 0 1 2 0

1 2 70 0 1 0 0 0 0 0 0 1 2 0

1 1 55 0 0 0 0 0 0 0 0 1 2 0

1 1 54 0 0 0 0 0 0 0 0 1 2 0

1 1 58 0 0 0 0 0 0 0 0 1 2 0

1 1 15 0 0 0 0 0 0 0 0 1 2 0

1 1 37 0 0 0 0 0 0 0 0 1 2 0

1 1 79 0 0 0 0 0 0 0 0 1 2 0

1 1 28 0 0 0 0 0 0 0 0 1 2 0

1 1 58 0 0 1 0 0 0 0 0 1 2 0

1 1 65 0 0 1 0 0 0 0 0 1 2 0

1 1 48 0 0 1 0 0 0 0 0 1 2 0

1 1 70 0 0 1 0 0 0 0 0 1 2 0

1 2 40 0 0 1 0 0 0 0 0 1 2 0

1 2 59 0 0 1 0 0 0 0 0 1 2 0

1 2 55 0 0 1 0 0 0 0 0 1 2 0

1 1 86 0 0 0 0 0 0 0 0 1 2 0

1 1 89 0 0 0 0 0 0 0 0 1 2 0

1 1 86 0 0 0 0 0 0 0 0 1 2 0

1 1 78 0 0 0 0 0 0 0 0 1 2 0

1 1 59 0 0 0 0 0 0 0 0 1 2 0

1 1 79 0 0 0 0 0 0 0 0 1 2 0

1 1 83 0 0 0 0 0 0 0 0 1 2 0

1 1 81 0 0 0 0 0 0 0 0 1 2 0

1 1 78 0 0 0 0 0 0 0 0 1 2 0

1 1 82 0 0 0 0 0 0 0 0 1 2 0

1 1 70 0 0 0 0 0 0 0 0 1 2 0

1 1 84 0 0 0 0 0 0 0 0 1 2 0

1 1 75 0 0 0 0 0 0 0 0 1 2 0

1 1 75 0 0 0 0 0 0 0 0 1 2 0

1 1 84 0 0 0 0 0 0 0 0 1 2 0

1 1 56 0 0 0 0 0 0 0 0 1 2 0

1 1 84 0 0 0 0 0 0 0 0 1 2 0

1 1 88 0 0 0 0 0 0 0 0 1 2 0

1 1 63 0 0 0 0 0 0 0 0 1 2 0

1 1 80 0 0 0 0 0 0 0 0 1 2 0

1 1 89 0 0 0 0 0 0 0 0 1 2 0

1 1 74 0 0 0 0 0 0 0 0 1 2 0

1 1 55 0 0 0 0 0 0 0 0 1 2 0

1 1 64 0 0 0 0 0 0 0 0 1 2 0

1 1 65 0 0 0 1 0 0 0 0 1 2 0

1 1 70 0 0 0 0 0 0 0 0 1 2 0

1 1 55 0 0 0 0 0 0 0 0 1 2 0

1 1 54 0 0 0 0 0 0 0 0 1 2 0

1 1 79 0 0 0 0 0 0 0 0 1 2 0

1 1 55 0 0 0 0 0 0 0 0 1 2 0

1 1 88 0 0 0 0 0 0 0 0 1 2 0

1 1 60 0 0 0 0 0 0 0 0 1 2 0

1 1 59 0 0 0 0 0 0 0 0 1 2 0

1 1 54 0 0 0 0 0 0 0 0 1 2 0

1 1 83 0 0 0 0 0 0 0 0 1 2 0

1 1 84 0 0 0 0 0 0 0 0 1 2 0

1 1 79 0 0 0 0 0 0 0 0 1 2 0

1 1 39 0 0 0 0 0 0 0 0 1 2 0

1 1 85 0 0 0 0 0 0 0 0 1 2 0

1 1 76 0 0 0 0 0 0 0 0 1 2 0

1 1 81 0 0 0 0 0 0 0 0 1 2 0

1 1 86 0 0 0 0 0 0 0 0 1 2 0

1 1 87 0 0 0 0 0 0 0 0 1 2 0

1 1 75 0 0 0 0 0 0 0 0 1 2 0

1 1 65 0 0 0 0 0 0 0 0 1 2 0

1 1 79 0 0 0 0 0 0 0 0 1 3 0

1 1 29 0 0 0 0 0 0 0 0 1 3 0

1 1 30 0 0 0 1 0 0 0 0 1 3 0

1 1 96 0 0 0 0 0 0 0 0 1 3 0

1 1 76 0 0 0 0 0 0 0 0 1 3 0

1 1 72 0 0 0 0 0 0 0 0 1 3 0

1 1 59 0 0 0 0 0 0 0 0 1 3 0

1 1 72 0 0 0 0 0 0 0 0 1 3 0

1 1 82 0 0 0 0 0 0 0 0 1 3 0

1 1 62 0 0 0 0 0 0 0 0 1 3 0

1 1 65 0 0 0 0 0 0 0 0 1 3 0

1 1 70 0 0 0 0 0 0 0 0 1 3 0

1 1 47 0 0 0 0 0 0 0 0 1 3 0

1 1 79 0 0 0 0 0 0 0 0 1 3 0

1 1 87 0 0 0 0 0 0 0 0 1 3 0

1 1 42 0 0 0 0 0 0 0 0 1 3 0

1 1 62 0 0 0 0 0 0 0 0 1 3 0

1 1 83 0 0 0 0 0 0 0 0 1 3 0

1 1 38 0 0 0 0 0 0 0 0 1 3 0

1 1 47 0 0 0 0 0 0 0 0 1 3 0

1 1 63 0 0 0 0 0 0 0 0 1 3 0

1 1 24 0 0 0 0 0 0 0 0 1 3 0

1 1 84 0 0 0 0 0 0 0 0 1 3 0

1 1 82 0 0 0 0 0 0 0 0 1 3 0

1 1 59 0 0 0 0 0 0 0 0 1 3 0

1 1 54 0 0 0 0 0 0 0 0 1 3 0

1 1 81 0 0 0 0 0 0 0 0 1 3 0

1 1 44 0 0 0 0 0 0 0 0 1 3 0

1 1 42 0 0 0 0 0 0 0 0 1 3 0

1 1 72 0 0 0 0 0 0 0 0 1 3 0

1 1 44 0 0 0 0 0 0 0 0 1 3 0

1 1 70 0 0 0 0 0 0 0 0 1 3 0

1 1 39 0 0 0 0 0 0 0 0 1 3 0

1 1 49 0 0 0 0 0 0 0 0 1 3 0

1 1 75 0 0 0 0 0 0 0 0 1 4 0

1 1 58 0 0 0 0 0 0 0 0 1 4 0

1 1 89 0 0 0 0 0 0 0 0 1 4 0

1 1 79 0 0 0 0 0 0 0 0 1 4 0

1 1 46 0 0 0 0 0 0 0 0 1 4 0

1 1 57 0 0 0 0 0 0 0 0 1 4 0

1 1 64 0 0 0 0 0 0 0 0 1 4 0

1 1 85 0 0 0 0 0 0 0 0 1 4 0

1 1 83 0 0 0 0 0 0 0 0 1 4 0

1 1 85 0 0 0 0 0 0 0 0 1 4 0

1 1 38 0 0 0 0 0 0 0 0 1 4 0

1 1 82 0 0 0 0 0 0 0 0 1 4 0

1 1 89 0 0 0 0 0 0 0 0 1 4 0

1 1 54 0 0 0 0 0 0 0 0 1 4 0

1 1 48 0 0 0 0 0 0 0 0 1 0 0

1 1 70 0 0 0 0 0 0 0 0 1 0 0

1 1 57 0 0 0 0 0 0 0 0 1 0 0

1 1 59 0 0 0 0 0 0 0 0 1 0 0

1 1 63 0 0 0 0 0 0 0 0 1 0 0

1 1 80 0 0 0 0 0 0 0 0 1 2 0

1 1 80 0 0 0 0 0 0 0 0 1 2 0

1 1 39 0 0 0 0 0 0 0 0 1 2 0

1 1 55 0 0 0 0 0 0 0 0 1 2 0

1 1 68 0 0 0 0 0 0 0 0 1 2 0

1 1 62 0 0 0 0 0 0 0 0 1 2 0

1 1 75 0 0 0 0 0 0 0 0 1 2 0

1 1 80 0 0 0 0 0 0 0 0 1 3 0

1 1 75 0 0 0 0 0 0 0 0 1 3 0

1 2 63 0 0 0 0 0 0 0 0 1 3 0

1 2 56 0 0 0 0 0 0 0 0 1 0 0

1 2 58 0 0 0 0 0 0 0 0 1 0 0

1 2 67 0 0 0 0 0 0 0 0 1 0 0

1 2 76 0 0 0 0 0 0 0 0 1 0 0

1 2 66 0 0 0 0 0 0 0 0 1 0 0

1 2 53 0 0 0 0 0 0 0 0 1 0 0

1 2 52 0 0 0 0 0 0 0 0 1 0 0

1 2 46 0 0 0 0 0 0 0 0 1 0 0

1 2 76 0 0 0 0 0 0 0 0 1 2 0

1 2 87 0 0 0 0 0 0 0 0 1 4 0

1 2 75 0 0 0 0 0 0 0 0 1 0 0

1 2 59 0 0 0 0 0 0 0 0 1 0 0

1 2 66 0 0 0 0 0 0 0 0 1 0 0

1 2 71 0 0 0 0 0 0 0 0 1 0 0

1 2 69 0 0 0 0 0 0 0 0 1 0 0

1 2 50 0 0 0 1 0 0 0 0 1 0 0

1 2 66 0 0 0 0 0 0 0 0 1 0 0

1 2 68 0 0 0 0 0 0 0 0 1 0 0

1 2 69 0 0 0 0 0 0 0 0 1 0 0

1 2 62 0 0 0 0 0 0 0 0 1 0 0

1 2 76 0 0 0 0 0 0 0 0 1 2 0

1 2 65 0 0 0 0 0 0 0 0 1 0 0

1 2 65 0 0 0 0 0 0 0 0 1 0 0

1 2 75 0 0 0 0 0 0 0 0 1 0 0

1 2 75 0 0 0 0 0 0 0 0 1 0 0

1 2 78 0 0 0 0 0 0 0 0 1 0 0

1 2 59 0 0 0 0 0 0 0 0 1 0 0

1 2 80 0 0 0 0 0 0 0 0 1 0 0

1 2 82 0 0 0 0 0 0 0 0 1 0 0

1 2 75 0 0 0 0 0 0 0 0 1 0 0

1 2 85 0 0 0 0 0 0 0 0 1 0 0

1 2 73 0 0 0 0 0 0 0 0 1 0 0

1 2 87 0 0 0 0 0 0 0 0 1 0 0

1 2 69 0 0 0 0 0 0 0 0 1 0 0

1 2 58 0 0 0 0 0 0 0 0 1 0 0

1 2 72 0 0 0 0 0 0 0 0 1 0 0

1 2 30 0 0 0 0 0 0 0 0 1 0 0

1 2 33 0 0 0 0 0 0 0 0 1 0 0

1 2 49 0 0 0 0 0 0 0 0 1 0 0

1 2 84 0 0 0 0 0 0 0 0 1 0 0

1 2 57 0 0 0 0 0 0 0 0 1 0 0

1 2 61 0 0 0 0 0 0 0 0 1 0 0

1 2 60 0 0 0 1 0 0 0 0 1 0 0

1 2 46 0 0 0 1 0 0 0 0 1 0 0

1 2 82 0 0 0 0 0 0 0 0 1 0 0

1 2 78 0 0 0 0 0 0 0 0 1 0 0

1 2 99 0 0 0 0 0 0 0 0 1 0 0

1 2 46 0 0 0 0 0 0 0 0 1 0 0

1 2 71 0 0 0 0 0 0 0 0 1 0 0

1 2 66 0 0 0 0 0 0 0 0 1 0 0

1 2 71 0 0 0 0 0 0 0 0 1 0 0

1 2 77 0 0 0 0 0 0 0 0 1 0 0

1 2 85 0 0 0 0 0 0 0 0 1 0 0

1 2 77 0 0 0 0 0 0 0 0 1 0 0

1 2 73 0 0 0 0 0 0 0 0 1 0 0

1 2 81 0 0 0 0 0 0 0 0 1 0 0

1 2 59 0 0 0 0 0 0 0 0 1 0 0

1 2 34 0 0 0 0 0 0 0 0 1 0 0

1 2 71 0 0 0 0 0 0 0 0 1 0 0

1 2 64 0 0 0 0 0 0 0 0 1 0 0

1 2 69 0 0 0 0 0 0 0 0 1 0 0

1 2 34 0 0 0 1 0 0 0 0 1 0 0

1 2 56 0 0 0 1 0 0 0 0 1 0 0

1 2 60 0 0 0 0 0 0 0 0 1 0 0

1 2 72 0 0 0 0 0 0 0 0 1 0 0

1 2 76 0 0 0 0 0 0 0 0 1 0 0

1 2 72 0 0 0 0 0 0 0 0 1 3 0

1 2 37 0 0 0 0 0 0 0 0 1 3 0

1 2 41 0 0 0 0 0 0 0 0 1 3 0

1 2 51 0 0 0 0 0 0 0 0 1 3 0

1 2 39 0 0 0 0 0 0 0 0 1 3 0

1 2 28 0 0 0 0 0 0 0 0 1 3 0

1 2 19 0 0 0 0 0 0 0 0 1 3 0

1 2 52 0 0 0 0 0 0 0 0 1 3 0

1 2 54 0 0 0 0 0 0 0 0 1 3 0

1 2 84 0 0 0 0 0 0 0 0 1 3 0

1 2 59 0 0 0 0 0 0 0 0 1 3 0

1 2 54 0 0 0 0 0 0 0 0 1 3 0

1 2 83 0 0 0 0 0 0 0 0 1 3 0

1 2 83 0 0 0 0 0 0 0 0 1 3 0

1 2 84 0 0 0 0 0 0 0 0 1 3 0

1 2 47 0 0 0 0 0 0 0 0 1 3 0

1 2 12 0 0 0 0 0 0 0 0 1 3 0

1 2 27 0 0 0 0 0 0 0 0 1 3 0

1 2 54 0 0 0 0 0 0 0 0 1 3 0

1 2 93 0 0 0 0 0 0 0 0 1 3 0

1 2 65 0 0 0 0 0 0 0 0 1 3 0

1 2 79 0 0 0 0 0 0 0 0 1 3 0

0 1 64 0 1 0 0 0 0 0 0 1 3 0

0 1 63 0 1 0 0 0 0 0 0 1 3 0

0 1 78 0 1 0 0 0 0 0 0 1 3 0

0 1 52 0 1 0 0 0 0 0 0 1 3 0

0 1 81 0 1 0 0 0 0 0 0 1 3 0

0 1 72 0 1 0 0 0 0 0 0 1 3 0

0 2 83 0 1 0 0 0 0 0 0 1 3 0

0 2 51 0 1 0 0 0 0 0 0 1 3 0

0 2 54 0 1 0 0 0 0 0 0 1 3 0

0 2 50 0 0 0 0 1 0 0 0 1 3 0

0 1 65 0 0 0 0 0 0 0 0 1 3 0

0 1 75 0 0 1 0 0 0 0 0 1 3 0

0 2 57 0 0 1 0 0 0 0 0 1 3 0

0 2 70 0 0 1 0 0 0 0 0 1 3 0

0 1 59 0 0 0 0 0 0 0 0 1 3 0

0 1 64 0 0 0 0 0 0 0 0 1 3 0

0 1 75 0 0 0 0 0 0 0 0 1 3 0

0 1 83 0 0 0 0 0 0 0 0 1 3 0

0 1 80 0 0 0 0 0 0 0 0 1 3 0

0 1 29 0 0 0 0 0 0 0 0 1 0 0

0 1 69 0 0 0 0 0 0 0 0 1 0 0

0 1 69 0 0 0 0 0 0 0 0 1 0 0

0 1 44 0 0 0 0 0 0 0 0 1 0 0

0 1 76 0 0 0 0 0 0 0 0 1 0 0

0 1 81 0 0 0 0 0 0 0 0 1 0 0

0 1 85 0 0 0 0 0 0 0 0 1 0 0

0 1 82 0 0 0 0 0 0 0 0 1 0 0

0 1 82 0 0 0 0 0 0 0 0 1 0 0

0 1 76 0 0 0 0 0 0 0 0 1 0 0

0 1 56 0 0 0 0 0 0 0 0 1 0 0

0 1 77 0 0 0 0 0 0 0 0 1 0 0

0 1 50 0 0 0 0 0 0 0 0 1 0 0

0 1 66 0 0 0 0 0 0 0 0 1 0 0

0 1 80 0 0 0 0 0 0 0 0 1 0 0

0 1 31 0 0 0 0 0 0 0 0 1 0 0

0 1 61 0 0 0 0 0 0 0 0 1 0 0

0 1 78 0 0 0 0 0 0 0 0 1 0 0

0 1 75 0 0 0 0 0 0 0 0 1 0 0

0 1 79 0 0 0 0 0 0 0 0 1 0 0

0 1 73 0 0 0 0 0 0 0 0 1 0 0

0 1 84 0 0 0 0 0 0 0 0 1 0 0

0 1 68 0 0 0 0 0 0 0 0 1 0 0

0 1 67 0 0 0 0 0 0 0 0 1 0 0

0 1 67 0 0 0 0 0 0 0 0 1 0 0

0 1 83 0 0 0 0 0 0 0 0 1 0 0

0 1 18 0 0 0 0 0 0 0 0 1 0 0

0 1 54 0 0 0 0 0 0 0 0 1 0 0

0 1 59 0 0 0 0 0 0 0 0 1 0 0

0 1 76 0 0 0 0 0 0 0 0 1 0 0

0 1 24 0 0 0 0 0 0 0 0 1 0 0

0 1 81 0 0 0 0 0 0 0 0 1 0 0

0 1 87 0 0 0 0 0 0 0 0 1 0 0

0 1 57 0 0 0 0 0 0 0 0 1 0 0

0 1 79 0 0 0 0 0 0 0 0 1 0 0

0 1 71 0 0 0 0 0 0 0 0 1 0 0

0 2 81 0 0 0 0 0 0 0 0 1 0 0

0 2 64 0 0 0 0 0 0 0 0 1 0 0

0 2 73 0 0 0 0 0 0 0 0 1 0 0

0 2 76 0 0 0 0 0 0 0 0 1 0 0

0 2 58 0 0 0 0 0 0 0 0 1 0 0

0 2 73 0 0 0 0 0 0 0 0 1 0 0

0 2 79 0 0 0 0 0 0 0 0 1 0 0

0 2 55 0 0 0 0 0 0 0 0 1 0 0

0 2 57 0 0 0 0 0 0 0 0 1 0 0

0 2 62 0 0 0 0 0 0 0 0 1 0 0

0 2 91 0 0 0 0 0 0 0 0 1 0 0

0 2 64 0 0 0 0 0 0 0 0 1 0 0

0 2 65 0 0 0 0 0 0 0 0 1 0 0

0 2 65 0 0 0 0 0 0 0 0 1 0 0

0 2 59 0 0 0 0 0 0 0 0 1 0 0

0 2 52 0 0 0 0 0 0 0 0 1 0 0

0 2 54 0 0 0 0 0 0 0 0 1 0 0

0 2 74 0 0 0 0 0 0 0 0 1 0 0

0 2 80 0 0 0 0 0 0 0 0 1 0 0

0 2 75 0 0 0 0 0 0 0 0 1 0 0

0 2 67 0 0 0 0 0 0 0 0 1 0 0

0 2 31 0 0 0 0 0 0 0 0 1 0 0

0 2 79 0 0 0 0 0 0 0 0 1 0 0

0 2 74 0 0 0 0 0 0 0 0 1 0 0

0 2 58 0 0 0 0 0 0 0 0 1 0 0

0 2 77 0 0 0 0 0 0 0 0 1 0 0

0 2 47 0 0 0 0 0 0 0 0 1 0 0

0 2 56 0 0 0 0 0 0 0 0 1 0 0

0 2 81 0 0 0 0 0 0 0 0 1 0 0

0 2 72 0 0 0 0 0 0 0 0 1 0 0

0 2 85 0 0 0 0 0 0 0 0 1 0 0

0 2 70 0 0 0 0 0 0 0 0 1 0 0

0 2 71 0 0 0 0 0 0 0 0 1 0 0

0 2 43 0 0 0 0 0 0 0 0 1 0 0

0 2 80 0 0 0 0 0 0 0 0 1 0 0

0 2 51 0 0 0 0 0 0 0 0 1 0 0

0 2 47 0 0 0 0 0 0 0 0 1 0 0

0 2 62 0 0 0 0 0 0 0 0 1 0 0

0 2 84 0 0 0 0 0 0 0 0 1 0 0

0 2 48 0 0 0 0 0 0 0 0 1 0 0

0 2 81 0 0 0 0 0 0 0 0 1 0 0

0 2 60 0 0 0 0 0 0 0 0 1 0 0

0 2 40 0 0 0 0 0 0 0 0 1 0 0

0 2 55 0 0 0 0 0 0 0 0 1 0 0

0 2 79 0 0 0 0 0 0 0 0 1 0 0

0 2 65 0 0 0 0 0 0 0 0 1 0 0

0 1 74 0 1 0 0 0 0 1 0 1 0 0

0 1 61 0 1 0 0 0 0 1 0 1 0 0

0 2 78 0 1 0 0 0 0 1 0 1 0 0

0 2 47 0 1 0 0 0 0 1 0 1 0 0

0 2 57 0 1 0 0 0 0 1 0 1 0 0

0 1 71 0 1 1 0 0 0 1 0 1 0 0

0 1 71 0 1 1 0 0 0 1 0 1 0 0

0 1 71 0 1 1 0 0 0 1 0 1 0 0

0 2 64 0 1 1 0 0 0 1 0 1 0 0

0 2 74 0 1 0 0 0 0 1 0 1 0 0

0 1 50 0 1 0 0 0 0 1 0 1 0 0

0 1 48 0 1 0 0 0 0 1 0 1 0 0

0 1 68 0 1 0 0 0 0 1 0 1 0 0

0 1 61 0 1 0 0 0 0 1 0 1 0 0

0 1 54 0 1 0 0 0 0 1 0 1 0 0

0 1 46 0 1 0 0 0 0 1 0 1 0 0

0 1 66 0 1 0 0 0 0 1 0 1 0 0

0 1 59 0 1 0 0 0 0 1 0 1 0 0

0 1 59 0 1 0 0 0 0 1 0 1 0 0

0 1 72 0 1 0 0 0 0 1 0 1 0 0

0 1 80 0 1 0 0 0 0 1 0 1 0 0

0 1 76 0 1 0 0 0 0 1 0 1 0 0

0 1 56 0 1 0 0 0 0 1 0 1 0 0

0 1 66 0 1 0 0 0 0 1 0 1 0 0

0 1 67 0 1 0 0 0 0 1 0 1 0 0

0 1 61 0 1 0 0 0 0 1 0 1 0 0

0 1 46 0 1 0 0 0 0 1 0 1 0 0

0 1 57 0 1 0 0 0 0 1 0 1 0 0

0 1 58 0 1 0 0 0 0 1 0 1 0 0

0 1 66 0 1 0 0 0 0 1 0 1 0 0

0 1 77 0 1 0 0 0 0 1 0 1 0 0

0 1 75 0 1 0 0 0 0 1 0 1 0 0

0 1 58 0 1 0 0 0 0 1 0 1 0 0

0 1 65 0 1 0 0 0 0 1 0 1 0 0

0 2 61 0 1 0 0 0 0 1 0 1 0 0

0 2 56 0 1 0 0 0 0 1 0 1 0 0

0 2 58 0 1 0 0 0 0 1 0 1 0 0

0 2 56 0 1 0 0 0 0 1 0 1 0 0

0 2 57 0 1 0 0 0 0 1 0 1 0 0

0 2 57 0 1 0 0 0 0 1 0 1 0 0

0 2 58 0 1 0 0 0 0 1 0 1 0 0

0 2 64 0 1 0 0 0 0 1 0 1 0 0

0 2 65 0 1 0 0 0 0 1 0 1 0 0

0 2 63 0 1 0 0 0 0 1 0 1 0 0

0 2 67 0 1 0 0 0 0 1 0 1 0 0

0 2 60 0 1 0 0 0 0 1 0 1 0 0

0 2 60 0 1 0 0 0 0 1 0 1 0 0

0 2 71 0 1 0 0 0 0 1 0 1 0 0

0 2 63 0 1 0 0 0 0 1 0 1 0 0

0 2 72 0 1 0 0 0 0 1 0 1 0 0

0 2 67 0 1 0 0 0 0 1 0 1 0 0

0 2 61 0 1 0 0 0 0 1 0 1 0 0

0 2 61 0 1 0 0 0 0 1 0 1 0 0

0 2 63 0 1 0 0 0 0 1 0 1 0 0

0 2 85 0 1 0 0 0 0 1 0 1 0 0

0 2 70 0 1 0 0 0 0 1 0 1 0 0

0 2 93 0 1 0 0 0 0 1 0 1 0 0

0 2 65 0 1 0 0 0 0 1 0 1 0 0

0 2 62 0 1 0 0 0 0 1 0 1 0 0

0 2 67 0 1 0 0 0 0 1 0 1 0 0

0 2 66 0 1 0 0 0 0 1 0 1 0 0

0 2 66 0 1 0 0 0 0 1 0 1 0 0

0 2 54 0 1 0 0 0 0 1 0 1 0 0

0 2 74 0 1 0 0 0 0 1 0 1 0 0

0 2 58 0 1 0 0 0 0 1 0 1 0 0

0 2 48 0 1 0 0 0 0 1 0 1 0 0

0 2 73 0 1 0 0 0 0 1 0 0 0 0

0 2 85 0 1 0 0 0 0 1 0 0 2 0

0 2 63 0 1 0 0 0 0 1 0 0 2 0

0 2 51 0 1 0 1 0 0 1 0 0 0 0

0 2 62 0 1 0 0 0 0 1 0 0 0 0

0 2 52 0 1 0 0 0 0 1 0 0 0 0

0 2 53 0 1 0 0 0 0 1 0 0 0 0

0 2 49 0 1 0 0 0 0 1 0 0 0 0

0 2 68 0 1 0 0 0 0 1 0 0 0 0

0 1 78 0 1 0 0 0 0 1 0 0 0 0

0 1 61 0 1 0 0 0 0 1 0 0 0 0

0 1 68 0 1 0 0 0 0 1 0 0 0 0

0 1 80 0 1 0 0 0 0 1 0 0 0 0

0 1 69 0 1 0 0 0 0 1 0 0 0 0

0 1 62 0 1 0 0 0 0 1 0 0 0 0

0 1 68 0 1 0 0 0 0 1 0 0 0 0

0 1 66 0 1 0 0 0 0 1 0 0 0 0

0 1 69 0 1 0 0 0 0 1 0 0 0 0

0 1 46 0 1 0 0 0 0 0 0 0 0 0

0 1 84 0 1 0 0 0 0 0 0 0 0 0

0 1 81 0 1 0 0 0 0 0 0 0 0 0

0 1 78 0 1 0 0 0 0 0 0 0 0 0

0 1 72 0 1 0 0 0 0 0 0 0 0 0

0 1 68 0 1 0 0 0 0 0 0 0 0 0

0 1 56 0 1 0 0 0 0 0 0 0 0 0

0 1 57 0 1 0 0 0 0 0 0 0 0 0

0 1 70 0 1 0 0 0 0 0 0 0 0 0

0 1 63 0 1 0 0 0 0 0 0 0 0 0

0 1 71 0 1 0 0 0 0 0 0 0 0 0

0 1 47 0 1 0 1 0 0 0 0 0 0 0

0 1 62 0 1 0 0 0 0 0 0 0 0 0

0 1 63 0 1 0 0 0 0 0 0 0 3 0

0 1 70 0 1 0 0 0 0 0 0 0 3 0

0 1 68 0 1 0 0 0 0 0 0 0 3 0

0 1 49 0 1 0 0 0 0 0 0 0 3 0

0 1 53 0 1 0 0 0 0 0 0 0 0 0

0 1 71 0 1 0 0 0 0 0 0 0 0 0

0 1 54 0 1 0 0 0 0 0 0 0 2 0

0 1 65 0 1 0 0 0 0 0 1 0 0 0

0 1 65 0 1 0 0 0 0 0 1 0 0 0

0 1 78 0 1 0 0 0 0 0 1 0 4 0

0 1 73 0 1 0 0 0 0 0 1 0 0 0

0 1 75 0 1 0 0 0 0 0 1 0 3 0

0 1 54 0 1 0 0 0 0 0 1 0 0 0

0 1 54 0 1 0 0 0 0 0 1 0 0 0

0 1 79 0 1 0 0 0 0 0 1 0 0 0

0 1 83 0 1 0 0 0 0 0 1 0 4 0

0 1 72 0 1 0 0 0 0 0 1 0 0 0

0 1 53 0 1 0 0 0 0 0 1 0 0 0

0 1 71 0 1 0 0 0 0 0 1 0 0 0

0 1 71 0 1 0 0 0 0 0 1 0 0 0

0 1 65 0 1 0 0 0 0 0 1 0 0 0

0 1 65 0 1 0 0 0 0 0 1 0 0 0

0 1 74 0 1 0 0 0 0 0 1 0 0 0

0 1 73 0 1 0 0 0 0 0 1 0 0 0

0 1 75 0 1 0 0 0 0 0 1 0 0 0

0 1 60 0 1 0 0 0 0 0 1 0 0 0

0 1 76 0 1 0 0 0 0 0 1 0 0 0

0 1 74 0 1 0 0 0 0 0 1 0 0 0

0 1 73 0 1 0 0 0 0 0 1 0 0 0

0 1 73 0 1 0 0 0 0 0 1 0 0 0

0 1 81 0 1 0 0 0 0 0 1 0 2 0

0 1 81 0 1 0 0 0 0 0 1 0 2 0

0 1 61 0 1 0 0 0 0 0 1 0 2 0

0 1 67 0 1 0 0 0 0 0 1 0 2 0

0 1 71 0 1 0 0 0 0 0 1 0 2 0

0 1 50 0 1 0 0 0 0 0 1 0 3 0

0 1 75 0 1 0 0 0 0 0 1 0 3 0

0 1 71 0 1 0 0 0 0 0 1 0 3 0

0 1 76 0 1 0 0 0 0 0 1 0 3 0

0 1 58 0 1 0 0 0 0 0 1 0 3 0

0 1 79 0 1 0 0 0 0 0 1 0 3 0

0 1 76 0 1 0 0 0 0 0 1 0 3 0

0 1 76 0 1 0 0 0 0 0 1 0 4 0

0 1 83 0 1 0 0 0 0 0 1 0 4 0

0 1 79 0 1 0 0 0 0 0 1 0 4 0

0 1 72 0 1 0 0 0 0 0 1 0 4 0

0 1 72 0 1 0 0 0 0 0 1 0 0 0

0 1 70 0 1 0 0 0 0 0 1 0 4 0

0 1 63 0 1 0 0 0 0 0 1 0 1 0

0 1 75 0 1 0 0 0 0 0 1 0 2 0

0 1 34 0 1 0 0 0 0 0 1 0 3 0

0 1 48 0 1 0 0 0 0 0 1 0 3 0

0 1 57 0 1 0 0 0 0 0 1 0 3 0

0 1 58 0 1 0 0 0 0 0 1 0 3 0

0 1 59 0 1 0 0 0 0 0 1 0 4 0

0 1 36 0 1 0 0 0 0 0 1 0 4 0

0 1 57 0 1 0 0 0 0 0 1 0 0 0

0 1 72 0 1 0 0 0 0 0 1 0 0 0

0 1 67 0 1 0 0 0 0 0 1 0 0 0

0 1 73 0 1 0 0 0 0 0 1 0 0 0

0 1 65 0 1 0 0 0 0 0 1 0 0 0

0 1 66 0 1 0 0 0 0 0 1 0 0 0

0 1 63 0 1 0 0 0 0 0 1 0 0 0

0 1 62 0 1 0 0 0 0 0 1 0 3 0

0 1 72 0 1 0 0 0 0 0 1 0 3 0

0 1 70 0 1 0 0 0 0 0 1 0 0 0

0 1 46 0 1 0 0 0 0 0 1 0 0 0

0 1 78 0 1 0 0 0 0 0 1 0 0 0

0 1 75 0 1 0 0 0 0 0 0 0 0 0

0 1 83 0 1 0 0 0 0 0 0 0 0 0

0 1 66 0 1 0 0 0 0 0 0 0 0 0

0 1 81 0 1 0 0 0 0 0 0 0 0 0

0 1 75 0 1 0 0 0 0 0 0 0 0 0

0 1 43 0 1 0 0 0 0 0 0 0 0 0

0 1 38 0 1 0 0 0 0 0 0 0 0 0

0 1 67 0 1 0 0 0 0 0 0 0 0 0

0 1 63 0 1 0 0 0 0 0 0 0 0 0

0 1 62 0 1 0 0 0 0 0 0 0 0 0

0 1 50 0 1 0 0 0 0 0 0 0 0 0

0 1 76 0 1 0 0 0 0 0 0 0 0 0

0 1 66 0 1 0 0 0 0 0 0 0 0 0

0 1 75 0 1 0 0 0 0 0 0 0 0 0

0 1 44 0 1 0 0 0 0 0 0 0 0 0

0 1 78 0 1 0 0 0 0 0 0 0 0 0

0 1 78 0 1 0 0 0 0 0 0 0 0 0

0 1 78 0 1 0 0 0 0 0 0 0 0 0

0 1 83 0 1 0 0 0 0 0 0 0 0 0

0 1 66 0 1 0 0 0 0 0 0 0 0 0

0 1 70 0 1 0 0 0 0 0 0 0 0 0

0 1 58 0 1 0 0 0 0 0 0 0 4 0

0 1 84 0 1 0 0 0 0 0 0 0 2 0

0 1 74 0 1 0 0 0 0 0 0 0 4 0

0 1 63 0 1 0 0 0 0 0 0 0 4 0

0 1 77 0 1 0 0 0 0 0 0 0 0 0

0 1 76 0 1 0 0 0 0 0 0 0 0 0

0 1 35 0 1 0 0 0 0 0 0 0 0 0

0 1 56 0 1 0 0 0 0 0 0 0 3 0

0 1 67 0 1 0 0 0 0 0 0 0 3 0

0 1 74 0 1 0 0 0 0 0 0 0 0 0

0 1 74 0 1 0 0 0 0 0 0 0 3 0

0 1 69 0 1 0 0 0 0 0 0 0 2 0

0 1 72 0 1 0 0 0 0 0 0 0 0 0

0 1 63 0 1 0 0 0 0 0 0 0 0 0

0 1 82 0 1 0 0 0 0 0 0 0 0 0

0 1 50 0 1 0 0 0 0 0 0 0 0 0

0 1 70 0 1 0 0 0 0 0 0 0 0 0

0 1 74 0 1 0 0 0 0 0 0 0 0 0

0 1 75 0 1 0 0 0 0 0 0 0 0 0

0 1 74 0 1 0 0 0 0 0 0 0 0 0

0 1 66 0 1 0 0 0 0 0 0 0 0 0

0 1 60 0 1 0 0 0 0 0 0 0 0 0

0 1 58 0 1 0 0 0 0 0 0 0 0 0

0 1 58 0 1 0 0 0 0 0 0 0 0 0

0 1 60 0 1 0 0 0 0 0 0 0 0 0

0 1 73 0 1 0 0 0 0 0 0 0 0 0

0 2 80 0 1 0 0 0 0 0 0 0 0 0

0 2 72 0 1 0 0 0 0 0 0 0 0 0

0 2 72 0 1 0 0 0 0 0 0 0 0 0

0 2 77 0 1 0 0 0 0 0 0 0 0 0

0 2 71 0 1 0 0 0 0 0 0 0 0 0

0 2 61 0 1 0 0 0 0 0 0 0 0 0

0 2 60 0 1 0 0 0 0 0 0 0 4 0

0 2 76 0 1 0 0 0 0 0 0 0 0 0

0 2 48 0 1 0 0 0 0 0 0 0 0 0

0 2 80 0 1 0 0 0 0 0 0 0 4 0

0 2 71 0 1 0 0 0 0 0 0 0 0 0

0 2 55 0 1 0 0 0 0 0 0 0 0 0

0 2 61 0 1 0 0 0 0 0 0 0 0 0

0 2 72 0 1 0 0 0 0 0 0 0 0 0

0 2 75 0 1 0 1 0 0 0 0 0 0 0

0 2 57 0 1 0 1 0 0 0 0 0 2 0

0 2 60 0 1 0 1 0 0 0 0 0 2 0

0 2 61 0 1 0 1 0 0 0 0 0 2 0

0 2 77 0 1 0 0 0 0 0 0 0 3 0

0 2 58 0 1 0 0 0 0 0 0 0 3 0

0 2 62 0 1 0 0 0 0 0 0 0 3 0

0 2 56 0 1 0 0 0 0 0 0 0 0 0

0 2 58 0 1 0 0 0 0 0 0 0 0 0

0 2 46 0 1 0 0 0 0 0 0 0 0 0

0 2 55 0 1 0 0 0 0 0 0 0 0 0

0 2 48 0 1 0 0 0 0 0 0 0 0 0

0 2 53 0 1 0 0 0 0 0 0 0 2 0

0 2 64 0 1 0 0 0 0 0 0 0 2 0

0 2 40 0 1 0 0 0 0 0 0 0 2 0

0 2 47 0 1 0 0 0 0 0 0 0 2 0

0 2 58 0 1 0 0 0 0 0 0 0 3 0

0 2 56 0 1 0 0 0 0 0 0 0 3 0

0 2 62 0 1 0 0 0 0 0 0 0 4 0

0 2 61 0 1 0 0 0 0 0 0 0 4 0

0 2 51 0 1 0 0 0 0 0 0 0 0 0

0 2 41 0 1 0 0 0 0 0 0 0 0 0

0 2 63 0 1 0 0 0 0 0 0 0 0 0

0 2 77 0 1 0 0 0 0 0 0 0 0 0

0 2 57 0 1 0 0 0 0 0 0 0 3 0

0 2 68 0 1 0 0 0 0 0 0 0 3 0

0 2 61 0 1 0 0 0 0 0 0 0 4 0

0 2 79 0 1 0 0 0 0 0 0 0 4 0

0 2 59 0 1 0 0 0 0 0 0 0 0 0

0 2 60 0 1 0 0 0 0 0 0 0 0 0

0 2 45 0 1 0 0 0 0 0 0 0 4 0

0 2 64 0 1 0 0 0 0 0 0 0 3 0

0 2 66 0 1 0 0 0 0 0 0 0 3 0

0 2 57 0 1 0 0 0 0 0 0 0 3 0

0 2 71 0 1 0 0 0 0 0 0 0 0 0

0 2 63 0 1 0 0 0 0 0 0 0 0 0

0 2 57 0 1 0 0 0 0 0 0 0 0 0

0 2 73 0 1 0 0 0 0 0 0 0 0 0

0 2 60 0 1 0 0 0 0 0 0 0 3 0

0 2 55 0 1 0 0 0 0 0 0 0 4 0

0 2 71 0 1 0 0 0 0 0 0 0 0 0

0 2 35 0 1 0 0 0 0 0 0 0 0 0

0 2 46 0 1 0 0 0 0 0 0 0 0 0

0 2 61 0 1 0 0 0 0 0 0 0 2 0

0 2 62 0 1 0 0 0 0 0 0 0 0 0

0 2 59 0 1 0 0 0 0 0 0 0 0 0

0 2 68 0 1 0 0 0 0 0 0 0 0 0

0 2 63 0 1 0 0 0 0 0 0 0 0 0

0 2 68 0 1 0 0 0 0 0 0 0 0 0

0 2 82 0 1 0 0 0 0 0 0 0 0 0

0 2 63 0 1 0 0 0 0 0 0 0 0 0

0 2 54 0 1 0 0 0 0 0 0 0 0 0

0 2 51 0 1 0 0 0 0 0 0 0 0 0

0 2 76 0 1 0 0 0 0 0 0 0 0 0

0 2 70 0 1 0 0 0 0 0 0 0 0 0

0 2 74 0 1 0 0 0 0 0 0 0 0 0

0 2 76 0 1 0 0 0 0 0 0 0 0 0

0 2 86 0 1 0 0 0 0 0 0 0 0 0

0 2 74 0 1 0 0 0 0 0 0 0 0 0

0 2 84 0 1 0 0 0 0 0 0 0 0 0

0 2 80 0 1 0 0 0 0 0 0 0 0 0

0 2 80 0 1 0 0 0 0 0 0 0 0 0

0 2 59 0 1 0 0 0 0 0 0 0 0 0

0 2 65 0 1 0 0 0 0 0 0 0 0 0

0 2 75 0 1 0 0 0 0 0 0 0 0 0

0 2 75 0 1 0 0 0 0 0 0 0 0 0

0 2 79 0 1 0 0 0 0 0 0 0 0 0

0 2 65 0 1 0 0 0 0 0 0 0 0 0

0 2 53 0 1 0 0 0 0 0 0 0 0 0

0 2 64 0 1 0 0 0 0 0 0 0 0 0

0 2 67 0 1 0 0 0 0 0 0 0 0 0

0 2 65 0 1 0 0 0 0 0 0 0 0 0

0 2 70 0 1 0 0 0 0 0 0 0 0 0

0 2 75 0 1 0 0 0 0 0 0 0 0 0

0 2 75 0 1 0 0 0 0 0 0 0 0 0

0 2 70 0 1 0 0 0 0 0 0 0 0 0

0 2 76 0 1 0 0 0 0 0 0 0 0 0

0 2 61 0 1 0 0 0 0 0 0 0 2 0

0 2 72 0 1 0 0 0 0 0 0 0 0 0

0 2 78 0 1 0 0 0 0 0 0 0 0 0

0 2 71 0 1 0 0 0 0 0 0 0 0 0

0 2 54 0 1 0 0 0 0 0 0 0 0 0

0 2 76 0 1 0 0 0 0 0 0 0 0 0

0 2 68 0 1 0 0 0 0 0 0 0 0 0

0 2 66 0 1 0 0 0 0 0 0 0 0 0

0 2 74 0 1 0 0 0 0 0 0 0 0 0

0 2 64 0 1 0 0 0 0 0 0 0 0 0

0 2 89 0 1 0 0 0 0 0 0 0 0 0

0 2 74 0 1 0 0 0 0 0 0 0 2 0

0 2 47 0 1 0 0 0 0 0 0 0 0 0

0 2 66 0 1 0 0 0 0 0 0 0 0 0

0 2 60 0 1 0 0 0 0 0 0 0 0 0

0 2 75 0 1 0 0 0 0 0 0 0 0 0

0 2 69 0 1 0 0 0 0 0 0 0 0 0

0 2 71 0 1 0 0 0 0 0 0 0 0 0

0 2 73 0 1 0 0 0 0 0 0 0 0 0

0 2 73 0 1 0 0 0 0 0 0 0 0 0

0 2 74 0 1 0 0 0 0 0 0 0 0 0

0 2 80 0 1 0 0 0 0 0 0 0 0 0

0 2 80 0 1 0 0 0 0 0 0 0 0 0

0 2 74 0 1 0 0 0 0 0 0 0 0 0

0 2 72 0 1 0 0 0 0 0 0 0 0 0

0 2 74 0 1 0 0 0 0 0 0 0 0 0

0 2 71 0 1 0 0 0 0 0 0 0 0 0

0 2 83 0 1 0 0 0 0 0 0 0 0 0

0 2 83 0 1 0 0 0 0 0 0 0 0 0

0 2 50 0 1 0 0 0 0 0 0 0 0 0

0 2 65 0 1 0 0 0 0 0 0 0 0 0

0 2 80 0 1 0 0 0 0 0 0 0 2 0

0 2 78 0 1 0 0 0 0 0 0 0 2 0

0 2 48 0 1 0 0 0 0 0 0 0 2 0

0 2 66 0 1 0 0 0 0 0 0 0 3 0

0 2 53 0 1 0 0 0 0 0 0 0 0 0

0 2 81 0 1 0 0 0 0 0 0 0 0 0

0 2 61 0 1 0 0 0 0 0 0 0 0 0

0 2 33 0 1 0 0 0 0 0 0 0 3 0

0 2 84 0 1 0 0 0 0 0 0 0 0 0

0 2 75 0 1 0 0 0 0 0 0 0 0 0

0 2 61 0 1 0 0 0 0 0 0 0 2 0

0 2 67 0 1 0 0 0 0 0 0 0 0 0

0 2 70 0 1 0 0 0 0 0 0 0 0 0

0 2 70 0 1 0 0 0 0 0 0 0 0 0

0 2 69 0 1 0 0 0 0 0 0 0 0 0

0 2 80 0 1 0 0 0 0 0 0 0 0 0

0 2 72 0 1 0 0 0 0 0 0 0 0 0

0 2 66 0 1 0 0 0 0 0 0 0 0 0

0 2 75 0 1 0 0 0 0 0 0 0 0 0

0 2 54 0 1 0 0 0 0 0 0 0 0 0

0 2 67 0 1 0 0 0 0 0 0 0 0 0

0 2 74 0 1 0 0 0 0 0 0 0 0 0

0 2 47 0 1 0 0 0 0 0 0 0 0 0

0 2 50 0 1 0 0 0 0 0 0 0 0 0

0 2 51 0 1 0 0 0 0 0 0 0 0 0

0 2 50 0 1 0 0 0 0 0 0 0 0 0

0 2 54 0 1 0 0 0 0 0 0 0 0 0

0 2 69 0 1 0 0 0 0 0 0 0 0 0

0 2 62 0 1 0 0 0 0 0 0 0 0 0

0 2 72 0 1 0 0 0 0 0 0 0 0 0

0 2 66 0 1 0 0 0 0 0 0 0 0 0

0 2 67 0 1 0 0 0 0 0 0 0 0 0

0 2 54 0 1 0 0 0 0 0 0 0 0 0

0 2 61 0 1 0 0 0 0 0 0 0 0 0

0 2 58 0 1 0 0 0 0 0 0 0 0 0

0 2 71 0 1 0 0 0 0 0 0 0 0 0

0 2 64 0 1 0 0 0 0 0 0 0 0 0

0 2 74 0 1 0 0 0 0 0 0 0 0 0

0 2 83 0 1 0 0 0 0 0 0 0 0 0

0 2 53 0 1 0 0 0 0 0 0 0 0 0

0 2 79 0 1 0 0 0 0 0 0 0 0 0

0 2 62 0 1 0 0 0 0 0 0 0 0 0

0 2 60 0 1 0 0 0 0 0 0 0 0 0

0 2 56 0 1 0 0 0 0 0 0 0 0 0

0 2 72 0 1 0 0 0 0 0 0 0 0 0

0 2 66 0 1 0 0 0 0 0 0 0 0 0

0 2 75 0 1 0 0 0 0 0 0 0 0 0

0 2 54 0 1 0 0 0 0 0 0 0 0 0

0 2 58 0 1 0 0 0 0 0 0 0 0 0

0 2 28 0 1 0 0 0 0 0 0 0 0 0

0 2 59 0 1 0 0 0 0 0 0 0 0 0

0 2 83 0 1 0 0 0 0 0 0 0 0 0

0 2 64 0 1 0 0 0 0 0 0 0 0 0

0 2 74 0 1 0 0 0 0 0 0 0 0 0

0 2 65 0 1 0 0 0 0 0 0 0 0 0

0 2 64 0 1 0 1 0 0 0 0 0 0 0

0 2 80 0 1 0 0 0 0 0 0 0 0 0

0 2 69 0 1 0 0 0 0 0 0 0 0 0

0 2 90 0 1 0 0 0 0 0 0 0 0 0

0 2 72 0 1 0 0 0 0 0 0 0 0 0

0 2 69 0 1 0 0 0 0 0 0 0 0 0

0 2 45 0 1 0 0 0 0 0 0 0 0 0

0 2 73 0 1 0 0 0 0 0 0 0 0 0

0 2 61 0 1 0 0 0 0 0 0 0 3 0

0 2 60 0 1 0 0 0 0 0 0 0 3 0

0 2 58 0 1 0 0 0 0 0 0 0 3 0

0 2 67 0 1 0 0 0 0 0 0 0 3 0

0 2 54 0 1 0 0 0 0 0 0 0 3 0

0 2 74 0 1 0 0 0 0 0 0 0 3 0

0 2 66 0 1 0 0 0 0 0 0 0 3 0

0 2 58 0 1 0 0 0 0 0 0 0 3 0

0 2 71 0 1 0 0 0 0 0 0 0 3 0

0 2 56 0 1 0 0 0 0 0 0 0 0 0

0 2 85 0 1 0 0 0 0 0 0 0 0 0

0 2 76 0 1 0 0 0 0 0 0 0 0 0

0 2 71 0 1 0 0 0 0 0 0 0 0 0

0 2 61 0 1 0 0 0 0 0 0 0 0 0

0 2 57 0 1 0 0 0 0 0 0 0 0 0

0 2 75 0 1 0 0 0 0 0 0 0 3 0

0 2 85 0 1 0 0 0 0 0 0 0 0 0

0 2 85 0 1 0 0 0 0 0 0 0 0 0

0 2 82 0 0 1 0 1 0 0 0 0 0 0

0 1 38 0 0 0 0 1 0 0 0 0 0 0

0 1 32 0 0 0 0 1 0 0 0 0 0 0

0 1 63 0 0 0 0 1 0 0 0 0 0 0

0 1 31 0 0 0 0 1 0 0 0 0 0 0

0 1 44 0 0 0 0 1 0 0 0 0 4 0

0 1 50 0 0 0 0 1 0 0 0 0 0 0

0 1 16 0 0 0 0 1 0 0 0 0 0 0

0 1 59 0 0 0 0 1 0 0 0 0 3 0

0 1 44 0 0 0 0 1 0 0 0 0 4 0

0 1 11 0 0 0 0 1 0 0 0 0 0 0

0 1 21 0 0 0 0 1 0 0 0 0 0 0

0 1 7 0 0 0 0 1 0 0 0 0 0 0

0 1 18 0 0 0 0 1 0 0 0 0 0 0

0 1 20 0 0 0 0 1 0 0 0 0 0 0

0 1 7 0 0 0 0 1 0 0 0 0 0 0

0 1 17 0 0 0 0 1 0 0 0 0 0 0

0 1 37 0 0 0 0 1 0 0 0 0 0 0

0 1 31 0 0 0 0 1 0 0 0 0 0 0

0 1 48 0 0 0 0 1 0 0 0 0 2 0

0 1 3 0 0 0 0 1 0 0 0 0 0 0

0 1 43 0 0 0 0 1 0 0 0 0 0 0

0 1 43 0 0 0 0 1 0 0 0 0 0 0

0 1 54 0 0 0 0 1 0 0 0 0 3 0

0 2 38 0 0 0 0 1 0 0 0 0 0 0

0 2 52 0 0 0 0 1 0 0 0 0 0 0

0 2 47 0 0 0 0 1 0 0 0 0 0 0

0 2 29 0 0 0 0 1 0 0 0 0 0 0

0 2 41 0 0 0 0 1 0 0 0 0 0 0

0 2 22 0 0 0 0 1 0 0 0 0 0 0

0 2 12 0 0 0 0 1 0 0 0 0 0 0

0 2 48 0 0 0 0 1 0 0 0 0 0 0

0 2 5 0 0 0 0 1 0 0 0 0 0 0

0 2 37 0 0 0 0 1 0 0 0 0 0 0

0 2 17 0 0 0 0 1 0 0 0 0 0 0

0 2 65 0 0 0 0 1 0 0 0 0 0 0

0 2 73 0 0 0 0 1 0 0 0 0 0 0

0 2 57 0 0 0 0 1 0 0 0 0 0 0

0 2 39 0 0 0 0 1 0 0 0 0 0 0

0 2 35 0 0 0 0 1 0 0 0 0 0 0

0 2 38 0 0 0 0 1 0 0 0 0 0 0

0 2 15 0 0 0 0 1 0 0 0 0 0 0

0 2 55 0 0 0 0 1 0 0 0 0 0 0

0 2 65 0 0 0 0 1 0 0 0 0 0 0

0 2 58 0 0 0 0 1 0 0 0 0 0 0

0 2 42 0 0 0 0 1 0 0 0 0 0 0

0 2 26 0 0 0 0 1 0 0 0 0 0 0

0 2 45 0 0 0 0 1 0 0 0 0 0 0

0 2 4 0 0 0 0 1 0 0 0 0 0 0

0 2 32 0 0 0 0 1 0 0 0 0 0 0

0 2 35 0 0 0 0 1 0 0 0 0 0 0

0 2 41 0 0 0 0 1 0 0 0 0 0 0

0 2 52 0 0 0 0 1 0 0 0 0 0 0

0 2 15 0 0 0 0 1 0 0 0 0 0 0

0 2 72 0 0 0 0 1 0 0 0 0 0 0

0 2 56 0 0 0 0 1 0 0 0 0 0 0

0 2 36 0 0 0 0 1 0 0 0 0 0 0

0 2 55 0 0 0 0 1 0 0 0 0 0 0

0 2 23 0 0 0 0 1 0 0 0 0 0 0

0 2 42 0 0 0 0 1 0 0 0 0 0 0

0 2 19 0 0 0 0 1 0 0 0 0 0 0

0 2 38 0 0 0 0 1 0 0 0 0 0 0

0 2 26 0 0 0 0 1 0 0 0 0 0 0

0 2 39 0 0 0 0 1 0 0 0 0 0 0

0 2 16 0 0 0 0 1 0 0 0 0 0 0

0 1 70 0 0 1 0 0 0 0 0 0 0 0

0 1 79 0 0 1 0 0 0 0 0 0 0 0

0 1 55 0 0 1 0 0 0 0 0 0 0 0

0 1 19 0 0 1 0 0 0 0 0 0 0 0

0 1 68 0 0 1 0 0 0 0 0 0 0 0

0 1 37 0 0 1 0 0 0 0 0 0 0 0

0 2 60 0 0 1 0 0 0 0 0 0 0 0

0 2 59 0 0 1 0 0 0 0 0 0 0 0

0 1 73 0 0 0 0 0 0 0 0 0 0 0

0 1 60 0 0 0 0 0 0 0 0 0 0 0

0 1 38 0 0 0 0 0 0 0 0 0 0 0

0 1 24 0 0 0 0 0 0 0 0 0 0 0

0 1 38 0 0 0 0 0 0 0 0 0 0 0

0 1 45 0 0 0 0 0 0 0 0 0 0 0

0 1 48 0 0 0 0 0 0 0 0 0 0 0

0 1 64 0 0 0 0 0 0 0 0 0 0 0

0 1 36 0 0 0 0 0 0 0 0 0 0 0

0 1 27 0 0 0 0 0 0 0 0 0 0 0

0 1 53 0 0 0 1 0 0 0 0 0 0 0

0 1 28 0 0 0 0 0 0 0 0 0 0 0

0 1 59 0 0 0 0 0 0 0 0 0 0 0

0 1 64 0 0 0 0 0 0 0 0 0 0 0

0 1 63 0 0 0 0 0 0 0 0 0 0 0

0 1 63 0 0 0 0 0 0 0 0 0 0 0

0 1 49 0 0 0 0 0 0 0 0 0 0 0

0 1 65 0 0 0 0 0 0 0 0 0 0 0

0 1 65 0 0 0 0 0 0 0 0 0 0 0

0 1 73 0 0 0 0 0 0 0 0 0 0 0

0 1 27 0 0 0 0 0 0 0 0 0 0 0

0 1 60 0 0 0 0 0 0 0 0 0 0 0

0 1 82 0 0 0 0 0 0 0 0 0 0 0

0 1 48 0 0 0 0 0 0 0 0 0 0 0

0 1 66 0 0 0 0 0 0 0 0 0 0 0

0 1 61 0 0 0 0 0 0 0 0 0 0 0

0 1 83 0 0 0 0 0 0 0 0 0 0 0

0 1 29 0 0 0 0 0 0 0 0 0 0 0

0 1 48 0 0 0 0 0 0 0 0 0 0 0

0 1 55 0 0 0 0 0 0 0 0 0 0 0

0 1 47 0 0 0 0 0 0 0 0 0 0 0

0 1 24 0 0 0 0 0 0 0 0 0 0 0

0 1 52 0 0 0 0 0 0 0 0 0 0 0

0 1 81 0 0 0 0 0 0 0 0 0 1 0

0 1 60 0 0 0 0 0 0 0 0 0 1 0

0 1 67 0 0 0 0 0 0 0 0 0 1 0

0 1 67 0 0 0 0 0 0 0 0 0 1 0

0 1 66 0 0 0 0 0 0 0 0 0 1 0

0 1 51 0 0 0 0 0 0 0 0 0 1 0

0 1 61 0 0 0 0 0 0 0 0 0 1 0

0 1 72 0 0 0 0 0 0 0 0 0 1 0

0 1 66 0 0 0 0 0 0 0 0 0 1 0

0 1 61 0 0 0 0 0 0 0 0 0 1 0

0 1 61 0 0 0 0 0 0 0 0 0 2 0

0 1 49 0 0 0 0 0 0 0 0 0 2 0

0 1 53 0 0 0 0 0 0 0 0 0 2 0

0 1 37 0 0 0 0 0 0 0 0 0 2 0

0 1 65 0 0 0 0 0 0 0 0 0 2 0

0 1 63 0 0 0 0 0 0 0 0 0 2 0

0 1 48 0 0 0 0 0 0 0 0 0 2 0

0 1 42 0 0 0 0 0 0 0 0 0 2 0

0 1 53 0 0 0 0 0 0 0 0 0 2 0

0 1 76 0 0 0 0 0 0 0 0 0 2 0

0 1 40 0 0 0 0 0 0 0 0 0 2 0

0 1 63 0 0 0 0 0 0 0 0 0 2 0

0 1 59 0 0 0 0 0 0 0 0 0 2 0

0 1 59 0 0 0 0 0 0 0 0 0 2 0

0 1 54 0 0 0 0 0 0 0 0 0 3 0

0 1 53 0 0 0 0 0 0 0 0 0 3 0

0 1 73 0 0 0 0 0 0 0 0 0 3 0

0 1 50 0 0 0 0 0 0 0 0 0 3 0

0 1 76 0 0 0 0 0 0 0 0 0 3 0

0 1 63 0 0 0 0 0 0 0 0 0 4 0

0 1 72 0 0 0 0 0 0 0 0 0 4 0

0 1 61 0 0 0 0 0 0 0 0 0 0 0

0 1 72 0 0 0 0 0 0 0 0 0 1 0

0 1 72 0 0 0 0 0 0 0 0 0 1 0

0 1 53 0 0 0 0 0 0 0 0 0 1 0

0 1 73 0 0 0 0 0 0 0 0 0 1 0

0 1 73 0 0 0 0 0 0 0 0 0 2 0

0 1 27 0 0 0 0 0 0 0 0 0 2 0

0 1 48 0 0 0 0 0 0 0 0 0 2 0

0 1 60 0 0 0 0 0 0 0 0 0 2 0

0 1 43 0 0 0 0 0 0 0 0 0 2 0

0 1 51 0 0 0 0 0 0 0 0 0 2 0

0 1 72 0 0 0 0 0 0 0 0 0 2 0

0 1 72 0 0 0 0 0 0 0 0 0 2 0

0 1 49 0 0 0 0 0 0 0 0 0 2 0

0 1 50 0 0 0 0 0 0 0 0 0 2 0

0 1 60 0 0 0 0 0 0 0 0 0 2 0

0 1 74 0 0 0 0 0 0 0 0 0 2 0

0 1 34 0 0 0 0 0 0 0 0 0 2 0

0 1 56 0 0 0 0 0 0 0 0 0 2 0

0 1 58 0 0 0 0 0 0 0 0 0 2 0

0 1 37 0 0 0 0 0 0 0 0 0 2 0

0 1 38 0 0 0 0 0 0 0 0 0 2 0

0 1 43 0 0 0 0 0 0 0 0 0 2 0

0 1 86 0 0 0 0 0 0 0 0 0 2 0

0 1 86 0 0 0 0 0 0 0 0 0 2 0

0 1 64 0 0 0 0 0 0 0 0 0 2 0

0 1 31 0 0 0 0 0 0 0 0 0 3 0

0 1 86 0 0 0 0 0 0 0 0 0 3 0

0 1 19 0 0 0 0 0 0 0 0 0 3 0

0 1 54 0 0 0 0 0 0 0 0 0 3 0

0 1 50 0 0 0 0 0 0 0 0 0 3 0

0 1 17 0 0 0 0 0 0 0 0 0 3 0

0 1 17 0 0 0 0 0 0 0 0 0 3 0

0 1 60 0 0 0 0 0 0 0 0 0 3 0

0 1 59 0 0 0 0 0 0 0 0 0 3 0

0 1 60 0 0 0 0 0 0 0 0 0 3 0

0 1 37 0 0 0 0 0 0 0 0 0 3 0

0 1 49 0 0 0 0 0 0 0 0 0 3 0

0 1 53 0 0 0 0 0 0 0 0 0 3 0

0 1 72 0 0 0 0 0 0 0 0 0 3 0

0 1 29 0 0 0 0 0 0 0 0 0 3 0

0 1 65 0 0 0 0 0 0 0 0 0 3 0

0 1 63 0 0 0 0 0 0 0 0 0 3 0

0 1 73 0 0 0 0 0 0 0 0 0 3 0

0 1 20 0 0 0 0 0 0 0 0 0 4 0

0 1 68 0 0 0 0 0 0 0 0 0 0 0

0 1 79 0 0 0 0 0 0 0 0 0 0 0

0 1 63 0 0 0 0 0 0 0 0 0 0 0

0 1 63 0 0 0 0 0 0 0 0 0 0 0

0 1 83 0 0 0 0 0 0 0 0 0 0 0

0 1 64 0 0 0 0 0 0 0 0 0 0 0

0 2 60 0 0 0 0 0 0 0 0 0 0 0

0 2 70 0 0 0 0 0 0 0 0 0 0 0

0 2 70 0 0 0 0 0 0 0 0 0 0 0

0 2 60 0 0 0 0 0 0 0 0 0 0 0

0 2 75 0 0 0 0 0 0 0 0 0 0 0

0 2 75 0 0 0 0 0 0 0 0 0 0 0

0 2 66 0 0 0 0 0 0 0 0 0 0 0

0 2 32 0 0 0 0 0 0 0 0 0 0 0

0 2 57 0 0 0 0 0 0 0 0 0 0 0

0 2 76 0 0 0 0 0 0 0 0 0 0 0

0 2 77 0 0 0 0 0 0 0 0 0 0 0

0 2 40 0 0 0 0 0 0 0 0 0 0 0

0 2 60 0 0 0 0 0 0 0 0 0 0 0

0 2 61 0 0 0 0 0 0 0 0 0 0 0

0 2 55 0 0 0 0 0 0 0 0 0 0 0

0 2 63 0 0 0 0 0 0 0 0 0 0 0

0 2 63 0 0 0 0 0 0 0 0 0 0 0

0 2 48 0 0 0 0 0 0 0 0 0 0 0

0 2 53 0 0 0 0 0 0 0 0 0 0 0

0 2 76 0 0 0 0 0 0 0 0 0 0 0

0 2 56 0 0 0 0 0 0 0 0 0 0 0

0 2 64 0 0 0 0 0 0 0 0 0 0 0

0 2 55 0 0 0 0 0 0 0 0 0 0 0

0 2 71 0 0 0 0 0 0 0 0 0 0 0

0 2 56 0 0 0 0 0 0 0 0 0 0 0

0 2 63 0 0 0 0 0 0 0 0 0 0 0

0 2 82 0 0 0 0 0 0 0 0 0 0 0

0 2 65 0 0 0 0 0 0 0 0 0 0 0

0 2 53 0 0 0 0 0 0 0 0 0 0 0

0 2 68 0 0 0 0 0 0 0 0 0 0 0

0 2 67 0 0 0 0 0 0 0 0 0 0 0

0 2 54 0 0 0 0 0 0 0 0 0 0 0

0 2 83 0 0 0 0 0 0 0 0 0 0 0

0 2 77 0 0 0 0 0 0 0 0 0 0 0

0 2 46 0 0 0 0 0 0 0 0 0 0 0

0 2 59 0 0 0 0 0 0 0 0 0 0 0

0 2 47 0 0 0 0 0 0 0 0 0 0 0

0 2 48 0 0 0 0 0 0 0 0 0 0 0

0 2 48 0 0 0 0 0 0 0 0 0 0 0

0 2 49 0 0 0 0 0 0 0 0 0 0 0

0 2 58 0 0 0 0 0 0 0 0 0 0 0

0 2 58 0 0 0 0 0 0 0 0 0 0 0

0 2 46 0 0 0 0 0 0 0 0 0 0 0

0 2 73 0 0 0 0 0 0 0 0 0 0 0

0 2 46 0 0 0 0 0 0 0 0 0 0 0

0 2 57 0 0 0 0 0 0 0 0 0 0 0

0 2 60 0 0 0 0 0 0 0 0 0 0 0

0 2 60 0 0 0 0 0 0 0 0 0 0 0

0 2 57 0 0 0 0 0 0 0 0 0 0 0

0 2 24 0 0 0 0 0 0 0 0 0 0 0

0 2 47 0 0 0 0 0 0 0 0 0 0 0

0 2 59 0 0 0 0 0 0 0 0 0 0 0

0 2 58 0 0 0 0 0 0 0 0 0 0 0

0 2 59 0 0 0 0 0 0 0 0 0 0 0

0 2 60 0 0 0 0 0 0 0 0 0 0 0

0 2 62 0 0 0 0 0 0 0 0 0 0 0

0 2 62 0 0 0 0 0 0 0 0 0 0 0

0 2 47 0 0 0 0 0 0 0 0 0 0 0

0 2 50 0 0 0 0 0 0 0 0 0 0 0

0 2 14 0 0 0 0 0 0 0 0 0 0 0

0 2 26 0 0 0 0 0 0 0 0 0 0 0

0 2 69 0 0 0 0 0 0 0 0 0 0 0

0 2 26 0 0 0 0 0 0 0 0 0 0 0

0 2 58 0 0 0 0 0 0 0 0 0 0 0

0 2 70 0 0 0 0 0 0 0 0 0 0 0

0 2 73 0 0 0 0 0 0 0 0 0 0 0

0 1 79 0 0 1 0 0 0 0 0 0 0 0

0 1 25 0 0 1 0 0 0 0 0 0 0 0

0 1 62 0 0 1 0 0 0 0 0 0 0 0

0 1 49 0 0 1 0 0 0 0 0 0 0 0

0 1 78 0 0 1 0 0 0 0 0 0 0 0

0 1 88 0 0 1 0 0 0 0 0 0 0 0

0 1 71 0 0 1 0 0 0 0 0 0 0 0

0 1 84 0 0 1 0 0 0 0 0 0 0 0

0 1 80 0 0 1 0 0 0 0 0 0 0 0

0 1 70 0 0 1 0 0 0 0 0 0 0 0

0 1 83 0 0 1 0 0 0 0 0 0 0 0

0 1 18 0 0 1 0 0 0 0 0 0 0 0

0 1 61 0 0 1 0 0 0 0 0 0 0 0

0 1 55 0 0 1 0 0 0 0 0 0 0 0

0 1 58 0 0 1 0 0 0 0 0 0 0 0

0 1 66 0 0 1 0 0 0 0 0 0 0 0

0 1 62 0 0 1 0 0 0 0 0 0 0 0

0 1 75 0 0 1 0 0 0 0 0 0 0 0

0 1 64 0 0 1 0 0 0 0 0 0 0 0

0 1 53 0 0 1 0 0 0 0 0 0 0 0

0 1 59 0 0 1 0 0 0 0 0 0 0 0

0 1 69 0 0 1 0 0 0 0 0 0 0 0

0 1 72 0 0 1 0 0 0 0 0 0 0 0

0 1 68 0 0 1 0 0 0 0 0 0 0 0

0 1 70 0 0 1 0 0 0 0 0 0 0 0

0 1 77 0 0 1 0 0 0 0 0 0 0 0

0 1 77 0 0 1 0 0 0 0 0 0 0 0

0 1 79 0 0 1 0 0 0 0 0 0 0 0

0 1 68 0 0 1 0 0 0 0 0 0 0 0

0 1 39 0 0 1 0 0 0 0 0 0 0 0

0 1 46 0 0 1 0 0 0 0 0 0 0 0

0 1 79 0 0 1 0 0 0 0 0 0 0 0

0 1 77 0 0 1 0 0 0 0 0 0 0 0

0 1 71 0 0 1 0 0 0 0 0 0 0 0

0 1 60 0 0 1 0 0 0 0 0 0 0 0

0 1 69 0 0 1 0 0 0 0 0 0 0 0

0 1 70 0 0 1 0 0 0 0 0 0 0 0

0 1 91 0 0 1 0 0 0 0 0 0 0 0

0 1 61 0 0 1 0 0 0 0 0 0 0 0

0 1 68 0 0 1 0 0 0 0 0 0 0 0

0 1 58 0 0 1 0 0 0 0 0 0 0 0

0 1 92 0 0 1 0 0 0 0 0 0 0 0

0 1 76 0 0 1 0 0 0 0 0 0 0 0

0 1 91 0 0 1 0 0 0 0 0 0 0 0

0 1 75 0 0 1 0 0 0 0 0 0 0 0

0 1 59 0 0 1 0 0 0 0 0 0 0 0

0 1 46 0 0 1 0 0 0 0 0 0 0 0

0 1 71 0 0 1 0 0 0 0 0 0 0 0

0 1 78 0 0 1 0 0 0 0 0 0 0 0

0 1 77 0 0 1 0 0 0 0 0 0 0 0

0 1 82 0 0 1 0 0 0 0 0 0 0 0

0 1 68 0 0 1 0 0 0 0 0 0 0 0

0 1 76 0 0 1 0 0 0 0 0 0 0 0

0 1 46 0 0 1 0 0 0 0 0 0 0 0

0 1 61 0 0 1 0 0 0 0 0 0 0 0

0 1 68 0 0 1 0 0 0 0 0 0 0 0

0 1 81 0 0 1 0 0 0 0 0 0 0 0

0 1 66 0 0 1 0 0 0 0 0 0 0 0

0 1 72 0 0 1 0 0 0 0 0 0 0 0

0 1 53 0 0 1 0 0 0 0 0 0 0 0

0 1 54 0 0 1 0 0 0 0 0 0 0 0

0 1 44 0 0 1 0 0 0 0 0 0 0 0

0 2 24 0 0 1 0 0 0 0 0 0 0 0

0 2 71 0 0 1 0 0 0 0 0 0 0 0

0 2 71 0 0 1 0 0 0 0 0 0 0 0

0 2 61 0 0 1 0 0 0 0 0 0 0 0

0 2 94 0 0 1 0 0 0 0 0 0 0 0

0 2 89 0 0 1 0 0 0 0 0 0 0 0

0 2 64 0 0 1 0 0 0 0 0 0 0 0

0 2 66 0 0 1 0 0 0 0 0 0 0 0

0 2 78 0 0 1 0 0 0 0 0 0 0 0

0 2 75 0 0 1 0 0 0 0 0 0 0 0

0 2 66 0 0 1 0 0 0 0 0 0 0 0

0 2 61 0 0 1 0 0 0 0 0 0 0 0

0 2 84 0 0 1 0 0 0 0 0 0 0 0

0 2 64 0 0 1 0 0 0 0 0 0 0 0

0 2 58 0 0 1 0 0 0 0 0 0 0 0

0 2 24 0 0 1 0 0 0 0 0 0 0 0

0 2 86 0 0 1 0 0 0 0 0 0 0 0

0 2 60 0 0 1 0 0 0 0 0 0 0 0

0 2 75 0 0 1 0 0 0 0 0 0 0 0

0 2 73 0 0 1 0 0 0 0 0 0 0 0

0 2 67 0 0 1 0 0 0 0 0 0 0 0

0 2 81 0 0 1 0 0 0 0 0 0 0 0

0 2 57 0 0 1 0 0 0 0 0 0 0 0

0 2 24 0 0 1 0 0 0 0 0 0 0 0

0 2 79 0 0 1 0 0 0 0 0 0 0 0

0 2 67 0 0 1 0 0 0 0 0 0 0 0

0 2 24 0 0 1 0 0 0 0 0 0 0 0

0 2 24 0 0 1 0 0 0 0 0 0 0 0

0 2 76 0 0 1 0 0 0 0 0 0 0 0

0 2 74 0 0 1 0 0 0 0 0 0 0 0

0 2 66 0 0 1 0 0 0 0 0 0 0 0

0 2 45 0 0 1 0 0 0 0 0 0 0 0

0 2 59 0 0 1 0 0 0 0 0 0 0 0

0 2 58 0 0 1 0 0 0 0 0 0 0 0

0 2 80 0 0 1 0 0 0 0 0 0 0 0

0 2 76 0 0 1 0 0 0 0 0 0 0 0

0 2 72 0 0 1 0 0 0 0 0 0 0 0

0 2 68 0 0 1 0 0 0 0 0 0 0 0

0 2 58 0 0 1 0 0 0 0 0 0 0 0

0 2 72 0 0 1 0 0 0 0 0 0 0 0

0 2 61 0 0 1 0 0 0 0 0 0 0 0

0 2 61 0 0 1 0 0 0 0 0 0 0 0

0 2 76 0 0 1 0 0 0 0 0 0 0 0

0 2 90 0 0 1 0 0 0 0 0 0 0 0

0 2 76 0 0 1 0 0 0 0 0 0 0 0

0 2 62 0 0 1 0 0 0 0 0 0 0 0

0 2 75 0 0 1 0 0 0 0 0 0 0 0

0 2 76 0 0 1 0 0 0 0 0 0 0 0

0 2 85 0 0 1 0 0 0 0 0 0 0 0

0 2 65 0 0 1 0 0 0 0 0 0 0 0

0 2 71 0 0 1 0 0 0 0 0 0 0 0

0 2 78 0 0 1 0 0 0 0 0 0 0 0

0 2 69 0 0 1 0 0 0 0 0 0 0 0

0 2 61 0 0 1 0 0 0 0 0 0 0 0

0 2 67 0 0 1 0 0 0 0 0 0 0 0

0 2 58 0 0 1 0 0 0 0 0 0 0 0

0 2 74 0 0 1 0 0 0 0 0 0 0 0

0 2 57 0 0 1 0 0 0 0 0 0 0 0

0 2 80 0 0 1 0 0 0 0 0 0 0 0

0 2 84 0 0 1 0 0 0 0 0 0 0 0

0 2 60 0 0 1 0 0 0 0 0 0 0 0

0 2 59 0 0 1 0 0 0 0 0 0 0 0

0 2 80 0 0 1 0 0 0 0 0 0 0 0

0 2 59 0 0 1 0 0 0 0 0 0 0 0

0 2 59 0 0 1 0 0 0 0 0 0 0 0

0 2 65 0 0 1 0 0 0 0 0 0 0 0

0 2 76 0 0 1 0 0 0 0 0 0 0 0

0 2 82 0 0 1 0 0 0 0 0 0 0 0

0 2 76 0 0 1 0 0 0 0 0 0 0 0

0 2 83 0 0 1 0 0 0 0 0 0 0 0

0 2 74 0 0 1 0 0 0 0 0 0 0 0

0 2 61 0 0 1 0 0 0 0 0 0 0 0

0 2 70 0 0 1 0 0 0 0 0 0 0 0

0 2 55 0 0 1 0 0 0 0 0 0 0 0

0 2 55 0 0 1 0 0 0 0 0 0 0 0

0 2 45 0 0 1 0 0 0 0 0 0 0 0

0 2 66 0 0 1 0 0 0 0 0 0 0 0

0 2 53 0 0 1 0 0 0 0 0 0 0 0

0 2 27 0 0 1 0 0 0 0 0 0 0 0

0 1 59 0 0 0 0 0 0 0 0 0 0 0

0 1 59 0 0 0 0 0 0 0 0 0 0 0

0 1 71 0 0 0 0 0 0 0 0 0 0 0

0 1 68 0 0 0 0 0 0 0 0 0 0 0

0 1 65 0 0 0 0 0 0 0 0 0 0 0

0 1 70 0 0 0 0 0 0 0 0 0 0 0

0 1 79 0 0 0 0 0 0 0 0 0 0 0

0 1 64 0 0 0 0 0 0 0 0 0 0 0

0 1 65 0 0 0 0 0 0 0 0 0 0 0

0 1 56 0 0 0 0 0 0 0 0 0 0 0

0 1 52 0 0 0 0 0 0 0 0 0 0 0

0 1 64 0 0 0 0 0 0 0 0 0 0 0

0 1 66 0 0 0 0 0 0 0 0 0 0 0

0 1 42 0 0 0 0 0 0 0 0 0 0 0

0 1 79 0 0 0 0 0 0 0 0 0 0 0

0 1 51 0 0 0 0 0 0 0 0 0 0 0

0 1 47 0 0 0 0 0 0 0 0 0 0 0

0 1 36 0 0 0 0 0 0 0 0 0 0 0

0 1 58 0 0 0 0 0 0 0 0 0 0 0

0 1 76 0 0 0 0 0 0 0 0 0 0 0

0 1 82 0 0 0 0 0 0 0 0 0 0 0

0 1 80 0 0 0 0 0 0 0 0 0 0 0

0 1 83 0 0 0 0 0 0 0 0 0 0 0

0 1 79 0 0 0 0 0 0 0 0 0 0 0

0 1 57 0 0 0 0 0 0 0 0 0 0 0

0 1 82 0 0 0 0 0 0 0 0 0 0 0

0 1 59 0 0 0 0 0 0 0 0 0 0 0

0 1 82 0 0 0 0 0 0 0 0 0 0 0

0 1 71 0 0 0 0 0 0 0 0 0 0 0

0 1 61 0 0 0 0 0 0 0 0 0 0 0

0 1 57 0 0 0 0 0 0 0 0 0 0 0

0 1 71 0 0 0 0 0 0 0 0 0 0 0

0 1 81 0 0 0 0 0 0 0 0 0 0 0

0 1 71 0 0 0 0 0 0 0 0 0 0 0

0 1 66 0 0 0 0 0 0 0 0 0 0 0

0 1 55 0 0 0 0 0 0 0 0 0 0 0

0 1 58 0 0 0 0 0 0 0 0 0 0 0

0 1 66 0 0 0 0 0 0 0 0 0 0 0

0 1 64 0 0 0 0 0 0 0 0 0 0 0

0 1 76 0 0 0 0 0 0 0 0 0 0 0

0 1 60 0 0 0 0 0 0 0 0 0 0 0

0 1 39 0 0 0 0 0 0 0 0 0 0 0

0 1 47 0 0 0 0 0 0 0 0 0 0 0

0 1 63 0 0 0 0 0 0 0 0 0 0 0

0 1 47 0 0 0 0 0 0 0 0 0 0 0

0 1 24 0 0 0 0 0 0 0 0 0 0 0

0 1 23 0 0 0 0 0 0 0 0 0 0 0

0 1 44 0 0 0 0 0 0 0 0 0 0 0

0 1 44 0 0 0 0 0 0 0 0 0 0 0

0 1 47 0 0 0 0 0 0 0 0 0 0 0

0 1 25 0 0 0 0 0 0 0 0 0 0 0

0 1 36 0 0 0 0 0 0 0 0 0 0 0

0 1 62 0 0 0 0 0 0 0 0 0 0 0

0 1 31 0 0 0 0 0 0 0 0 0 0 0

0 1 56 0 0 0 0 0 0 0 0 0 0 0

0 1 16 0 0 0 0 0 0 0 0 0 0 0

0 1 40 0 0 0 0 0 0 0 0 0 0 0

0 1 53 0 0 0 0 0 0 0 0 0 0 0

0 1 63 0 0 0 0 0 0 0 0 0 0 0

0 1 47 0 0 0 0 0 0 0 0 0 0 0

0 1 39 0 0 0 0 0 0 0 0 0 0 0

0 1 38 0 0 0 0 0 0 0 0 0 0 0

0 1 40 0 0 0 0 0 0 0 0 0 0 0

0 1 74 0 0 0 0 0 0 0 0 0 0 0

0 1 55 0 0 0 0 0 0 0 0 0 0 0

0 1 46 0 0 0 0 0 0 0 0 0 0 0

0 1 59 0 0 0 0 0 0 0 0 0 0 0

0 1 36 0 0 0 0 0 0 0 0 0 0 0

0 1 35 0 0 0 0 0 0 0 0 0 0 0

0 1 39 0 0 0 0 0 0 0 0 0 0 0

0 1 54 0 0 0 0 0 0 0 0 0 0 0

0 1 50 0 0 0 0 0 0 0 0 0 0 0

0 1 58 0 0 0 0 0 0 0 0 0 0 0

0 1 40 0 0 0 0 0 0 0 0 0 0 0

0 1 66 0 0 0 0 0 0 0 0 0 0 0

0 1 53 0 0 0 0 0 0 0 0 0 0 0

0 1 56 0 0 0 0 0 0 0 0 0 0 0

0 1 64 0 0 0 0 0 0 0 0 0 0 0

0 1 57 0 0 0 0 0 0 0 0 0 0 0

0 1 45 0 0 0 0 0 0 0 0 0 0 0

0 1 84 0 0 0 0 0 0 0 0 0 0 0

0 1 82 0 0 0 0 0 0 0 0 0 0 0

0 1 74 0 0 0 0 0 0 0 0 0 0 0

0 1 60 0 0 0 0 0 0 0 0 0 0 0

0 1 86 0 0 0 0 0 0 0 0 0 0 0

0 1 64 0 0 0 0 0 0 0 0 0 0 0

0 1 61 0 0 0 0 0 0 0 0 0 0 0

0 1 77 0 0 0 0 0 0 0 0 0 0 0

0 1 38 0 0 0 1 0 0 0 0 0 0 0

0 1 87 0 0 0 0 0 0 0 0 0 0 0

0 1 78 0 0 0 0 0 0 0 0 0 0 0

0 1 70 0 0 0 0 0 0 0 0 0 0 0

0 1 71 0 0 0 0 0 0 0 0 0 0 0

0 1 75 0 0 0 0 0 0 0 0 0 0 0

0 1 78 0 0 0 0 0 0 0 0 0 0 0

0 1 62 0 0 0 0 0 0 0 0 0 0 0

0 1 79 0 0 0 0 0 0 0 0 0 0 0

0 1 65 0 0 0 0 0 0 0 0 0 0 0

0 1 52 0 0 0 0 0 0 0 0 0 0 0

0 1 56 0 0 0 0 0 0 0 0 0 0 0

0 1 70 0 0 0 0 0 0 0 0 0 0 0

0 1 57 0 0 0 0 0 0 0 0 0 0 0

0 1 77 0 0 0 0 0 0 0 0 0 0 0

0 1 71 0 0 0 0 0 0 0 0 0 0 0

0 1 49 0 0 0 0 0 0 0 0 0 0 0

0 1 68 0 0 0 0 0 0 0 0 0 0 0

0 1 84 0 0 0 0 0 0 0 0 0 0 0

0 1 61 0 0 0 0 0 0 0 0 0 0 0

0 1 80 0 0 0 0 0 0 0 0 0 0 0

0 1 62 0 0 0 0 0 0 0 0 0 0 0

0 1 42 0 0 0 0 0 0 0 0 0 0 0

0 1 38 0 0 0 0 0 0 0 0 0 0 0

0 1 44 0 0 0 0 0 0 0 0 0 0 0

0 1 69 0 0 0 0 0 0 0 0 0 0 0

0 1 68 0 0 0 0 0 0 0 0 0 0 0

0 1 73 0 0 0 0 0 0 0 0 0 0 0

0 1 66 0 0 0 0 0 0 0 0 0 0 0

0 1 49 0 0 0 0 0 0 0 0 0 0 0

0 1 61 0 0 0 0 0 0 0 0 0 0 0

0 1 44 0 0 0 0 0 0 0 0 0 0 0

0 1 55 0 0 0 0 0 0 0 0 0 0 0

0 1 67 0 0 0 0 0 0 0 0 0 0 0

0 1 83 0 0 0 0 0 0 0 0 0 0 0

0 1 57 0 0 0 0 0 0 0 0 0 0 0

0 1 62 0 0 0 0 0 0 0 0 0 0 0

0 1 78 0 0 0 0 0 0 0 0 0 0 0

0 1 56 0 0 0 0 0 0 0 0 0 0 0

0 1 58 0 0 0 0 0 0 0 0 0 0 0

0 1 58 0 0 0 0 0 0 0 0 0 0 0

0 1 59 0 0 0 0 0 0 0 0 0 0 0

0 1 68 0 0 0 0 0 0 0 0 0 0 0

0 1 69 0 0 0 0 0 0 0 0 0 0 0

0 1 52 0 0 0 0 0 0 0 0 0 0 0

0 1 66 0 0 0 0 0 0 0 0 0 0 0

0 1 69 0 0 0 0 0 0 0 0 0 0 0

0 1 70 0 0 0 0 0 0 0 0 0 0 0

0 1 62 0 0 0 0 0 0 0 0 0 0 0

0 1 58 0 0 0 0 0 0 0 0 0 0 0

0 1 51 0 0 0 0 0 0 0 0 0 0 0

0 1 63 0 0 0 0 0 0 0 0 0 0 0

0 1 65 0 0 0 0 0 0 0 0 0 0 0

0 1 45 0 0 0 0 0 0 0 0 0 0 0

0 1 70 0 0 0 0 0 0 0 0 0 0 0

0 1 71 0 0 0 0 0 0 0 0 0 0 0

0 1 47 0 0 0 0 0 0 0 0 0 0 0

0 1 35 0 0 0 0 0 0 0 0 0 0 0

0 1 60 0 0 0 0 0 0 0 0 0 0 0

0 1 49 0 0 0 0 0 0 0 0 0 0 0

0 1 85 0 0 0 0 0 0 0 0 0 0 0

0 1 65 0 0 0 0 0 0 0 0 0 0 0

0 1 60 0 0 0 0 0 0 0 0 0 0 0

0 1 63 0 0 0 0 0 0 0 0 0 0 0

0 1 77 0 0 0 0 0 0 0 0 0 0 0

0 1 63 0 0 0 0 0 0 0 0 0 0 0

0 1 56 0 0 0 0 0 0 0 0 0 0 0

0 1 77 0 0 0 0 0 0 0 0 0 0 0

0 1 53 0 0 0 0 0 0 0 0 0 0 0

0 1 58 0 0 0 0 0 0 0 0 0 0 0

0 1 69 0 0 0 1 0 0 0 0 0 0 0

0 1 51 0 0 0 1 0 0 0 0 0 0 0

0 1 64 0 0 0 1 0 0 0 0 0 0 0

0 1 45 0 0 0 1 0 0 0 0 0 0 0

0 1 29 0 0 0 1 0 0 0 0 0 0 0

0 1 63 0 0 0 0 0 0 0 0 0 0 0

0 1 57 0 0 0 0 0 0 0 0 0 0 0

0 1 76 0 0 0 0 0 0 0 0 0 0 0

0 1 89 0 0 0 0 0 0 0 0 0 0 0

0 1 55 0 0 0 0 0 0 0 0 0 0 0

0 1 46 0 0 0 0 0 0 0 0 0 0 0

0 1 55 0 0 0 0 0 0 0 0 0 0 0

0 1 59 0 0 0 0 0 0 0 0 0 0 0

0 1 73 0 0 0 0 0 0 0 0 0 0 0

0 1 62 0 0 0 0 0 0 0 0 0 0 0

0 1 61 0 0 0 0 0 0 0 0 0 4 0

0 1 83 0 0 0 0 0 0 0 0 0 0 0

0 1 80 0 0 0 0 0 0 0 0 0 0 0

0 1 67 0 0 0 0 0 0 0 0 0 0 0

0 1 85 0 0 0 0 0 0 0 0 0 0 0

0 1 56 0 0 0 0 0 0 0 0 0 0 0

0 1 43 0 0 0 0 0 0 0 0 0 0 0

0 1 63 0 0 0 0 0 0 0 0 0 0 0

0 1 44 0 0 0 0 0 0 0 0 0 0 0

0 1 60 0 0 0 0 0 0 0 0 0 0 0

0 1 52 0 0 0 0 0 0 0 0 0 0 0

0 1 60 0 0 0 0 0 0 0 0 0 0 0

0 1 26 0 0 0 0 0 0 0 0 0 0 0

0 1 57 0 0 0 0 0 0 0 0 0 0 0

0 1 57 0 0 0 0 0 0 0 0 0 0 0

0 1 85 0 0 0 0 0 0 0 0 0 0 0

0 1 45 0 0 0 0 0 0 0 0 0 0 0

0 1 65 0 0 0 0 0 0 0 0 0 0 0

0 1 72 0 0 0 0 0 0 0 0 0 0 0

0 1 55 0 0 0 0 0 0 0 0 0 0 0

0 1 29 0 0 0 0 0 0 0 0 0 0 0

0 1 63 0 0 0 0 0 0 0 0 0 0 0

0 1 48 0 0 0 0 0 0 0 0 0 0 0

0 1 62 0 0 0 0 0 0 0 0 0 0 0

0 1 58 0 0 0 0 0 0 0 0 0 0 0

0 1 58 0 0 0 0 0 0 0 0 0 0 0

0 1 64 0 0 0 0 0 0 0 0 0 0 0

0 1 57 0 0 0 0 0 0 0 0 0 0 0

0 1 65 0 0 0 0 0 0 0 0 0 0 0

0 1 52 0 0 0 0 0 0 0 0 0 0 0

0 1 17 0 0 0 0 0 0 0 0 0 0 0

0 1 76 0 0 0 0 0 0 0 0 0 0 0

0 1 73 0 0 0 0 0 0 0 0 0 0 0

0 1 68 0 0 0 0 0 0 0 0 0 0 0

0 1 33 0 0 0 0 0 0 0 0 0 0 0

0 1 60 0 0 0 0 0 0 0 0 0 0 0

0 1 58 0 0 0 0 0 0 0 0 0 0 0

0 1 44 0 0 0 0 0 0 0 0 0 0 0

0 1 68 0 0 0 0 0 0 0 0 0 1 0

0 1 35 0 0 0 0 0 0 0 0 0 2 0

0 1 41 0 0 0 0 0 0 0 0 0 2 0

0 1 81 0 0 0 0 0 0 0 0 0 2 0

0 1 84 0 0 0 0 0 0 0 0 0 2 0

0 1 83 0 0 0 0 0 0 0 0 0 2 0

0 1 71 0 0 0 0 0 0 0 0 0 2 0

0 1 71 0 0 0 0 0 0 0 0 0 2 0

0 1 76 0 0 0 0 0 0 0 0 0 0 0

0 1 70 0 0 0 0 0 0 0 0 0 0 0

0 1 53 0 0 0 0 0 0 0 0 0 0 0

0 1 16 0 0 0 0 0 0 0 0 0 0 0

0 1 80 0 0 0 0 0 0 0 0 0 0 0

0 1 73 0 0 0 0 0 0 0 0 0 0 0

0 1 44 0 0 0 0 0 0 0 0 0 0 0

0 1 65 0 0 0 0 0 0 0 0 0 0 0

0 1 22 0 0 0 0 0 0 0 0 0 0 0

0 1 70 0 0 0 0 0 0 0 0 0 0 0

0 1 62 0 0 0 0 0 0 0 0 0 0 0

0 1 21 0 0 0 0 0 0 0 0 0 0 0

0 1 60 0 0 0 0 0 0 0 0 0 0 0

0 1 60 0 0 0 0 0 0 0 0 0 0 0

0 1 72 0 0 0 0 0 0 0 0 0 0 0

0 1 52 0 0 0 0 0 0 0 0 0 0 0

0 1 60 0 0 0 0 0 0 0 0 0 0 0

0 1 46 0 0 0 0 0 0 0 0 0 0 0

0 1 72 0 0 0 0 0 0 0 0 0 0 0

0 1 29 0 0 0 0 0 0 0 0 0 0 0

0 1 29 0 0 0 0 0 0 0 0 0 0 0

0 1 33 0 0 0 0 0 0 0 0 0 0 0

0 1 29 0 0 0 0 0 0 0 0 0 0 0

0 1 45 0 0 0 0 0 0 0 0 0 0 0

0 1 47 0 0 0 0 0 0 0 0 0 0 0

0 1 36 0 0 0 0 0 0 0 0 0 0 0

0 1 65 0 0 0 0 0 0 0 0 0 0 0

0 1 61 0 0 0 0 0 0 0 0 0 0 0

0 1 52 0 0 0 0 0 0 0 0 0 0 0

0 1 42 0 0 0 0 0 0 0 0 0 0 0

0 1 52 0 0 0 0 0 0 0 0 0 0 0

0 1 56 0 0 0 0 0 0 0 0 0 0 0

0 1 47 0 0 0 0 0 0 0 0 0 0 0

0 1 58 0 0 0 0 0 0 0 0 0 0 0

0 1 46 0 0 0 0 0 0 0 0 0 0 0

0 1 48 0 0 0 0 0 0 0 0 0 0 0

0 1 48 0 0 0 0 0 0 0 0 0 0 0

0 1 41 0 0 0 0 0 0 0 0 0 0 0

0 1 38 0 0 0 0 0 0 0 0 0 0 0

0 1 53 0 0 0 0 0 0 0 0 0 0 0

0 1 23 0 0 0 0 0 0 0 0 0 0 0

0 1 39 0 0 0 0 0 0 0 0 0 0 0

0 1 47 0 0 0 0 0 0 0 0 0 0 0

0 1 45 0 0 0 0 0 0 0 0 0 0 0

0 1 74 0 0 0 0 0 0 0 0 0 0 0

0 1 59 0 0 0 0 0 0 0 0 0 0 0

0 1 39 0 0 0 0 0 0 0 0 0 0 0

0 1 24 0 0 0 0 0 0 0 0 0 0 0

0 1 31 0 0 0 0 0 0 0 0 0 0 0

0 1 42 0 0 0 0 0 0 0 0 0 0 0

0 1 59 0 0 0 0 0 0 0 0 0 0 0

0 1 23 0 0 0 0 0 0 0 0 0 0 0

0 1 60 0 0 0 0 0 0 0 0 0 0 0

0 1 36 0 0 0 0 0 0 0 0 0 0 0

0 1 73 0 0 0 0 0 0 0 0 0 0 0

0 1 31 0 0 0 0 0 0 0 0 0 0 0

0 1 15 0 0 0 0 0 0 0 0 0 0 0

0 1 64 0 0 0 0 0 0 0 0 0 1 0

0 1 27 0 0 0 0 0 0 0 0 0 2 0

0 1 42 0 0 0 0 0 0 0 0 0 2 0

0 1 17 0 0 0 0 0 0 0 0 0 2 0

0 1 34 0 0 0 0 0 0 0 0 0 2 0

0 1 77 0 0 0 0 0 0 0 0 0 2 0

0 1 48 0 0 0 0 0 0 0 0 0 3 0

0 1 47 0 0 0 0 0 0 0 0 0 2 0

0 1 47 0 0 0 0 0 0 0 0 0 1 0

0 1 54 0 0 0 0 0 0 0 0 0 3 0

0 1 72 0 0 0 0 0 0 0 0 0 0 0

0 1 42 0 0 0 0 0 0 0 0 0 1 0

0 1 48 0 0 0 0 0 0 0 0 0 1 0

0 1 18 0 0 0 0 0 0 0 0 0 1 0

0 1 51 0 0 0 0 0 0 0 0 0 1 0

0 1 56 0 0 0 0 0 0 0 0 0 1 0

0 1 55 0 0 0 0 0 0 0 0 0 1 0

0 1 39 0 0 0 0 0 0 0 0 0 1 0

0 1 68 0 0 0 0 0 0 0 0 0 1 0

0 1 44 0 0 0 0 0 0 0 0 0 1 0

0 1 53 0 0 0 0 0 0 0 0 0 1 0

0 1 51 0 0 0 0 0 0 0 0 0 1 0

0 1 42 0 0 0 0 0 0 0 0 0 1 0

0 1 35 0 0 0 0 0 0 0 0 0 1 0

0 1 56 0 0 0 0 0 0 0 0 0 1 0

0 1 11 0 0 0 0 0 0 0 0 0 1 0

0 1 16 0 0 0 0 0 0 0 0 0 1 0

0 1 26 0 0 0 0 0 0 0 0 0 1 0

0 1 45 0 0 0 0 0 0 0 0 0 1 0

0 1 65 0 0 0 0 0 0 0 0 0 1 0

0 1 42 0 0 0 0 0 0 0 0 0 1 0

0 1 56 0 0 0 0 0 0 0 0 0 1 0

0 1 22 0 0 0 0 0 0 0 0 0 1 0

0 1 23 0 0 0 0 0 0 0 0 0 1 0

0 1 62 0 0 0 0 0 0 0 0 0 1 0

0 1 71 0 0 0 0 0 0 0 0 0 1 0

0 1 77 0 0 0 0 0 0 0 0 0 1 0

0 1 49 0 0 0 0 0 0 0 0 0 1 0

0 1 80 0 0 0 0 0 0 0 0 0 1 0

0 1 77 0 0 0 0 0 0 0 0 0 1 0

0 1 32 0 0 0 0 0 0 0 0 0 1 0

0 1 71 0 0 0 0 0 0 0 0 0 1 0

0 1 71 0 0 0 0 0 0 0 0 0 2 0

0 1 76 0 0 0 0 0 0 0 0 0 2 0

0 1 77 0 0 0 0 0 0 0 0 0 2 0

0 1 71 0 0 0 0 0 0 0 0 0 2 0

0 1 68 0 0 0 0 0 0 0 0 0 2 0

0 1 82 0 0 0 0 0 0 0 0 0 2 0

0 1 59 0 0 0 0 0 0 0 0 0 2 0

0 1 65 0 0 0 0 0 0 0 0 0 2 0

0 1 69 0 0 0 0 0 0 0 0 0 2 0

0 1 70 0 0 0 0 0 0 0 0 0 2 0

0 1 79 0 0 0 0 0 0 0 0 0 2 0

0 1 71 0 0 0 0 0 0 0 0 0 2 0

0 1 71 0 0 0 0 0 0 0 0 0 2 0

0 1 81 0 0 0 0 0 0 0 0 0 2 0

0 1 85 0 0 0 0 0 0 0 0 0 2 0

0 1 71 0 0 0 0 0 0 0 0 0 2 0

0 1 57 0 0 0 0 0 0 0 0 0 2 0

0 1 62 0 0 0 0 0 0 0 0 0 2 0

0 1 72 0 0 0 0 0 0 0 0 0 2 0

0 1 71 0 0 0 0 0 0 0 0 0 2 0

0 1 63 0 0 0 0 0 0 0 0 0 2 0

0 1 54 0 0 0 0 0 0 0 0 0 2 0

0 1 54 0 0 0 0 0 0 0 0 0 2 0

0 1 80 0 0 0 0 0 0 0 0 0 2 0

0 1 80 0 0 0 0 0 0 0 0 0 2 0

0 1 83 0 0 0 0 0 0 0 0 0 2 0

0 1 66 0 0 0 0 0 0 0 0 0 2 0

0 1 80 0 0 0 0 0 0 0 0 0 2 0

0 1 55 0 0 0 0 0 0 0 0 0 2 0

0 1 77 0 0 0 0 0 0 0 0 0 2 0

0 1 66 0 0 0 0 0 0 0 0 0 2 0

0 1 70 0 0 0 0 0 0 0 0 0 2 0

0 1 75 0 0 0 0 0 0 0 0 0 2 0

0 1 73 0 0 0 0 0 0 0 0 0 2 0

0 1 61 0 0 0 0 0 0 0 0 0 2 0

0 1 83 0 0 0 0 0 0 0 0 0 2 0

0 1 77 0 0 0 0 0 0 0 0 0 2 0

0 1 72 0 0 0 0 0 0 0 0 0 2 0

0 1 75 0 0 0 0 0 0 0 0 0 2 0

0 1 80 0 0 0 0 0 0 0 0 0 2 0

0 1 54 0 0 0 0 0 0 0 0 0 2 0

0 1 76 0 0 0 0 0 0 0 0 0 2 0

0 1 57 0 0 0 0 0 0 0 0 0 2 0

0 1 78 0 0 0 1 0 0 0 0 0 2 0

0 1 80 0 0 0 0 0 0 0 0 0 2 0

0 1 56 0 0 0 1 0 0 0 0 0 2 0

0 1 63 0 0 0 1 0 0 0 0 0 2 0

0 1 40 0 0 0 1 0 0 0 0 0 2 0

0 1 47 0 0 0 1 0 0 0 0 0 2 0

0 1 85 0 0 0 1 0 0 0 0 0 2 0

0 1 69 0 0 0 1 0 0 0 0 0 2 0

0 1 70 0 0 0 1 0 0 0 0 0 2 0

0 1 61 0 0 0 1 0 0 0 0 0 2 0

0 1 66 0 0 0 1 0 0 0 0 0 2 0

0 1 72 0 0 0 1 0 0 0 0 0 2 0

0 1 84 0 0 0 0 0 0 0 0 0 2 0

0 1 59 0 0 0 0 0 0 0 0 0 2 0

0 1 43 0 0 0 0 0 0 0 0 0 2 0

0 1 36 0 0 0 0 0 0 0 0 0 2 0

0 1 62 0 0 0 0 0 0 0 0 0 2 0

0 1 57 0 0 0 0 0 0 0 0 0 2 0

0 1 70 0 0 0 0 0 0 0 0 0 2 0

0 1 71 0 0 0 0 0 0 0 0 0 2 0

0 1 75 0 0 0 0 0 0 0 0 0 2 0

0 1 42 0 0 0 0 0 0 0 0 0 2 0

0 1 55 0 0 0 0 0 0 0 0 0 2 0

0 1 55 0 0 0 0 0 0 0 0 0 2 0

0 1 74 0 0 0 0 0 0 0 0 0 2 0

0 1 81 0 0 0 0 0 0 0 0 0 2 0

0 1 46 0 0 0 0 0 0 0 0 0 2 0

0 1 53 0 0 0 0 0 0 0 0 0 2 0

0 1 54 0 0 0 0 0 0 0 0 0 2 0

0 1 56 0 0 0 0 0 0 0 0 0 2 0

0 1 62 0 0 0 0 0 0 0 0 0 2 0

0 1 63 0 0 0 0 0 0 0 0 0 2 0

0 1 69 0 0 0 0 0 0 0 0 0 2 0

0 1 41 0 0 0 0 0 0 0 0 0 2 0

0 1 82 0 0 0 0 0 0 0 0 0 2 0

0 1 69 0 0 0 0 0 0 0 0 0 2 0

0 1 82 0 0 0 0 0 0 0 0 0 2 0

0 1 83 0 0 0 0 0 0 0 0 0 2 0

0 1 67 0 0 0 0 0 0 0 0 0 2 0

0 1 68 0 0 0 0 0 0 0 0 0 2 0

0 1 79 0 0 0 0 0 0 0 0 0 2 0

0 1 84 0 0 0 0 0 0 0 0 0 2 0

0 1 75 0 0 0 0 0 0 0 0 0 2 0

0 1 88 0 0 0 0 0 0 0 0 0 2 0

0 1 88 0 0 0 0 0 0 0 0 0 2 0

0 1 76 0 0 0 0 0 0 0 0 0 2 0

0 1 85 0 0 0 0 0 0 0 0 0 2 0

0 1 84 0 0 0 0 0 0 0 0 0 2 0

0 1 54 0 0 0 0 0 0 0 0 0 2 0

0 1 51 0 0 0 0 0 0 0 0 0 2 0

0 1 63 0 0 0 0 0 0 0 0 0 2 0

0 1 79 0 0 0 0 0 0 0 0 0 2 0

0 1 54 0 0 0 0 0 0 0 0 0 2 0

0 1 77 0 0 0 0 0 0 0 0 0 2 0

0 1 69 0 0 0 0 0 0 0 0 0 2 0

0 1 67 0 0 0 0 0 0 0 0 0 2 0

0 1 69 0 0 0 0 0 0 0 0 0 2 0

0 1 81 0 0 0 0 0 0 0 0 0 2 0

0 1 60 0 0 0 0 0 0 0 0 0 2 0

0 1 69 0 0 0 0 0 0 0 0 0 2 0

0 1 62 0 0 0 0 0 0 0 0 0 2 0

0 1 84 0 0 0 0 0 0 0 0 0 2 0

0 1 63 0 0 0 0 0 0 0 0 0 2 0

0 1 55 0 0 0 0 0 0 0 0 0 2 0

0 1 63 0 0 0 0 0 0 0 0 0 2 0

0 1 60 0 0 0 0 0 0 0 0 0 2 0

0 1 60 0 0 0 0 0 0 0 0 0 2 0

0 1 69 0 0 0 0 0 0 0 0 0 2 0

0 1 54 0 0 0 0 0 0 0 0 0 2 0

0 1 77 0 0 0 0 0 0 0 0 0 2 0

0 1 52 0 0 0 0 0 0 0 0 0 2 0

0 1 61 0 0 0 0 0 0 0 0 0 2 0

0 1 82 0 0 0 0 0 0 0 0 0 2 0

0 1 60 0 0 0 0 0 0 0 0 0 2 0

0 1 66 0 0 0 0 0 0 0 0 0 2 0

0 1 69 0 0 0 0 0 0 0 0 0 2 0

0 1 78 0 0 0 0 0 0 0 0 0 2 0

0 1 68 0 0 0 0 0 0 0 0 0 2 0

0 1 70 0 0 0 0 0 0 0 0 0 2 0

0 1 70 0 0 0 0 0 0 0 0 0 2 0

0 1 70 0 0 0 0 0 0 0 0 0 3 0

0 1 75 0 0 0 0 0 0 0 0 0 3 0

0 1 65 0 0 0 0 0 0 0 0 0 3 0

0 1 56 0 0 0 0 0 0 0 0 0 3 0

0 1 41 0 0 0 0 0 0 0 0 0 3 0

0 1 75 0 0 0 0 0 0 0 0 0 3 0

0 1 76 0 0 0 0 0 0 0 0 0 3 0

0 1 76 0 0 0 0 0 0 0 0 0 3 0

0 1 53 0 0 0 0 0 0 0 0 0 3 0

0 1 65 0 0 0 0 0 0 0 0 0 3 0

0 1 51 0 0 0 0 0 0 0 0 0 3 0

0 1 62 0 0 0 0 0 0 0 0 0 3 0

0 1 70 0 0 0 0 0 0 0 0 0 3 0

0 1 69 0 0 0 0 0 0 0 0 0 3 0

0 1 44 0 0 0 0 0 0 0 0 0 3 0

0 1 40 0 0 0 0 0 0 0 0 0 3 0

0 1 74 0 0 0 0 0 0 0 0 0 3 0

0 1 27 0 0 0 0 0 0 0 0 0 3 0

0 1 62 0 0 0 0 0 0 0 0 0 3 0

0 1 70 0 0 0 0 0 0 0 0 0 3 0

0 1 51 0 0 0 0 0 0 0 0 0 3 0

0 1 33 0 0 0 0 0 0 0 0 0 3 0

0 1 51 0 0 0 0 0 0 0 0 0 3 0

0 1 69 0 0 0 0 0 0 0 0 0 3 0

0 1 23 0 0 0 0 0 0 0 0 0 3 0

0 1 55 0 0 0 0 0 0 0 0 0 3 0

0 1 79 0 0 0 0 0 0 0 0 0 3 0

0 1 49 0 0 0 0 0 0 0 0 0 3 0

0 1 49 0 0 0 0 0 0 0 0 0 3 0

0 1 64 0 0 0 0 0 0 0 0 0 3 0

0 1 58 0 0 0 0 0 0 0 0 0 3 0

0 1 55 0 0 0 0 0 0 0 0 0 3 0

0 1 75 0 0 0 0 0 0 0 0 0 3 0

0 1 48 0 0 0 0 0 0 0 0 0 3 0

0 1 67 0 0 0 0 0 0 0 0 0 3 0

0 1 41 0 0 0 0 0 0 0 0 0 3 0

0 1 56 0 0 0 0 0 0 0 0 0 3 0

0 1 52 0 0 0 0 0 0 0 0 0 3 0

0 1 56 0 0 0 0 0 0 0 0 0 3 0

0 1 58 0 0 0 0 0 0 0 0 0 3 0

0 1 76 0 0 0 0 0 0 0 0 0 3 0

0 1 76 0 0 0 0 0 0 0 0 0 3 0

0 1 37 0 0 0 0 0 0 0 0 0 3 0

0 1 74 0 0 0 0 0 0 0 0 0 3 0

0 1 67 0 0 0 0 0 0 0 0 0 3 0

0 1 69 0 0 0 0 0 0 0 0 0 3 0

0 1 56 0 0 0 0 0 0 0 0 0 3 0

0 1 53 0 0 0 0 0 0 0 0 0 3 0

0 1 59 0 0 0 0 0 0 0 0 0 3 0

0 1 47 0 0 0 0 0 0 0 0 0 3 0

0 1 63 0 0 0 0 0 0 0 0 0 3 0

0 1 34 0 0 0 0 0 0 0 0 0 3 0

0 1 68 0 0 0 0 0 0 0 0 0 3 0

0 1 39 0 0 0 0 0 0 0 0 0 3 0

0 1 81 0 0 0 0 0 0 0 0 0 3 0

0 1 54 0 0 0 0 0 0 0 0 0 3 0

0 1 61 0 0 0 0 0 0 0 0 0 3 0

0 1 69 0 0 0 0 0 0 0 0 0 3 0

0 1 63 0 0 0 0 0 0 0 0 0 3 0

0 1 74 0 0 0 0 0 0 0 0 0 3 0

0 1 79 0 0 0 0 0 0 0 0 0 3 0

0 1 68 0 0 0 0 0 0 0 0 0 3 0

0 1 70 0 0 0 0 0 0 0 0 0 3 0

0 1 65 0 0 0 0 0 0 0 0 0 3 0

0 1 65 0 0 0 0 0 0 0 0 0 3 0

0 1 64 0 0 0 0 0 0 0 0 0 3 0

0 1 72 0 0 0 0 0 0 0 0 0 3 0

0 1 83 0 0 0 0 0 0 0 0 0 3 0

0 1 52 0 0 0 0 0 0 0 0 0 3 0

0 1 67 0 0 0 0 0 0 0 0 0 3 0

0 1 87 0 0 0 0 0 0 0 0 0 3 0

0 1 82 0 0 0 0 0 0 0 0 0 3 0

0 1 64 0 0 0 0 0 0 0 0 0 3 0

0 1 72 0 0 0 0 0 0 0 0 0 3 0

0 1 72 0 0 0 0 0 0 0 0 0 3 0

0 1 74 0 0 0 0 0 0 0 0 0 3 0

0 1 57 0 0 0 0 0 0 0 0 0 3 0

0 1 70 0 0 0 0 0 0 0 0 0 3 0

0 1 82 0 0 0 0 0 0 0 0 0 3 0

0 1 69 0 0 0 0 0 0 0 0 0 3 0

0 1 52 0 0 0 0 0 0 0 0 0 3 0

0 1 53 0 0 0 0 0 0 0 0 0 4 0

0 1 69 0 0 0 0 0 0 0 0 0 4 0

0 1 32 0 0 0 0 0 0 0 0 0 4 0

0 1 69 0 0 0 0 0 0 0 0 0 4 0

0 1 57 0 0 0 0 0 0 0 0 0 4 0

0 1 70 0 0 0 0 0 0 0 0 0 4 0

0 1 75 0 0 0 0 0 0 0 0 0 4 0

0 1 56 0 0 0 0 0 0 0 0 0 4 0

0 1 56 0 0 0 0 0 0 0 0 0 4 0

0 1 75 0 0 0 0 0 0 0 0 0 4 0

0 1 75 0 0 0 0 0 0 0 0 0 4 0

0 1 75 0 0 0 0 0 0 0 0 0 4 0

0 1 35 0 0 0 0 0 0 0 0 0 4 0

0 1 75 0 0 0 0 0 0 0 0 0 4 0

0 1 47 0 0 0 0 0 0 0 0 0 4 0

0 1 56 0 0 0 0 0 0 0 0 0 4 0

0 1 65 0 0 0 0 0 0 0 0 0 4 0

0 1 53 0 0 0 0 0 0 0 0 0 4 0

0 1 71 0 0 0 0 0 0 0 0 0 4 0

0 1 74 0 0 0 0 0 0 0 0 0 4 0

0 1 76 0 0 0 0 0 0 0 0 0 4 0

0 1 76 0 0 0 0 0 0 0 0 0 4 0

0 1 66 0 0 0 0 0 0 0 0 0 4 0

0 1 72 0 0 0 0 0 0 0 0 0 4 0

0 1 74 0 0 0 0 0 0 0 0 0 4 0

0 1 76 0 0 0 0 0 0 0 0 0 4 0

0 1 70 0 0 0 0 0 0 0 0 0 4 0

0 1 58 0 0 0 0 0 0 0 0 0 4 0

0 1 72 0 0 0 0 0 0 0 0 0 4 0

0 1 83 0 0 0 0 0 0 0 0 0 4 0

0 1 67 0 0 0 0 0 0 0 0 0 4 0

0 1 75 0 0 0 0 0 0 0 0 0 4 0

0 1 75 0 0 0 0 0 0 0 0 0 4 0

0 1 54 0 0 0 0 0 0 0 0 0 0 0

0 1 65 0 0 0 0 0 0 0 0 0 0 0

0 1 72 0 0 0 0 0 0 0 0 0 0 0

0 1 42 0 0 0 0 0 0 0 0 0 0 0

0 1 75 0 0 0 0 0 0 0 0 0 0 0

0 1 69 0 0 0 0 0 0 0 0 0 0 0

0 1 58 0 0 0 0 0 0 0 0 0 0 0

0 1 81 0 0 0 0 0 0 0 0 0 0 0

0 1 66 0 0 0 0 0 0 0 0 0 0 0

0 1 45 0 0 0 0 0 0 0 0 0 0 0

0 1 63 0 0 0 0 0 0 0 0 0 0 0

0 1 46 0 0 0 0 0 0 0 0 0 0 0

0 1 56 0 0 0 1 0 0 0 0 0 0 0

0 1 75 0 0 0 1 0 0 0 0 0 0 0

0 1 71 0 0 0 1 0 0 0 0 0 0 0

0 1 48 0 0 0 0 0 0 0 0 0 0 0

0 1 77 0 0 0 0 0 0 0 0 0 0 0

0 1 50 0 0 0 1 0 0 0 0 0 0 0

0 1 74 0 0 0 1 0 0 0 0 0 0 0

0 1 47 0 0 0 1 0 0 0 0 0 0 0

0 1 64 0 0 0 1 0 0 0 0 0 0 0

0 1 72 0 0 0 1 0 0 0 0 0 0 0

0 1 54 0 0 0 1 0 0 0 0 0 0 0

0 1 56 0 0 0 0 0 0 0 0 0 0 0

0 1 54 0 0 0 0 0 0 0 0 0 0 0

0 1 49 0 0 0 0 0 0 0 0 0 0 0

0 1 86 0 0 0 0 0 0 0 0 0 0 0

0 1 66 0 0 0 0 0 0 0 0 0 0 0

0 1 64 0 0 0 0 0 0 0 0 0 0 0

0 1 73 0 0 0 0 0 0 0 0 0 0 0

0 1 60 0 0 0 0 0 0 0 0 0 0 0

0 1 67 0 0 0 0 0 0 0 0 0 0 0

0 1 66 0 0 0 0 0 0 0 0 0 0 0

0 1 39 0 0 0 0 0 0 0 0 0 0 0

0 1 61 0 0 0 0 0 0 0 0 0 0 0

0 1 60 0 0 0 0 0 0 0 0 0 0 0

0 1 63 0 0 0 0 0 0 0 0 0 0 0

0 1 47 0 0 0 0 0 0 0 0 0 0 0

0 1 50 0 0 0 0 0 0 0 0 0 0 0

0 1 63 0 0 0 0 0 0 0 0 0 0 0

0 1 54 0 0 0 0 0 0 0 0 0 0 0

0 1 45 0 0 0 0 0 0 0 0 0 0 0

0 1 74 0 0 0 0 0 0 0 0 0 1 0

0 1 69 0 0 0 0 0 0 0 0 0 2 0

0 1 79 0 0 0 0 0 0 0 0 0 2 0

0 1 75 0 0 0 0 0 0 0 0 0 2 0

0 1 66 0 0 0 0 0 0 0 0 0 2 0

0 1 71 0 0 0 0 0 0 0 0 0 2 0

0 1 48 0 0 0 0 0 0 0 0 0 2 0

0 1 30 0 0 0 0 0 0 0 0 0 1 0

0 1 53 0 0 0 0 0 0 0 0 0 1 0

0 1 78 0 0 0 0 0 0 0 0 0 1 0

0 1 75 0 0 0 0 0 0 0 0 0 2 0

0 1 46 0 0 0 0 0 0 0 0 0 2 0

0 1 47 0 0 0 0 0 0 0 0 0 2 0

0 1 40 0 0 0 0 0 0 0 0 0 2 0

0 1 79 0 0 0 0 0 0 0 0 0 2 0

0 1 75 0 0 0 0 0 0 0 0 0 2 0

0 1 84 0 0 0 0 0 0 0 0 0 2 0

0 1 67 0 0 0 0 0 0 0 0 0 2 0

0 1 74 0 0 0 0 0 0 0 0 0 3 0

0 1 61 0 0 0 0 0 0 0 0 0 3 0

0 1 58 0 0 0 0 0 0 0 0 0 3 0

0 1 79 0 0 0 0 0 0 0 0 0 3 0

0 1 54 0 0 0 0 0 0 0 0 0 4 0

0 1 61 0 0 0 0 0 0 0 0 0 4 0

0 1 56 0 0 0 0 0 0 0 0 0 4 0

0 1 75 0 0 0 0 0 0 0 0 0 1 0

0 1 58 0 0 0 0 0 0 0 0 0 1 0

0 1 44 0 0 0 0 0 0 0 0 0 0 0

0 1 67 0 0 0 0 0 0 0 0 0 0 0

0 1 80 0 0 0 0 0 0 0 0 0 0 0

0 1 70 0 0 0 0 0 0 0 0 0 0 0

0 1 75 0 0 0 0 0 0 0 0 0 0 0

0 1 40 0 0 0 0 0 0 0 0 0 0 0

0 1 57 0 0 0 0 0 0 0 0 0 0 0

0 1 54 0 0 0 0 0 0 0 0 0 0 0

0 1 76 0 0 0 0 0 0 0 0 0 0 0

0 1 76 0 0 0 0 0 0 0 0 0 0 0

0 1 76 0 0 0 0 0 0 0 0 0 0 0

0 1 76 0 0 0 0 0 0 0 0 0 0 0

0 1 76 0 0 0 0 0 0 0 0 0 0 0

0 1 68 0 0 0 0 0 0 0 0 0 0 0

0 1 76 0 0 0 0 0 0 0 0 0 0 0

0 1 60 0 0 0 0 0 0 0 0 0 0 0

0 1 78 0 0 0 0 0 0 0 0 0 0 0

0 1 73 0 0 0 0 0 0 0 0 0 0 0

0 1 74 0 0 0 0 0 0 0 0 0 0 0

0 1 74 0 0 0 0 0 0 0 0 0 0 0

0 1 76 0 0 0 0 0 0 0 0 0 1 0

0 1 77 0 0 0 0 0 0 0 0 0 2 0

0 1 77 0 0 0 0 0 0 0 0 0 2 0

0 1 77 0 0 0 0 0 0 0 0 0 2 0

0 1 77 0 0 0 0 0 0 0 0 0 2 0

0 1 77 0 0 0 0 0 0 0 0 0 2 0

0 1 77 0 0 0 0 0 0 0 0 0 2 0

0 1 78 0 0 0 0 0 0 0 0 0 2 0

0 1 78 0 0 0 0 0 0 0 0 0 3 0

0 1 78 0 0 0 0 0 0 0 0 0 0 0

0 1 78 0 0 0 0 0 0 0 0 0 0 0

0 1 78 0 0 0 0 0 0 0 0 0 0 0

0 1 78 0 0 0 0 0 0 0 0 0 0 0

0 1 79 0 0 0 0 0 0 0 0 0 0 0

0 1 78 0 0 0 0 0 0 0 0 0 0 0

0 1 78 0 0 0 0 0 0 0 0 0 0 0

0 1 77 0 0 0 0 0 0 0 0 0 0 0

0 1 77 0 0 0 0 0 0 0 0 0 2 0

0 1 56 0 0 0 0 0 0 0 0 0 2 0

0 1 79 0 0 0 0 0 0 0 0 0 2 0

0 1 78 0 0 0 0 0 0 0 0 0 2 0

0 1 66 0 0 0 0 0 0 0 0 0 3 0

0 1 65 0 0 0 0 0 0 0 0 0 3 0

0 1 54 0 0 0 0 0 0 0 0 0 3 0

0 1 72 0 0 0 0 0 0 0 0 0 0 0

0 1 70 0 0 0 0 0 0 0 0 0 0 0

0 1 75 0 0 0 0 0 0 0 0 0 0 0

0 1 76 0 0 0 0 0 0 0 0 0 0 0

0 1 73 0 0 0 0 0 0 0 0 0 0 0

0 1 62 0 0 0 0 0 0 0 0 0 0 0

0 1 65 0 0 0 0 0 0 0 0 0 0 0

0 1 82 0 0 0 0 0 0 0 0 0 0 0

0 1 74 0 0 0 0 0 0 0 0 0 0 0

0 1 77 0 0 0 0 0 0 0 0 0 0 0

0 1 56 0 0 0 0 0 0 0 0 0 0 0

0 1 62 0 0 0 0 0 0 0 0 0 0 0

0 1 84 0 0 0 0 0 0 0 0 0 0 0

0 1 71 0 0 0 0 0 0 0 0 0 0 0

0 1 64 0 0 0 0 0 0 0 0 0 0 0

0 1 70 0 0 0 0 0 0 0 0 0 0 0

0 1 82 0 0 0 0 0 0 0 0 0 0 0

0 1 37 0 0 0 0 0 0 0 0 0 0 0

0 1 45 0 0 0 0 0 0 0 0 0 0 0

0 1 47 0 0 0 0 0 0 0 0 0 0 0

0 1 75 0 0 0 0 0 0 0 0 0 0 0

0 1 69 0 0 0 0 0 0 0 0 0 0 0

0 1 69 0 0 0 0 0 0 0 0 0 0 0

0 1 80 0 0 0 0 0 0 0 0 0 0 0

0 1 78 0 0 0 0 0 0 0 0 0 0 0

0 1 65 0 0 0 0 0 0 0 0 0 0 0

0 1 60 0 0 0 0 0 0 0 0 0 0 0

0 1 70 0 0 0 0 0 0 0 0 0 0 0

0 1 66 0 0 0 0 0 0 0 0 0 0 0

0 1 43 0 0 0 0 0 0 0 0 0 0 0

0 1 61 0 0 0 0 0 0 0 0 0 0 0

0 1 64 0 0 0 0 0 0 0 0 0 0 0

0 1 56 0 0 0 0 0 0 0 0 0 0 0

0 1 57 0 0 0 0 0 0 0 0 0 0 0

0 1 65 0 0 0 0 0 0 0 0 0 0 0

0 1 70 0 0 0 0 0 0 0 0 0 0 0

0 1 84 0 0 0 0 0 0 0 0 0 0 0

0 1 81 0 0 0 0 0 0 0 0 0 0 0

0 1 56 0 0 0 0 0 0 0 0 0 0 0

0 1 70 0 0 0 0 0 0 0 0 0 0 0

0 1 57 0 0 0 0 0 0 0 0 0 0 0

0 1 70 0 0 0 0 0 0 0 0 0 0 0

0 1 63 0 0 0 0 0 0 0 0 0 0 0

0 1 64 0 0 0 0 0 0 0 0 0 0 0

0 1 77 0 0 0 0 0 0 0 0 0 0 0

0 1 82 0 0 0 0 0 0 0 0 0 0 0

0 1 61 0 0 0 0 0 0 0 0 0 0 0

0 1 57 0 0 0 0 0 0 0 0 0 0 0

0 1 45 0 0 0 0 0 0 0 0 0 0 0

0 1 70 0 0 0 0 0 0 0 0 0 0 0

0 1 41 0 0 0 0 0 0 0 0 0 0 0

0 1 57 0 0 0 0 0 0 0 0 0 0 0

0 1 80 0 0 0 0 0 0 0 0 0 0 0

0 1 65 0 0 0 0 0 0 0 0 0 0 0

0 1 81 0 0 0 0 0 0 0 0 0 0 0

0 1 62 0 0 0 0 0 0 0 0 0 0 0

0 1 66 0 0 0 0 0 0 0 0 0 0 0

0 1 54 0 0 0 0 0 0 0 0 0 0 0

0 1 42 0 0 0 0 0 0 0 0 0 0 0

0 1 37 0 0 0 0 0 0 0 0 0 0 0

0 1 56 0 0 0 0 0 0 0 0 0 0 0

0 1 36 0 0 0 0 0 0 0 0 0 0 0

0 1 80 0 0 0 0 0 0 0 0 0 0 0

0 1 40 0 0 0 0 0 0 0 0 0 0 0

0 1 35 0 0 0 0 0 0 0 0 0 0 0

0 1 59 0 0 0 0 0 0 0 0 0 0 0

0 1 32 0 0 0 0 0 0 0 0 0 0 0

0 1 69 0 0 0 0 0 0 0 0 0 0 0

0 1 45 0 0 0 0 0 0 0 0 0 0 0

0 1 33 0 0 0 0 0 0 0 0 0 0 0

0 1 66 0 0 0 0 0 0 0 0 0 0 0

0 1 33 0 0 0 0 0 0 0 0 0 0 0

0 1 69 0 0 0 0 0 0 0 0 0 0 0

0 1 56 0 0 0 0 0 0 0 0 0 0 0

0 1 72 0 0 0 0 0 0 0 0 0 0 0

0 1 46 0 0 0 0 0 0 0 0 0 0 0

0 1 80 0 0 0 0 0 0 0 0 0 0 0

0 1 58 0 0 0 0 0 0 0 0 0 0 0

0 1 78 0 0 0 0 0 0 0 0 0 0 0

0 1 78 0 0 0 0 0 0 0 0 0 0 0

0 1 74 0 0 0 0 0 0 0 0 0 0 0

0 1 76 0 0 0 0 0 0 0 0 0 0 0

0 1 58 0 0 0 0 0 0 0 0 0 0 0

0 1 73 0 0 0 0 0 0 0 0 0 0 0

0 1 52 0 0 0 0 0 0 0 0 0 0 0

0 1 39 0 0 0 0 0 0 0 0 0 0 0

0 1 49 0 0 0 0 0 0 0 0 0 0 0

0 1 61 0 0 0 0 0 0 0 0 0 0 0

0 1 74 0 0 0 0 0 0 0 0 0 0 0

0 1 75 0 0 0 0 0 0 0 0 0 0 0

0 1 66 0 0 0 0 0 0 0 0 0 0 0

0 1 66 0 0 0 0 0 0 0 0 0 4 0

0 1 70 0 0 0 0 0 0 0 0 0 3 0

0 1 73 0 0 0 0 0 0 0 0 0 4 0

0 1 47 0 0 0 0 0 0 0 0 0 4 0

0 1 40 0 0 0 0 0 0 0 0 0 4 0

0 1 48 0 0 0 0 0 0 0 0 0 4 0

0 1 66 0 0 0 1 0 0 0 0 0 4 0

0 1 58 0 0 0 1 0 0 0 0 0 4 0

0 1 43 0 0 0 1 0 0 0 0 0 4 0

0 1 41 0 0 0 1 0 0 0 0 0 4 0

0 1 80 0 0 0 0 0 0 0 0 0 0 0

0 1 38 0 0 0 0 0 0 0 0 0 4 0

0 1 58 0 0 0 0 0 0 0 0 0 4 0

0 1 71 0 0 0 0 0 0 0 0 0 0 0

0 1 46 0 0 0 0 0 0 0 0 0 3 0

0 1 43 0 0 0 0 0 0 0 0 0 3 0

0 1 84 0 0 0 0 0 0 0 0 0 3 0

0 1 84 0 0 0 0 0 0 0 0 0 4 0

0 1 52 0 0 0 0 0 0 0 0 0 4 0

0 1 56 0 0 0 0 0 0 0 0 0 4 0

0 1 72 0 0 0 0 0 0 0 0 0 4 0

0 1 56 0 0 0 0 0 0 0 0 0 4 0

0 1 82 0 0 0 0 0 0 0 0 0 4 0

0 1 62 0 0 0 0 0 0 0 0 0 4 0

0 1 48 0 0 0 0 0 0 0 0 0 4 0

0 1 56 0 0 0 0 0 0 0 0 0 4 0

0 1 61 0 0 0 0 0 0 0 0 0 4 0

0 1 66 0 0 0 0 0 0 0 0 0 4 0

0 1 46 0 0 0 0 0 0 0 0 0 4 0

0 1 60 0 0 0 0 0 0 0 0 0 4 0

0 1 54 0 0 0 0 0 0 0 0 0 0 0

0 1 76 0 0 0 0 0 0 0 0 0 0 0

0 1 79 0 0 0 0 0 0 0 0 0 0 0

0 1 46 0 0 0 0 0 0 0 0 0 0 0

0 1 74 0 0 0 0 0 0 0 0 0 0 0

0 1 73 0 0 0 0 0 0 0 0 0 0 0

0 1 33 0 0 0 0 0 0 0 0 0 0 0

0 1 51 0 0 0 0 0 0 0 0 0 0 0

0 1 55 0 0 0 0 0 0 0 0 0 0 0

0 1 45 0 0 0 0 0 0 0 0 0 0 0

0 1 60 0 0 0 0 0 0 0 0 0 0 0

0 1 73 0 0 0 0 0 0 0 0 0 0 0

0 1 50 0 0 0 0 0 0 0 0 0 0 0

0 1 70 0 0 0 0 0 0 0 0 0 0 0

0 1 43 0 0 0 0 0 0 0 0 0 0 0

0 1 76 0 0 0 0 0 0 0 0 0 0 0

0 1 47 0 0 0 0 0 0 0 0 0 0 0

0 1 43 0 0 0 1 0 0 0 0 0 0 0

0 1 69 0 0 0 0 0 0 0 0 0 0 0

0 1 57 0 0 0 0 0 0 0 0 0 0 0

0 1 70 0 0 0 0 0 0 0 0 0 0 0

0 1 72 0 0 0 0 0 0 0 0 0 0 0

0 1 39 0 0 0 0 0 0 0 0 0 0 0

0 1 69 0 0 0 0 0 0 0 0 0 0 0

0 1 76 0 0 0 0 0 0 0 0 0 0 0

0 1 76 0 0 0 0 0 0 0 0 0 4 0

0 1 54 0 0 0 0 0 0 0 0 0 0 0

0 1 65 0 0 0 0 0 0 0 0 0 0 0

0 1 46 0 0 0 0 0 0 0 0 0 0 0

0 1 58 0 0 0 0 0 0 0 0 0 0 0

0 1 72 0 0 0 0 0 0 0 0 0 0 0

0 1 77 0 0 0 0 0 0 0 0 0 0 0

0 1 55 0 0 0 0 0 0 0 0 0 0 0

0 1 83 0 0 0 0 0 0 0 0 0 0 0

0 1 56 0 0 0 0 0 0 0 0 0 0 0

0 1 67 0 0 0 0 0 0 0 0 0 0 0

0 1 72 0 0 0 0 0 0 0 0 0 0 0

0 1 36 0 0 0 0 0 0 0 0 0 0 0

0 1 40 0 0 0 0 0 0 0 0 0 0 0

0 1 59 0 0 0 0 0 0 0 0 0 0 0

0 1 66 0 0 0 0 0 0 0 0 0 0 0

0 1 56 0 0 0 0 0 0 0 0 0 0 0

0 1 69 0 0 0 0 0 0 0 0 0 0 0

0 1 76 0 0 0 0 0 0 0 0 0 0 0

0 1 58 0 0 0 0 0 0 0 0 0 0 0

0 1 56 0 0 0 0 0 0 0 0 0 0 0

0 1 55 0 0 0 0 0 0 0 0 0 0 0

0 1 84 0 0 0 0 0 0 0 0 0 0 0

0 1 81 0 0 0 0 0 0 0 0 0 0 0

0 1 68 0 0 0 0 0 0 0 0 0 0 0

0 1 80 0 0 0 0 0 0 0 0 0 0 0

0 1 80 0 0 0 0 0 0 0 0 0 0 0

0 1 75 0 0 0 0 0 0 0 0 0 0 0

0 1 65 0 0 0 0 0 0 0 0 0 0 0

0 1 69 0 0 0 0 0 0 0 0 0 0 0

0 1 56 0 0 0 0 0 0 0 0 0 0 0

0 1 71 0 0 0 0 0 0 0 0 0 0 0

0 1 64 0 0 0 0 0 0 0 0 0 0 0

0 1 82 0 0 0 0 0 0 0 0 0 0 0

0 1 59 0 0 0 0 0 0 0 0 0 0 0

0 1 60 0 0 0 0 0 0 0 0 0 0 0

0 1 82 0 0 0 0 0 0 0 0 0 0 0

0 1 68 0 0 0 0 0 0 0 0 0 0 0

0 1 82 0 0 0 0 0 0 0 0 0 0 0

0 1 64 0 0 0 0 0 0 0 0 0 0 0

0 1 73 0 0 0 0 0 0 0 0 0 0 0

0 1 30 0 0 0 0 0 0 0 0 0 0 0

0 1 58 0 0 0 0 0 0 0 0 0 0 0

0 1 51 0 0 0 0 0 0 0 0 0 0 0

0 1 55 0 0 0 0 0 0 0 0 0 0 0

0 1 72 0 0 0 0 0 0 0 0 0 0 0

0 1 46 0 0 0 0 0 0 0 0 0 0 0

0 1 76 0 0 0 0 0 0 0 0 0 0 0

0 1 68 0 0 0 0 0 0 0 0 0 0 0

0 1 72 0 0 0 0 0 0 0 0 0 0 0

0 1 73 0 0 0 0 0 0 0 0 0 0 0

0 1 81 0 0 0 0 0 0 0 0 0 0 0

0 1 59 0 0 0 0 0 0 0 0 0 0 0

0 1 78 0 0 0 0 0 0 0 0 0 0 0

0 1 71 0 0 0 0 0 0 0 0 0 0 0

0 1 61 0 0 0 0 0 0 0 0 0 0 0

0 1 70 0 0 0 0 0 0 0 0 0 0 0

0 1 66 0 0 0 0 0 0 0 0 0 0 0

0 1 72 0 0 0 0 0 0 0 0 0 0 0

0 1 71 0 0 0 0 0 0 0 0 0 0 0

0 1 59 0 0 0 0 0 0 0 0 0 0 0

0 1 82 0 0 0 0 0 0 0 0 0 0 0

0 1 80 0 0 0 0 0 0 0 0 0 0 0

0 1 75 0 0 0 0 0 0 0 0 0 0 0

0 1 71 0 0 0 0 0 0 0 0 0 0 0

0 1 65 0 0 0 0 0 0 0 0 0 0 0

0 1 63 0 0 0 0 0 0 0 0 0 0 0

0 1 72 0 0 0 0 0 0 0 0 0 0 0

0 1 82 0 0 0 0 0 0 0 0 0 0 0

0 1 71 0 0 0 0 0 0 0 0 0 0 0

0 1 55 0 0 0 0 0 0 0 0 0 0 0

0 1 68 0 0 0 0 0 0 0 0 0 0 0

0 1 55 0 0 0 0 0 0 0 0 0 0 0

0 1 61 0 0 0 0 0 0 0 0 0 0 0

0 1 84 0 0 0 0 0 0 0 0 0 0 0

0 1 82 0 0 0 0 0 0 0 0 0 0 0

0 1 66 0 0 0 0 0 0 0 0 0 0 0

0 1 84 0 0 0 0 0 0 0 0 0 0 0

0 1 52 0 0 0 0 0 0 0 0 0 0 0

0 1 68 0 0 0 0 0 0 0 0 0 0 0

0 1 45 0 0 0 0 0 0 0 0 0 0 0

0 1 72 0 0 0 0 0 0 0 0 0 0 0

0 1 68 0 0 0 0 0 0 0 0 0 0 0

0 1 70 0 0 0 0 0 0 0 0 0 0 0

0 1 79 0 0 0 0 0 0 0 0 0 0 0

0 1 78 0 0 0 0 0 0 0 0 0 0 0

0 1 77 0 0 0 0 0 0 0 0 0 0 0

0 1 70 0 0 0 0 0 0 0 0 0 0 0

0 1 70 0 0 0 0 0 0 0 0 0 0 0

0 1 67 0 0 0 0 0 0 0 0 0 0 0

0 1 67 0 0 0 0 0 0 0 0 0 0 0

0 1 84 0 0 0 0 0 0 0 0 0 0 0

0 1 60 0 0 0 0 0 0 0 0 0 0 0

0 1 67 0 0 0 0 0 0 0 0 0 0 0

0 1 68 0 0 0 0 0 0 0 0 0 0 0

0 1 68 0 0 0 0 0 0 0 0 0 0 0

0 1 74 0 0 0 0 0 0 0 0 0 0 0

0 1 76 0 0 0 0 0 0 0 0 0 0 0

0 1 78 0 0 0 0 0 0 0 0 0 0 0

0 1 60 0 0 0 0 0 0 0 0 0 0 0

0 1 69 0 0 0 0 0 0 0 0 0 0 0

0 1 79 0 0 0 0 0 0 0 0 0 0 0

0 1 72 0 0 0 0 0 0 0 0 0 0 0

0 1 80 0 0 0 0 0 0 0 0 0 0 0

0 1 64 0 0 0 0 0 0 0 0 0 0 0

0 1 78 0 0 0 0 0 0 0 0 0 0 0

0 1 90 0 0 0 0 0 0 0 0 0 0 0

0 1 64 0 0 0 0 0 0 0 0 0 0 0

0 1 66 0 0 0 0 0 0 0 0 0 0 0

0 1 80 0 0 0 0 0 0 0 0 0 0 0

0 1 53 0 0 0 0 0 0 0 0 0 0 0

0 1 56 0 0 0 0 0 0 0 0 0 0 0

0 1 68 0 0 0 0 0 0 0 0 0 0 0

0 1 81 0 0 0 0 0 0 0 0 0 0 0

0 1 83 0 0 0 0 0 0 0 0 0 0 0

0 1 39 0 0 0 0 0 0 0 0 0 0 0

0 1 41 0 0 0 0 0 0 0 0 0 0 0

0 1 83 0 0 0 0 0 0 0 0 0 0 0

0 1 74 0 0 0 0 0 0 0 0 0 0 0

0 1 73 0 0 0 0 0 0 0 0 0 0 0

0 1 83 0 0 0 0 0 0 0 0 0 0 0

0 1 82 0 0 0 0 0 0 0 0 0 0 0

0 1 69 0 0 0 0 0 0 0 0 0 0 0

0 1 68 0 0 0 0 0 0 0 0 0 0 0

0 1 67 0 0 0 0 0 0 0 0 0 0 0

0 1 73 0 0 0 0 0 0 0 0 0 0 0

0 1 67 0 0 0 0 0 0 0 0 0 0 0

0 1 52 0 0 0 0 0 0 0 0 0 0 0

0 1 67 0 0 0 0 0 0 0 0 0 0 0

0 1 54 0 0 0 0 0 0 0 0 0 0 0

0 1 62 0 0 0 0 0 0 0 0 0 0 0

0 1 35 0 0 0 0 0 0 0 0 0 0 0

0 1 61 0 0 0 0 0 0 0 0 0 0 0

0 1 46 0 0 0 0 0 0 0 0 0 0 0

0 1 51 0 0 0 0 0 0 0 0 0 0 0

0 1 35 0 0 0 0 0 0 0 0 0 0 0

0 1 60 0 0 0 0 0 0 0 0 0 0 0

0 1 53 0 0 0 0 0 0 0 0 0 0 0

0 1 44 0 0 0 0 0 0 0 0 0 0 0

0 1 78 0 0 0 0 0 0 0 0 0 0 0

0 1 78 0 0 0 0 0 0 0 0 0 0 0

0 1 78 0 0 0 0 0 0 0 0 0 0 0

0 1 65 0 0 0 0 0 0 0 0 0 0 0

0 1 71 0 0 0 0 0 0 0 0 0 0 0

0 1 72 0 0 0 0 0 0 0 0 0 0 0

0 1 66 0 0 0 0 0 0 0 0 0 0 0

0 1 47 0 0 0 0 0 0 0 0 0 0 0

0 1 74 0 0 0 0 0 0 0 0 0 1 0

0 1 73 0 0 0 0 0 0 0 0 0 0 0

0 1 77 0 0 0 0 0 0 0 0 0 1 0

0 1 70 0 0 0 0 0 0 0 0 0 1 0

0 1 52 0 0 0 0 0 0 0 0 0 1 0

0 1 78 0 0 0 0 0 0 0 0 0 1 0

0 1 79 0 0 0 0 0 0 0 0 0 1 0

0 1 83 0 0 0 0 0 0 0 0 0 1 0

0 1 62 0 0 0 1 0 0 0 0 0 1 0

0 1 75 0 0 0 0 0 0 0 0 0 1 0

0 1 63 0 0 0 0 0 0 0 0 0 1 0

0 1 61 0 0 0 0 0 0 0 0 0 1 0

0 1 68 0 0 0 0 0 0 0 0 0 1 0

0 1 70 0 0 0 0 0 0 0 0 0 1 0

0 1 77 0 0 0 0 0 0 0 0 0 2 0

0 1 83 0 0 0 0 0 0 0 0 0 2 0

0 1 92 0 0 0 0 0 0 0 0 0 2 0

0 1 67 0 0 0 0 0 0 0 0 0 2 0

0 1 82 0 0 0 0 0 0 0 0 0 2 0

0 1 52 0 0 0 0 0 0 0 0 0 2 0

0 1 73 0 0 0 0 0 0 0 0 0 2 0

0 1 48 0 0 0 0 0 0 0 0 0 2 0

0 1 80 0 0 0 0 0 0 0 0 0 2 0

0 1 42 0 0 0 0 0 0 0 0 0 2 0

0 1 81 0 0 0 0 0 0 0 0 0 2 0

0 1 70 0 0 0 0 0 0 0 0 0 2 0

0 1 69 0 0 0 0 0 0 0 0 0 2 0

0 1 63 0 0 0 0 0 0 0 0 0 2 0

0 1 57 0 0 0 0 0 0 0 0 0 2 0

0 1 78 0 0 0 0 0 0 0 0 0 3 0

0 1 73 0 0 0 0 0 0 0 0 0 3 0

0 1 76 0 0 0 0 0 0 0 0 0 3 0

0 1 82 0 0 0 0 0 0 0 0 0 3 0

0 1 77 0 0 0 0 0 0 0 0 0 3 0

0 1 88 0 0 0 0 0 0 0 0 0 3 0

0 1 77 0 0 0 0 0 0 0 0 0 3 0

0 1 57 0 0 0 0 0 0 0 0 0 3 0

0 1 58 0 0 0 0 0 0 0 0 0 3 0

0 1 62 0 0 0 0 0 0 0 0 0 3 0

0 1 60 0 0 0 0 0 0 0 0 0 4 0

0 1 72 0 0 0 0 0 0 0 0 0 4 0

0 1 68 0 0 0 0 0 0 0 0 0 4 0

0 1 61 0 0 0 0 0 0 0 0 0 4 0

0 1 54 0 0 0 0 0 0 0 0 0 4 0

0 1 83 0 0 0 0 0 0 0 0 0 4 0

0 1 72 0 0 0 0 0 0 0 0 0 1 0

0 1 76 0 0 0 0 0 0 0 0 0 1 0

0 1 39 0 0 0 0 0 0 0 0 0 1 0

0 1 54 0 0 0 0 0 0 0 0 0 1 0

0 1 80 0 0 0 0 0 0 0 0 0 1 0

0 1 65 0 0 0 0 0 0 0 0 0 1 0

0 1 76 0 0 0 0 0 0 0 0 0 1 0

0 1 86 0 0 0 0 0 0 0 0 0 1 0

0 1 28 0 0 0 0 0 0 0 0 0 1 0

0 1 76 0 0 0 0 0 0 0 0 0 1 0

0 1 80 0 0 0 0 0 0 0 0 0 1 0

0 1 70 0 0 0 0 0 0 0 0 0 1 0

0 1 79 0 0 0 0 0 0 0 0 0 1 0

0 1 65 0 0 0 0 0 0 0 0 0 1 0

0 1 67 0 0 0 0 0 0 0 0 0 1 0

0 1 47 0 0 0 0 0 0 0 0 0 1 0

0 1 39 0 0 0 0 0 0 0 0 0 1 0

0 1 78 0 0 0 0 0 0 0 0 0 1 0

0 1 76 0 0 0 0 0 0 0 0 0 1 0

0 1 68 0 0 0 0 0 0 0 0 0 1 0

0 1 82 0 0 0 0 0 0 0 0 0 1 0

0 1 74 0 0 0 0 0 0 0 0 0 1 0

0 1 68 0 0 0 0 0 0 0 0 0 1 0

0 1 48 0 0 0 0 0 0 0 0 0 1 0

0 1 47 0 0 0 0 0 0 0 0 0 1 0

0 1 66 0 0 0 0 0 0 0 0 0 1 0

0 1 75 0 0 0 0 0 0 0 0 0 1 0

0 1 75 0 0 0 0 0 0 0 0 0 1 0

0 1 61 0 0 0 0 0 0 0 0 0 1 0

0 1 63 0 0 0 0 0 0 0 0 0 1 0

0 1 86 0 0 0 0 0 0 0 0 0 1 0

0 1 75 0 0 0 0 0 0 0 0 0 1 0

0 1 63 0 0 0 0 0 0 0 0 0 1 0

0 1 61 0 0 0 0 0 0 0 0 0 1 0

0 1 68 0 0 0 0 0 0 0 0 0 2 0

0 1 80 0 0 0 0 0 0 0 0 0 1 0

0 1 80 0 0 0 0 0 0 0 0 0 2 0

0 1 63 0 0 0 0 0 0 0 0 0 2 0

0 1 76 0 0 0 0 0 0 0 0 0 2 0

0 1 47 0 0 0 0 0 0 0 0 0 2 0

0 1 82 0 0 0 0 0 0 0 0 0 2 0

0 1 82 0 0 0 0 0 0 0 0 0 2 0

0 1 75 0 0 0 0 0 0 0 0 0 2 0

0 1 59 0 0 0 0 0 0 0 0 0 2 0

0 1 71 0 0 0 0 0 0 0 0 0 2 0

0 1 74 0 0 0 0 0 0 0 0 0 2 0

0 1 85 0 0 0 0 0 0 0 0 0 2 0

0 1 65 0 0 0 0 0 0 0 0 0 2 0

0 1 78 0 0 0 0 0 0 0 0 0 2 0

0 1 78 0 0 0 0 0 0 0 0 0 2 0

0 1 86 0 0 0 0 0 0 0 0 0 2 0

0 1 59 0 0 0 0 0 0 0 0 0 2 0

0 1 68 0 0 0 0 0 0 0 0 0 2 0

0 1 85 0 0 0 0 0 0 0 0 0 2 0

0 1 78 0 0 0 0 0 0 0 0 0 2 0

0 1 79 0 0 0 0 0 0 0 0 0 2 0

0 1 54 0 0 0 0 0 0 0 0 0 2 0

0 1 61 0 0 0 0 0 0 0 0 0 2 0

0 1 69 0 0 0 0 0 0 0 0 0 2 0

0 1 82 0 0 0 0 0 0 0 0 0 2 0

0 1 73 0 0 0 0 0 0 0 0 0 2 0

0 1 65 0 0 0 0 0 0 0 0 0 2 0

0 1 67 0 0 0 0 0 0 0 0 0 2 0

0 1 77 0 0 0 0 0 0 0 0 0 2 0

0 1 57 0 0 0 0 0 0 0 0 0 2 0

0 1 77 0 0 0 0 0 0 0 0 0 2 0

0 1 70 0 0 0 0 0 0 0 0 0 2 0

0 1 87 0 0 0 0 0 0 0 0 0 2 0

0 1 89 0 0 0 0 0 0 0 0 0 2 0

0 1 80 0 0 0 0 0 0 0 0 0 2 0

0 1 84 0 0 0 0 0 0 0 0 0 2 0

0 1 77 0 0 0 0 0 0 0 0 0 2 0

0 1 54 0 0 0 0 0 0 0 0 0 2 0

0 1 80 0 0 0 0 0 0 0 0 0 2 0

0 1 65 0 0 0 0 0 0 0 0 0 2 0

0 1 60 0 0 0 0 0 0 0 0 0 2 0

0 1 64 0 0 0 0 0 0 0 0 0 2 0

0 1 44 0 0 0 0 0 0 0 0 0 2 0

0 1 44 0 0 0 0 0 0 0 0 0 2 0

0 1 44 0 0 0 0 0 0 0 0 0 2 0

0 1 19 0 0 0 0 0 0 0 0 0 2 0

0 1 70 0 0 0 0 0 0 0 0 0 2 0

0 1 60 0 0 0 0 0 0 0 0 0 2 0

0 1 60 0 0 0 0 0 0 0 0 0 2 0

0 1 69 0 0 0 0 0 0 0 0 0 2 0

0 1 49 0 0 0 0 0 0 0 0 0 2 0

0 1 63 0 0 0 0 0 0 0 0 0 2 0

0 1 57 0 0 0 0 0 0 0 0 0 2 0

0 1 70 0 0 0 0 0 0 0 0 0 2 0

0 1 81 0 0 0 0 0 0 0 0 0 2 0

0 1 48 0 0 0 0 0 0 0 0 0 2 0

0 1 35 0 0 0 1 0 0 0 0 0 2 0

0 1 52 0 0 0 0 0 0 0 0 0 2 0

0 1 33 0 0 0 0 0 0 0 0 0 2 0

0 1 69 0 0 0 0 0 0 0 0 0 2 0

0 1 27 0 0 0 0 0 0 0 0 0 2 0

0 1 28 0 0 0 0 0 0 0 0 0 2 0

0 1 66 0 0 0 0 0 0 0 0 0 2 0

0 1 41 0 0 0 0 0 0 0 0 0 2 0

0 1 17 0 0 0 0 0 0 0 0 0 2 0

0 1 17 0 0 0 0 0 0 0 0 0 2 0

0 1 32 0 0 0 0 0 0 0 0 0 2 0

0 1 19 0 0 0 0 0 0 0 0 0 2 0

0 1 15 0 0 0 0 0 0 0 0 0 2 0

0 1 33 0 0 0 0 0 0 0 0 0 2 0

0 1 62 0 0 0 0 0 0 0 0 0 2 0

0 1 45 0 0 0 0 0 0 0 0 0 2 0

0 1 53 0 0 0 0 0 0 0 0 0 2 0

0 1 26 0 0 0 0 0 0 0 0 0 2 0

0 1 49 0 0 0 0 0 0 0 0 0 2 0

0 1 69 0 0 0 0 0 0 0 0 0 2 0

0 1 77 0 0 0 0 0 0 0 0 0 2 0

0 1 71 0 0 0 0 0 0 0 0 0 2 0

0 1 64 0 0 0 0 0 0 0 0 0 2 0

0 1 77 0 0 0 0 0 0 0 0 0 2 0

0 1 52 0 0 0 0 0 0 0 0 0 2 0

0 1 41 0 0 0 0 0 0 0 0 0 2 0

0 1 61 0 0 0 0 0 0 0 0 0 2 0

0 1 29 0 0 0 0 0 0 0 0 0 2 0

0 1 54 0 0 0 0 0 0 0 0 0 2 0

0 1 61 0 0 0 0 0 0 0 0 0 2 0

0 1 59 0 0 0 0 0 0 0 0 0 2 0

0 1 41 0 0 0 0 0 0 0 0 0 2 0

0 1 34 0 0 0 0 0 0 0 0 0 2 0

0 1 14 0 0 0 0 0 0 0 0 0 3 0

0 1 41 0 0 0 0 0 0 0 0 0 3 0

0 1 56 0 0 0 0 0 0 0 0 0 3 0

0 1 84 0 0 0 0 0 0 0 0 0 3 0

0 1 80 0 0 0 0 0 0 0 0 0 3 0

0 1 70 0 0 0 0 0 0 0 0 0 3 0

0 1 88 0 0 0 0 0 0 0 0 0 3 0

0 1 77 0 0 0 0 0 0 0 0 0 3 0

0 1 54 0 0 0 0 0 0 0 0 0 3 0

0 1 52 0 0 0 0 0 0 0 0 0 3 0

0 1 79 0 0 0 1 0 0 0 0 0 3 0

0 1 52 0 0 0 0 0 0 0 0 0 3 0

0 1 75 0 0 0 0 0 0 0 0 0 3 0

0 1 60 0 0 0 0 0 0 0 0 0 3 0

0 1 74 0 0 0 0 0 0 0 0 0 3 0

0 1 76 0 0 0 0 0 0 0 0 0 3 0

0 1 52 0 0 0 0 0 0 0 0 0 3 0

0 1 76 0 0 0 0 0 0 0 0 0 3 0

0 1 74 0 0 0 0 0 0 0 0 0 3 0

0 1 79 0 0 0 0 0 0 0 0 0 3 0

0 1 59 0 0 0 0 0 0 0 0 0 3 0

0 1 72 0 0 0 0 0 0 0 0 0 3 0

0 1 74 0 0 0 0 0 0 0 0 0 3 0

0 1 67 0 0 0 0 0 0 0 0 0 3 0

0 1 74 0 0 0 0 0 0 0 0 0 3 0

0 1 70 0 0 0 0 0 0 0 0 0 3 0

0 1 64 0 0 0 0 0 0 0 0 0 3 0

0 1 74 0 0 0 0 0 0 0 0 0 3 0

0 1 62 0 0 0 0 0 0 0 0 0 3 0

0 1 80 0 0 0 0 0 0 0 0 0 3 0

0 1 77 0 0 0 0 0 0 0 0 0 3 0

0 1 73 0 0 0 0 0 0 0 0 0 3 0

0 1 61 0 0 0 0 0 0 0 0 0 3 0

0 1 58 0 0 0 0 0 0 0 0 0 3 0

0 1 74 0 0 0 0 0 0 0 0 0 3 0

0 1 58 0 0 0 0 0 0 0 0 0 3 0

0 1 71 0 0 0 0 0 0 0 0 0 3 0

0 1 57 0 0 0 0 0 0 0 0 0 3 0

0 1 54 0 0 0 0 0 0 0 0 0 3 0

0 1 20 0 0 0 0 0 0 0 0 0 3 0

0 1 68 0 0 0 0 0 0 0 0 0 3 0

0 1 17 0 0 0 0 0 0 0 0 0 3 0

0 1 64 0 0 0 0 0 0 0 0 0 3 0

0 1 55 0 0 0 0 0 0 0 0 0 3 0

0 1 37 0 0 0 0 0 0 0 0 0 3 0

0 1 51 0 0 0 0 0 0 0 0 0 3 0

0 1 29 0 0 0 0 0 0 0 0 0 3 0

0 1 67 0 0 0 0 0 0 0 0 0 3 0

0 1 56 0 0 0 0 0 0 0 0 0 3 0

0 1 71 0 0 0 0 0 0 0 0 0 3 0

0 1 35 0 0 0 0 0 0 0 0 0 3 0

0 1 19 0 0 0 0 0 0 0 0 0 3 0

0 1 57 0 0 0 0 0 0 0 0 0 3 0

0 1 33 0 0 0 0 0 0 0 0 0 3 0

0 1 68 0 0 0 0 0 0 0 0 0 3 0

0 1 40 0 0 0 0 0 0 0 0 0 3 0

0 1 17 0 0 0 0 0 0 0 0 0 3 0

0 1 64 0 0 0 0 0 0 0 0 0 3 0

0 1 47 0 0 0 0 0 0 0 0 0 3 0

0 1 62 0 0 0 0 0 0 0 0 0 3 0

0 1 20 0 0 0 0 0 0 0 0 0 3 0

0 1 55 0 0 0 0 0 0 0 0 0 3 0

0 1 63 0 0 0 0 0 0 0 0 0 3 0

0 1 43 0 0 0 0 0 0 0 0 0 3 0

0 1 56 0 0 0 0 0 0 0 0 0 3 0

0 1 47 0 0 0 0 0 0 0 0 0 3 0

0 1 63 0 0 0 0 0 0 0 0 0 3 0

0 1 63 0 0 0 0 0 0 0 0 0 3 0

0 1 47 0 0 0 0 0 0 0 0 0 3 0

0 1 58 0 0 0 0 0 0 0 0 0 3 0

0 1 57 0 0 0 0 0 0 0 0 0 3 0

0 1 29 0 0 0 0 0 0 0 0 0 3 0

0 1 65 0 0 0 0 0 0 0 0 0 3 0

0 1 50 0 0 0 0 0 0 0 0 0 3 0

0 1 23 0 0 0 0 0 0 0 0 0 3 0

0 1 20 0 0 0 0 0 0 0 0 0 3 0

0 1 16 0 0 0 0 0 0 0 0 0 3 0

0 1 77 0 0 0 0 0 0 0 0 0 3 0

0 1 66 0 0 0 0 0 0 0 0 0 3 0

0 1 66 0 0 0 0 0 0 0 0 0 3 0

0 1 83 0 0 0 0 0 0 0 0 0 3 0

0 1 60 0 0 0 0 0 0 0 0 0 3 0

0 1 66 0 0 0 0 0 0 0 0 0 3 0

0 1 78 0 0 0 0 0 0 0 0 0 3 0

0 1 54 0 0 0 0 0 0 0 0 0 3 0

0 1 65 0 0 0 0 0 0 0 0 0 3 0

0 1 25 0 0 0 0 0 0 0 0 0 3 0

0 1 78 0 0 0 0 0 0 0 0 0 3 0

0 1 29 0 0 0 0 0 0 0 0 0 3 0

0 1 28 0 0 0 0 0 0 0 0 0 3 0

0 1 19 0 0 0 0 0 0 0 0 0 3 0

0 1 48 0 0 0 0 0 0 0 0 0 3 0

0 1 61 0 0 0 0 0 0 0 0 0 3 0

0 1 71 0 0 0 0 0 0 0 0 0 3 0

0 1 46 0 0 0 0 0 0 0 0 0 3 0

0 1 72 0 0 0 0 0 0 0 0 0 3 0

0 1 57 0 0 0 0 0 0 0 0 0 3 0

0 1 75 0 0 0 0 0 0 0 0 0 3 0

0 1 78 0 0 0 0 0 0 0 0 0 4 0

0 1 71 0 0 0 0 0 0 0 0 0 4 0

0 1 79 0 0 0 0 0 0 0 0 0 4 0

0 1 63 0 0 0 0 0 0 0 0 0 4 0

0 1 63 0 0 0 0 0 0 0 0 0 4 0

0 1 15 0 0 0 0 0 0 0 0 0 4 0

0 1 40 0 0 0 0 0 0 0 0 0 4 0

0 1 63 0 0 0 0 0 0 0 0 0 4 0

0 1 45 0 0 0 0 0 0 0 0 0 4 0

0 1 23 0 0 0 0 0 0 0 0 0 4 0

0 1 38 0 0 0 0 0 0 0 0 0 4 0

0 1 46 0 0 0 0 0 0 0 0 0 4 0

0 1 74 0 0 0 0 0 0 0 0 0 4 0

0 1 72 0 0 0 0 0 0 0 0 0 4 0

0 1 53 0 0 0 0 0 0 0 0 0 4 0

0 1 74 0 0 0 0 0 0 0 0 0 4 0

0 1 73 0 0 0 0 0 0 0 0 0 4 0

0 1 86 0 0 0 0 0 0 0 0 0 4 0

0 1 76 0 0 0 0 0 0 0 0 0 4 0

0 1 44 0 0 0 0 0 0 0 0 0 4 0

0 1 53 0 0 0 0 0 0 0 0 0 4 0

0 1 72 0 0 0 0 0 0 0 0 0 0 0

0 1 66 0 0 0 0 0 0 0 0 0 0 0

0 1 64 0 0 0 0 0 0 0 0 0 0 0

0 1 70 0 0 0 0 0 0 0 0 0 0 0

0 1 53 0 0 0 0 0 0 0 0 0 0 0

0 1 63 0 0 0 0 0 0 0 0 0 0 0

0 1 63 0 0 0 0 0 0 0 0 0 0 0

0 1 88 0 0 0 0 0 0 0 0 0 0 0

0 1 61 0 0 0 0 0 0 0 0 0 0 0

0 1 72 0 0 0 0 0 0 0 0 0 0 0

0 1 62 0 0 0 0 0 0 0 0 0 1 0

0 1 63 0 0 0 0 0 0 0 0 0 1 0

0 1 69 0 0 0 0 0 0 0 0 0 1 0

0 1 63 0 0 0 0 0 0 0 0 0 1 0

0 1 59 0 0 0 0 0 0 0 0 0 1 0

0 1 43 0 0 0 0 0 0 0 0 0 1 0

0 1 51 0 0 0 0 0 0 0 0 0 2 0

0 1 57 0 0 0 0 0 0 0 0 0 2 0

0 1 69 0 0 0 0 0 0 0 0 0 2 0

0 1 60 0 0 0 0 0 0 0 0 0 2 0

0 1 70 0 0 0 0 0 0 0 0 0 2 0

0 1 18 0 0 0 0 0 0 0 0 0 2 0

0 1 85 0 0 0 0 0 0 0 0 0 2 0

0 1 85 0 0 0 0 0 0 0 0 0 2 0

0 1 66 0 0 0 0 0 0 0 0 0 2 0

0 1 76 0 0 0 0 0 0 0 0 0 2 0

0 1 74 0 0 0 0 0 0 0 0 0 2 0

0 1 82 0 0 0 0 0 0 0 0 0 2 0

0 1 66 0 0 0 0 0 0 0 0 0 2 0

0 1 66 0 0 0 0 0 0 0 0 0 2 0

0 1 33 0 0 0 0 0 0 0 0 0 2 0

0 1 48 0 0 0 1 0 0 0 0 0 3 0

0 1 28 0 0 0 1 0 0 0 0 0 4 0

0 1 56 0 0 0 1 0 0 0 0 0 0 0

0 1 31 0 0 0 1 0 0 0 0 0 0 0

0 1 44 0 0 0 1 0 0 0 0 0 0 0

0 1 63 0 0 0 1 0 0 0 0 0 0 0

0 1 33 0 0 0 1 0 0 0 0 0 0 0

0 1 59 0 0 0 1 0 0 0 0 0 0 0

0 1 46 0 0 0 1 0 0 0 0 0 0 0

0 1 44 0 0 0 1 0 0 0 0 0 0 0

0 1 30 0 0 0 1 0 0 0 0 0 4 0

0 1 58 0 0 0 1 0 0 0 0 0 4 0

0 1 59 0 0 0 0 0 0 0 0 0 0 0

0 1 42 0 0 0 0 0 0 0 0 0 0 0

0 1 54 0 0 0 0 0 0 0 0 0 0 0

0 1 39 0 0 0 0 0 0 0 0 0 0 0

0 1 48 0 0 0 0 0 0 0 0 0 0 0

0 1 65 0 0 0 0 0 0 0 0 0 0 0

0 1 21 0 0 0 0 0 0 0 0 0 0 0

0 1 39 0 0 0 0 0 0 0 0 0 0 0

0 1 44 0 0 0 0 0 0 0 0 0 0 0

0 1 63 0 0 0 0 0 0 0 0 0 0 0

0 1 22 0 0 0 0 0 0 0 0 0 0 0

0 1 27 0 0 0 0 0 0 0 0 0 0 0

0 1 17 0 0 0 0 0 0 0 0 0 0 0

0 1 26 0 0 0 0 0 0 0 0 0 0 0

0 1 50 0 0 0 0 0 0 0 0 0 0 0

0 1 77 0 0 0 0 0 0 0 0 0 0 0

0 1 54 0 0 0 0 0 0 0 0 0 0 0

0 1 58 0 0 0 0 0 0 0 0 0 0 0

0 1 47 0 0 0 0 0 0 0 0 0 0 0

0 1 75 0 0 0 0 0 0 0 0 0 0 0

0 1 53 0 0 0 0 0 0 0 0 0 0 0

0 1 17 0 0 0 0 0 0 0 0 0 0 0

0 1 45 0 0 0 0 0 0 0 0 0 0 0

0 1 75 0 0 0 0 0 0 0 0 0 0 0

0 1 80 0 0 0 0 0 0 0 0 0 0 0

0 1 15 0 0 0 0 0 0 0 0 0 0 0

0 1 84 0 0 0 0 0 0 0 0 0 0 0

0 1 75 0 0 0 0 0 0 0 0 0 0 0

0 1 14 0 0 0 0 0 0 0 0 0 0 0

0 1 19 0 0 0 0 0 0 0 0 0 0 0

0 1 30 0 0 0 0 0 0 0 0 0 0 0

0 1 48 0 0 0 0 0 0 0 0 0 0 0

0 1 41 0 0 0 0 0 0 0 0 0 0 0

0 1 91 0 0 0 0 0 0 0 0 0 0 0

0 1 76 0 0 0 0 0 0 0 0 0 0 0

0 1 66 0 0 0 0 0 0 0 0 0 0 0

0 1 69 0 0 0 0 0 0 0 0 0 0 0

0 1 82 0 0 0 0 0 0 0 0 0 0 0

0 1 82 0 0 0 0 0 0 0 0 0 0 0

0 1 81 0 0 0 0 0 0 0 0 0 0 0

0 1 76 0 0 0 0 0 0 0 0 0 0 0

0 1 73 0 0 0 0 0 0 0 0 0 0 0

0 1 46 0 0 0 0 0 0 0 0 0 0 0

0 1 82 0 0 0 0 0 0 0 0 0 0 0

0 1 71 0 0 0 0 0 0 0 0 0 0 0

0 1 75 0 0 0 0 0 0 0 0 0 0 0

0 1 62 0 0 0 0 0 0 0 0 0 0 0

0 1 62 0 0 0 0 0 0 0 0 0 0 0

0 1 70 0 0 0 0 0 0 0 0 0 0 0

0 1 71 0 0 0 0 0 0 0 0 0 0 0

0 1 67 0 0 0 0 0 0 0 0 0 0 0

0 1 83 0 0 0 0 0 0 0 0 0 0 0

0 1 71 0 0 0 0 0 0 0 0 0 0 0

0 1 88 0 0 0 0 0 0 0 0 0 0 0

0 1 81 0 0 0 0 0 0 0 0 0 0 0

0 1 73 0 0 0 0 0 0 0 0 0 0 0

0 1 79 0 0 0 0 0 0 0 0 0 0 0

0 1 72 0 0 0 0 0 0 0 0 0 0 0

0 1 82 0 0 0 0 0 0 0 0 0 0 0

0 1 82 0 0 0 0 0 0 0 0 0 0 0

0 1 77 0 0 0 0 0 0 0 0 0 0 0

0 1 85 0 0 0 0 0 0 0 0 0 0 0

0 1 84 0 0 0 0 0 0 0 0 0 0 0

0 1 71 0 0 0 0 0 0 0 0 0 0 0

0 1 64 0 0 0 0 0 0 0 0 0 0 0

0 1 81 0 0 0 0 0 0 0 0 0 0 0

0 1 57 0 0 0 0 0 0 0 0 0 0 0

0 1 73 0 0 0 0 0 0 0 0 0 0 0

0 1 85 0 0 0 0 0 0 0 0 0 0 0

0 1 78 0 0 0 0 0 0 0 0 0 0 0

0 1 63 0 0 0 0 0 0 0 0 0 0 0

0 1 72 0 0 0 0 0 0 0 0 0 0 0

0 1 54 0 0 0 0 0 0 0 0 0 0 0

0 1 54 0 0 0 0 0 0 0 0 0 0 0

0 1 44 0 0 0 0 0 0 0 0 0 0 0

0 1 48 0 0 0 0 0 0 0 0 0 0 0

0 1 90 0 0 0 0 0 0 0 0 0 0 0

0 1 40 0 0 0 1 0 0 0 0 0 0 0

0 1 59 0 0 0 0 0 0 0 0 0 0 0

0 1 40 0 0 0 0 0 0 0 0 0 0 0

0 1 40 0 0 0 0 0 0 0 0 0 0 0

0 1 56 0 0 0 0 0 0 0 0 0 0 0

0 1 31 0 0 0 0 0 0 0 0 0 0 0

0 1 61 0 0 0 0 0 0 0 0 0 0 0

0 1 76 0 0 0 0 0 0 0 0 0 0 0

0 1 52 0 0 0 0 0 0 0 0 0 0 0

0 1 65 0 0 0 0 0 0 0 0 0 0 0

0 1 58 0 0 0 0 0 0 0 0 0 0 0

0 1 54 0 0 0 0 0 0 0 0 0 0 0

0 1 57 0 0 0 0 0 0 0 0 0 0 0

0 1 73 0 0 0 0 0 0 0 0 0 0 0

0 1 79 0 0 0 0 0 0 0 0 0 0 0

0 1 53 0 0 0 0 0 0 0 0 0 0 0

0 1 63 0 0 0 0 0 0 0 0 0 0 0

0 1 73 0 0 0 0 0 0 0 0 0 0 0

0 1 61 0 0 0 0 0 0 0 0 0 0 0

0 1 46 0 0 0 0 0 0 0 0 0 0 0

0 1 40 0 0 0 0 0 0 0 0 0 0 0

0 1 47 0 0 0 0 0 0 0 0 0 0 0

0 1 53 0 0 0 0 0 0 0 0 0 0 0

0 1 46 0 0 0 0 0 0 0 0 0 0 0

0 1 45 0 0 0 1 0 0 0 0 0 0 0

0 1 71 0 0 0 0 0 0 0 0 0 0 0

0 1 56 0 0 0 0 0 0 0 0 0 0 0

0 1 62 0 0 0 0 0 0 0 0 0 2 0

0 1 50 0 0 0 0 0 0 0 0 0 2 0

0 1 68 0 0 0 0 0 0 0 0 0 2 0

0 1 63 0 0 0 0 0 0 0 0 0 3 0

0 1 38 0 0 0 0 0 0 0 0 0 4 0

0 1 70 0 0 0 0 0 0 0 0 0 0 0

0 1 59 0 0 0 0 0 0 0 0 0 0 0

0 1 39 0 0 0 0 0 0 0 0 0 0 0

0 1 52 0 0 0 0 0 0 0 0 0 0 0

0 1 52 0 0 0 0 0 0 0 0 0 0 0

0 1 40 0 0 0 0 0 0 0 0 0 0 0

0 1 35 0 0 0 0 0 0 0 0 0 0 0

0 1 36 0 0 0 0 0 0 0 0 0 0 0

0 1 52 0 0 0 0 0 0 0 0 0 0 0

0 1 60 0 0 0 0 0 0 0 0 0 0 0

0 1 71 0 0 0 0 0 0 0 0 0 0 0

0 1 72 0 0 0 0 0 0 0 0 0 0 0

0 1 54 0 0 0 0 0 0 0 0 0 0 0

0 1 64 0 0 0 0 0 0 0 0 0 0 0

0 1 15 0 0 0 0 0 0 0 0 0 0 0

0 1 47 0 0 0 0 0 0 0 0 0 0 0

0 1 60 0 0 0 0 0 0 0 0 0 0 0

0 1 64 0 0 0 0 0 0 0 0 0 0 0

0 1 72 0 0 0 0 0 0 0 0 0 0 0

0 1 84 0 0 0 0 0 0 0 0 0 0 0

0 1 46 0 0 0 0 0 0 0 0 0 0 0

0 1 68 0 0 0 0 0 0 0 0 0 0 0

0 1 68 0 0 0 0 0 0 0 0 0 0 0

0 1 46 0 0 0 0 0 0 0 0 0 0 0

0 1 55 0 0 0 0 0 0 0 0 0 0 0

0 1 44 0 0 0 0 0 0 0 0 0 0 0

0 1 37 0 0 0 0 0 0 0 0 0 0 0

0 1 57 0 0 0 0 0 0 0 0 0 0 0

0 1 35 0 0 0 0 0 0 0 0 0 0 0

0 1 63 0 0 0 0 0 0 0 0 0 0 0

0 1 56 0 0 0 0 0 0 0 0 0 0 0

0 1 39 0 0 0 0 0 0 0 0 0 0 0

0 1 30 0 0 0 0 0 0 0 0 0 0 0

0 1 90 0 0 0 0 0 0 0 0 0 0 0

0 1 20 0 0 0 0 0 0 0 0 0 0 0

0 1 59 0 0 0 0 0 0 0 0 0 0 0

0 1 84 0 0 0 0 0 0 0 0 0 0 0

0 1 57 0 0 0 0 0 0 0 0 0 0 0

0 1 68 0 0 0 0 0 0 0 0 0 0 0

0 1 78 0 0 0 0 0 0 0 0 0 0 0

0 1 73 0 0 0 0 0 0 0 0 0 0 0

0 1 69 0 0 0 0 0 0 0 0 0 0 0

0 1 79 0 0 0 0 0 0 0 0 0 0 0

0 1 75 0 0 0 0 0 0 0 0 0 2 0

0 1 84 0 0 0 0 0 0 0 0 0 2 0

0 1 83 0 0 0 0 0 0 0 0 0 0 0

0 1 68 0 0 0 0 0 0 0 0 0 3 0

0 1 84 0 0 0 0 0 0 0 0 0 4 0

0 1 76 0 0 0 0 0 0 0 0 0 4 0

0 1 80 0 0 0 0 0 0 0 0 0 4 0

0 1 73 0 0 0 0 0 0 0 0 0 0 0

0 1 63 0 0 0 0 0 0 0 0 0 0 0

0 1 82 0 0 0 0 0 0 0 0 0 0 0

0 1 58 0 0 0 0 0 0 0 0 0 0 0

0 1 80 0 0 0 0 0 0 0 0 0 0 0

0 1 52 0 0 0 0 0 0 0 0 0 0 0

0 1 78 0 0 0 0 0 0 0 0 0 0 0

0 1 67 0 0 0 0 0 0 0 0 0 0 0

0 1 67 0 0 0 0 0 0 0 0 0 0 0

0 1 64 0 0 0 0 0 0 0 0 0 0 0

0 1 81 0 0 0 0 0 0 0 0 0 0 0

0 1 69 0 0 0 1 0 0 0 0 0 0 0

0 1 62 0 0 0 0 0 0 0 0 0 0 0

0 1 66 0 0 0 0 0 0 0 0 0 0 0

0 1 77 0 0 0 0 0 0 0 0 0 0 0

0 1 71 0 0 0 0 0 0 0 0 0 0 0

0 1 53 0 0 0 0 0 0 0 0 0 0 0

0 1 58 0 0 0 0 0 0 0 0 0 0 0

0 1 60 0 0 0 0 0 0 0 0 0 0 0

0 1 65 0 0 0 0 0 0 0 0 0 0 0

0 1 68 0 0 0 0 0 0 0 0 0 0 0

0 1 55 0 0 0 0 0 0 0 0 0 0 0

0 1 37 0 0 0 0 0 0 0 0 0 0 0

0 1 53 0 0 0 0 0 0 0 0 0 0 0

0 1 54 0 0 0 0 0 0 0 0 0 1 0

0 1 54 0 0 0 0 0 0 0 0 0 2 0

0 1 50 0 0 0 0 0 0 0 0 0 2 0

0 1 39 0 0 0 0 0 0 0 0 0 2 0

0 1 71 0 0 0 0 0 0 0 0 0 2 0

0 1 47 0 0 0 0 0 0 0 0 0 2 0

0 1 83 0 0 0 0 0 0 0 0 0 2 0

0 1 66 0 0 0 0 0 0 0 0 0 2 0

0 1 64 0 0 0 0 0 0 0 0 0 2 0

0 1 59 0 0 0 0 0 0 0 0 0 2 0

0 1 86 0 0 0 0 0 0 0 0 0 2 0

0 1 87 0 0 0 0 0 0 0 0 0 2 0

0 1 63 0 0 0 0 0 0 0 0 0 2 0

0 1 73 0 0 0 0 0 0 0 0 0 2 0

0 1 74 0 0 0 1 0 0 0 0 0 2 0

0 1 77 0 0 0 0 0 0 0 0 0 3 0

0 1 78 0 0 0 1 0 0 0 0 0 3 0

0 1 64 0 0 0 0 0 0 0 0 0 3 0

0 1 61 0 0 0 0 0 0 0 0 0 3 0

0 1 53 0 0 0 0 0 0 0 0 0 3 0

0 1 59 0 0 0 0 0 0 0 0 0 3 0

0 1 85 0 0 0 0 0 0 0 0 0 3 0

0 1 43 0 0 0 0 0 0 0 0 0 3 0

0 1 42 0 0 0 0 0 0 0 0 0 3 0

0 1 19 0 0 0 0 0 0 0 0 0 3 0

0 1 18 0 0 0 0 0 0 0 0 0 3 0

0 1 18 0 0 0 0 0 0 0 0 0 3 0

0 1 28 0 0 0 0 0 0 0 0 0 3 0

0 1 51 0 0 0 0 0 0 0 0 0 3 0

0 1 81 0 0 0 0 0 0 0 0 0 3 0

0 1 68 0 0 0 0 0 0 0 0 0 3 0

0 1 39 0 0 0 0 0 0 0 0 0 3 0

0 1 39 0 0 0 0 0 0 0 0 0 3 0

0 1 47 0 0 0 0 0 0 0 0 0 3 0

0 1 51 0 0 0 0 0 0 0 0 0 3 0

0 1 62 0 0 0 0 0 0 0 0 0 3 0

0 1 53 0 0 0 0 0 0 0 0 0 3 0

0 1 41 0 0 0 0 0 0 0 0 0 3 0

0 1 51 0 0 0 0 0 0 0 0 0 3 0

0 1 23 0 0 0 0 0 0 0 0 0 3 0

0 1 47 0 0 0 0 0 0 0 0 0 3 0

0 1 73 0 0 0 0 0 0 0 0 0 3 0

0 1 78 0 0 0 0 0 0 0 0 0 3 0

0 1 19 0 0 0 0 0 0 0 0 0 3 0

0 1 15 0 0 0 0 0 0 0 0 0 3 0

0 1 25 0 0 0 1 0 0 0 0 0 3 0

0 1 58 0 0 0 0 0 0 0 0 0 3 0

0 1 21 0 0 0 0 0 0 0 0 0 3 0

0 1 18 0 0 0 0 0 0 0 0 0 3 0

0 1 58 0 0 0 0 0 0 0 0 0 3 0

0 1 49 0 0 0 0 0 0 0 0 0 3 0

0 1 41 0 0 0 0 0 0 0 0 0 3 0

0 1 18 0 0 0 0 0 0 0 0 0 3 0

0 1 46 0 0 0 0 0 0 0 0 0 3 0

0 1 24 0 0 0 0 0 0 0 0 0 3 0

0 1 39 0 0 0 0 0 0 0 0 0 3 0

0 1 65 0 0 0 0 0 0 0 0 0 3 0

0 1 64 0 0 0 0 0 0 0 0 0 3 0

0 1 61 0 0 0 0 0 0 0 0 0 3 0

0 1 84 0 0 0 0 0 0 0 0 0 3 0

0 1 85 0 0 0 0 0 0 0 0 0 3 0

0 1 47 0 0 0 0 0 0 0 0 0 3 0

0 1 62 0 0 0 0 0 0 0 0 0 3 0

0 1 66 0 0 0 0 0 0 0 0 0 3 0

0 1 38 0 0 0 0 0 0 0 0 0 3 0

0 1 54 0 0 0 0 0 0 0 0 0 3 0

0 1 19 0 0 0 0 0 0 0 0 0 4 0

0 1 71 0 0 0 0 0 0 0 0 0 4 0

0 1 18 0 0 0 0 0 0 0 0 0 4 0

0 1 39 0 0 0 0 0 0 0 0 0 4 0

0 1 37 0 0 0 1 0 0 0 0 0 4 0

0 1 36 0 0 0 0 0 0 0 0 0 4 0

0 1 52 0 0 0 0 0 0 0 0 0 4 0

0 1 71 0 0 0 0 0 0 0 0 0 4 0

0 1 70 0 0 0 0 0 0 0 0 0 4 0

0 1 56 0 0 0 0 0 0 0 0 0 4 0

0 1 49 0 0 0 0 0 0 0 0 0 4 0

0 1 35 0 0 0 0 0 0 0 0 0 4 0

0 1 74 0 0 0 0 0 0 0 0 0 4 0

0 1 38 0 0 0 0 0 0 0 0 0 4 0

0 1 57 0 0 0 0 0 0 0 0 0 4 0

0 1 39 0 0 0 0 0 0 0 0 0 4 0

0 1 77 0 0 0 0 0 0 0 0 0 4 0

0 1 63 0 0 0 0 0 0 0 0 0 4 0

0 1 79 0 0 0 0 0 0 0 0 0 4 0

0 1 53 0 0 0 0 0 0 0 0 0 4 0

0 1 60 0 0 0 0 0 0 0 0 0 4 0

0 1 66 0 0 0 0 0 0 0 0 0 4 0

0 1 47 0 0 0 0 0 0 0 0 0 4 0

0 1 47 0 0 0 0 0 0 0 0 0 4 0

0 1 61 0 0 0 0 0 0 0 0 0 4 0

0 1 82 0 0 0 0 0 0 0 0 0 4 0

0 1 58 0 0 0 0 0 0 0 0 0 4 0

0 1 54 0 0 0 0 0 0 0 0 0 4 0

0 1 88 0 0 0 0 0 0 0 0 0 0 0

0 1 52 0 0 0 0 0 0 0 0 0 3 0

0 1 42 0 0 0 0 0 0 0 0 0 0 0

0 1 50 0 0 0 0 0 0 0 0 0 4 0

0 1 80 0 0 0 0 0 0 0 0 0 4 0

0 1 60 0 0 0 0 0 0 0 0 0 0 0

0 1 47 0 0 0 0 0 0 0 0 0 0 0

0 1 45 0 0 0 0 0 0 0 0 0 0 0

0 1 48 0 0 0 0 0 0 0 0 0 0 0

0 1 61 0 0 0 0 0 0 0 0 0 0 0

0 1 75 0 0 0 0 0 0 0 0 0 0 0

0 1 84 0 0 0 0 0 0 0 0 0 0 0

0 1 72 0 0 0 0 0 0 0 0 0 0 0

0 1 67 0 0 0 0 0 0 0 0 0 0 0

0 1 82 0 0 0 0 0 0 0 0 0 0 0

0 1 63 0 0 0 0 0 0 0 0 0 0 0

0 1 86 0 0 0 0 0 0 0 0 0 0 0

0 1 69 0 0 0 0 0 0 0 0 0 0 0

0 1 74 0 0 0 0 0 0 0 0 0 0 0

0 1 63 0 0 0 0 0 0 0 0 0 0 0

0 1 52 0 0 0 0 0 0 0 0 0 0 0

0 1 58 0 0 0 0 0 0 0 0 0 0 0

0 1 76 0 0 0 0 0 0 0 0 0 0 0

0 1 79 0 0 0 0 0 0 0 0 0 0 0

0 1 71 0 0 0 0 0 0 0 0 0 0 0

0 1 72 0 0 0 0 0 0 0 0 0 0 0

0 1 62 0 0 0 0 0 0 0 0 0 0 0

0 1 58 0 0 0 0 0 0 0 0 0 0 0

0 1 61 0 0 0 0 0 0 0 0 0 0 0

0 1 61 0 0 0 0 0 0 0 0 0 0 0

0 1 17 0 0 0 0 0 0 0 0 0 0 0

0 1 70 0 0 0 0 0 0 0 0 0 0 0

0 1 79 0 0 0 0 0 0 0 0 0 0 0

0 1 70 0 0 0 0 0 0 0 0 0 0 0

0 1 76 0 0 0 0 0 0 0 0 0 0 0

0 1 73 0 0 0 0 0 0 0 0 0 0 0

0 1 74 0 0 0 0 0 0 0 0 0 0 0

0 1 78 0 0 0 0 0 0 0 0 0 0 0

0 1 87 0 0 0 0 0 0 0 0 0 0 0

0 1 62 0 0 0 0 0 0 0 0 0 0 0

0 1 70 0 0 0 0 0 0 0 0 0 0 0

0 1 16 0 0 0 0 0 0 0 0 0 0 0

0 1 65 0 0 0 0 0 0 0 0 0 0 0

0 1 56 0 0 0 0 0 0 0 0 0 0 0

0 1 66 0 0 0 0 0 0 0 0 0 0 0

0 1 63 0 0 0 0 0 0 0 0 0 0 0

0 1 42 0 0 0 0 0 0 0 0 0 0 0

0 1 56 0 0 0 1 0 0 0 0 0 0 0

0 1 61 0 0 0 0 0 0 0 0 0 0 0

0 1 47 0 0 0 0 0 0 0 0 0 0 0

0 1 44 0 0 0 0 0 0 0 0 0 0 0

0 1 60 0 0 0 0 0 0 0 0 0 0 0

0 1 54 0 0 0 0 0 0 0 0 0 0 0

0 1 57 0 0 0 0 0 0 0 0 0 0 0

0 1 51 0 0 0 0 0 0 0 0 0 0 0

0 1 45 0 0 0 0 0 0 0 0 0 0 0

0 1 61 0 0 0 0 0 0 0 0 0 0 0

0 1 42 0 0 0 0 0 0 0 0 0 0 0

0 1 39 0 0 0 0 0 0 0 0 0 0 0

0 1 84 0 0 0 0 0 0 0 0 0 0 0

0 1 59 0 0 0 0 0 0 0 0 0 0 0

0 1 40 0 0 0 0 0 0 0 0 0 0 0

0 1 68 0 0 0 0 0 0 0 0 0 0 0

0 1 79 0 0 0 0 0 0 0 0 0 0 0

0 1 67 0 0 0 0 0 0 0 0 0 4 0

0 1 73 0 0 0 0 0 0 0 0 0 4 0

0 1 68 0 0 0 0 0 0 0 0 0 4 0

0 1 70 0 0 0 0 0 0 0 0 0 4 0

0 1 75 0 0 0 0 0 0 0 0 0 4 0

0 1 51 0 0 0 1 0 0 0 0 0 0 0

0 1 82 0 0 0 0 0 0 0 0 0 4 0

0 1 85 0 0 0 0 0 0 0 0 0 4 0

0 1 24 0 0 0 0 0 0 0 0 0 0 0

0 1 65 0 0 0 0 0 0 0 0 0 0 0

0 1 61 0 0 0 0 0 0 0 0 0 0 0

0 1 63 0 0 0 0 0 0 0 0 0 0 0

0 1 76 0 0 0 0 0 0 0 0 0 0 0

0 1 50 0 0 0 0 0 0 0 0 0 0 0

0 1 68 0 0 0 0 0 0 0 0 0 0 0

0 1 70 0 0 0 0 0 0 0 0 0 0 0

0 1 46 0 0 0 0 0 0 0 0 0 0 0

0 1 75 0 0 0 0 0 0 0 0 0 0 0

0 1 69 0 0 0 0 0 0 0 0 0 0 0

0 1 74 0 0 0 0 0 0 0 0 0 0 0

0 1 69 0 0 0 0 0 0 0 0 0 2 0

0 1 53 0 0 0 0 0 0 0 0 0 2 0

0 1 77 0 0 0 0 0 0 0 0 0 2 0

0 1 37 0 0 0 0 0 0 0 0 0 2 0

0 1 38 0 0 0 0 0 0 0 0 0 2 0

0 1 48 0 0 0 0 0 0 0 0 0 2 0

0 1 84 0 0 0 0 0 0 0 0 0 2 0

0 1 73 0 0 0 0 0 0 0 0 0 2 0

0 1 46 0 0 0 0 0 0 0 0 0 3 0

0 1 52 0 0 0 0 0 0 0 0 0 3 0

0 1 62 0 0 0 0 0 0 0 0 0 3 0

0 1 60 0 0 0 0 0 0 0 0 0 3 0

0 1 69 0 0 0 0 0 0 0 0 0 3 0

0 1 49 0 0 0 0 0 0 0 0 0 3 0

0 1 50 0 0 0 0 0 0 0 0 0 3 0

0 1 72 0 0 0 0 0 0 0 0 0 3 0

0 1 54 0 0 0 0 0 0 0 0 0 3 0

0 1 48 0 0 0 0 0 0 0 0 0 3 0

0 1 53 0 0 0 0 0 0 0 0 0 3 0

0 1 70 0 0 0 0 0 0 0 0 0 3 0

0 1 70 0 0 0 0 0 0 0 0 0 3 0

0 1 72 0 0 0 0 0 0 0 0 0 3 0

0 1 71 0 0 0 0 0 0 0 0 0 3 0

0 1 75 0 0 0 0 0 0 0 0 0 3 0

0 1 60 0 0 0 0 0 0 0 0 0 4 0

0 1 90 0 0 0 0 0 0 0 0 0 4 0

0 1 60 0 0 0 0 0 0 0 0 0 4 0

0 1 47 0 0 0 0 0 0 0 0 0 4 0

0 1 39 0 0 0 0 0 0 0 0 0 4 0

0 1 83 0 0 0 0 0 0 0 0 0 4 0

0 1 69 0 0 0 0 0 0 0 0 0 4 0

0 1 18 0 0 0 0 0 0 0 0 0 4 0

0 1 18 0 0 0 0 0 0 0 0 0 0 0

0 1 27 0 0 0 0 0 0 0 0 0 0 0

0 1 13 0 0 0 0 0 0 0 0 0 0 0

0 1 29 0 0 0 0 0 0 0 0 0 0 0

0 1 37 0 0 0 0 0 0 0 0 0 0 0

0 1 24 0 0 0 0 0 0 0 0 0 0 0

0 1 90 0 0 0 0 0 0 0 0 0 0 0

0 1 62 0 0 0 0 0 0 0 0 0 0 0

0 1 58 0 0 0 0 0 0 0 0 0 0 0

0 1 54 0 0 0 1 0 0 0 0 0 0 0

0 1 74 0 0 0 0 0 0 0 0 0 0 0

0 1 60 0 0 0 0 0 0 0 0 0 0 0

0 1 60 0 0 0 1 0 0 0 0 0 0 0

0 1 52 0 0 0 1 0 0 0 0 0 0 0

0 1 64 0 0 0 0 0 0 0 0 0 0 0

0 1 36 0 0 0 0 0 0 0 0 0 0 0

0 1 44 0 0 0 0 0 0 0 0 0 0 0

0 1 41 0 0 0 0 0 0 0 0 0 0 0

0 1 59 0 0 0 0 0 0 0 0 0 0 0

0 1 52 0 0 0 0 0 0 0 0 0 0 0

0 1 41 0 0 0 0 0 0 0 0 0 0 0

0 1 45 0 0 0 0 0 0 0 0 0 0 0

0 1 30 0 0 0 0 0 0 0 0 0 0 0

0 1 32 0 0 0 0 0 0 0 0 0 0 0

0 1 46 0 0 0 0 0 0 0 0 0 0 0

0 1 58 0 0 0 0 0 0 0 0 0 0 0

0 1 34 0 0 0 0 0 0 0 0 0 0 0

0 1 20 0 0 0 0 0 0 0 0 0 0 0

0 1 55 0 0 0 0 0 0 0 0 0 0 0

0 1 53 0 0 0 0 0 0 0 0 0 0 0

0 1 82 0 0 0 0 0 0 0 0 0 0 0

0 1 52 0 0 0 0 0 0 0 0 0 0 0

0 1 64 0 0 0 0 0 0 0 0 0 2 0

0 1 40 0 0 0 0 0 0 0 0 0 0 0

0 1 28 0 0 0 0 0 0 0 0 0 0 0

0 1 30 0 0 0 0 0 0 0 0 0 0 0

0 1 65 0 0 0 0 0 0 0 0 0 0 0

0 1 58 0 0 0 0 0 0 0 0 0 0 0

0 1 51 0 0 0 0 0 0 0 0 0 0 0

0 1 63 0 0 0 0 0 0 0 0 0 0 0

0 1 39 0 0 0 0 0 0 0 0 0 0 0

0 1 36 0 0 0 0 0 0 0 0 0 0 0

0 1 21 0 0 0 0 0 0 0 0 0 0 0

0 1 21 0 0 0 0 0 0 0 0 0 0 0

0 1 78 0 0 0 0 0 0 0 0 0 0 0

0 1 50 0 0 0 0 0 0 0 0 0 0 0

0 1 21 0 0 0 0 0 0 0 0 0 0 0

0 1 84 0 0 0 0 0 0 0 0 0 0 0

0 1 70 0 0 0 0 0 0 0 0 0 0 0

0 1 22 0 0 0 0 0 0 0 0 0 0 0

0 1 58 0 0 0 0 0 0 0 0 0 0 0

0 1 43 0 0 0 0 0 0 0 0 0 0 0

0 1 62 0 0 0 0 0 0 0 0 0 0 0

0 1 60 0 0 0 0 0 0 0 0 0 0 0

0 1 74 0 0 0 0 0 0 0 0 0 0 0

0 1 57 0 0 0 0 0 0 0 0 0 0 0

0 1 64 0 0 0 0 0 0 0 0 0 0 0

0 1 50 0 0 0 0 0 0 0 0 0 2 0

0 1 76 0 0 0 0 0 0 0 0 0 3 0

0 1 69 0 0 0 0 0 0 0 0 0 3 0

0 1 60 0 0 0 0 0 0 0 0 0 3 0

0 1 61 0 0 0 0 0 0 0 0 0 3 0

0 1 46 0 0 0 0 0 0 0 0 0 4 0

0 1 59 0 0 0 0 0 0 0 0 0 0 0

0 1 77 0 0 0 0 0 0 0 0 0 0 0

0 1 54 0 0 0 0 0 0 0 0 0 0 0

0 1 35 0 0 0 0 0 0 0 0 0 0 0

0 1 60 0 0 0 0 0 0 0 0 0 0 0

0 1 55 0 0 0 0 0 0 0 0 0 0 0

0 1 55 0 0 0 0 0 0 0 0 0 0 0

0 1 39 0 0 0 0 0 0 0 0 0 0 0

0 1 49 0 0 0 0 0 0 0 0 0 0 0

0 1 25 0 0 0 0 0 0 0 0 0 0 0

0 1 63 0 0 0 0 0 0 0 0 0 0 0

0 1 73 0 0 0 0 0 0 0 0 0 0 0

0 1 48 0 0 0 0 0 0 0 0 0 0 0

0 1 44 0 0 0 0 0 0 0 0 0 0 0

0 1 27 0 0 0 0 0 0 0 0 0 0 0

0 1 52 0 0 0 0 0 0 0 0 0 0 0

0 1 53 0 0 0 1 0 0 0 0 0 0 0

0 1 42 0 0 0 0 0 0 0 0 0 0 0

0 1 36 0 0 0 0 0 0 0 0 0 0 0

0 1 44 0 0 0 1 0 0 0 0 0 0 0

0 1 26 0 0 0 0 0 0 0 0 0 0 0

0 1 39 0 0 0 0 0 0 0 0 0 0 0

0 1 25 0 0 0 0 0 0 0 0 0 0 0

0 1 32 0 0 0 0 0 0 0 0 0 0 0

0 1 37 0 0 0 0 0 0 0 0 0 0 0

0 1 44 0 0 0 0 0 0 0 0 0 0 0

0 1 55 0 0 0 0 0 0 0 0 0 3 0

0 1 41 0 0 0 0 0 0 0 0 0 0 0

0 1 63 0 0 0 0 0 0 0 0 0 0 0

0 1 62 0 0 0 0 0 0 0 0 0 0 0

0 1 62 0 0 0 0 0 0 0 0 0 0 0

0 1 43 0 0 0 0 0 0 0 0 0 0 0

0 1 53 0 0 0 0 0 0 0 0 0 0 0

0 1 52 0 0 0 0 0 0 0 0 0 0 0

0 1 29 0 0 0 0 0 0 0 0 0 0 0

0 1 50 0 0 0 0 0 0 0 0 0 0 0

0 1 40 0 0 0 0 0 0 0 0 0 0 0

0 1 16 0 0 0 0 0 0 0 0 0 0 0

0 1 64 0 0 0 0 0 0 0 0 0 0 0

0 1 50 0 0 0 0 0 0 0 0 0 0 0

0 1 47 0 0 0 0 0 0 0 0 0 0 0

0 1 42 0 0 0 0 0 0 0 0 0 0 0

0 1 26 0 0 0 0 0 0 0 0 0 0 0

0 1 42 0 0 0 0 0 0 0 0 0 0 0

0 1 67 0 0 0 0 0 0 0 0 0 0 0

0 1 25 0 0 0 0 0 0 0 0 0 0 0

0 1 24 0 0 0 0 0 0 0 0 0 0 0

0 1 45 0 0 0 0 0 0 0 0 0 0 0

0 1 39 0 0 0 0 0 0 0 0 0 0 0

0 1 53 0 0 0 0 0 0 0 0 0 0 0

0 1 25 0 0 0 0 0 0 0 0 0 0 0

0 1 55 0 0 0 0 0 0 0 0 0 0 0

0 1 90 0 0 0 0 0 0 0 0 0 0 0

0 1 78 0 0 0 0 0 0 0 0 0 0 0

0 1 67 0 0 0 0 0 0 0 0 0 0 0

0 1 67 0 0 0 0 0 0 0 0 0 0 0

0 1 73 0 0 0 0 0 0 0 0 0 0 0

0 1 44 0 0 0 0 0 0 0 0 0 0 0

0 1 78 0 0 0 0 0 0 0 0 0 0 0

0 1 75 0 0 0 0 0 0 0 0 0 0 0

0 1 56 0 0 0 0 0 0 0 0 0 0 0

0 1 39 0 0 0 0 0 0 0 0 0 0 0

0 1 72 0 0 0 0 0 0 0 0 0 0 0

0 1 72 0 0 0 0 0 0 0 0 0 0 0

0 1 72 0 0 0 0 0 0 0 0 0 0 0

0 1 65 0 0 0 0 0 0 0 0 0 0 0

0 1 56 0 0 0 0 0 0 0 0 0 0 0

0 1 56 0 0 0 0 0 0 0 0 0 0 0

0 1 71 0 0 0 0 0 0 0 0 0 0 0

0 1 54 0 0 0 0 0 0 0 0 0 0 0

0 1 75 0 0 0 0 0 0 0 0 0 0 0

0 1 55 0 0 0 0 0 0 0 0 0 0 0

0 1 78 0 0 0 0 0 0 0 0 0 0 0

0 1 49 0 0 0 0 0 0 0 0 0 0 0

0 1 62 0 0 0 0 0 0 0 0 0 0 0

0 1 64 0 0 0 0 0 0 0 0 0 0 0

0 1 47 0 0 0 0 0 0 0 0 0 0 0

0 1 59 0 0 0 0 0 0 0 0 0 0 0

0 1 55 0 0 0 0 0 0 0 0 0 0 0

0 1 62 0 0 0 0 0 0 0 0 0 0 0

0 1 55 0 0 0 0 0 0 0 0 0 0 0

0 1 69 0 0 0 0 0 0 0 0 0 0 0

0 1 60 0 0 0 0 0 0 0 0 0 0 0

0 1 72 0 0 0 0 0 0 0 0 0 0 0

0 1 57 0 0 0 0 0 0 0 0 0 0 0

0 1 74 0 0 0 0 0 0 0 0 0 0 0

0 1 55 0 0 0 0 0 0 0 0 0 0 0

0 1 66 0 0 0 0 0 0 0 0 0 0 0

0 1 27 0 0 0 0 0 0 0 0 0 0 0

0 1 79 0 0 0 0 0 0 0 0 0 1 0

0 1 83 0 0 0 0 0 0 0 0 0 0 0

0 1 38 0 0 0 0 0 0 0 0 0 0 0

0 1 60 0 0 0 0 0 0 0 0 0 0 0

0 1 67 0 0 0 0 0 0 0 0 0 0 0

0 1 70 0 0 0 0 0 0 0 0 0 0 0

0 1 36 0 0 0 0 0 0 0 0 0 0 0

0 1 27 0 0 0 0 0 0 0 0 0 0 0

0 1 62 0 0 0 0 0 0 0 0 0 0 0

0 1 43 0 0 0 0 0 0 0 0 0 0 0

0 1 71 0 0 0 0 0 0 0 0 0 0 0

0 1 63 0 0 0 0 0 0 0 0 0 0 0

0 1 53 0 0 0 0 0 0 0 0 0 0 0

0 1 58 0 0 0 0 0 0 0 0 0 0 0

0 1 83 0 0 0 0 0 0 0 0 0 0 0

0 1 84 0 0 0 0 0 0 0 0 0 0 0

0 1 56 0 0 0 0 0 0 0 0 0 0 0

0 1 65 0 0 0 0 0 0 0 0 0 0 0

0 1 86 0 0 0 0 0 0 0 0 0 0 0

0 1 48 0 0 0 1 0 0 0 0 0 0 0

0 1 88 0 0 0 0 0 0 0 0 0 0 0

0 1 16 0 0 0 0 0 0 0 0 0 0 0

0 1 75 0 0 0 0 0 0 0 0 0 0 0

0 1 75 0 0 0 0 0 0 0 0 0 0 0

0 1 77 0 0 0 0 0 0 0 0 0 0 0

0 1 81 0 0 0 0 0 0 0 0 0 0 0

0 1 62 0 0 0 0 0 0 0 0 0 0 0

0 1 69 0 0 0 0 0 0 0 0 0 0 0

0 1 58 0 0 0 0 0 0 0 0 0 0 0

0 1 70 0 0 0 0 0 0 0 0 0 0 0

0 1 62 0 0 0 0 0 0 0 0 0 0 0

0 1 62 0 0 0 0 0 0 0 0 0 0 0

0 1 64 0 0 0 0 0 0 0 0 0 0 0

0 1 54 0 0 0 0 0 0 0 0 0 0 0

0 1 72 0 0 0 0 0 0 0 0 0 0 0

0 1 76 0 0 0 0 0 0 0 0 0 0 0

0 1 70 0 0 0 0 0 0 0 0 0 0 0

0 1 73 0 0 0 0 0 0 0 0 0 0 0

0 1 68 0 0 0 0 0 0 0 0 0 0 0

0 1 42 0 0 0 0 0 0 0 0 0 0 0

0 1 59 0 0 0 0 0 0 0 0 0 0 0

0 1 62 0 0 0 0 0 0 0 0 0 0 0

0 1 65 0 0 0 0 0 0 0 0 0 0 0

0 2 73 0 0 0 0 0 0 0 0 0 0 0

0 2 79 0 0 0 0 0 0 0 0 0 0 0

0 2 62 0 0 0 0 0 0 0 0 0 0 0

0 2 69 0 0 0 0 0 0 0 0 0 0 0

0 2 60 0 0 0 0 0 0 0 0 0 0 0

0 2 60 0 0 0 0 0 0 0 0 0 0 0

0 2 42 0 0 0 0 0 0 0 0 0 0 0

0 2 42 0 0 0 0 0 0 0 0 0 0 0

0 2 17 0 0 0 0 0 0 0 0 0 0 0

0 2 60 0 0 0 0 0 0 0 0 0 0 0

0 2 60 0 0 0 0 0 0 0 0 0 0 0

0 2 37 0 0 0 0 0 0 0 0 0 0 0

0 2 46 0 0 0 0 0 0 0 0 0 0 0

0 2 67 0 0 0 0 0 0 0 0 0 0 0

0 2 52 0 0 0 0 0 0 0 0 0 0 0

0 2 54 0 0 0 0 0 0 0 0 0 0 0

0 2 43 0 0 0 0 0 0 0 0 0 0 0

0 2 52 0 0 0 0 0 0 0 0 0 0 0

0 2 45 0 0 0 0 0 0 0 0 0 0 0

0 2 72 0 0 0 0 0 0 0 0 0 0 0

0 2 46 0 0 0 0 0 0 0 0 0 0 0

0 2 17 0 0 0 0 0 0 0 0 0 0 0

0 2 35 0 0 0 0 0 0 0 0 0 0 0

0 2 23 0 0 0 0 0 0 0 0 0 0 0

0 2 43 0 0 0 0 0 0 0 0 0 0 0

0 2 31 0 0 0 0 0 0 0 0 0 0 0

0 2 70 0 0 0 0 0 0 0 0 0 0 0

0 2 53 0 0 0 0 0 0 0 0 0 0 0

0 2 77 0 0 0 0 0 0 0 0 0 0 0

0 2 41 0 0 0 0 0 0 0 0 0 0 0

0 2 51 0 0 0 0 0 0 0 0 0 0 0

0 2 61 0 0 0 0 0 0 0 0 0 0 0

0 2 81 0 0 0 0 0 0 0 0 0 0 0

0 2 59 0 0 0 0 0 0 0 0 0 0 0

0 2 53 0 0 0 0 0 0 0 0 0 0 0

0 2 82 0 0 0 0 0 0 0 0 0 0 0

0 2 82 0 0 0 0 0 0 0 0 0 0 0

0 2 64 0 0 0 0 0 0 0 0 0 0 0

0 2 71 0 0 0 0 0 0 0 0 0 0 0

0 2 78 0 0 0 0 0 0 0 0 0 0 0

0 2 68 0 0 0 0 0 0 0 0 0 0 0

0 2 62 0 0 0 0 0 0 0 0 0 0 0

0 2 58 0 0 0 0 0 0 0 0 0 0 0

0 2 62 0 0 0 0 0 0 0 0 0 0 0

0 2 64 0 0 0 0 0 0 0 0 0 1 0

0 2 77 0 0 0 1 0 0 0 0 0 4 0

0 2 67 0 0 0 1 0 0 0 0 0 2 0

0 2 82 0 0 0 0 0 0 0 0 0 2 0

0 2 56 0 0 0 0 0 0 0 0 0 2 0

0 2 81 0 0 0 0 0 0 0 0 0 2 0

0 2 77 0 0 0 0 0 0 0 0 0 3 0

0 2 41 0 0 0 0 0 0 0 0 0 3 0

0 2 66 0 0 0 0 0 0 0 0 0 3 0

0 2 48 0 0 0 0 0 0 0 0 0 0 0

0 2 31 0 0 0 0 0 0 0 0 0 0 0

0 2 39 0 0 0 0 0 0 0 0 0 0 0

0 2 18 0 0 0 0 0 0 0 0 0 0 0

0 2 59 0 0 0 0 0 0 0 0 0 0 0

0 2 40 0 0 0 0 0 0 0 0 0 0 0

0 2 41 0 0 0 0 0 0 0 0 0 0 0

0 2 65 0 0 0 0 0 0 0 0 0 0 0

0 2 37 0 0 0 0 0 0 0 0 0 0 0

0 2 47 0 0 0 0 0 0 0 0 0 0 0

0 2 58 0 0 0 0 0 0 0 0 0 0 0

0 2 49 0 0 0 0 0 0 0 0 0 0 0

0 2 65 0 0 0 0 0 0 0 0 0 0 0

0 2 45 0 0 0 0 0 0 0 0 0 0 0

0 2 51 0 0 0 0 0 0 0 0 0 0 0

0 2 54 0 0 0 0 0 0 0 0 0 0 0

0 2 17 0 0 0 0 0 0 0 0 0 0 0

0 2 54 0 0 0 0 0 0 0 0 0 0 0

0 2 35 0 0 0 0 0 0 0 0 0 0 0

0 2 65 0 0 0 0 0 0 0 0 0 0 0

0 2 68 0 0 0 0 0 0 0 0 0 0 0

0 2 59 0 0 0 0 0 0 0 0 0 0 0

0 2 47 0 0 0 0 0 0 0 0 0 0 0

0 2 54 0 0 0 0 0 0 0 0 0 0 0

0 2 45 0 0 0 0 0 0 0 0 0 0 0

0 2 45 0 0 0 0 0 0 0 0 0 0 0

0 2 73 0 0 0 0 0 0 0 0 0 0 0

0 2 32 0 0 0 0 0 0 0 0 0 0 0

0 2 40 0 0 0 0 0 0 0 0 0 0 0

0 2 40 0 0 0 0 0 0 0 0 0 0 0

0 2 75 0 0 0 0 0 0 0 0 0 0 0

0 2 47 0 0 0 0 0 0 0 0 0 0 0

0 2 57 0 0 0 0 0 0 0 0 0 0 0

0 2 58 0 0 0 0 0 0 0 0 0 0 0

0 2 58 0 0 0 0 0 0 0 0 0 0 0

0 2 44 0 0 0 0 0 0 0 0 0 0 0

0 2 47 0 0 0 0 0 0 0 0 0 0 0

0 2 36 0 0 0 0 0 0 0 0 0 0 0

0 2 79 0 0 0 0 0 0 0 0 0 0 0

0 2 80 0 0 0 0 0 0 0 0 0 0 0

0 2 75 0 0 0 0 0 0 0 0 0 0 0

0 2 40 0 0 0 0 0 0 0 0 0 0 0

0 2 69 0 0 0 0 0 0 0 0 0 0 0

0 2 63 0 0 0 0 0 0 0 0 0 0 0

0 2 69 0 0 0 0 0 0 0 0 0 0 0

0 2 70 0 0 0 1 0 0 0 0 0 0 0

0 2 57 0 0 0 0 0 0 0 0 0 0 0

0 2 76 0 0 0 0 0 0 0 0 0 0 0

0 2 69 0 0 0 0 0 0 0 0 0 0 0

0 2 66 0 0 0 0 0 0 0 0 0 0 0

0 2 72 0 0 0 0 0 0 0 0 0 0 0

0 2 72 0 0 0 0 0 0 0 0 0 0 0

0 2 79 0 0 0 0 0 0 0 0 0 0 0

0 2 82 0 0 0 0 0 0 0 0 0 0 0

0 2 80 0 0 0 0 0 0 0 0 0 0 0

0 2 81 0 0 0 0 0 0 0 0 0 0 0

0 2 64 0 0 0 0 0 0 0 0 0 0 0

0 2 63 0 0 0 0 0 0 0 0 0 0 0

0 2 58 0 0 0 0 0 0 0 0 0 0 0

0 2 89 0 0 0 0 0 0 0 0 0 0 0

0 2 38 0 0 0 0 0 0 0 0 0 0 0

0 2 38 0 0 0 0 0 0 0 0 0 0 0

0 2 40 0 0 0 0 0 0 0 0 0 0 0

0 2 39 0 0 0 0 0 0 0 0 0 0 0

0 2 39 0 0 0 0 0 0 0 0 0 0 0

0 2 39 0 0 0 0 0 0 0 0 0 0 0

0 2 38 0 0 0 0 0 0 0 0 0 0 0

0 2 39 0 0 0 0 0 0 0 0 0 0 0

0 2 39 0 0 0 0 0 0 0 0 0 0 0

0 2 39 0 0 0 0 0 0 0 0 0 0 0

0 2 39 0 0 0 0 0 0 0 0 0 0 0

0 2 39 0 0 0 0 0 0 0 0 0 0 0

0 2 53 0 0 0 0 0 0 0 0 0 0 0

0 2 53 0 0 0 0 0 0 0 0 0 0 0

0 2 55 0 0 0 0 0 0 0 0 0 0 0

0 2 64 0 0 0 0 0 0 0 0 0 0 0

0 2 34 0 0 0 0 0 0 0 0 0 0 0

0 2 54 0 0 0 0 0 0 0 0 0 0 0

0 2 27 0 0 0 0 0 0 0 0 0 0 0

0 2 63 0 0 0 0 0 0 0 0 0 0 0

0 2 51 0 0 0 0 0 0 0 0 0 0 0

0 2 50 0 0 0 0 0 0 0 0 0 0 0

0 2 75 0 0 0 0 0 0 0 0 0 0 0

0 2 57 0 0 0 0 0 0 0 0 0 0 0

0 2 55 0 0 0 0 0 0 0 0 0 0 0

0 2 62 0 0 0 0 0 0 0 0 0 0 0

0 2 51 0 0 0 0 0 0 0 0 0 0 0

0 2 48 0 0 0 0 0 0 0 0 0 0 0

0 2 69 0 0 0 0 0 0 0 0 0 0 0

0 2 34 0 0 0 0 0 0 0 0 0 0 0

0 2 63 0 0 0 0 0 0 0 0 0 0 0

0 2 57 0 0 0 0 0 0 0 0 0 0 0

0 2 59 0 0 0 0 0 0 0 0 0 0 0

0 2 55 0 0 0 0 0 0 0 0 0 0 0

0 2 59 0 0 0 0 0 0 0 0 0 0 0

0 2 55 0 0 0 0 0 0 0 0 0 0 0

0 2 58 0 0 0 0 0 0 0 0 0 0 0

0 2 62 0 0 0 0 0 0 0 0 0 0 0

0 2 57 0 0 0 0 0 0 0 0 0 0 0

0 2 57 0 0 0 0 0 0 0 0 0 0 0

0 2 58 0 0 0 0 0 0 0 0 0 0 0

0 2 57 0 0 0 0 0 0 0 0 0 0 0

0 2 54 0 0 0 0 0 0 0 0 0 0 0

0 2 67 0 0 0 0 0 0 0 0 0 0 0

0 2 58 0 0 0 0 0 0 0 0 0 0 0

0 2 74 0 0 0 0 0 0 0 0 0 0 0

0 2 46 0 0 0 0 0 0 0 0 0 0 0

0 2 34 0 0 0 0 0 0 0 0 0 0 0

0 2 54 0 0 0 0 0 0 0 0 0 0 0

0 2 67 0 0 0 0 0 0 0 0 0 0 0

0 2 61 0 0 0 0 0 0 0 0 0 0 0

0 2 52 0 0 0 0 0 0 0 0 0 0 0

0 2 52 0 0 0 0 0 0 0 0 0 0 0

0 2 55 0 0 0 0 0 0 0 0 0 0 0

0 2 85 0 0 0 0 0 0 0 0 0 0 0

0 2 49 0 0 0 0 0 0 0 0 0 0 0

0 2 66 0 0 0 0 0 0 0 0 0 0 0

0 2 50 0 0 0 0 0 0 0 0 0 0 0

0 2 47 0 0 0 0 0 0 0 0 0 0 0

0 2 67 0 0 0 0 0 0 0 0 0 0 0

0 2 42 0 0 0 0 0 0 0 0 0 0 0

0 2 38 0 0 0 0 0 0 0 0 0 0 0

0 2 51 0 0 0 0 0 0 0 0 0 0 0

0 2 70 0 0 0 0 0 0 0 0 0 0 0

0 2 56 0 0 0 1 0 0 0 0 0 0 0

0 2 50 0 0 0 0 0 0 0 0 0 2 0

0 2 45 0 0 0 1 0 0 0 0 0 3 0

0 2 67 0 0 0 1 0 0 0 0 0 3 0

0 2 34 0 0 0 1 0 0 0 0 0 3 0

0 2 61 0 0 0 1 0 0 0 0 0 3 0

0 2 59 0 0 0 1 0 0 0 0 0 3 0

0 2 58 0 0 0 1 0 0 0 0 0 3 0

0 2 54 0 0 0 1 0 0 0 0 0 3 0

0 2 68 0 0 0 1 0 0 0 0 0 4 0

0 2 82 0 0 0 0 0 0 0 0 0 0 0

0 2 88 0 0 0 0 0 0 0 0 0 0 0

0 2 39 0 0 0 0 0 0 0 0 0 0 0

0 2 58 0 0 0 0 0 0 0 0 0 0 0

0 2 53 0 0 0 0 0 0 0 0 0 0 0

0 2 52 0 0 0 0 0 0 0 0 0 0 0

0 2 52 0 0 0 0 0 0 0 0 0 0 0

0 2 52 0 0 0 0 0 0 0 0 0 0 0

0 2 45 0 0 0 0 0 0 0 0 0 0 0

0 2 67 0 0 0 0 0 0 0 0 0 0 0

0 2 72 0 0 0 0 0 0 0 0 0 0 0

0 2 44 0 0 0 0 0 0 0 0 0 0 0

0 2 69 0 0 0 0 0 0 0 0 0 0 0

0 2 69 0 0 0 0 0 0 0 0 0 0 0

0 2 21 0 0 0 0 0 0 0 0 0 0 0

0 2 47 0 0 0 0 0 0 0 0 0 0 0

0 2 55 0 0 0 0 0 0 0 0 0 0 0

0 2 61 0 0 0 0 0 0 0 0 0 0 0

0 2 42 0 0 0 0 0 0 0 0 0 0 0

0 2 50 0 0 0 0 0 0 0 0 0 0 0

0 2 41 0 0 0 0 0 0 0 0 0 0 0

0 2 54 0 0 0 0 0 0 0 0 0 0 0

0 2 54 0 0 0 0 0 0 0 0 0 0 0

0 2 71 0 0 0 0 0 0 0 0 0 0 0

0 2 56 0 0 0 0 0 0 0 0 0 0 0

0 2 68 0 0 0 0 0 0 0 0 0 0 0

0 2 54 0 0 0 0 0 0 0 0 0 0 0

0 2 56 0 0 0 0 0 0 0 0 0 0 0

0 2 59 0 0 0 0 0 0 0 0 0 0 0

0 2 54 0 0 0 0 0 0 0 0 0 0 0

0 2 64 0 0 0 0 0 0 0 0 0 0 0

0 2 65 0 0 0 0 0 0 0 0 0 0 0

0 2 69 0 0 0 0 0 0 0 0 0 0 0

0 2 40 0 0 0 0 0 0 0 0 0 0 0

0 2 37 0 0 0 0 0 0 0 0 0 0 0

0 2 59 0 0 0 0 0 0 0 0 0 0 0

0 2 62 0 0 0 0 0 0 0 0 0 0 0

0 2 40 0 0 0 0 0 0 0 0 0 0 0

0 2 19 0 0 0 0 0 0 0 0 0 0 0

0 2 50 0 0 0 0 0 0 0 0 0 0 0

0 2 69 0 0 0 0 0 0 0 0 0 0 0

0 2 58 0 0 0 0 0 0 0 0 0 0 0

0 2 60 0 0 0 0 0 0 0 0 0 0 0

0 2 84 0 0 0 0 0 0 0 0 0 0 0

0 2 61 0 0 0 0 0 0 0 0 0 0 0

0 2 43 0 0 0 0 0 0 0 0 0 0 0

0 2 44 0 0 0 0 0 0 0 0 0 0 0

0 2 28 0 0 0 0 0 0 0 0 0 0 0

0 2 27 0 0 0 0 0 0 0 0 0 0 0

0 2 28 0 0 0 0 0 0 0 0 0 0 0

0 2 29 0 0 0 0 0 0 0 0 0 0 0

0 2 34 0 0 0 0 0 0 0 0 0 0 0

0 2 35 0 0 0 0 0 0 0 0 0 0 0

0 2 35 0 0 0 0 0 0 0 0 0 0 0

0 2 41 0 0 0 0 0 0 0 0 0 0 0

0 2 39 0 0 0 0 0 0 0 0 0 0 0

0 2 61 0 0 0 0 0 0 0 0 0 0 0

0 2 51 0 0 0 0 0 0 0 0 0 0 0

0 2 47 0 0 0 0 0 0 0 0 0 0 0

0 2 42 0 0 0 0 0 0 0 0 0 0 0

0 2 16 0 0 0 0 0 0 0 0 0 0 0

0 2 37 0 0 0 0 0 0 0 0 0 0 0

0 2 67 0 0 0 0 0 0 0 0 0 0 0

0 2 46 0 0 0 0 0 0 0 0 0 0 0

0 2 62 0 0 0 0 0 0 0 0 0 0 0

0 2 63 0 0 0 0 0 0 0 0 0 0 0

0 2 27 0 0 0 0 0 0 0 0 0 0 0

0 2 53 0 0 0 0 0 0 0 0 0 0 0

0 2 34 0 0 0 0 0 0 0 0 0 0 0

0 2 56 0 0 0 0 0 0 0 0 0 0 0

0 2 45 0 0 0 0 0 0 0 0 0 0 0

0 2 28 0 0 0 0 0 0 0 0 0 0 0

0 2 41 0 0 0 0 0 0 0 0 0 0 0

0 2 47 0 0 0 0 0 0 0 0 0 0 0

0 2 83 0 0 0 0 0 0 0 0 0 0 0

0 2 74 0 0 0 0 0 0 0 0 0 0 0

0 2 41 0 0 0 0 0 0 0 0 0 0 0

0 2 76 0 0 0 0 0 0 0 0 0 0 0

0 2 52 0 0 0 0 0 0 0 0 0 0 0

0 2 68 0 0 0 0 0 0 0 0 0 0 0

0 2 70 0 0 0 0 0 0 0 0 0 0 0

0 2 57 0 0 0 0 0 0 0 0 0 0 0

0 2 46 0 0 0 0 0 0 0 0 0 0 0

0 2 27 0 0 0 0 0 0 0 0 0 0 0

0 2 45 0 0 0 0 0 0 0 0 0 0 0

0 2 57 0 0 0 0 0 0 0 0 0 0 0

0 2 74 0 0 0 0 0 0 0 0 0 0 0

0 2 43 0 0 0 0 0 0 0 0 0 0 0

0 2 36 0 0 0 0 0 0 0 0 0 0 0

0 2 59 0 0 0 0 0 0 0 0 0 0 0

0 2 58 0 0 0 0 0 0 0 0 0 0 0

0 2 76 0 0 0 0 0 0 0 0 0 0 0

0 2 53 0 0 0 0 0 0 0 0 0 0 0

0 2 51 0 0 0 0 0 0 0 0 0 0 0

0 2 37 0 0 0 0 0 0 0 0 0 0 0

0 2 43 0 0 0 0 0 0 0 0 0 0 0

0 2 38 0 0 0 0 0 0 0 0 0 0 0

0 2 34 0 0 0 0 0 0 0 0 0 0 0

0 2 51 0 0 0 0 0 0 0 0 0 0 0

0 2 49 0 0 0 0 0 0 0 0 0 0 0

0 2 37 0 0 0 0 0 0 0 0 0 0 0

0 2 84 0 0 0 0 0 0 0 0 0 0 0

0 2 37 0 0 0 0 0 0 0 0 0 0 0

0 2 57 0 0 0 0 0 0 0 0 0 0 0

0 2 30 0 0 0 0 0 0 0 0 0 0 0

0 2 59 0 0 0 0 0 0 0 0 0 0 0

0 2 46 0 0 0 0 0 0 0 0 0 0 0

0 2 30 0 0 0 0 0 0 0 0 0 0 0

0 2 40 0 0 0 0 0 0 0 0 0 0 0

0 2 19 0 0 0 0 0 0 0 0 0 0 0

0 2 46 0 0 0 0 0 0 0 0 0 0 0

0 2 21 0 0 0 0 0 0 0 0 0 0 0

0 2 54 0 0 0 0 0 0 0 0 0 0 0

0 2 63 0 0 0 0 0 0 0 0 0 0 0

0 2 40 0 0 0 0 0 0 0 0 0 0 0

0 2 52 0 0 0 0 0 0 0 0 0 0 0

0 2 55 0 0 0 0 0 0 0 0 0 0 0

0 2 36 0 0 0 0 0 0 0 0 0 0 0

0 2 48 0 0 0 0 0 0 0 0 0 0 0

0 2 34 0 0 0 0 0 0 0 0 0 0 0

0 2 54 0 0 0 0 0 0 0 0 0 0 0

0 2 65 0 0 0 0 0 0 0 0 0 0 0

0 2 45 0 0 0 0 0 0 0 0 0 0 0

0 2 38 0 0 0 0 0 0 0 0 0 0 0

0 2 48 0 0 0 0 0 0 0 0 0 0 0

0 2 40 0 0 0 0 0 0 0 0 0 0 0

0 2 50 0 0 0 0 0 0 0 0 0 0 0

0 2 46 0 0 0 0 0 0 0 0 0 0 0

0 2 64 0 0 0 0 0 0 0 0 0 0 0

0 2 32 0 0 0 0 0 0 0 0 0 0 0

0 2 32 0 0 0 0 0 0 0 0 0 0 0

0 2 26 0 0 0 0 0 0 0 0 0 0 0

0 2 40 0 0 0 0 0 0 0 0 0 0 0

0 2 31 0 0 0 0 0 0 0 0 0 0 0

0 2 31 0 0 0 0 0 0 0 0 0 0 0

0 2 59 0 0 0 0 0 0 0 0 0 0 0

0 2 18 0 0 0 0 0 0 0 0 0 0 0

0 2 40 0 0 0 0 0 0 0 0 0 0 0

0 2 49 0 0 0 0 0 0 0 0 0 0 0

0 2 58 0 0 0 0 0 0 0 0 0 0 0

0 2 75 0 0 0 0 0 0 0 0 0 0 0

0 2 39 0 0 0 0 0 0 0 0 0 0 0

0 2 56 0 0 0 0 0 0 0 0 0 0 0

0 2 55 0 0 0 0 0 0 0 0 0 0 0

0 2 34 0 0 0 0 0 0 0 0 0 0 0

0 2 53 0 0 0 0 0 0 0 0 0 0 0

0 2 55 0 0 0 0 0 0 0 0 0 0 0

0 2 39 0 0 0 0 0 0 0 0 0 0 0

0 2 36 0 0 0 0 0 0 0 0 0 0 0

0 2 36 0 0 0 0 0 0 0 0 0 0 0

0 2 40 0 0 0 0 0 0 0 0 0 0 0

0 2 47 0 0 0 0 0 0 0 0 0 0 0

0 2 59 0 0 0 0 0 0 0 0 0 0 0

0 2 39 0 0 0 0 0 0 0 0 0 0 0

0 2 58 0 0 0 0 0 0 0 0 0 0 0

0 2 49 0 0 0 0 0 0 0 0 0 0 0

0 2 37 0 0 0 0 0 0 0 0 0 0 0

0 2 56 0 0 0 0 0 0 0 0 0 0 0

0 2 55 0 0 0 0 0 0 0 0 0 0 0

0 2 11 0 0 0 0 0 0 0 0 0 0 0

0 2 54 0 0 0 0 0 0 0 0 0 0 0

0 2 37 0 0 0 0 0 0 0 0 0 0 0

0 2 45 0 0 0 0 0 0 0 0 0 0 0

0 2 51 0 0 0 0 0 0 0 0 0 0 0

0 2 48 0 0 0 0 0 0 0 0 0 0 0

0 2 54 0 0 0 0 0 0 0 0 0 0 0

0 2 62 0 0 0 0 0 0 0 0 0 0 0

0 2 48 0 0 0 0 0 0 0 0 0 0 0

0 2 47 0 0 0 0 0 0 0 0 0 0 0

0 2 18 0 0 0 0 0 0 0 0 0 0 0

0 2 16 0 0 0 0 0 0 0 0 0 0 0

0 2 16 0 0 0 0 0 0 0 0 0 0 0

0 2 28 0 0 0 0 0 0 0 0 0 0 0

0 2 54 0 0 0 0 0 0 0 0 0 0 0

0 2 27 0 0 0 0 0 0 0 0 0 0 0

0 2 36 0 0 0 0 0 0 0 0 0 0 0

0 2 18 0 0 0 0 0 0 0 0 0 0 0

0 2 44 0 0 0 0 0 0 0 0 0 0 0

0 2 44 0 0 0 0 0 0 0 0 0 0 0

0 2 18 0 0 0 0 0 0 0 0 0 0 0

0 2 14 0 0 0 0 0 0 0 0 0 0 0

0 2 14 0 0 0 0 0 0 0 0 0 0 0

0 2 13 0 0 0 0 0 0 0 0 0 0 0

0 2 34 0 0 0 0 0 0 0 0 0 0 0

0 2 57 0 0 0 0 0 0 0 0 0 0 0

0 2 55 0 0 0 0 0 0 0 0 0 0 0

0 2 38 0 0 0 0 0 0 0 0 0 0 0

0 2 15 0 0 0 0 0 0 0 0 0 0 0

0 2 60 0 0 0 0 0 0 0 0 0 0 0

0 2 13 0 0 0 0 0 0 0 0 0 0 0

0 2 40 0 0 0 0 0 0 0 0 0 0 0

0 2 51 0 0 0 0 0 0 0 0 0 0 0

0 2 51 0 0 0 0 0 0 0 0 0 0 0

0 2 56 0 0 0 0 0 0 0 0 0 0 0

0 2 19 0 0 0 0 0 0 0 0 0 0 0

0 2 42 0 0 0 0 0 0 0 0 0 0 0

0 2 26 0 0 0 0 0 0 0 0 0 0 0

0 2 27 0 0 0 0 0 0 0 0 0 0 0

0 2 16 0 0 0 0 0 0 0 0 0 0 0

0 2 16 0 0 0 0 0 0 0 0 0 0 0

0 2 35 0 0 0 0 0 0 0 0 0 0 0

0 2 52 0 0 0 0 0 0 0 0 0 0 0

0 2 44 0 0 0 0 0 0 0 0 0 0 0

0 2 18 0 0 0 0 0 0 0 0 0 0 0

0 2 44 0 0 0 0 0 0 0 0 0 0 0

0 2 20 0 0 0 0 0 0 0 0 0 0 0

0 2 27 0 0 0 0 0 0 0 0 0 0 0

0 2 51 0 0 0 0 0 0 0 0 0 0 0

0 2 59 0 0 0 0 0 0 0 0 0 0 0

0 2 26 0 0 0 0 0 0 0 0 0 0 0

0 2 17 0 0 0 0 0 0 0 0 0 0 0

0 2 75 0 0 0 0 0 0 0 0 0 0 0

0 2 60 0 0 0 0 0 0 0 0 0 0 0

0 2 57 0 0 0 0 0 0 0 0 0 0 0

0 2 77 0 0 0 1 0 0 0 0 0 0 0

0 2 58 0 0 0 0 0 0 0 0 0 0 0

0 2 53 0 0 0 0 0 0 0 0 0 0 0

0 2 36 0 0 0 0 0 0 0 0 0 0 0

0 2 42 0 0 0 0 0 0 0 0 0 0 0

0 2 46 0 0 0 0 0 0 0 0 0 0 0

0 2 58 0 0 0 0 0 0 0 0 0 0 0

0 2 55 0 0 0 0 0 0 0 0 0 0 0

0 2 57 0 0 0 0 0 0 0 0 0 0 0

0 2 72 0 0 0 0 0 0 0 0 0 0 0

0 2 70 0 0 0 0 0 0 0 0 0 0 0

0 2 44 0 0 0 0 0 0 0 0 0 0 0

0 2 73 0 0 0 0 0 0 0 0 0 0 0

0 2 73 0 0 0 0 0 0 0 0 0 0 0

0 2 48 0 0 0 0 0 0 0 0 0 0 0

0 2 79 0 0 0 0 0 0 0 0 0 0 0

0 2 70 0 0 0 0 0 0 0 0 0 0 0

0 2 91 0 0 0 0 0 0 0 0 0 0 0

0 2 72 0 0 0 0 0 0 0 0 0 0 0

0 2 62 0 0 0 0 0 0 0 0 0 0 0

0 2 72 0 0 0 0 0 0 0 0 0 0 0

0 2 72 0 0 0 0 0 0 0 0 0 0 0

0 2 62 0 0 0 0 0 0 0 0 0 0 0

0 2 61 0 0 0 0 0 0 0 0 0 0 0

0 2 57 0 0 0 0 0 0 0 0 0 0 0

0 2 76 0 0 0 0 0 0 0 0 0 0 0

0 2 55 0 0 0 0 0 0 0 0 0 0 0

0 2 81 0 0 0 0 0 0 0 0 0 0 0

0 2 76 0 0 0 0 0 0 0 0 0 0 0

0 2 74 0 0 0 0 0 0 0 0 0 0 0

0 2 53 0 0 0 0 0 0 0 0 0 0 0

0 2 48 0 0 0 0 0 0 0 0 0 0 0

0 2 72 0 0 0 0 0 0 0 0 0 0 0

0 2 66 0 0 0 0 0 0 0 0 0 0 0

0 2 74 0 0 0 0 0 0 0 0 0 0 0

0 2 57 0 0 0 0 0 0 0 0 0 0 0

0 2 62 0 0 0 0 0 0 0 0 0 0 0

0 2 71 0 0 0 0 0 0 0 0 0 0 0

0 2 57 0 0 0 0 0 0 0 0 0 0 0

0 2 64 0 0 0 0 0 0 0 0 0 0 0

0 2 55 0 0 0 0 0 0 0 0 0 0 0

0 2 54 0 0 0 0 0 0 0 0 0 0 0

0 2 67 0 0 0 0 0 0 0 0 0 0 0

0 2 71 0 0 0 0 0 0 0 0 0 0 0

0 2 48 0 0 0 0 0 0 0 0 0 0 0

0 2 63 0 0 0 0 0 0 0 0 0 0 0

0 2 77 0 0 0 0 0 0 0 0 0 0 0

0 2 80 0 0 0 0 0 0 0 0 0 0 0

0 2 68 0 0 0 0 0 0 0 0 0 0 0

0 2 74 0 0 0 0 0 0 0 0 0 0 0

0 2 61 0 0 0 1 0 0 0 0 0 0 0

0 2 51 0 0 0 1 0 0 0 0 0 0 0

0 2 67 0 0 0 1 0 0 0 0 0 0 0

0 2 61 0 0 0 1 0 0 0 0 0 0 0

0 2 61 0 0 0 1 0 0 0 0 0 0 0

0 2 43 0 0 0 1 0 0 0 0 0 0 0

0 2 72 0 0 0 1 0 0 0 0 0 0 0

0 2 51 0 0 0 1 0 0 0 0 0 0 0

0 2 62 0 0 0 1 0 0 0 0 0 0 0

0 2 51 0 0 0 1 0 0 0 0 0 0 0

0 2 73 0 0 0 1 0 0 0 0 0 0 0

0 2 46 0 0 0 1 0 0 0 0 0 0 0

0 2 49 0 0 0 1 0 0 0 0 0 0 0

0 2 91 0 0 0 1 0 0 0 0 0 0 0

0 2 56 0 0 0 1 0 0 0 0 0 0 0

0 2 80 0 0 0 1 0 0 0 0 0 0 0

0 2 54 0 0 0 1 0 0 0 0 0 4 0

0 2 83 0 0 0 1 0 0 0 0 0 0 0

0 2 69 0 0 0 1 0 0 0 0 0 3 0

0 2 66 0 0 0 1 0 0 0 0 0 4 0

0 2 64 0 0 0 1 0 0 0 0 0 0 0

0 2 77 0 0 0 0 0 0 0 0 0 0 0

0 2 35 0 0 0 0 0 0 0 0 0 0 0

0 2 65 0 0 0 0 0 0 0 0 0 0 0

0 2 57 0 0 0 0 0 0 0 0 0 0 0

0 2 55 0 0 0 0 0 0 0 0 0 0 0

0 2 78 0 0 0 0 0 0 0 0 0 0 0

0 2 80 0 0 0 0 0 0 0 0 0 0 0

0 2 62 0 0 0 0 0 0 0 0 0 0 0

0 2 57 0 0 0 0 0 0 0 0 0 0 0

0 2 64 0 0 0 0 0 0 0 0 0 0 0

0 2 39 0 0 0 0 0 0 0 0 0 0 0

0 2 53 0 0 0 0 0 0 0 0 0 0 0

0 2 65 0 0 0 0 0 0 0 0 0 0 0

0 2 58 0 0 0 0 0 0 0 0 0 0 0

0 2 56 0 0 0 0 0 0 0 0 0 0 0

0 2 44 0 0 0 0 0 0 0 0 0 2 0

0 2 43 0 0 0 0 0 0 0 0 0 3 0

0 2 59 0 0 0 0 0 0 0 0 0 2 0

0 2 80 0 0 0 0 0 0 0 0 0 0 0

0 2 78 0 0 0 0 0 0 0 0 0 0 0

0 2 76 0 0 0 0 0 0 0 0 0 0 0

0 2 66 0 0 0 0 0 0 0 0 0 0 0

0 2 57 0 0 0 0 0 0 0 0 0 0 0

0 2 83 0 0 0 0 0 0 0 0 0 0 0

0 2 44 0 0 0 0 0 0 0 0 0 0 0

0 2 73 0 0 0 0 0 0 0 0 0 0 0

0 2 75 0 0 0 0 0 0 0 0 0 0 0

0 2 61 0 0 0 0 0 0 0 0 0 0 0

0 2 57 0 0 0 0 0 0 0 0 0 0 0

0 2 38 0 0 0 0 0 0 0 0 0 0 0

0 2 76 0 0 0 0 0 0 0 0 0 0 0

0 2 71 0 0 0 0 0 0 0 0 0 0 0

0 2 40 0 0 0 0 0 0 0 0 0 0 0

0 2 65 0 0 0 0 0 0 0 0 0 0 0

0 2 58 0 0 0 0 0 0 0 0 0 0 0

0 2 25 0 0 0 0 0 0 0 0 0 0 0

0 2 61 0 0 0 0 0 0 0 0 0 0 0

0 2 63 0 0 0 0 0 0 0 0 0 0 0

0 2 60 0 0 0 0 0 0 0 0 0 0 0

0 2 67 0 0 0 0 0 0 0 0 0 0 0

0 2 59 0 0 0 0 0 0 0 0 0 0 0

0 2 56 0 0 0 0 0 0 0 0 0 0 0

0 2 57 0 0 0 0 0 0 0 0 0 0 0

0 2 63 0 0 0 0 0 0 0 0 0 0 0

0 2 92 0 0 0 0 0 0 0 0 0 0 0

0 2 85 0 0 0 0 0 0 0 0 0 0 0

0 2 54 0 0 0 0 0 0 0 0 0 0 0

0 2 47 0 0 0 0 0 0 0 0 0 0 0

0 2 74 0 0 0 0 0 0 0 0 0 0 0

0 2 66 0 0 0 0 0 0 0 0 0 0 0

0 2 56 0 0 0 0 0 0 0 0 0 0 0

0 2 58 0 0 0 0 0 0 0 0 0 0 0

0 2 58 0 0 0 0 0 0 0 0 0 0 0

0 2 57 0 0 0 0 0 0 0 0 0 0 0

0 2 58 0 0 0 0 0 0 0 0 0 0 0

0 2 60 0 0 0 0 0 0 0 0 0 0 0

0 2 39 0 0 0 0 0 0 0 0 0 0 0

0 2 55 0 0 0 0 0 0 0 0 0 0 0

0 2 75 0 0 0 0 0 0 0 0 0 0 0

0 2 46 0 0 0 0 0 0 0 0 0 0 0

0 2 53 0 0 0 0 0 0 0 0 0 0 0

0 2 55 0 0 0 0 0 0 0 0 0 0 0

0 2 74 0 0 0 0 0 0 0 0 0 0 0

0 2 58 0 0 0 0 0 0 0 0 0 0 0

0 2 75 0 0 0 0 0 0 0 0 0 0 0

0 2 57 0 0 0 0 0 0 0 0 0 0 0

0 2 48 0 0 0 0 0 0 0 0 0 0 0

0 2 48 0 0 0 0 0 0 0 0 0 0 0

0 2 60 0 0 0 0 0 0 0 0 0 0 0

0 2 55 0 0 0 0 0 0 0 0 0 0 0

0 2 52 0 0 0 0 0 0 0 0 0 0 0

0 2 53 0 0 0 0 0 0 0 0 0 0 0

0 2 49 0 0 0 0 0 0 0 0 0 0 0

0 2 46 0 0 0 0 0 0 0 0 0 0 0

0 2 39 0 0 0 0 0 0 0 0 0 0 0

0 2 64 0 0 0 0 0 0 0 0 0 0 0

0 2 63 0 0 0 0 0 0 0 0 0 0 0

0 2 54 0 0 0 0 0 0 0 0 0 0 0

0 2 47 0 0 0 0 0 0 0 0 0 0 0

0 2 69 0 0 0 0 0 0 0 0 0 0 0

0 2 53 0 0 0 0 0 0 0 0 0 0 0

0 2 53 0 0 0 0 0 0 0 0 0 0 0

0 2 53 0 0 0 0 0 0 0 0 0 0 0

0 2 59 0 0 0 0 0 0 0 0 0 0 0

0 2 56 0 0 0 0 0 0 0 0 0 0 0

0 2 58 0 0 0 0 0 0 0 0 0 0 0

0 2 55 0 0 0 0 0 0 0 0 0 0 0

0 2 56 0 0 0 0 0 0 0 0 0 0 0

0 2 60 0 0 0 0 0 0 0 0 0 0 0

0 2 77 0 0 0 0 0 0 0 0 0 0 0

0 2 61 0 0 0 0 0 0 0 0 0 0 0

0 2 75 0 0 0 0 0 0 0 0 0 0 0

0 2 77 0 0 0 0 0 0 0 0 0 0 0

0 2 33 0 0 0 0 0 0 0 0 0 0 0

0 2 40 0 0 0 0 0 0 0 0 0 0 0

0 2 36 0 0 0 0 0 0 0 0 0 0 0

0 2 54 0 0 0 0 0 0 0 0 0 0 0

0 2 74 0 0 0 0 0 0 0 0 0 0 0

0 2 58 0 0 0 0 0 0 0 0 0 0 0

0 2 62 0 0 0 0 0 0 0 0 0 0 0

0 2 71 0 0 0 0 0 0 0 0 0 0 0

0 2 79 0 0 0 0 0 0 0 0 0 0 0

0 2 53 0 0 0 0 0 0 0 0 0 0 0

0 2 43 0 0 0 0 0 0 0 0 0 0 0

0 2 41 0 0 0 0 0 0 0 0 0 0 0

0 2 57 0 0 0 0 0 0 0 0 0 0 0

0 2 73 0 0 0 0 0 0 0 0 0 0 0

0 2 69 0 0 0 0 0 0 0 0 0 0 0

0 2 49 0 0 0 0 0 0 0 0 0 0 0

0 2 51 0 0 0 0 0 0 0 0 0 0 0

0 2 65 0 0 0 0 0 0 0 0 0 0 0

0 2 41 0 0 0 0 0 0 0 0 0 0 0

0 2 55 0 0 0 0 0 0 0 0 0 0 0

0 2 85 0 0 0 0 0 0 0 0 0 0 0

0 2 74 0 0 0 0 0 0 0 0 0 0 0

0 2 54 0 0 0 0 0 0 0 0 0 0 0

0 2 69 0 0 0 0 0 0 0 0 0 0 0

0 2 76 0 0 0 0 0 0 0 0 0 0 0

0 2 48 0 0 0 0 0 0 0 0 0 0 0

0 2 82 0 0 0 0 0 0 0 0 0 0 0

0 2 71 0 0 0 0 0 0 0 0 0 0 0

0 2 80 0 0 0 0 0 0 0 0 0 0 0

0 2 70 0 0 0 0 0 0 0 0 0 0 0

0 2 75 0 0 0 0 0 0 0 0 0 0 0

0 2 65 0 0 0 0 0 0 0 0 0 0 0

0 2 83 0 0 0 0 0 0 0 0 0 0 0

0 2 54 0 0 0 0 0 0 0 0 0 0 0

0 2 64 0 0 0 0 0 0 0 0 0 0 0

0 2 75 0 0 0 0 0 0 0 0 0 0 0

0 2 76 0 0 0 0 0 0 0 0 0 0 0

0 2 72 0 0 0 0 0 0 0 0 0 0 0

0 2 46 0 0 0 0 0 0 0 0 0 0 0

0 2 74 0 0 0 0 0 0 0 0 0 0 0

0 2 56 0 0 0 0 0 0 0 0 0 0 0

0 2 56 0 0 0 0 0 0 0 0 0 0 0

0 2 68 0 0 0 0 0 0 0 0 0 0 0

0 2 78 0 0 0 0 0 0 0 0 0 0 0

0 2 65 0 0 0 0 0 0 0 0 0 0 0

0 2 81 0 0 0 0 0 0 0 0 0 0 0

0 2 76 0 0 0 0 0 0 0 0 0 0 0

0 2 65 0 0 0 0 0 0 0 0 0 0 0

0 2 60 0 0 0 0 0 0 0 0 0 0 0

0 2 77 0 0 0 0 0 0 0 0 0 0 0

0 2 87 0 0 0 0 0 0 0 0 0 0 0

0 2 63 0 0 0 0 0 0 0 0 0 0 0

0 2 63 0 0 0 0 0 0 0 0 0 0 0

0 2 61 0 0 0 0 0 0 0 0 0 0 0

0 2 57 0 0 0 0 0 0 0 0 0 0 0

0 2 80 0 0 0 0 0 0 0 0 0 0 0

0 2 75 0 0 0 0 0 0 0 0 0 0 0

0 2 78 0 0 0 0 0 0 0 0 0 0 0

0 2 60 0 0 0 0 0 0 0 0 0 0 0

0 2 62 0 0 0 0 0 0 0 0 0 0 0

0 2 61 0 0 0 0 0 0 0 0 0 0 0

0 2 77 0 0 0 0 0 0 0 0 0 0 0

0 2 75 0 0 0 0 0 0 0 0 0 0 0

0 2 74 0 0 0 0 0 0 0 0 0 0 0

0 2 60 0 0 0 0 0 0 0 0 0 0 0

0 2 73 0 0 0 0 0 0 0 0 0 0 0

0 2 74 0 0 0 0 0 0 0 0 0 0 0

0 2 70 0 0 0 0 0 0 0 0 0 0 0

0 2 62 0 0 0 0 0 0 0 0 0 0 0

0 2 57 0 0 0 0 0 0 0 0 0 0 0

0 2 74 0 0 0 0 0 0 0 0 0 0 0

0 2 80 0 0 0 0 0 0 0 0 0 0 0

0 2 62 0 0 0 0 0 0 0 0 0 0 0

0 2 76 0 0 0 0 0 0 0 0 0 0 0

0 2 63 0 0 0 0 0 0 0 0 0 0 0

0 2 59 0 0 0 0 0 0 0 0 0 0 0

0 2 81 0 0 0 0 0 0 0 0 0 0 0

0 2 82 0 0 0 0 0 0 0 0 0 0 0

0 2 41 0 0 0 0 0 0 0 0 0 0 0

0 2 52 0 0 0 0 0 0 0 0 0 0 0

0 2 85 0 0 0 1 0 0 0 0 0 0 0

0 2 49 0 0 0 1 0 0 0 0 0 0 0

0 2 61 0 0 0 1 0 0 0 0 0 0 0

0 2 78 0 0 0 1 0 0 0 0 0 0 0

0 2 75 0 0 0 1 0 0 0 0 0 0 0

0 2 58 0 0 0 1 0 0 0 0 0 0 0

0 2 56 0 0 0 1 0 0 0 0 0 0 0

0 2 49 0 0 0 1 0 0 0 0 0 0 0

0 2 60 0 0 0 1 0 0 0 0 0 0 0

0 2 73 0 0 0 1 0 0 0 0 0 0 0

0 2 59 0 0 0 1 0 0 0 0 0 1 0

0 2 59 0 0 0 1 0 0 0 0 0 0 0

0 2 44 0 0 0 1 0 0 0 0 0 0 0

0 2 61 0 0 0 1 0 0 0 0 0 0 0

0 2 59 0 0 0 1 0 0 0 0 0 0 0

0 2 68 0 0 0 1 0 0 0 0 0 0 0

0 2 32 0 0 0 1 0 0 0 0 0 0 0

0 2 38 0 0 0 1 0 0 0 0 0 0 0

0 2 80 0 0 0 1 0 0 0 0 0 0 0

0 2 50 0 0 0 1 0 0 0 0 0 3 0

0 2 71 0 0 0 1 0 0 0 0 0 0 0

0 2 78 0 0 0 1 0 0 0 0 0 1 0

0 2 80 0 0 0 1 0 0 0 0 0 2 0

0 2 60 0 0 0 1 0 0 0 0 0 0 0

0 2 63 0 0 0 1 0 0 0 0 0 0 0

0 2 72 0 0 0 1 0 0 0 0 0 0 0

0 2 48 0 0 0 1 0 0 0 0 0 0 0

0 2 84 0 0 0 1 0 0 0 0 0 0 0

0 2 45 0 0 0 1 0 0 0 0 0 3 0

0 2 64 0 0 0 1 0 0 0 0 0 0 0

0 2 56 0 0 0 1 0 0 0 0 0 0 0

0 2 69 0 0 0 1 0 0 0 0 0 0 0

0 2 53 0 0 0 1 0 0 0 0 0 0 0

0 2 88 0 0 0 0 0 0 0 0 0 0 0

0 2 53 0 0 0 0 0 0 0 0 0 0 0

0 2 63 0 0 0 0 0 0 0 0 0 0 0

0 2 66 0 0 0 0 0 0 0 0 0 0 0

0 2 49 0 0 0 0 0 0 0 0 0 0 0

0 2 61 0 0 0 0 0 0 0 0 0 0 0

0 2 54 0 0 0 0 0 0 0 0 0 0 0

0 2 53 0 0 0 0 0 0 0 0 0 0 0

0 2 58 0 0 0 0 0 0 0 0 0 0 0

0 2 63 0 0 0 0 0 0 0 0 0 0 0

0 2 68 0 0 0 0 0 0 0 0 0 0 0

0 2 62 0 0 0 0 0 0 0 0 0 0 0

0 2 72 0 0 0 0 0 0 0 0 0 0 0

0 2 69 0 0 0 0 0 0 0 0 0 0 0

0 2 69 0 0 0 0 0 0 0 0 0 0 0

0 2 69 0 0 0 0 0 0 0 0 0 0 0

0 2 48 0 0 0 0 0 0 0 0 0 0 0

0 2 56 0 0 0 0 0 0 0 0 0 0 0

0 2 54 0 0 0 0 0 0 0 0 0 0 0

0 2 49 0 0 0 0 0 0 0 0 0 0 0

0 2 53 0 0 0 0 0 0 0 0 0 0 0

0 2 48 0 0 0 0 0 0 0 0 0 0 0

0 2 61 0 0 0 0 0 0 0 0 0 0 0

0 2 37 0 0 0 0 0 0 0 0 0 0 0

0 2 57 0 0 0 0 0 0 0 0 0 0 0

0 2 53 0 0 0 0 0 0 0 0 0 0 0

0 2 87 0 0 0 0 0 0 0 0 0 0 0

0 2 43 0 0 0 0 0 0 0 0 0 0 0

0 2 45 0 0 0 0 0 0 0 0 0 0 0

0 2 68 0 0 0 0 0 0 0 0 0 0 0

0 2 51 0 0 0 0 0 0 0 0 0 0 0

0 2 75 0 0 0 0 0 0 0 0 0 0 0

0 2 77 0 0 0 0 0 0 0 0 0 0 0

0 2 77 0 0 0 0 0 0 0 0 0 0 0

0 2 75 0 0 0 0 0 0 0 0 0 0 0

0 2 70 0 0 0 0 0 0 0 0 0 0 0

0 2 48 0 0 0 0 0 0 0 0 0 0 0

0 2 46 0 0 0 0 0 0 0 0 0 0 0

0 2 71 0 0 0 0 0 0 0 0 0 0 0

0 2 42 0 0 0 0 0 0 0 0 0 0 0

0 2 45 0 0 0 0 0 0 0 0 0 0 0

0 2 73 0 0 0 0 0 0 0 0 0 0 0

0 2 75 0 0 0 0 0 0 0 0 0 0 0

0 2 83 0 0 0 0 0 0 0 0 0 3 0

0 2 72 0 0 0 0 0 0 0 0 0 0 0

0 2 64 0 0 0 0 0 0 0 0 0 0 0

0 2 76 0 0 0 0 0 0 0 0 0 0 0

0 2 63 0 0 0 0 0 0 0 0 0 0 0

0 2 59 0 0 0 0 0 0 0 0 0 0 0

0 2 49 0 0 0 0 0 0 0 0 0 0 0

0 2 59 0 0 0 0 0 0 0 0 0 0 0

0 2 58 0 0 0 0 0 0 0 0 0 0 0

0 2 65 0 0 0 0 0 0 0 0 0 0 0

0 2 65 0 0 0 0 0 0 0 0 0 0 0

0 2 53 0 0 0 0 0 0 0 0 0 0 0

0 2 65 0 0 0 0 0 0 0 0 0 0 0

0 2 36 0 0 0 0 0 0 0 0 0 0 0

0 2 59 0 0 0 0 0 0 0 0 0 0 0

0 2 75 0 0 0 0 0 0 0 0 0 0 0

0 2 68 0 0 0 0 0 0 0 0 0 0 0

0 2 46 0 0 0 0 0 0 0 0 0 0 0

0 2 62 0 0 0 0 0 0 0 0 0 0 0

0 2 57 0 0 0 0 0 0 0 0 0 3 0

0 2 66 0 0 0 0 0 0 0 0 0 0 0

0 2 59 0 0 0 0 0 0 0 0 0 4 0

0 2 72 0 0 0 0 0 0 0 0 0 0 0

0 2 80 0 0 0 0 0 0 0 0 0 0 0

0 2 84 0 0 0 0 0 0 0 0 0 0 0

0 2 74 0 0 0 0 0 0 0 0 0 0 0

0 2 54 0 0 0 0 0 0 0 0 0 0 0

0 2 81 0 0 0 0 0 0 0 0 0 0 0

0 2 83 0 0 0 0 0 0 0 0 0 0 0

0 2 74 0 0 0 0 0 0 0 0 0 0 0

0 2 48 0 0 0 0 0 0 0 0 0 0 0

0 2 55 0 0 0 0 0 0 0 0 0 0 0

0 2 43 0 0 0 0 0 0 0 0 0 0 0

0 2 62 0 0 0 0 0 0 0 0 0 0 0

0 2 76 0 0 0 0 0 0 0 0 0 0 0

0 2 51 0 0 0 0 0 0 0 0 0 0 0

0 2 82 0 0 0 0 0 0 0 0 0 0 0

0 2 83 0 0 0 0 0 0 0 0 0 0 0

0 2 75 0 0 0 0 0 0 0 0 0 3 0

0 2 44 0 0 0 0 0 0 0 0 0 0 0

0 2 61 0 0 0 0 0 0 0 0 0 0 0

0 2 68 0 0 0 0 0 0 0 0 0 0 0

0 2 56 0 0 0 0 0 0 0 0 0 0 0

0 2 61 0 0 0 0 0 0 0 0 0 0 0

0 2 67 0 0 0 0 0 0 0 0 0 0 0

0 2 61 0 0 0 0 0 0 0 0 0 0 0

0 2 79 0 0 0 0 0 0 0 0 0 0 0

0 2 77 0 0 0 0 0 0 0 0 0 0 0

0 2 61 0 0 0 0 0 0 0 0 0 0 0

0 2 60 0 0 0 0 0 0 0 0 0 0 0

0 2 60 0 0 0 0 0 0 0 0 0 0 0

0 2 64 0 0 0 0 0 0 0 0 0 0 0

0 2 76 0 0 0 0 0 0 0 0 0 0 0

0 2 61 0 0 0 0 0 0 0 0 0 2 0

0 2 45 0 0 0 0 0 0 0 0 0 0 0

0 2 61 0 0 0 0 0 0 0 0 0 0 0

0 2 42 0 0 0 0 0 0 0 0 0 2 0

0 2 46 0 0 0 0 0 0 0 0 0 0 0

0 2 59 0 0 0 0 0 0 0 0 0 0 0

0 2 56 0 0 0 0 0 0 0 0 0 0 0

0 2 46 0 0 0 0 0 0 0 0 0 0 0

0 2 61 0 0 0 0 0 0 0 0 0 0 0

0 2 40 0 0 0 0 0 0 0 0 0 0 0

0 2 49 0 0 0 0 0 0 0 0 0 0 0

0 2 49 0 0 0 0 0 0 0 0 0 0 0

0 2 64 0 0 0 0 0 0 0 0 0 0 0

0 2 66 0 0 0 0 0 0 0 0 0 0 0

0 2 68 0 0 0 0 0 0 0 0 0 0 0

0 2 34 0 0 0 0 0 0 0 0 0 0 0

0 2 79 0 0 0 0 0 0 0 0 0 0 0

0 2 61 0 0 0 0 0 0 0 0 0 0 0

0 2 68 0 0 0 0 0 0 0 0 0 0 0

0 2 64 0 0 0 0 0 0 0 0 0 0 0

0 2 55 0 0 0 0 0 0 0 0 0 0 0

0 2 54 0 0 0 0 0 0 0 0 0 0 0

0 2 75 0 0 0 0 0 0 0 0 0 0 0

0 2 66 0 0 0 0 0 0 0 0 0 0 0

0 2 73 0 0 0 0 0 0 0 0 0 0 0

0 2 83 0 0 0 0 0 0 0 0 0 0 0

0 2 65 0 0 0 0 0 0 0 0 0 0 0

0 2 85 0 0 0 0 0 0 0 0 0 0 0

0 2 77 0 0 0 0 0 0 0 0 0 0 0

0 2 72 0 0 0 0 0 0 0 0 0 0 0

0 2 59 0 0 0 0 0 0 0 0 0 0 0

0 2 78 0 0 0 0 0 0 0 0 0 0 0

0 2 60 0 0 0 0 0 0 0 0 0 0 0

0 2 43 0 0 0 0 0 0 0 0 0 0 0

0 2 67 0 0 0 0 0 0 0 0 0 0 0

0 2 60 0 0 0 0 0 0 0 0 0 0 0

0 2 75 0 0 0 0 0 0 0 0 0 0 0

0 2 72 0 0 0 0 0 0 0 0 0 0 0

0 2 65 0 0 0 0 0 0 0 0 0 0 0

0 2 64 0 0 0 0 0 0 0 0 0 0 0

0 2 81 0 0 0 0 0 0 0 0 0 0 0

0 2 69 0 0 0 0 0 0 0 0 0 0 0

0 2 66 0 0 0 0 0 0 0 0 0 0 0

0 2 74 0 0 0 0 0 0 0 0 0 0 0

0 2 75 0 0 0 0 0 0 0 0 0 0 0

0 2 67 0 0 0 0 0 0 0 0 0 0 0

0 2 74 0 0 0 0 0 0 0 0 0 0 0

0 2 58 0 0 0 0 0 0 0 0 0 0 0

0 2 56 0 0 0 0 0 0 0 0 0 0 0

0 2 71 0 0 0 0 0 0 0 0 0 0 0

0 2 53 0 0 0 0 0 0 0 0 0 0 0

0 2 60 0 0 0 0 0 0 0 0 0 0 0

0 2 53 0 0 0 0 0 0 0 0 0 0 0

0 2 57 0 0 0 0 0 0 0 0 0 0 0

0 2 75 0 0 0 0 0 0 0 0 0 0 0

0 2 80 0 0 0 0 0 0 0 0 0 0 0

0 2 44 0 0 0 0 0 0 0 0 0 0 0

0 2 73 0 0 0 0 0 0 0 0 0 0 0

0 2 59 0 0 0 0 0 0 0 0 0 0 0

0 2 45 0 0 0 0 0 0 0 0 0 0 0

0 2 86 0 0 0 0 0 0 0 0 0 0 0

0 2 77 0 0 0 0 0 0 0 0 0 0 0

0 2 79 0 0 0 0 0 0 0 0 0 4 0

0 2 36 0 0 0 0 0 0 0 0 0 0 0

0 2 61 0 0 0 0 0 0 0 0 0 0 0

0 2 78 0 0 0 0 0 0 0 0 0 0 0

0 2 55 0 0 0 0 0 0 0 0 0 0 0

0 2 48 0 0 0 0 0 0 0 0 0 0 0

0 2 49 0 0 0 0 0 0 0 0 0 0 0

0 2 49 0 0 0 0 0 0 0 0 0 0 0

0 2 48 0 0 0 0 0 0 0 0 0 0 0

0 2 48 0 0 0 0 0 0 0 0 0 0 0

0 2 47 0 0 0 0 0 0 0 0 0 0 0

0 2 58 0 0 0 0 0 0 0 0 0 0 0

0 2 76 0 0 0 0 0 0 0 0 0 0 0

0 2 75 0 0 0 0 0 0 0 0 0 0 0

0 2 67 0 0 0 0 0 0 0 0 0 2 0

0 2 65 0 0 0 0 0 0 0 0 0 0 0

0 2 69 0 0 0 0 0 0 0 0 0 0 0

0 2 76 0 0 0 0 0 0 0 0 0 0 0

0 2 75 0 0 0 0 0 0 0 0 0 0 0

0 2 46 0 0 0 0 0 0 0 0 0 0 0

0 2 56 0 0 0 0 0 0 0 0 0 0 0

0 2 43 0 0 0 0 0 0 0 0 0 0 0

0 2 52 0 0 0 0 0 0 0 0 0 0 0

0 2 75 0 0 0 0 0 0 0 0 0 0 0

0 2 64 0 0 0 0 0 0 0 0 0 0 0

0 2 85 0 0 0 0 0 0 0 0 0 0 0

0 2 51 0 0 0 0 0 0 0 0 0 0 0

0 2 67 0 0 0 0 0 0 0 0 0 0 0

0 2 65 0 0 0 0 0 0 0 0 0 0 0

0 2 64 0 0 0 0 0 0 0 0 0 0 0

0 2 50 0 0 0 0 0 0 0 0 0 0 0

0 2 53 0 0 0 0 0 0 0 0 0 0 0

0 2 59 0 0 0 0 0 0 0 0 0 0 0

0 2 27 0 0 0 0 0 0 0 0 0 0 0

0 2 55 0 0 0 0 0 0 0 0 0 0 0

0 2 65 0 0 0 0 0 0 0 0 0 0 0

0 2 57 0 0 0 0 0 0 0 0 0 4 0

0 2 61 0 0 0 0 0 0 0 0 0 0 0

0 2 76 0 0 0 0 0 0 0 0 0 0 0

0 2 70 0 0 0 0 0 0 0 0 0 3 0

0 2 53 0 0 0 0 0 0 0 0 0 0 0

0 2 51 0 0 0 0 0 0 0 0 0 0 0

0 2 51 0 0 0 0 0 0 0 0 0 0 0

0 2 51 0 0 0 0 0 0 0 0 0 0 0

0 2 73 0 0 0 0 0 0 0 0 0 0 0

0 2 52 0 0 0 0 0 0 0 0 0 0 0

0 2 43 0 0 0 0 0 0 0 0 0 0 0

0 2 59 0 0 0 0 0 0 0 0 0 0 0

0 2 59 0 0 0 0 0 0 0 0 0 0 0

0 2 55 0 0 0 0 0 0 0 0 0 0 0

0 2 50 0 0 0 0 0 0 0 0 0 0 0

0 2 59 0 0 0 0 0 0 0 0 0 0 0

0 2 78 0 0 0 0 0 0 0 0 0 3 0

0 2 53 0 0 0 0 0 0 0 0 0 3 0

0 2 50 0 0 0 0 0 0 0 0 0 3 0

0 2 47 0 0 0 0 0 0 0 0 0 3 0

0 2 53 0 0 0 0 0 0 0 0 0 3 0

0 2 53 0 0 0 0 0 0 0 0 0 3 0

0 2 71 0 0 0 0 0 0 0 0 0 3 0

0 2 49 0 0 0 0 0 0 0 0 0 3 0

0 2 70 0 0 0 0 0 0 0 0 0 3 0

0 2 50 0 0 0 0 0 0 0 0 0 3 0

0 2 84 0 0 0 0 0 0 0 0 0 3 0

0 2 55 0 0 0 0 0 0 0 0 0 3 0

0 2 64 0 0 0 0 0 0 0 0 0 3 0

0 2 85 0 0 0 0 0 0 0 0 0 3 0

0 2 57 0 0 0 0 0 0 0 0 0 3 0

0 2 68 0 0 0 0 0 0 0 0 0 3 0

0 2 66 0 0 0 0 0 0 0 0 0 3 0

0 2 78 0 0 0 0 0 0 0 0 0 3 0

0 2 63 0 0 0 0 0 0 0 0 0 3 0

0 2 62 0 0 0 0 0 0 0 0 0 3 0

0 2 79 0 0 0 0 0 0 0 0 0 3 0

0 2 86 0 0 0 0 0 0 0 0 0 3 0

0 2 84 0 0 0 0 0 0 0 0 0 3 0

0 2 55 0 0 0 0 0 0 0 0 0 3 0

0 2 45 0 0 0 1 0 0 0 0 0 3 0

0 2 46 0 0 0 1 0 0 0 0 0 3 0

0 2 59 0 0 0 1 0 0 0 0 0 3 0

0 2 64 0 0 0 1 0 0 0 0 0 3 0

0 2 59 0 0 0 1 0 0 0 0 0 3 0

0 2 65 0 0 0 1 0 0 0 0 0 3 0

0 2 74 0 0 0 1 0 0 0 0 0 4 0

0 2 52 0 0 0 1 0 0 0 0 0 4 0

0 2 55 0 0 0 1 0 0 0 0 0 4 0

0 2 66 0 0 0 1 0 0 0 0 0 4 0

0 2 65 0 0 0 0 0 0 0 0 0 4 0

0 2 48 0 0 0 0 0 0 0 0 0 4 0

0 2 76 0 0 0 0 0 0 0 0 0 4 0

0 2 55 0 0 0 0 0 0 0 0 0 4 0

0 2 52 0 0 0 0 0 0 0 0 0 4 0

0 2 55 0 0 0 0 0 0 0 0 0 4 0

0 2 63 0 0 0 0 0 0 0 0 0 4 0

0 2 40 0 0 0 0 0 0 0 0 0 4 0

0 2 69 0 0 0 0 0 0 0 0 0 4 0

0 2 71 0 0 0 0 0 0 0 0 0 4 0

0 2 71 0 0 0 0 0 0 0 0 0 4 0

0 2 71 0 0 0 0 0 0 0 0 0 4 0

0 2 67 0 0 0 0 0 0 0 0 0 4 0

0 2 65 0 0 0 0 0 0 0 0 0 4 0

0 2 65 0 0 0 0 0 0 0 0 0 4 0

0 2 84 0 0 0 0 0 0 0 0 0 4 0

0 2 52 0 0 0 0 0 0 0 0 0 4 0

0 2 50 0 0 0 0 0 0 0 0 0 4 0

0 2 64 0 0 0 0 0 0 0 0 0 4 0

0 2 57 0 0 0 0 0 0 0 0 0 4 0

0 2 58 0 0 0 0 0 0 0 0 0 4 0

0 2 40 0 0 0 0 0 0 0 0 0 4 0

0 2 50 0 0 0 0 0 0 0 0 0 4 0

0 2 68 0 0 0 0 0 0 0 0 0 4 0

0 2 50 0 0 0 0 0 0 0 0 0 4 0

0 2 48 0 0 0 0 0 0 0 0 0 4 0

0 2 46 0 0 0 0 0 0 0 0 0 4 0

0 2 63 0 0 0 0 0 0 0 0 0 0 0

0 2 79 0 0 0 0 0 0 0 0 0 0 0

0 2 57 0 0 0 0 0 0 0 0 0 0 0

0 2 73 0 0 0 0 0 0 0 0 0 0 0

0 2 69 0 0 0 0 0 0 0 0 0 0 0

0 2 64 0 0 0 0 0 0 0 0 0 0 0

0 2 49 0 0 0 0 0 0 0 0 0 0 0

0 2 73 0 0 0 0 0 0 0 0 0 2 0

0 2 62 0 0 0 0 0 0 0 0 0 0 0

0 2 56 0 0 0 0 0 0 0 0 0 0 0

0 2 56 0 0 0 0 0 0 0 0 0 0 0

0 2 68 0 0 0 0 0 0 0 0 0 0 0

0 2 83 0 0 0 0 0 0 0 0 0 0 0

0 2 85 0 0 0 0 0 0 0 0 0 0 0

0 2 60 0 0 0 0 0 0 0 0 0 0 0

0 2 56 0 0 0 0 0 0 0 0 0 0 0

0 2 89 0 0 0 0 0 0 0 0 0 0 0

0 2 71 0 0 0 0 0 0 0 0 0 0 0

0 2 62 0 0 0 0 0 0 0 0 0 0 0

0 2 68 0 0 0 0 0 0 0 0 0 0 0

0 2 53 0 0 0 0 0 0 0 0 0 0 0

0 2 86 0 0 0 0 0 0 0 0 0 0 0

0 2 66 0 0 0 0 0 0 0 0 0 0 0

0 2 61 0 0 0 0 0 0 0 0 0 0 0

0 2 43 0 0 0 0 0 0 0 0 0 0 0

0 2 43 0 0 0 0 0 0 0 0 0 0 0

0 2 59 0 0 0 0 0 0 0 0 0 0 0

0 2 46 0 0 0 0 0 0 0 0 0 0 0

0 2 60 0 0 0 0 0 0 0 0 0 0 0

0 2 60 0 0 0 0 0 0 0 0 0 0 0

0 2 68 0 0 0 0 0 0 0 0 0 1 0

0 2 75 0 0 0 0 0 0 0 0 0 0 0

0 2 75 0 0 0 0 0 0 0 0 0 0 0

0 2 76 0 0 0 0 0 0 0 0 0 0 0

0 2 66 0 0 0 0 0 0 0 0 0 0 0

0 2 75 0 0 0 0 0 0 0 0 0 0 0

0 2 78 0 0 0 0 0 0 0 0 0 0 0

0 2 65 0 0 0 0 0 0 0 0 0 0 0

0 2 57 0 0 0 0 0 0 0 0 0 0 0

0 2 58 0 0 0 0 0 0 0 0 0 0 0

0 2 80 0 0 0 0 0 0 0 0 0 0 0

0 2 75 0 0 0 0 0 0 0 0 0 0 0

0 2 44 0 0 0 0 0 0 0 0 0 0 0

0 2 65 0 0 0 0 0 0 0 0 0 0 0

0 2 77 0 0 0 0 0 0 0 0 0 0 0

0 2 66 0 0 0 0 0 0 0 0 0 0 0

0 2 78 0 0 0 0 0 0 0 0 0 0 0

0 2 75 0 0 0 0 0 0 0 0 0 0 0

0 2 82 0 0 0 0 0 0 0 0 0 0 0

0 2 70 0 0 0 0 0 0 0 0 0 0 0

0 2 80 0 0 0 0 0 0 0 0 0 0 0

0 2 66 0 0 0 0 0 0 0 0 0 0 0

0 2 62 0 0 0 0 0 0 0 0 0 0 0

0 2 47 0 0 0 0 0 0 0 0 0 0 0

0 2 67 0 0 0 0 0 0 0 0 0 0 0

0 2 53 0 0 0 0 0 0 0 0 0 0 0

0 2 82 0 0 0 0 0 0 0 0 0 0 0

0 2 62 0 0 0 0 0 0 0 0 0 0 0

0 2 89 0 0 0 0 0 0 0 0 0 0 0

0 2 84 0 0 0 0 0 0 0 0 0 0 0

0 2 74 0 0 0 0 0 0 0 0 0 0 0

0 2 60 0 0 0 0 0 0 0 0 0 0 0

0 2 54 0 0 0 0 0 0 0 0 0 0 0

0 2 60 0 0 0 0 0 0 0 0 0 0 0

0 2 52 0 0 0 0 0 0 0 0 0 0 0

0 2 58 0 0 0 0 0 0 0 0 0 0 0

0 2 64 0 0 0 0 0 0 0 0 0 0 0

0 2 53 0 0 0 0 0 0 0 0 0 0 0

0 2 54 0 0 0 0 0 0 0 0 0 0 0

0 2 27 0 0 0 0 0 0 0 0 0 0 0

0 2 84 0 0 0 0 0 0 0 0 0 0 0

0 2 54 0 0 0 0 0 0 0 0 0 0 0

0 2 74 0 0 0 0 0 0 0 0 0 0 0

0 2 74 0 0 0 0 0 0 0 0 0 0 0

0 2 65 0 0 0 0 0 0 0 0 0 0 0

0 2 51 0 0 0 0 0 0 0 0 0 0 0

0 2 53 0 0 0 0 0 0 0 0 0 0 0

0 2 72 0 0 0 0 0 0 0 0 0 0 0

0 2 64 0 0 0 0 0 0 0 0 0 0 0

0 2 77 0 0 0 0 0 0 0 0 0 0 0

0 2 72 0 0 0 0 0 0 0 0 0 0 0

0 2 54 0 0 0 0 0 0 0 0 0 0 0

0 2 46 0 0 0 0 0 0 0 0 0 0 0

0 2 81 0 0 0 0 0 0 0 0 0 0 0

0 2 47 0 0 0 0 0 0 0 0 0 0 0

0 2 60 0 0 0 0 0 0 0 0 0 0 0

0 2 65 0 0 0 0 0 0 0 0 0 0 0

0 2 79 0 0 0 0 0 0 0 0 0 0 0

0 2 60 0 0 0 0 0 0 0 0 0 0 0

0 2 72 0 0 0 0 0 0 0 0 0 0 0

0 2 77 0 0 0 0 0 0 0 0 0 0 0

0 2 75 0 0 0 0 0 0 0 0 0 0 0

0 2 76 0 0 0 0 0 0 0 0 0 0 0

0 2 71 0 0 0 0 0 0 0 0 0 0 0

0 2 77 0 0 0 0 0 0 0 0 0 0 0

0 2 57 0 0 0 0 0 0 0 0 0 0 0

0 2 76 0 0 0 0 0 0 0 0 0 0 0

0 2 77 0 0 0 0 0 0 0 0 0 0 0

0 2 63 0 0 0 0 0 0 0 0 0 0 0

0 2 72 0 0 0 0 0 0 0 0 0 0 0

0 2 80 0 0 0 0 0 0 0 0 0 0 0

0 2 51 0 0 0 0 0 0 0 0 0 0 0

0 2 81 0 0 0 0 0 0 0 0 0 0 0

0 2 77 0 0 0 0 0 0 0 0 0 0 0

0 2 72 0 0 0 0 0 0 0 0 0 0 0

0 2 59 0 0 0 0 0 0 0 0 0 0 0

0 2 64 0 0 0 0 0 0 0 0 0 0 0

0 2 64 0 0 0 0 0 0 0 0 0 0 0

0 2 69 0 0 0 0 0 0 0 0 0 0 0

0 2 51 0 0 0 0 0 0 0 0 0 0 0

0 2 76 0 0 0 0 0 0 0 0 0 0 0

0 2 76 0 0 0 0 0 0 0 0 0 0 0

0 2 72 0 0 0 0 0 0 0 0 0 0 0

0 2 71 0 0 0 0 0 0 0 0 0 0 0

0 2 70 0 0 0 0 0 0 0 0 0 0 0

0 2 54 0 0 0 0 0 0 0 0 0 0 0

0 2 74 0 0 0 0 0 0 0 0 0 0 0

0 2 76 0 0 0 0 0 0 0 0 0 0 0

0 2 71 0 0 0 0 0 0 0 0 0 0 0

0 2 76 0 0 0 0 0 0 0 0 0 0 0

0 2 65 0 0 0 0 0 0 0 0 0 0 0

0 2 72 0 0 0 0 0 0 0 0 0 0 0

0 2 76 0 0 0 0 0 0 0 0 0 0 0

0 2 75 0 0 0 0 0 0 0 0 0 0 0

0 2 80 0 0 0 0 0 0 0 0 0 0 0

0 2 54 0 0 0 0 0 0 0 0 0 0 0

0 2 56 0 0 0 0 0 0 0 0 0 0 0

0 2 52 0 0 0 0 0 0 0 0 0 0 0

0 2 62 0 0 0 0 0 0 0 0 0 0 0

0 2 82 0 0 0 0 0 0 0 0 0 0 0

0 2 75 0 0 0 0 0 0 0 0 0 0 0

0 2 84 0 0 0 0 0 0 0 0 0 0 0

0 2 70 0 0 0 0 0 0 0 0 0 0 0

0 2 61 0 0 0 0 0 0 0 0 0 0 0

0 2 59 0 0 0 0 0 0 0 0 0 0 0

0 2 83 0 0 0 0 0 0 0 0 0 0 0

0 2 68 0 0 0 0 0 0 0 0 0 0 0

0 2 74 0 0 0 0 0 0 0 0 0 0 0

0 2 75 0 0 0 0 0 0 0 0 0 0 0

0 2 59 0 0 0 0 0 0 0 0 0 0 0

0 2 73 0 0 0 0 0 0 0 0 0 0 0

0 2 72 0 0 0 0 0 0 0 0 0 0 0

0 2 81 0 0 0 0 0 0 0 0 0 0 0

0 2 42 0 0 0 0 0 0 0 0 0 0 0

0 2 62 0 0 0 0 0 0 0 0 0 0 0

0 2 65 0 0 0 0 0 0 0 0 0 0 0

0 2 72 0 0 0 0 0 0 0 0 0 0 0

0 2 72 0 0 0 0 0 0 0 0 0 0 0

0 2 74 0 0 0 0 0 0 0 0 0 0 0

0 2 78 0 0 0 0 0 0 0 0 0 0 0

0 2 60 0 0 0 0 0 0 0 0 0 0 0

0 2 54 0 0 0 0 0 0 0 0 0 0 0

0 2 60 0 0 0 0 0 0 0 0 0 0 0

0 2 48 0 0 0 0 0 0 0 0 0 0 0

0 2 64 0 0 0 0 0 0 0 0 0 0 0

0 2 81 0 0 0 0 0 0 0 0 0 0 0

0 2 61 0 0 0 0 0 0 0 0 0 0 0

0 2 88 0 0 0 0 0 0 0 0 0 0 0

0 2 75 0 0 0 0 0 0 0 0 0 1 0

0 2 62 0 0 0 0 0 0 0 0 0 4 0

0 2 69 0 0 0 0 0 0 0 0 0 2 0

0 2 86 0 0 0 0 0 0 0 0 0 3 0

0 2 85 0 0 0 0 0 0 0 0 0 0 0

0 2 55 0 0 0 0 0 0 0 0 0 0 0

0 2 60 0 0 0 0 0 0 0 0 0 0 0

0 2 65 0 0 0 0 0 0 0 0 0 0 0

0 2 43 0 0 0 0 0 0 0 0 0 0 0

0 2 80 0 0 0 0 0 0 0 0 0 0 0

0 2 64 0 0 0 0 0 0 0 0 0 0 0

0 2 66 0 0 0 0 0 0 0 0 0 0 0

0 2 80 0 0 0 0 0 0 0 0 0 0 0

0 2 65 0 0 0 0 0 0 0 0 0 0 0

0 2 59 0 0 0 0 0 0 0 0 0 0 0

0 2 74 0 0 0 0 0 0 0 0 0 0 0

0 2 72 0 0 0 0 0 0 0 0 0 3 0

0 2 81 0 0 0 0 0 0 0 0 0 3 0

0 2 56 0 0 0 0 0 0 0 0 0 0 0

0 2 75 0 0 0 0 0 0 0 0 0 0 0

0 2 84 0 0 0 0 0 0 0 0 0 0 0

0 2 87 0 0 0 0 0 0 0 0 0 0 0

0 2 61 0 0 0 0 0 0 0 0 0 0 0

0 2 70 0 0 0 0 0 0 0 0 0 0 0

0 2 62 0 0 0 0 0 0 0 0 0 0 0

0 2 57 0 0 0 0 0 0 0 0 0 0 0

0 2 65 0 0 0 0 0 0 0 0 0 3 0

0 2 76 0 0 0 0 0 0 0 0 0 0 0

0 2 49 0 0 0 0 0 0 0 0 0 0 0

0 2 78 0 0 0 0 0 0 0 0 0 0 0

0 2 68 0 0 0 0 0 0 0 0 0 0 0

0 2 85 0 0 0 0 0 0 0 0 0 1 0

0 2 64 0 0 0 0 0 0 0 0 0 0 0

0 2 74 0 0 0 0 0 0 0 0 0 0 0

0 2 51 0 0 0 0 0 0 0 0 0 2 0

0 2 56 0 0 0 0 0 0 0 0 0 0 0

0 2 82 0 0 0 0 0 0 0 0 0 0 0

0 2 59 0 0 0 0 0 0 0 0 0 0 0

0 2 43 0 0 0 0 0 0 0 0 0 0 0

0 2 62 0 0 0 0 0 0 0 0 0 0 0

0 2 68 0 0 0 0 0 0 0 0 0 0 0

0 2 50 0 0 0 0 0 0 0 0 0 0 0

0 2 71 0 0 0 0 0 0 0 0 0 0 0

0 2 73 0 0 0 0 0 0 0 0 0 0 0

0 2 73 0 0 0 0 0 0 0 0 0 0 0

0 2 75 0 0 0 0 0 0 0 0 0 0 0

0 2 70 0 0 0 0 0 0 0 0 0 0 0

0 2 70 0 0 0 0 0 0 0 0 0 0 0

0 2 78 0 0 0 0 0 0 0 0 0 1 0

0 2 66 0 0 0 0 0 0 0 0 0 0 0

0 2 81 0 0 0 0 0 0 0 0 0 0 0

0 2 67 0 0 0 0 0 0 0 0 0 0 0

0 2 63 0 0 0 0 0 0 0 0 0 0 0

0 2 58 0 0 0 1 0 0 0 0 0 0 0

0 2 72 0 0 0 1 0 0 0 0 0 3 0

0 2 59 0 0 0 1 0 0 0 0 0 0 0

0 2 72 0 0 0 1 0 0 0 0 0 0 0

0 2 52 0 0 0 0 0 0 0 0 0 0 0

0 2 56 0 0 0 0 0 0 0 0 0 0 0

0 2 71 0 0 0 0 0 0 0 0 0 0 0

0 2 74 0 0 0 0 0 0 0 0 0 0 0

0 2 76 0 0 0 0 0 0 0 0 0 0 0

0 2 64 0 0 0 0 0 0 0 0 0 0 0

0 2 54 0 0 0 0 0 0 0 0 0 0 0

0 2 70 0 0 0 0 0 0 0 0 0 0 0

0 2 52 0 0 0 0 0 0 0 0 0 0 0

0 2 63 0 0 0 0 0 0 0 0 0 0 0

0 2 63 0 0 0 0 0 0 0 0 0 0 0

0 2 73 0 0 0 0 0 0 0 0 0 0 0

0 2 72 0 0 0 0 0 0 0 0 0 0 0

0 2 51 0 0 0 0 0 0 0 0 0 0 0

0 2 70 0 0 0 0 0 0 0 0 0 0 0

0 2 63 0 0 0 0 0 0 0 0 0 0 0

0 2 47 0 0 0 0 0 0 0 0 0 0 0

0 2 79 0 0 0 0 0 0 0 0 0 0 0

0 2 67 0 0 0 0 0 0 0 0 0 0 0

0 2 82 0 0 0 0 0 0 0 0 0 0 0

0 2 77 0 0 0 0 0 0 0 0 0 0 0

0 2 72 0 0 0 0 0 0 0 0 0 0 0

0 2 61 0 0 0 0 0 0 0 0 0 0 0

0 2 67 0 0 0 0 0 0 0 0 0 0 0

0 2 33 0 0 0 0 0 0 0 0 0 0 0

0 2 55 0 0 0 0 0 0 0 0 0 0 0

0 2 75 0 0 0 0 0 0 0 0 0 2 0

0 2 87 0 0 0 0 0 0 0 0 0 0 0

0 2 50 0 0 0 0 0 0 0 0 0 0 0

0 2 86 0 0 0 0 0 0 0 0 0 0 0

0 2 74 0 0 0 0 0 0 0 0 0 0 0

0 2 88 0 0 0 0 0 0 0 0 0 0 0

0 2 71 0 0 0 0 0 0 0 0 0 0 0

0 2 86 0 0 0 0 0 0 0 0 0 0 0

0 2 58 0 0 0 0 0 0 0 0 0 0 0

0 2 54 0 0 0 0 0 0 0 0 0 0 0

0 2 77 0 0 0 0 0 0 0 0 0 0 0

0 2 77 0 0 0 0 0 0 0 0 0 0 0

0 2 68 0 0 0 0 0 0 0 0 0 0 0

0 2 75 0 0 0 0 0 0 0 0 0 0 0

0 2 30 0 0 0 0 0 0 0 0 0 0 0

0 2 53 0 0 0 0 0 0 0 0 0 0 0

0 2 71 0 0 0 0 0 0 0 0 0 0 0

0 2 62 0 0 0 0 0 0 0 0 0 0 0

0 2 72 0 0 0 0 0 0 0 0 0 0 0

0 2 60 0 0 0 0 0 0 0 0 0 0 0

0 2 73 0 0 0 0 0 0 0 0 0 0 0

0 2 65 0 0 0 0 0 0 0 0 0 0 0

0 2 53 0 0 0 0 0 0 0 0 0 0 0

0 2 61 0 0 0 0 0 0 0 0 0 0 0

0 2 41 0 0 0 0 0 0 0 0 0 0 0

0 2 38 0 0 0 0 0 0 0 0 0 0 0

0 2 76 0 0 0 0 0 0 0 0 0 0 0

0 2 47 0 0 0 0 0 0 0 0 0 0 0

0 2 74 0 0 0 0 0 0 0 0 0 0 0

0 2 53 0 0 0 0 0 0 0 0 0 0 0

0 2 34 0 0 0 0 0 0 0 0 0 0 0

0 2 46 0 0 0 0 0 0 0 0 0 0 0

0 2 14 0 0 0 0 0 0 0 0 0 0 0

0 2 34 0 0 0 0 0 0 0 0 0 0 0

0 2 37 0 0 0 0 0 0 0 0 0 0 0

0 2 20 0 0 0 0 0 0 0 0 0 0 0

0 2 31 0 0 0 0 0 0 0 0 0 0 0

0 2 45 0 0 0 0 0 0 0 0 0 0 0

0 2 38 0 0 0 0 0 0 0 0 0 0 0

0 2 26 0 0 0 0 0 0 0 0 0 0 0

0 2 18 0 0 0 0 0 0 0 0 0 0 0

0 2 48 0 0 0 0 0 0 0 0 0 0 0

0 2 71 0 0 0 0 0 0 0 0 0 0 0

0 2 60 0 0 0 0 0 0 0 0 0 0 0

0 2 66 0 0 0 0 0 0 0 0 0 0 0

0 2 52 0 0 0 0 0 0 0 0 0 0 0

0 2 85 0 0 0 0 0 0 0 0 0 0 0

0 2 85 0 0 0 0 0 0 0 0 0 0 0

0 2 39 0 0 0 0 0 0 0 0 0 0 0

0 2 54 0 0 0 0 0 0 0 0 0 0 0

0 2 38 0 0 0 0 0 0 0 0 0 0 0

0 2 77 0 0 0 0 0 0 0 0 0 0 0

0 2 78 0 0 0 0 0 0 0 0 0 0 0

0 2 48 0 0 0 1 0 0 0 0 0 0 0

0 2 59 0 0 0 0 0 0 0 0 0 0 0

0 2 25 0 0 0 0 0 0 0 0 0 0 0

0 2 15 0 0 0 0 0 0 0 0 0 0 0

0 2 52 0 0 0 0 0 0 0 0 0 0 0

0 2 18 0 0 0 0 0 0 0 0 0 0 0

0 2 72 0 0 0 0 0 0 0 0 0 0 0

0 2 83 0 0 0 0 0 0 0 0 0 0 0

0 2 72 0 0 0 0 0 0 0 0 0 0 0

0 2 71 0 0 0 0 0 0 0 0 0 0 0

0 2 69 0 0 0 0 0 0 0 0 0 0 0

0 2 78 0 0 0 0 0 0 0 0 0 0 0

0 2 53 0 0 0 0 0 0 0 0 0 0 0

0 2 71 0 0 0 0 0 0 0 0 0 0 0

0 2 59 0 0 0 0 0 0 0 0 0 0 0

0 2 66 0 0 0 0 0 0 0 0 0 0 0

0 2 70 0 0 0 0 0 0 0 0 0 0 0

0 2 78 0 0 0 0 0 0 0 0 0 0 0

0 2 69 0 0 0 0 0 0 0 0 0 0 0

0 2 80 0 0 0 0 0 0 0 0 0 0 0

0 2 51 0 0 0 0 0 0 0 0 0 0 0

0 2 63 0 0 0 0 0 0 0 0 0 0 0

0 2 81 0 0 0 0 0 0 0 0 0 0 0

0 2 74 0 0 0 0 0 0 0 0 0 0 0

0 2 45 0 0 0 0 0 0 0 0 0 0 0

0 2 66 0 0 0 0 0 0 0 0 0 0 0

0 2 58 0 0 0 0 0 0 0 0 0 0 0

0 2 69 0 0 0 0 0 0 0 0 0 0 0

0 2 58 0 0 0 0 0 0 0 0 0 0 0

0 2 63 0 0 0 0 0 0 0 0 0 0 0

0 2 74 0 0 0 0 0 0 0 0 0 0 0

0 2 63 0 0 0 0 0 0 0 0 0 0 0

0 2 83 0 0 0 0 0 0 0 0 0 0 0

0 2 84 0 0 0 0 0 0 0 0 0 0 0

0 2 85 0 0 0 0 0 0 0 0 0 0 0

0 2 69 0 0 0 0 0 0 0 0 0 0 0

0 2 65 0 0 0 0 0 0 0 0 0 0 0

0 2 70 0 0 0 0 0 0 0 0 0 0 0

0 2 79 0 0 0 0 0 0 0 0 0 0 0

0 2 60 0 0 0 1 0 0 0 0 0 0 0

0 2 52 0 0 0 0 0 0 0 0 0 0 0

0 2 57 0 0 0 0 0 0 0 0 0 0 0

0 2 32 0 0 0 0 0 0 0 0 0 0 0

0 2 31 0 0 0 0 0 0 0 0 0 0 0

0 2 51 0 0 0 0 0 0 0 0 0 0 0

0 2 29 0 0 0 0 0 0 0 0 0 0 0

0 2 31 0 0 0 0 0 0 0 0 0 0 0

0 2 62 0 0 0 0 0 0 0 0 0 0 0

0 2 61 0 0 0 1 0 0 0 0 0 0 0

0 2 29 0 0 0 0 0 0 0 0 0 0 0

0 2 44 0 0 0 0 0 0 0 0 0 0 0

0 2 16 0 0 0 0 0 0 0 0 0 0 0

0 2 42 0 0 0 0 0 0 0 0 0 0 0

0 2 66 0 0 0 0 0 0 0 0 0 0 0

0 2 37 0 0 0 0 0 0 0 0 0 0 0

0 2 15 0 0 0 0 0 0 0 0 0 0 0

0 2 18 0 0 0 0 0 0 0 0 0 0 0

0 2 15 0 0 0 0 0 0 0 0 0 0 0

0 2 17 0 0 0 0 0 0 0 0 0 0 0

0 2 18 0 0 0 0 0 0 0 0 0 0 0

0 2 21 0 0 0 0 0 0 0 0 0 3 0

0 2 24 0 0 0 0 0 0 0 0 0 0 0

0 2 57 0 0 0 0 0 0 0 0 0 0 0

0 2 18 0 0 0 0 0 0 0 0 0 0 0

0 2 34 0 0 0 0 0 0 0 0 0 0 0

0 2 43 0 0 0 0 0 0 0 0 0 0 0

0 2 40 0 0 0 0 0 0 0 0 0 0 0

0 2 66 0 0 0 0 0 0 0 0 0 0 0

0 2 73 0 0 0 0 0 0 0 0 0 0 0

0 2 86 0 0 0 0 0 0 0 0 0 0 0

0 2 62 0 0 0 0 0 0 0 0 0 0 0

0 2 71 0 0 0 0 0 0 0 0 0 0 0

0 2 46 0 0 0 0 0 0 0 0 0 0 0

0 2 15 0 0 0 0 0 0 0 0 0 0 0

0 2 54 0 0 0 0 0 0 0 0 0 0 0

0 2 49 0 0 0 0 0 0 0 0 0 0 0

0 2 62 0 0 0 0 0 0 0 0 0 0 0

0 2 41 0 0 0 0 0 0 0 0 0 3 0

0 2 69 0 0 0 0 0 0 0 0 0 0 0

0 2 39 0 0 0 0 0 0 0 0 0 0 0

0 2 39 0 0 0 0 0 0 0 0 0 0 0

0 2 59 0 0 0 0 0 0 0 0 0 0 0

0 2 65 0 0 0 0 0 0 0 0 0 0 0

0 2 47 0 0 0 0 0 0 0 0 0 0 0

0 2 82 0 0 0 0 0 0 0 0 0 0 0

0 2 71 0 0 0 0 0 0 0 0 0 0 0

0 2 42 0 0 0 0 0 0 0 0 0 0 0

0 2 57 0 0 0 0 0 0 0 0 0 0 0

0 2 58 0 0 0 0 0 0 0 0 0 0 0

0 2 29 0 0 0 0 0 0 0 0 0 0 0

0 2 39 0 0 0 0 0 0 0 0 0 0 0

0 2 59 0 0 0 0 0 0 0 0 0 0 0

0 2 77 0 0 0 0 0 0 0 0 0 0 0

0 2 76 0 0 0 0 0 0 0 0 0 0 0

0 2 17 0 0 0 0 0 0 0 0 0 0 0

0 2 70 0 0 0 0 0 0 0 0 0 0 0

0 2 27 0 0 0 0 0 0 0 0 0 4 0

0 2 36 0 0 0 0 0 0 0 0 0 0 0

0 2 35 0 0 0 0 0 0 0 0 0 0 0

0 2 37 0 0 0 0 0 0 0 0 0 0 0

0 2 54 0 0 0 0 0 0 0 0 0 0 0

0 2 51 0 0 0 0 0 0 0 0 0 0 0

0 2 63 0 0 0 0 0 0 0 0 0 0 0

0 2 50 0 0 0 0 0 0 0 0 0 0 0

0 2 56 0 0 0 0 0 0 0 0 0 0 0

0 2 57 0 0 0 0 0 0 0 0 0 0 0

0 2 62 0 0 0 0 0 0 0 0 0 0 0

0 2 80 0 0 0 0 0 0 0 0 0 0 0

0 2 43 0 0 0 0 0 0 0 0 0 0 0

0 2 46 0 0 0 0 0 0 0 0 0 0 0

0 2 69 0 0 0 0 0 0 0 0 0 0 0

0 2 51 0 0 0 0 0 0 0 0 0 3 0

0 2 70 0 0 0 0 0 0 0 0 0 0 0

0 2 52 0 0 0 0 0 0 0 0 0 0 0

0 2 71 0 0 0 0 0 0 0 0 0 0 0

0 2 48 0 0 0 0 0 0 0 0 0 0 0

0 2 52 0 0 0 0 0 0 0 0 0 0 0

0 2 51 0 0 0 0 0 0 0 0 0 0 0

0 2 73 0 0 0 0 0 0 0 0 0 0 0

0 2 44 0 0 0 0 0 0 0 0 0 0 0

0 2 43 0 0 0 0 0 0 0 0 0 0 0

0 2 59 0 0 0 0 0 0 0 0 0 0 0

0 2 82 0 0 0 0 0 0 0 0 0 0 0

0 2 51 0 0 0 0 0 0 0 0 0 0 0

0 2 64 0 0 0 0 0 0 0 0 0 0 0

0 2 65 0 0 0 0 0 0 0 0 0 0 0

0 2 47 0 0 0 0 0 0 0 0 0 0 0

0 2 67 0 0 0 0 0 0 0 0 0 0 0

0 2 59 0 0 0 0 0 0 0 0 0 0 0

0 2 56 0 0 0 0 0 0 0 0 0 0 0

0 2 59 0 0 0 1 0 0 0 0 0 0 0

0 2 29 0 0 0 1 0 0 0 0 0 3 0

0 2 65 0 0 0 1 0 0 0 0 0 0 0

0 2 36 0 0 0 1 0 0 0 0 0 0 0

0 2 59 0 0 0 1 0 0 0 0 0 0 0

0 2 51 0 0 0 1 0 0 0 0 0 0 0

0 2 54 0 0 0 1 0 0 0 0 0 0 0

0 2 52 0 0 0 1 0 0 0 0 0 0 0

0 2 57 0 0 0 1 0 0 0 0 0 0 0

0 2 65 0 0 0 1 0 0 0 0 0 0 0

0 2 56 0 0 0 1 0 0 0 0 0 1 0

0 2 54 0 0 0 1 0 0 0 0 0 0 0

0 2 57 0 0 0 1 0 0 0 0 0 0 0

0 2 50 0 0 0 1 0 0 0 0 0 0 0

0 2 70 0 0 0 1 0 0 0 0 0 0 0

0 2 54 0 0 0 1 0 0 0 0 0 0 0

0 2 50 0 0 0 1 0 0 0 0 0 0 0

0 2 53 0 0 0 1 0 0 0 0 0 0 0

0 2 48 0 0 0 0 0 0 0 0 0 0 0

0 2 56 0 0 0 0 0 0 0 0 0 0 0

0 2 71 0 0 0 0 0 0 0 0 0 0 0

0 2 81 0 0 0 0 0 0 0 0 0 0 0

0 2 84 0 0 0 0 0 0 0 0 0 0 0

0 2 86 0 0 0 0 0 0 0 0 0 0 0

0 2 35 0 0 0 0 0 0 0 0 0 0 0

0 2 72 0 0 0 0 0 0 0 0 0 0 0

0 2 22 0 0 0 0 0 0 0 0 0 2 0

0 2 70 0 0 0 0 0 0 0 0 0 0 0

0 2 27 0 0 0 0 0 0 0 0 0 0 0

0 2 53 0 0 0 0 0 0 0 0 0 0 0

0 2 49 0 0 0 0 0 0 0 0 0 0 0

0 2 37 0 0 0 0 0 0 0 0 0 0 0

0 2 47 0 0 0 0 0 0 0 0 0 0 0

0 2 16 0 0 0 0 0 0 0 0 0 0 0

0 2 36 0 0 0 0 0 0 0 0 0 0 0

0 2 70 0 0 0 0 0 0 0 0 0 0 0

0 2 80 0 0 0 0 0 0 0 0 0 0 0

0 2 20 0 0 0 0 0 0 0 0 0 0 0

0 2 48 0 0 0 0 0 0 0 0 0 0 0

0 2 59 0 0 0 0 0 0 0 0 0 0 0

0 2 39 0 0 0 0 0 0 0 0 0 0 0

0 2 50 0 0 0 0 0 0 0 0 0 0 0

0 2 21 0 0 0 0 0 0 0 0 0 0 0

0 2 38 0 0 0 0 0 0 0 0 0 0 0

0 2 27 0 0 0 0 0 0 0 0 0 0 0

0 2 34 0 0 0 0 0 0 0 0 0 0 0

0 2 28 0 0 0 0 0 0 0 0 0 0 0

0 2 47 0 0 0 0 0 0 0 0 0 0 0

0 2 75 0 0 0 0 0 0 0 0 0 0 0

0 2 68 0 0 0 0 0 0 0 0 0 0 0

0 2 71 0 0 0 0 0 0 0 0 0 0 0

0 2 76 0 0 0 0 0 0 0 0 0 0 0

0 2 85 0 0 0 0 0 0 0 0 0 0 0

0 2 70 0 0 0 0 0 0 0 0 0 0 0

0 2 75 0 0 0 0 0 0 0 0 0 0 0

0 2 72 0 0 0 0 0 0 0 0 0 0 0

0 2 72 0 0 0 0 0 0 0 0 0 0 0

0 2 92 0 0 0 0 0 0 0 0 0 0 0

0 2 94 0 0 0 0 0 0 0 0 0 0 0

0 2 90 0 0 0 0 0 0 0 0 0 0 0

0 2 72 0 0 0 0 0 0 0 0 0 0 0

0 2 90 0 0 0 0 0 0 0 0 0 0 0

0 2 70 0 0 0 0 0 0 0 0 0 0 0

0 2 68 0 0 0 0 0 0 0 0 0 0 0

0 2 78 0 0 0 0 0 0 0 0 0 0 0

0 2 57 0 0 0 0 0 0 0 0 0 0 0

0 2 59 0 0 0 0 0 0 0 0 0 0 0

0 2 48 0 0 0 0 0 0 0 0 0 0 0

0 2 83 0 0 0 0 0 0 0 0 0 0 0

0 2 63 0 0 0 0 0 0 0 0 0 0 0

0 2 80 0 0 0 0 0 0 0 0 0 0 0

0 2 81 0 0 0 0 0 0 0 0 0 0 0

0 2 85 0 0 0 0 0 0 0 0 0 0 0

0 2 80 0 0 0 0 0 0 0 0 0 0 0

0 2 90 0 0 0 0 0 0 0 0 0 0 0

0 2 78 0 0 0 0 0 0 0 0 0 0 0

0 2 61 0 0 0 0 0 0 0 0 0 0 0

0 2 15 0 0 0 0 0 0 0 0 0 0 0

0 2 57 0 0 0 0 0 0 0 0 0 0 0

0 2 52 0 0 0 0 0 0 0 0 0 0 0

0 2 31 0 0 0 0 0 0 0 0 0 0 0

0 2 53 0 0 0 1 0 0 0 0 0 0 0

0 2 74 0 0 0 0 0 0 0 0 0 0 0

0 2 50 0 0 0 0 0 0 0 0 0 0 0

0 2 57 0 0 0 0 0 0 0 0 0 0 0

0 2 49 0 0 0 0 0 0 0 0 0 0 0

0 2 50 0 0 0 0 0 0 0 0 0 0 0

0 2 51 0 0 0 0 0 0 0 0 0 0 0

0 2 60 0 0 0 0 0 0 0 0 0 0 0

0 2 28 0 0 0 0 0 0 0 0 0 0 0

0 2 78 0 0 0 0 0 0 0 0 0 0 0

0 2 20 0 0 0 0 0 0 0 0 0 0 0

0 2 39 0 0 0 0 0 0 0 0 0 0 0

0 2 43 0 0 0 0 0 0 0 0 0 0 0

0 2 92 0 0 0 0 0 0 0 0 0 0 0

0 2 59 0 0 0 0 0 0 0 0 0 0 0

0 2 71 0 0 0 0 0 0 0 0 0 0 0

0 2 47 0 0 0 0 0 0 0 0 0 0 0

0 2 58 0 0 0 0 0 0 0 0 0 0 0

0 2 74 0 0 0 0 0 0 0 0 0 0 0

0 2 62 0 0 0 0 0 0 0 0 0 0 0

0 2 74 0 0 0 0 0 0 0 0 0 0 0

0 2 58 0 0 0 0 0 0 0 0 0 0 0

0 2 54 0 0 0 0 0 0 0 0 0 3 0

0 2 58 0 0 0 1 0 0 0 0 0 4 0

0 2 53 0 0 0 1 0 0 0 0 0 0 0

0 2 55 0 0 0 0 0 0 0 0 0 0 0

0 2 73 0 0 0 0 0 0 0 0 0 0 0

0 2 73 0 0 0 0 0 0 0 0 0 0 0

0 2 74 0 0 0 0 0 0 0 0 0 0 0

0 2 62 0 0 0 0 0 0 0 0 0 0 0

0 2 46 0 0 0 0 0 0 0 0 0 3 0

0 2 60 0 0 0 0 0 0 0 0 0 0 0

0 2 56 0 0 0 0 0 0 0 0 0 0 0

0 2 52 0 0 0 0 0 0 0 0 0 2 0

0 2 49 0 0 0 0 0 0 0 0 0 2 0

0 2 54 0 0 0 0 0 0 0 0 0 2 0

0 2 64 0 0 0 0 0 0 0 0 0 2 0

0 2 63 0 0 0 0 0 0 0 0 0 2 0

0 2 66 0 0 0 0 0 0 0 0 0 2 0

0 2 43 0 0 0 0 0 0 0 0 0 2 0

0 2 52 0 0 0 1 0 0 0 0 0 3 0

0 2 62 0 0 0 1 0 0 0 0 0 3 0

0 2 50 0 0 0 1 0 0 0 0 0 3 0

0 2 48 0 0 0 0 0 0 0 0 0 3 0

0 2 64 0 0 0 0 0 0 0 0 0 4 0

0 2 67 0 0 0 0 0 0 0 0 0 4 0

0 2 57 0 0 0 0 0 0 0 0 0 4 0

0 2 60 0 0 0 0 0 0 0 0 0 0 0

0 2 41 0 0 0 0 0 0 0 0 0 0 0

0 2 36 0 0 0 0 0 0 0 0 0 0 0

0 2 56 0 0 0 0 0 0 0 0 0 0 0

0 2 58 0 0 0 0 0 0 0 0 0 0 0

0 2 56 0 0 0 0 0 0 0 0 0 0 0

0 2 22 0 0 0 0 0 0 0 0 0 0 0

0 2 38 0 0 0 0 0 0 0 0 0 0 0

0 2 20 0 0 0 0 0 0 0 0 0 0 0

0 2 48 0 0 0 0 0 0 0 0 0 0 0

0 2 48 0 0 0 0 0 0 0 0 0 0 0

0 2 58 0 0 0 0 0 0 0 0 0 0 0

0 2 62 0 0 0 0 0 0 0 0 0 0 0

0 2 78 0 0 0 0 0 0 0 0 0 0 0

0 2 49 0 0 0 0 0 0 0 0 0 0 0

0 2 77 0 0 0 0 0 0 0 0 0 0 0

0 2 62 0 0 0 0 0 0 0 0 0 0 0

0 2 58 0 0 0 0 0 0 0 0 0 0 0

0 2 48 0 0 0 0 0 0 0 0 0 0 0

0 2 61 0 0 0 0 0 0 0 0 0 0 0

0 2 41 0 0 0 0 0 0 0 0 0 0 0

0 2 61 0 0 0 0 0 0 0 0 0 0 0

0 2 40 0 0 0 0 0 0 0 0 0 0 0

0 2 43 0 0 0 0 0 0 0 0 0 0 0

0 2 53 0 0 0 0 0 0 0 0 0 0 0

0 2 51 0 0 0 0 0 0 0 0 0 0 0

0 2 41 0 0 0 0 0 0 0 0 0 0 0

0 2 62 0 0 0 0 0 0 0 0 0 0 0

0 2 53 0 0 0 0 0 0 0 0 0 0 0

0 2 56 0 0 0 0 0 0 0 0 0 0 0

0 2 16 0 0 0 0 0 0 0 0 0 0 0

0 2 28 0 0 0 0 0 0 0 0 0 0 0

0 2 38 0 0 0 0 0 0 0 0 0 0 0

0 2 89 0 0 0 0 0 0 0 0 0 0 0

0 2 85 0 0 0 0 0 0 0 0 0 0 0

0 2 61 0 0 0 0 0 0 0 0 0 0 0

0 2 73 0 0 0 0 0 0 0 0 0 0 0

0 2 24 0 0 0 0 0 0 0 0 0 0 0

0 2 60 0 0 0 0 0 0 0 0 0 0 0

0 2 59 0 0 0 0 0 0 0 0 0 0 0

0 2 81 0 0 0 0 0 0 0 0 0 0 0

0 2 54 0 0 0 0 0 0 0 0 0 0 0

0 2 78 0 0 0 0 0 0 0 0 0 0 0

0 2 69 0 0 0 0 0 0 0 0 0 0 0

0 2 49 0 0 0 0 0 0 0 0 0 0 0

0 2 64 0 0 0 0 0 0 0 0 0 0 0

0 2 55 0 0 0 0 0 0 0 0 0 0 0

0 2 62 0 0 0 0 0 0 0 0 0 0 0

0 2 61 0 0 0 0 0 0 0 0 0 0 0

0 2 49 0 0 0 0 0 0 0 0 0 0 0

0 2 53 0 0 0 0 0 0 0 0 0 0 0

0 2 80 0 0 0 0 0 0 0 0 0 0 0

0 2 78 0 0 0 0 0 0 0 0 0 0 0

0 2 44 0 0 0 0 0 0 0 0 0 0 0

0 2 44 0 0 0 0 0 0 0 0 0 0 0

0 2 54 0 0 0 0 0 0 0 0 0 0 0

0 2 49 0 0 0 0 0 0 0 0 0 0 0

0 2 50 0 0 0 0 0 0 0 0 0 0 0

0 2 56 0 0 0 0 0 0 0 0 0 0 0

0 2 55 0 0 0 0 0 0 0 0 0 0 0

0 2 48 0 0 0 0 0 0 0 0 0 0 0

0 2 45 0 0 0 0 0 0 0 0 0 0 0

0 2 67 0 0 0 0 0 0 0 0 0 0 0

0 2 72 0 0 0 0 0 0 0 0 0 0 0

0 2 48 0 0 0 0 0 0 0 0 0 0 0

0 2 43 0 0 0 0 0 0 0 0 0 0 0

0 2 47 0 0 0 0 0 0 0 0 0 0 0

0 2 29 0 0 0 1 0 0 0 0 0 0 0

0 2 86 0 0 0 0 0 0 0 0 0 0 0

0 2 58 0 0 0 0 0 0 0 0 0 0 0

0 2 30 0 0 0 0 0 0 0 0 0 0 0

0 2 22 0 0 0 0 0 0 0 0 0 0 0

0 2 29 0 0 0 0 0 0 0 0 0 0 0

0 2 22 0 0 0 0 0 0 0 0 0 0 0

0 2 35 0 0 0 0 0 0 0 0 0 0 0

0 2 23 0 0 0 0 0 0 0 0 0 0 0

0 2 57 0 0 0 0 0 0 0 0 0 0 0

0 2 72 0 0 0 0 0 0 0 0 0 0 0

0 2 56 0 0 0 0 0 0 0 0 0 0 0

0 2 61 0 0 0 0 0 0 0 0 0 0 0

0 2 75 0 0 0 0 0 0 0 0 0 0 0

0 2 60 0 0 0 0 0 0 0 0 0 0 0

0 2 37 0 0 0 0 0 0 0 0 0 0 0

0 2 84 0 0 0 0 0 0 0 0 0 0 0

0 2 24 0 0 0 0 0 0 0 0 0 0 0

0 2 55 0 0 0 0 0 0 0 0 0 0 0

0 2 48 0 0 0 0 0 0 0 0 0 0 0

0 2 50 0 0 0 0 0 0 0 0 0 0 0

0 2 27 0 0 0 0 0 0 0 0 0 0 0

0 2 31 0 0 0 0 0 0 0 0 0 0 0

0 2 29 0 0 0 0 0 0 0 0 0 0 0

0 2 18 0 0 0 0 0 0 0 0 0 0 0

0 2 15 0 0 0 0 0 0 0 0 0 0 0

0 2 35 0 0 0 0 0 0 0 0 0 0 0

0 2 21 0 0 0 0 0 0 0 0 0 0 0

0 2 24 0 0 0 0 0 0 0 0 0 0 0

0 2 25 0 0 0 0 0 0 0 0 0 0 0

0 2 23 0 0 0 0 0 0 0 0 0 0 0

0 2 29 0 0 0 0 0 0 0 0 0 0 0

0 2 71 0 0 0 0 0 0 0 0 0 0 0

0 2 76 0 0 0 0 0 0 0 0 0 0 0

0 2 25 0 0 0 0 0 0 0 0 0 0 0

0 2 24 0 0 0 0 0 0 0 0 0 0 0

0 2 43 0 0 0 0 0 0 0 0 0 0 0

0 2 41 0 0 0 0 0 0 0 0 0 0 0

0 2 37 0 0 0 0 0 0 0 0 0 0 0

0 2 65 0 0 0 0 0 0 0 0 0 0 0

0 2 53 0 0 0 0 0 0 0 0 0 0 0

0 2 34 0 0 0 0 0 0 0 0 0 0 0

0 2 31 0 0 0 0 0 0 0 0 0 0 0

0 2 15 0 0 0 0 0 0 0 0 0 0 0

0 2 54 0 0 0 0 0 0 0 0 0 0 0

0 2 50 0 0 0 0 0 0 0 0 0 0 0

0 2 55 0 0 0 0 0 0 0 0 0 0 0

0 2 34 0 0 0 0 0 0 0 0 0 0 0

0 2 77 0 0 0 0 0 0 0 0 0 0 0

0 2 72 0 0 0 0 0 0 0 0 0 0 0

0 2 24 0 0 0 0 0 0 0 0 0 0 0

0 2 62 0 0 0 0 0 0 0 0 0 0 0

0 2 56 0 0 0 0 0 0 0 0 0 0 0

0 2 60 0 0 0 0 0 0 0 0 0 0 0

0 2 66 0 0 0 0 0 0 0 0 0 0 0

0 2 14 0 0 0 0 0 0 0 0 0 0 0

0 2 48 0 0 0 0 0 0 0 0 0 0 0

0 2 24 0 0 0 0 0 0 0 0 0 0 0

0 2 83 0 0 0 0 0 0 0 0 0 0 0

0 2 58 0 0 0 0 0 0 0 0 0 0 0

0 2 38 0 0 0 0 0 0 0 0 0 0 0

0 2 52 0 0 0 0 0 0 0 0 0 0 0

0 2 38 0 0 0 0 0 0 0 0 0 0 0

0 2 76 0 0 0 0 0 0 0 0 0 0 0

0 2 79 0 0 0 0 0 0 0 0 0 0 0

0 2 55 0 0 0 0 0 0 0 0 0 0 0

0 2 27 0 0 0 0 0 0 0 0 0 0 0

0 2 75 0 0 0 0 0 0 0 0 0 0 0

0 2 48 0 0 0 0 0 0 0 0 0 0 0

0 2 47 0 0 0 0 0 0 0 0 0 0 0

0 2 56 0 0 0 0 0 0 0 0 0 0 0

0 2 72 0 0 0 0 0 0 0 0 0 0 0

0 2 77 0 0 0 0 0 0 0 0 0 0 0

0 2 59 0 0 0 0 0 0 0 0 0 0 0

0 2 89 0 0 0 0 0 0 0 0 0 0 0

0 2 73 0 0 0 0 0 0 0 0 0 3 0

0 2 63 0 0 0 0 0 0 0 0 0 0 0

0 2 85 0 0 0 0 0 0 0 0 0 0 0

0 2 75 0 0 0 0 0 0 0 0 0 0 0

0 2 79 0 0 0 0 0 0 0 0 0 0 0

0 2 42 0 0 0 0 0 0 0 0 0 0 0

0 2 79 0 0 0 0 0 0 0 0 0 0 0

0 2 76 0 0 0 0 0 0 0 0 0 0 0

0 2 77 0 0 0 0 0 0 0 0 0 3 0

0 2 80 0 0 0 0 0 0 0 0 0 0 0

0 2 61 0 0 0 0 0 0 0 0 0 0 0

0 2 73 0 0 0 0 0 0 0 0 0 0 0

0 2 68 0 0 0 0 0 0 0 0 0 0 0

0 2 75 0 0 0 0 0 0 0 0 0 0 0

0 2 72 0 0 0 0 0 0 0 0 0 0 0

0 2 77 0 0 0 0 0 0 0 0 0 0 0

0 2 77 0 0 0 0 0 0 0 0 0 0 0

0 2 48 0 0 0 0 0 0 0 0 0 0 0

0 2 84 0 0 0 0 0 0 0 0 0 0 0

0 2 47 0 0 0 0 0 0 0 0 0 0 0

0 2 70 0 0 0 0 0 0 0 0 0 0 0

0 2 73 0 0 0 0 0 0 0 0 0 0 0

0 2 86 0 0 0 0 0 0 0 0 0 0 0

0 2 62 0 0 0 0 0 0 0 0 0 0 0

0 2 73 0 0 0 0 0 0 0 0 0 0 0

0 2 68 0 0 0 0 0 0 0 0 0 0 0

0 2 65 0 0 0 0 0 0 0 0 0 0 0

0 2 65 0 0 0 0 0 0 0 0 0 0 0

0 2 52 0 0 0 0 0 0 0 0 0 0 0

0 2 44 0 0 0 0 0 0 0 0 0 0 0

0 2 75 0 0 0 0 0 0 0 0 0 0 0

0 2 87 0 0 0 0 0 0 0 0 0 0 0

0 2 24 0 0 0 0 0 0 0 0 0 0 0

0 2 60 0 0 0 0 0 0 0 0 0 0 0

0 2 46 0 0 0 0 0 0 0 0 0 0 0

0 2 45 0 0 0 0 0 0 0 0 0 0 0

0 2 63 0 0 0 0 0 0 0 0 0 0 0

0 2 75 0 0 0 0 0 0 0 0 0 0 0

0 2 66 0 0 0 0 0 0 0 0 0 0 0

0 2 52 0 0 0 0 0 0 0 0 0 0 0

0 2 49 0 0 0 0 0 0 0 0 0 0 0

0 2 41 0 0 0 0 0 0 0 0 0 0 0

0 2 31 0 0 0 0 0 0 0 0 0 0 0

0 2 42 0 0 0 0 0 0 0 0 0 0 0

0 2 52 0 0 0 0 0 0 0 0 0 0 0

0 2 74 0 0 0 0 0 0 0 0 0 0 0

0 2 59 0 0 0 0 0 0 0 0 0 0 0

0 2 44 0 0 0 0 0 0 0 0 0 0 0

0 2 30 0 0 0 0 0 0 0 0 0 0 0

0 2 75 0 0 0 0 0 0 0 0 0 0 0

0 2 63 0 0 0 0 0 0 0 0 0 0 0

0 2 50 0 0 0 0 0 0 0 0 0 0 0

0 2 44 0 0 0 0 0 0 0 0 0 0 0

0 2 77 0 0 0 0 0 0 0 0 0 0 0

0 2 40 0 0 0 0 0 0 0 0 0 0 0

0 2 53 0 0 0 0 0 0 0 0 0 0 0

0 2 75 0 0 0 0 0 0 0 0 0 0 0

0 2 62 0 0 0 0 0 0 0 0 0 0 0

0 2 53 0 0 0 0 0 0 0 0 0 0 0

0 2 69 0 0 0 0 0 0 0 0 0 0 0

0 2 60 0 0 0 0 0 0 0 0 0 0 0

0 2 50 0 0 0 0 0 0 0 0 0 0 0

0 2 51 0 0 0 0 0 0 0 0 0 0 0

0 2 54 0 0 0 0 0 0 0 0 0 0 0

0 2 55 0 0 0 0 0 0 0 0 0 0 0

0 2 71 0 0 0 0 0 0 0 0 0 0 0

0 2 61 0 0 0 0 0 0 0 0 0 0 0

0 2 37 0 0 0 0 0 0 0 0 0 0 0

0 2 76 0 0 0 0 0 0 0 0 0 0 0

0 2 52 0 0 0 0 0 0 0 0 0 0 0

0 2 24 0 0 0 0 0 0 0 0 0 0 0

0 2 50 0 0 0 0 0 0 0 0 0 0 0

0 2 49 0 0 0 0 0 0 0 0 0 0 0

0 2 38 0 0 0 0 0 0 0 0 0 0 0

0 2 48 0 0 0 0 0 0 0 0 0 0 0

0 2 38 0 0 0 0 0 0 0 0 0 0 0

0 2 63 0 0 0 0 0 0 0 0 0 0 0

0 2 64 0 0 0 0 0 0 0 0 0 0 0

0 2 61 0 0 0 0 0 0 0 0 0 0 0

0 2 82 0 0 0 0 0 0 0 0 0 0 0

0 2 79 0 0 0 0 0 0 0 0 0 0 0

0 2 89 0 0 0 0 0 0 0 0 0 0 0

0 2 75 0 0 0 0 0 0 0 0 0 0 0

0 2 73 0 0 0 0 0 0 0 0 0 0 0

0 2 43 0 0 0 0 0 0 0 0 0 0 0

0 2 59 0 0 0 0 0 0 0 0 0 0 0

0 2 66 0 0 0 0 0 0 0 0 0 0 0

0 2 77 0 0 0 0 0 0 0 0 0 0 0

0 2 69 0 0 0 0 0 0 0 0 0 0 0

0 2 61 0 0 0 0 0 0 0 0 0 0 0

0 2 53 0 0 0 0 0 0 0 0 0 0 0

0 2 59 0 0 0 0 0 0 0 0 0 0 0

0 2 59 0 0 0 0 0 0 0 0 0 0 0

0 2 32 0 0 0 0 0 0 0 0 0 0 0

0 2 49 0 0 0 0 0 0 0 0 0 0 0

0 2 63 0 0 0 0 0 0 0 0 0 0 0

0 2 49 0 0 0 0 0 0 0 0 0 0 0

0 2 82 0 0 0 0 0 0 0 0 0 0 0

0 2 64 0 0 0 0 0 0 0 0 0 0 0

0 2 73 0 0 0 0 0 0 0 0 0 0 0

0 2 24 0 0 0 0 0 0 0 0 0 0 0

0 2 70 0 0 0 0 0 0 0 0 0 0 0

0 2 78 0 0 0 0 0 0 0 0 0 0 0

0 2 50 0 0 0 0 0 0 0 0 0 0 0

0 2 38 0 0 0 0 0 0 0 0 0 0 0

0 2 35 0 0 0 0 0 0 0 0 0 0 0

0 2 40 0 0 0 0 0 0 0 0 0 0 0

0 2 25 0 0 0 0 0 0 0 0 0 0 0

0 2 25 0 0 0 0 0 0 0 0 0 0 0

0 2 15 0 0 0 0 0 0 0 0 0 0 0

0 2 48 0 0 0 0 0 0 0 0 0 0 0

0 2 55 0 0 0 0 0 0 0 0 0 0 0

0 2 28 0 0 0 0 0 0 0 0 0 0 0

0 2 46 0 0 0 0 0 0 0 0 0 0 0

0 2 23 0 0 0 0 0 0 0 0 0 0 0

0 2 51 0 0 0 0 0 0 0 0 0 0 0

0 2 52 0 0 0 0 0 0 0 0 0 0 0

0 2 32 0 0 0 0 0 0 0 0 0 0 0

0 2 56 0 0 0 0 0 0 0 0 0 0 0

0 2 52 0 0 0 0 0 0 0 0 0 0 0

0 2 63 0 0 0 0 0 0 0 0 0 0 0

0 2 68 0 0 0 0 0 0 0 0 0 0 0

0 2 44 0 0 0 0 0 0 0 0 0 0 0

0 2 52 0 0 0 0 0 0 0 0 0 0 0

0 2 55 0 0 0 1 0 0 0 0 0 0 0

0 2 40 0 0 0 0 0 0 0 0 0 0 0

0 2 37 0 0 0 0 0 0 0 0 0 0 0

0 2 56 0 0 0 0 0 0 0 0 0 0 0

0 2 70 0 0 0 0 0 0 0 0 0 0 0

0 2 28 0 0 0 0 0 0 0 0 0 0 0

0 2 42 0 0 0 0 0 0 0 0 0 0 0

0 2 31 0 0 0 0 0 0 0 0 0 0 0

0 2 60 0 0 0 0 0 0 0 0 0 0 0

0 2 54 0 0 0 0 0 0 0 0 0 0 0

0 2 51 0 0 0 0 0 0 0 0 0 0 0

0 2 47 0 0 0 0 0 0 0 0 0 0 0

0 2 41 0 0 0 0 0 0 0 0 0 0 0

0 2 53 0 0 0 0 0 0 0 0 0 0 0

0 2 42 0 0 0 0 0 0 0 0 0 0 0

0 2 57 0 0 0 0 0 0 0 0 0 0 0

0 2 61 0 0 0 0 0 0 0 0 0 0 0

0 2 55 0 0 0 0 0 0 0 0 0 0 0

0 2 48 0 0 0 0 0 0 0 0 0 0 0

0 2 55 0 0 0 0 0 0 0 0 0 0 0

0 2 42 0 0 0 0 0 0 0 0 0 0 0

0 2 69 0 0 0 0 0 0 0 0 0 0 0

0 2 17 0 0 0 0 0 0 0 0 0 0 0

0 2 46 0 0 0 0 0 0 0 0 0 0 0

0 2 65 0 0 0 0 0 0 0 0 0 0 0

0 2 53 0 0 0 0 0 0 0 0 0 0 0

0 2 62 0 0 0 0 0 0 0 0 0 0 0

0 2 67 0 0 0 0 0 0 0 0 0 0 0

0 2 73 0 0 0 0 0 0 0 0 0 0 0

0 2 47 0 0 0 0 0 0 0 0 0 0 0

0 2 75 0 0 0 0 0 0 0 0 0 0 0

0 2 62 0 0 0 0 0 0 0 0 0 0 0

0 2 68 0 0 0 0 0 0 0 0 0 0 0

0 2 43 0 0 0 0 0 0 0 0 0 0 0

0 2 51 0 0 0 0 0 0 0 0 0 0 0

0 2 62 0 0 0 0 0 0 0 0 0 0 0

0 2 32 0 0 0 0 0 0 0 0 0 0 0

0 2 77 0 0 0 0 0 0 0 0 0 0 0

0 2 60 0 0 0 0 0 0 0 0 0 0 0

0 2 28 0 0 0 0 0 0 0 0 0 0 0

0 2 62 0 0 0 0 0 0 0 0 0 0 0

0 2 32 0 0 0 0 0 0 0 0 0 0 0

0 2 58 0 0 0 0 0 0 0 0 0 0 0

0 2 15 0 0 0 0 0 0 0 0 0 0 0

0 2 20 0 0 0 0 0 0 0 0 0 0 0

0 2 51 0 0 0 0 0 0 0 0 0 0 0

0 2 66 0 0 0 0 0 0 0 0 0 0 0

0 2 55 0 0 0 0 0 0 0 0 0 0 0

0 2 52 0 0 0 0 0 0 0 0 0 0 0

0 2 22 0 0 0 0 0 0 0 0 0 0 0

0 2 37 0 0 0 0 0 0 0 0 0 0 0

0 2 41 0 0 0 0 0 0 0 0 0 0 0

0 2 77 0 0 0 0 0 0 0 0 0 0 0

0 2 55 0 0 0 0 0 0 0 0 0 0 0

0 2 53 0 0 0 0 0 0 0 0 0 0 0

0 2 52 0 0 0 0 0 0 0 0 0 0 0

0 2 48 0 0 0 0 0 0 0 0 0 0 0

0 2 63 0 0 0 0 0 0 0 0 0 0 0

0 2 64 0 0 0 0 0 0 0 0 0 0 0

0 2 49 0 0 0 0 0 0 0 0 0 0 0

0 2 32 0 0 0 0 0 0 0 0 0 0 0

0 2 71 0 0 0 0 0 0 0 0 0 0 0

0 2 56 0 0 0 0 0 0 0 0 0 0 0

0 2 41 0 0 0 0 0 0 0 0 0 0 0

0 2 58 0 0 0 0 0 0 0 0 0 0 0

0 2 58 0 0 0 0 0 0 0 0 0 0 0

0 2 59 0 0 0 0 0 0 0 0 0 0 0

0 2 64 0 0 0 0 0 0 0 0 0 0 0

0 2 37 0 0 0 0 0 0 0 0 0 0 0

0 2 46 0 0 0 0 0 0 0 0 0 0 0

0 2 55 0 0 0 0 0 0 0 0 0 0 0

0 2 77 0 0 0 0 0 0 0 0 0 0 0

0 2 70 0 0 0 0 0 0 0 0 0 0 0

0 2 55 0 0 0 0 0 0 0 0 0 0 0

0 2 63 0 0 0 0 0 0 0 0 0 0 0

0 2 74 0 0 0 0 0 0 0 0 0 0 0

0 2 39 0 0 0 0 0 0 0 0 0 0 0

0 2 75 0 0 0 0 0 0 0 0 0 0 0

0 2 40 0 0 0 0 0 0 0 0 0 0 0

0 2 32 0 0 0 0 0 0 0 0 0 0 0

0 2 19 0 0 0 0 0 0 0 0 0 0 0

0 2 66 0 0 0 0 0 0 0 0 0 0 0

0 2 53 0 0 0 0 0 0 0 0 0 0 0

0 2 58 0 0 0 0 0 0 0 0 0 3 0

0 2 56 0 0 0 0 0 0 0 0 0 0 0

0 2 56 0 0 0 0 0 0 0 0 0 0 0

0 2 56 0 0 0 0 0 0 0 0 0 0 0

0 2 63 0 0 0 0 0 0 0 0 0 0 0

0 2 64 0 0 0 1 0 0 0 0 0 0 0

0 2 54 0 0 0 0 0 0 0 0 0 0 0

0 2 60 0 0 0 1 0 0 0 0 0 0 0

0 2 58 0 0 0 1 0 0 0 0 0 0 0

0 2 39 0 0 0 0 0 0 0 0 0 3 0

0 2 56 0 0 0 1 0 0 0 0 0 0 0

0 2 50 0 0 0 1 0 0 0 0 0 0 0

0 2 47 0 0 0 1 0 0 0 0 0 0 0

0 2 52 0 0 0 1 0 0 0 0 0 0 0

0 2 44 0 0 0 1 0 0 0 0 0 0 0

0 2 52 0 0 0 0 0 0 0 0 0 0 0

0 2 57 0 0 0 0 0 0 0 0 0 0 0

0 2 40 0 0 0 0 0 0 0 0 0 0 0

0 2 43 0 0 0 0 0 0 0 0 0 0 0

0 2 54 0 0 0 0 0 0 0 0 0 0 0

0 2 52 0 0 0 0 0 0 0 0 0 0 0

0 2 56 0 0 0 0 0 0 0 0 0 0 0

0 2 63 0 0 0 0 0 0 0 0 0 0 0

0 2 44 0 0 0 0 0 0 0 0 0 0 0

0 2 50 0 0 0 0 0 0 0 0 0 0 0

0 2 59 0 0 0 0 0 0 0 0 0 0 0

0 2 57 0 0 0 0 0 0 0 0 0 0 0

0 2 56 0 0 0 0 0 0 0 0 0 0 0

0 2 57 0 0 0 0 0 0 0 0 0 0 0

0 2 55 0 0 0 0 0 0 0 0 0 0 0

0 2 58 0 0 0 0 0 0 0 0 0 0 0

0 2 41 0 0 0 0 0 0 0 0 0 0 0

0 2 63 0 0 0 0 0 0 0 0 0 0 0

0 2 56 0 0 0 0 0 0 0 0 0 0 0

0 2 20 0 0 0 0 0 0 0 0 0 0 0

0 2 42 0 0 0 0 0 0 0 0 0 0 0

0 2 39 0 0 0 0 0 0 0 0 0 0 0

0 2 57 0 0 0 0 0 0 0 0 0 0 0

0 2 57 0 0 0 0 0 0 0 0 0 0 0

0 2 58 0 0 0 0 0 0 0 0 0 2 0

0 2 58 0 0 0 0 0 0 0 0 0 0 0

0 2 42 0 0 0 0 0 0 0 0 0 3 0

0 2 52 0 0 0 0 0 0 0 0 0 0 0

0 2 31 0 0 0 0 0 0 0 0 0 0 0

0 2 58 0 0 0 0 0 0 0 0 0 0 0

0 2 54 0 0 0 0 0 0 0 0 0 0 0

0 2 61 0 0 0 0 0 0 0 0 0 0 0

0 2 38 0 0 0 0 0 0 0 0 0 0 0

0 2 32 0 0 0 0 0 0 0 0 0 0 0

0 2 42 0 0 0 0 0 0 0 0 0 0 0

0 2 41 0 0 0 0 0 0 0 0 0 0 0

0 2 53 0 0 0 0 0 0 0 0 0 0 0

0 2 29 0 0 0 0 0 0 0 0 0 0 0

0 2 15 0 0 0 0 0 0 0 0 0 0 0

0 2 62 0 0 0 0 0 0 0 0 0 0 0

0 2 54 0 0 0 0 0 0 0 0 0 0 0

0 2 42 0 0 0 0 0 0 0 0 0 0 0

0 2 67 0 0 0 0 0 0 0 0 0 0 0

0 2 46 0 0 0 0 0 0 0 0 0 0 0

0 2 59 0 0 0 0 0 0 0 0 0 0 0

0 2 54 0 0 0 0 0 0 0 0 0 0 0

0 2 49 0 0 0 0 0 0 0 0 0 0 0

0 2 45 0 0 0 0 0 0 0 0 0 0 0

0 2 37 0 0 0 0 0 0 0 0 0 0 0

0 2 33 0 0 0 0 0 0 0 0 0 0 0

0 2 29 0 0 0 0 0 0 0 0 0 0 0

0 2 52 0 0 0 0 0 0 0 0 0 0 0

0 2 28 0 0 0 0 0 0 0 0 0 0 0

0 2 38 0 0 0 0 0 0 0 0 0 0 0

0 2 53 0 0 0 0 0 0 0 0 0 0 0

0 2 36 0 0 0 0 0 0 0 0 0 0 0

0 2 32 0 0 0 0 0 0 0 0 0 0 0

0 2 35 0 0 0 0 0 0 0 0 0 0 0

0 2 35 0 0 0 0 0 0 0 0 0 0 0

0 2 58 0 0 0 0 0 0 0 0 0 0 0

0 2 41 0 0 0 0 0 0 0 0 0 0 0

0 2 37 0 0 0 0 0 0 0 0 0 0 0

0 2 37 0 0 0 0 0 0 0 0 0 0 0

0 2 36 0 0 0 0 0 0 0 0 0 0 0

0 2 23 0 0 0 0 0 0 0 0 0 0 0

0 2 44 0 0 0 0 0 0 0 0 0 0 0

0 2 41 0 0 0 0 0 0 0 0 0 0 0

0 2 58 0 0 0 0 0 0 0 0 0 0 0

0 2 59 0 0 0 0 0 0 0 0 0 0 0

0 2 38 0 0 0 0 0 0 0 0 0 0 0

0 2 48 0 0 0 0 0 0 0 0 0 3 0

0 2 31 0 0 0 0 0 0 0 0 0 1 0

0 2 38 0 0 0 0 0 0 0 0 0 1 0

0 2 37 0 0 0 0 0 0 0 0 0 2 0

0 2 80 0 0 0 0 0 0 0 0 0 2 0

0 2 50 0 0 0 0 0 0 0 0 0 2 0

0 2 40 0 0 0 0 0 0 0 0 0 2 0

0 2 41 0 0 0 0 0 0 0 0 0 2 0

0 2 26 0 0 0 0 0 0 0 0 0 2 0

0 2 35 0 0 0 0 0 0 0 0 0 2 0

0 2 47 0 0 0 0 0 0 0 0 0 2 0

0 2 53 0 0 0 0 0 0 0 0 0 2 0

0 2 22 0 0 0 0 0 0 0 0 0 2 0

0 2 23 0 0 0 0 0 0 0 0 0 2 0

0 2 36 0 0 0 0 0 0 0 0 0 2 0

0 2 35 0 0 0 0 0 0 0 0 0 2 0

0 2 36 0 0 0 0 0 0 0 0 0 2 0

0 2 43 0 0 0 0 0 0 0 0 0 2 0

0 2 25 0 0 0 0 0 0 0 0 0 2 0

0 2 59 0 0 0 0 0 0 0 0 0 2 0

0 2 55 0 0 0 0 0 0 0 0 0 3 0

0 2 49 0 0 0 0 0 0 0 0 0 3 0

0 2 58 0 0 0 0 0 0 0 0 0 3 0

0 2 60 0 0 0 0 0 0 0 0 0 3 0

0 2 22 0 0 0 0 0 0 0 0 0 3 0

0 2 48 0 0 0 0 0 0 0 0 0 4 0

0 2 58 0 0 0 0 0 0 0 0 0 4 0

0 2 38 0 0 0 0 0 0 0 0 0 4 0

0 2 39 0 0 0 0 0 0 0 0 0 4 0

0 2 56 0 0 0 0 0 0 0 0 0 0 0

0 2 84 0 0 0 0 0 0 0 0 0 0 0

0 2 46 0 0 0 0 0 0 0 0 0 0 0

0 2 65 0 0 0 0 0 0 0 0 0 0 0

0 2 64 0 0 0 0 0 0 0 0 0 0 0

0 2 62 0 0 0 0 0 0 0 0 0 0 0

0 2 74 0 0 0 0 0 0 0 0 0 0 0

0 2 58 0 0 0 0 0 0 0 0 0 0 0

0 2 72 0 0 0 0 0 0 0 0 0 0 0

0 2 58 0 0 0 0 0 0 0 0 0 0 0

0 2 64 0 0 0 0 0 0 0 0 0 0 0

0 2 47 0 0 0 0 0 0 0 0 0 0 0

0 2 63 0 0 0 0 0 0 0 0 0 0 0

0 2 62 0 0 0 0 0 0 0 0 0 0 0

0 2 61 0 0 0 0 0 0 0 0 0 0 0

0 2 82 0 0 0 0 0 0 0 0 0 2 0

0 2 52 0 0 0 0 0 0 0 0 0 2 0

0 2 55 0 0 0 0 0 0 0 0 0 3 0

0 2 62 0 0 0 0 0 0 0 0 0 3 0

0 2 56 0 0 0 0 0 0 0 0 0 3 0

0 2 78 0 0 0 0 0 0 0 0 0 3 0

0 2 45 0 0 0 0 0 0 0 0 0 3 0

0 2 74 0 0 0 0 0 0 0 0 0 3 0

0 2 52 0 0 0 0 0 0 0 0 0 3 0

0 2 57 0 0 0 0 0 0 0 0 0 3 0

0 2 60 0 0 0 0 0 0 0 0 0 3 0

0 2 57 0 0 0 0 0 0 0 0 0 3 0

0 2 72 0 0 0 0 0 0 0 0 0 3 0

0 2 60 0 0 0 0 0 0 0 0 0 3 0

0 2 75 0 0 0 0 0 0 0 0 0 3 0

0 2 66 0 0 0 0 0 0 0 0 0 3 0

0 2 76 0 0 0 0 0 0 0 0 0 3 0

0 2 76 0 0 0 0 0 0 0 0 0 3 0

0 2 76 0 0 0 0 0 0 0 0 0 4 0

0 2 50 0 0 0 0 0 0 0 0 0 4 0

0 2 33 0 0 0 0 0 0 0 0 0 4 0

0 2 74 0 0 0 0 0 0 0 0 0 4 0

0 2 69 0 0 0 0 0 0 0 0 0 0 0

0 2 60 0 0 0 0 0 0 0 0 0 0 0

0 2 76 0 0 0 0 0 0 0 0 0 0 0

0 2 45 0 0 0 0 0 0 0 0 0 0 0

0 2 45 0 0 0 0 0 0 0 0 0 0 0

0 2 65 0 0 0 0 0 0 0 0 0 0 0

0 2 71 0 0 0 0 0 0 0 0 0 0 0

0 2 57 0 0 0 0 0 0 0 0 0 0 0

1 1 38 0 1 0 0 1 0 0 0 1 4 1

0 1 25 1 1 0 0 1 0 0 0 1 4 1

0 2 45 0 1 0 0 1 0 0 0 1 0 1

1 1 74 1 1 0 0 0 0 1 0 1 4 1

1 1 52 0 1 0 0 0 0 1 0 1 2 1

1 2 82 0 1 0 0 0 0 1 0 1 4 1

0 1 70 1 1 1 0 0 0 0 1 1 3 1

0 1 72 0 1 0 0 0 0 0 1 1 3 1

1 2 45 0 1 0 0 0 0 0 1 1 3 1

0 1 57 1 1 0 0 0 0 0 1 1 4 1

0 2 69 0 1 0 0 0 0 0 1 1 2 1

1 1 58 1 1 0 0 0 0 0 1 1 1 1

1 1 60 0 1 0 0 0 0 0 1 1 3 1

1 2 51 1 1 0 0 0 0 0 1 1 3 1

0 1 58 0 1 0 0 0 0 0 1 1 4 1

0 1 60 1 1 0 0 0 0 0 1 1 0 1

0 2 73 0 1 0 0 0 0 0 1 1 0 1

0 2 59 0 1 0 0 0 0 0 1 1 2 1

0 2 59 0 1 0 1 0 0 0 1 1 3 1

0 2 33 1 1 0 0 0 0 0 1 1 0 1

0 2 60 0 1 0 0 0 0 0 1 1 0 1

0 2 55 0 1 0 0 0 0 0 1 1 0 1

0 1 65 1 1 1 0 0 0 0 0 1 4 1

1 2 61 0 1 1 0 0 0 0 0 1 4 1

0 2 56 0 1 1 0 0 0 0 0 1 0 1

0 2 47 0 1 1 0 0 0 0 0 1 0 1

0 2 66 0 1 1 0 0 0 0 0 1 0 1

0 1 81 1 1 1 0 0 0 0 0 1 2 1

1 1 57 0 1 1 0 0 0 0 0 1 3 1

0 2 54 0 1 1 0 0 0 0 0 1 2 1

0 2 54 1 1 1 0 0 0 0 0 1 0 1

0 2 63 0 1 1 0 0 0 0 0 1 4 1

0 2 47 0 1 1 0 0 0 0 0 1 0 1

1 1 72 0 1 0 0 0 0 0 0 1 0 1

1 1 76 1 1 0 0 0 0 0 0 1 4 1

0 1 66 1 1 0 0 0 0 0 0 1 2 1

0 2 84 1 1 0 0 0 0 0 0 1 0 1

1 1 58 0 1 0 0 0 0 0 0 1 0 1

1 1 72 0 1 0 0 0 0 0 0 1 0 1

1 1 80 0 1 0 0 0 0 0 0 1 0 1

1 1 70 0 1 0 0 0 0 0 0 1 4 1

1 1 70 0 1 0 0 0 0 0 0 1 0 1

1 1 63 0 1 0 0 0 0 0 0 1 0 1

1 1 71 0 1 0 0 0 0 0 0 1 3 1

1 1 70 0 1 0 0 0 0 0 0 1 4 1

1 2 86 0 1 0 0 0 0 0 0 1 0 1

1 2 70 0 1 0 1 0 0 0 0 1 4 1

1 2 65 0 1 0 0 0 0 0 0 1 0 1

1 2 73 0 1 0 0 0 0 0 0 1 4 1

1 2 72 0 1 0 0 0 0 0 0 1 2 1

0 1 62 0 1 0 0 0 0 0 0 1 0 1

0 1 55 1 1 0 0 0 0 0 0 1 3 1

0 1 75 0 1 0 0 0 0 0 0 1 0 1

0 1 75 0 1 0 1 0 0 0 0 1 2 1

0 1 85 1 1 0 0 0 0 0 0 1 0 1

0 1 80 0 1 0 0 0 0 0 0 1 4 1

0 1 70 0 1 0 0 0 0 0 0 1 0 1

0 1 47 1 1 0 0 0 0 0 0 1 0 1

0 1 61 0 1 0 0 0 0 0 0 1 3 1

0 1 83 1 1 0 0 0 0 0 0 1 0 1

0 1 69 0 1 0 0 0 0 0 0 1 0 1

0 1 39 1 1 0 0 0 0 0 0 1 0 1

0 2 80 0 1 0 0 0 0 0 0 1 3 1

0 2 62 0 1 0 0 0 0 0 0 1 0 1

0 2 70 0 1 0 0 0 0 0 0 1 0 1

0 2 68 1 1 0 0 0 0 0 0 1 0 1

0 2 79 0 1 0 0 0 0 0 0 1 0 1

0 2 81 0 1 0 0 0 0 0 0 1 0 1

0 2 72 0 1 0 0 0 0 0 0 1 0 1

0 2 78 1 1 0 0 0 0 0 0 1 2 1

0 2 67 0 1 0 0 0 0 0 0 1 0 1

0 2 50 0 1 0 0 0 0 0 0 1 4 1

1 1 81 1 1 0 0 0 0 0 0 1 4 1

1 1 77 1 1 0 0 0 0 0 0 1 0 1

1 1 85 1 1 0 0 0 0 0 0 1 4 1

1 2 61 1 1 0 0 0 0 0 0 1 0 1

1 2 69 1 1 0 0 0 0 0 0 1 3 1

1 2 72 1 1 0 0 0 0 0 0 1 0 1

1 2 76 1 1 0 0 0 0 0 0 1 4 1

0 1 51 1 1 0 0 0 0 0 0 1 2 1

0 1 38 1 1 0 0 0 0 0 0 1 0 1

0 1 76 1 1 0 0 0 0 0 0 1 2 1

0 1 85 1 1 0 0 0 0 0 0 1 0 1

0 1 87 1 1 0 0 0 0 0 0 1 4 1

0 1 53 1 1 0 0 0 0 0 0 1 0 1

0 1 91 1 1 0 0 0 0 0 0 1 2 1

0 1 52 1 1 0 0 0 0 0 0 1 2 1

0 1 77 1 1 0 0 0 0 0 0 1 0 1

0 2 69 1 1 0 0 0 0 0 0 1 0 1

0 2 62 1 1 0 0 0 0 0 0 1 3 1

1 1 75 0 1 0 0 0 0 0 0 1 0 1

1 1 22 0 1 0 0 0 0 0 0 1 0 1

1 1 68 0 1 0 0 0 0 0 0 1 0 1

1 1 77 0 1 0 0 0 0 0 0 1 3 1

1 1 83 0 1 0 0 0 0 0 0 1 2 1

1 1 34 0 1 0 0 0 0 0 0 1 3 1

1 1 65 0 1 0 0 0 0 0 0 1 4 1

1 2 77 0 1 0 0 0 0 0 0 1 0 1

1 2 45 0 1 0 0 0 0 0 0 1 0 1

1 2 79 0 1 0 0 0 0 0 0 1 0 1

1 2 76 0 1 0 0 0 0 0 0 1 3 1

0 1 62 0 1 0 0 0 0 0 0 1 4 1

0 1 72 0 1 0 0 0 0 0 0 1 3 1

0 1 82 0 1 0 0 0 0 0 0 1 3 1

0 1 52 0 1 0 0 0 0 0 0 1 0 1

0 1 57 0 1 0 0 0 0 0 0 1 0 1

0 1 74 0 1 0 0 0 0 0 0 1 3 1

0 1 50 0 1 0 0 0 0 0 0 1 0 1

0 1 74 0 1 0 0 0 0 0 0 0 4 1

0 1 56 0 1 0 0 0 0 0 0 0 0 1

0 1 45 0 1 0 0 0 0 0 0 0 0 1

0 1 80 0 1 0 1 0 0 0 0 0 2 1

0 1 74 0 1 0 1 0 0 0 0 0 0 1

0 1 65 0 1 0 0 0 0 0 0 0 0 1

0 1 57 0 1 0 0 0 0 0 0 0 0 1

0 1 76 0 1 0 0 0 0 0 0 0 3 1

0 1 76 0 1 0 0 0 0 0 0 0 4 1

0 1 78 1 1 0 0 0 0 0 0 0 0 1

0 2 56 1 1 0 0 0 0 0 0 0 3 1

1 1 47 0 1 0 1 0 0 0 0 0 0 1

1 1 93 0 1 0 0 0 0 0 0 0 2 1

1 2 53 0 1 0 0 0 0 0 0 0 0 1

0 1 79 0 1 0 0 0 0 0 0 0 2 1

0 1 79 0 1 0 0 0 0 0 0 0 0 1

0 1 63 0 1 0 0 0 0 0 0 0 0 1

0 1 67 0 1 0 0 0 0 0 0 0 2 1

0 1 61 0 1 0 0 0 0 0 0 0 3 1

0 2 74 0 1 0 0 0 0 0 0 0 0 1

0 2 62 0 1 0 0 0 0 0 0 0 4 1

0 2 37 0 1 0 0 0 0 0 0 0 2 1

0 2 64 0 1 0 0 0 0 0 0 0 0 1

0 2 70 0 1 0 0 0 0 0 0 0 0 1

0 2 69 0 1 0 0 0 0 0 0 0 0 1

0 2 80 0 1 0 0 0 0 0 0 0 4 1

0 2 80 0 1 0 0 0 0 0 0 0 4 1

0 2 76 0 1 0 0 0 0 0 0 0 0 1

0 2 80 0 1 0 1 0 0 0 0 0 3 1

0 2 48 0 1 0 0 0 0 0 0 0 0 1

0 2 78 0 1 0 0 0 0 0 0 0 3 1

0 2 81 0 1 0 0 0 0 0 0 0 0 1

0 2 80 0 1 0 0 0 0 0 0 0 0 1

0 2 67 1 0 0 0 1 0 0 0 1 0 1

0 2 61 0 0 0 0 1 0 0 0 1 4 1

0 2 81 1 0 0 0 1 0 0 0 1 0 1

0 2 87 1 0 0 0 1 0 0 0 1 4 1

0 2 74 1 0 0 0 1 0 0 0 1 0 1

0 2 36 1 0 0 0 1 0 0 0 1 3 1

1 2 58 0 0 0 0 1 0 0 0 1 3 1

0 1 67 0 0 0 0 1 0 0 0 1 2 1

0 2 57 1 0 0 0 1 0 0 0 1 4 1

0 2 48 0 0 1 0 0 0 1 0 1 0 1

0 2 49 0 0 1 0 0 0 1 0 1 4 1

1 1 62 1 0 0 0 0 0 1 0 1 4 1

1 2 82 0 0 0 0 0 0 1 0 1 0 1

0 1 54 0 0 0 0 0 0 1 0 1 0 1

0 1 64 1 0 0 0 0 0 1 0 1 4 1

0 2 72 0 0 0 0 0 0 1 0 1 3 1

0 2 84 1 0 0 0 0 0 1 0 1 0 1

0 2 58 1 0 0 0 0 0 1 0 1 3 1

1 1 77 1 0 0 0 0 0 1 0 1 2 1

1 1 68 1 0 0 0 0 0 1 0 1 0 1

0 1 75 1 0 0 1 0 0 1 0 1 2 1

0 1 73 1 0 0 0 0 0 1 0 1 3 1

0 2 69 1 0 0 0 0 0 1 0 1 3 1

0 2 73 1 0 0 0 0 0 1 0 1 0 1

1 1 53 0 0 0 0 0 0 1 0 1 0 1

1 1 74 0 0 0 0 0 0 1 0 1 0 1

1 1 66 1 0 0 0 0 0 1 0 1 0 1

1 1 60 1 0 0 0 0 0 1 0 1 4 1

1 1 16 0 0 0 0 0 0 1 0 1 0 1

1 1 62 0 0 0 0 0 0 1 0 1 3 1

1 2 64 1 0 0 0 0 0 1 0 1 0 1

0 1 80 1 0 0 0 0 0 1 0 1 0 1

0 1 65 0 0 0 0 0 0 1 0 1 0 1

0 1 62 0 0 0 0 0 0 1 0 1 0 1

0 1 64 1 0 0 0 0 0 1 0 1 4 1

0 1 60 0 0 0 0 0 0 1 0 1 3 1

0 1 60 1 0 0 0 0 0 1 0 1 0 1

0 1 58 0 0 0 0 0 0 1 0 1 0 1

0 1 64 1 0 0 0 0 0 1 0 1 0 1

0 1 84 1 0 0 0 0 0 1 0 1 3 1

0 1 82 0 0 0 0 0 0 1 0 1 0 1

0 1 53 1 0 0 1 0 0 1 0 1 3 1

0 1 57 0 0 0 0 0 0 1 0 1 1 1

0 1 51 1 0 0 0 0 0 1 0 1 0 1

0 1 62 0 0 0 0 0 0 1 0 1 0 1

0 1 86 1 0 0 1 0 0 1 0 1 0 1

0 2 85 1 0 0 0 0 0 1 0 1 0 1

0 2 69 0 0 0 0 0 0 1 0 1 0 1

0 2 60 1 0 0 0 0 0 1 0 1 0 1

0 2 70 0 0 0 0 0 0 1 0 1 0 1

0 2 75 0 0 0 0 0 0 1 0 1 0 1

0 2 48 0 0 1 0 0 0 0 1 1 4 1

0 2 72 1 0 1 0 0 0 0 1 1 0 1

0 1 68 1 0 0 0 0 0 0 1 1 3 1

0 1 54 1 0 0 0 0 0 0 1 1 4 1

1 1 71 1 0 0 0 0 0 0 1 1 0 1

1 2 61 0 0 0 0 0 0 0 1 1 0 1

0 1 67 0 0 0 1 0 0 0 1 1 3 1

0 1 69 0 0 0 0 0 0 0 1 1 3 1

0 1 78 1 0 0 0 0 0 0 1 1 2 1

0 1 50 0 0 0 0 0 0 0 1 1 3 1

0 1 58 0 0 0 0 0 0 0 1 1 0 1

0 1 59 1 0 0 0 0 0 0 1 1 4 1

0 1 67 0 0 0 0 0 0 0 1 1 0 1

0 1 35 1 0 0 0 0 0 0 1 1 0 1

0 1 47 0 0 0 0 0 0 0 1 1 0 1

0 1 78 1 0 0 0 0 0 0 1 1 0 1

0 1 42 0 0 0 0 0 0 0 1 1 0 1

0 1 35 1 0 0 0 0 0 0 1 1 2 1

0 2 61 0 0 0 0 0 0 0 1 1 4 1

0 2 58 0 0 0 0 0 0 0 1 1 2 1

0 2 46 0 0 0 0 0 0 0 1 1 0 1

0 2 76 0 0 0 0 0 0 0 1 1 0 1

0 2 60 1 0 0 0 0 0 0 1 1 0 1

0 2 42 0 0 0 0 0 0 0 1 1 3 1

0 2 59 0 0 0 0 0 0 0 1 1 0 1

0 2 49 1 0 0 0 0 0 0 1 1 0 1

0 2 71 0 0 0 0 0 0 0 1 1 0 1

0 2 49 0 0 0 0 0 0 0 1 1 0 1

0 2 35 1 0 0 0 0 0 0 1 1 0 1

0 2 81 0 0 0 0 0 0 0 1 1 0 1

0 2 64 0 0 0 0 0 0 0 1 1 0 1

0 2 68 1 0 0 0 0 0 0 1 1 4 1

0 2 49 0 0 0 0 0 0 0 1 1 0 1

0 2 58 1 0 0 0 0 0 0 1 1 0 1

0 2 63 0 0 0 0 0 0 0 1 1 1 1

0 1 76 1 0 0 0 0 0 0 1 1 3 1

0 2 64 1 0 0 0 0 0 0 1 1 0 1

0 2 68 1 0 0 0 0 0 0 1 1 3 1

0 2 65 1 0 0 0 0 0 0 1 1 0 1

0 2 57 1 0 0 0 0 0 0 1 1 0 1

0 2 75 1 0 0 0 0 0 0 1 1 3 1

0 2 63 1 0 0 0 0 0 0 1 1 3 1

0 2 51 1 0 0 0 0 0 0 1 1 0 1

1 1 50 0 0 0 0 0 0 0 1 1 0 1

1 1 60 1 0 0 0 0 0 0 1 1 3 1

1 1 65 0 0 0 0 0 0 0 1 1 4 1

1 1 75 0 0 0 0 0 0 0 1 1 0 1

1 1 68 1 0 0 0 0 0 0 1 1 0 1

1 1 53 1 0 0 0 0 0 0 1 1 0 1

1 1 51 0 0 0 0 0 0 0 1 1 0 1

1 2 49 0 0 0 0 0 0 0 1 1 3 1

1 2 60 1 0 0 0 0 0 0 1 1 0 1

1 2 57 0 0 0 0 0 0 0 1 1 0 1

1 2 45 0 0 0 0 0 0 0 1 1 3 1

1 2 29 0 0 0 0 0 0 0 1 1 0 1

0 1 75 1 0 0 0 0 0 0 1 1 0 1

0 1 72 0 0 0 0 0 0 0 1 1 0 1

0 1 56 0 0 0 0 0 0 0 1 1 0 1

0 1 45 0 0 0 0 0 0 0 1 1 0 1

0 1 65 1 0 0 0 0 0 0 1 1 0 1

0 1 51 0 0 0 0 0 0 0 1 1 0 1

0 1 59 1 0 0 0 0 0 0 1 1 0 1

0 1 62 0 0 0 0 0 0 0 1 1 0 1

0 1 58 0 0 0 0 0 0 0 1 1 1 1

0 1 40 1 0 0 0 0 0 0 1 1 0 1

0 1 47 0 0 0 0 0 0 0 1 1 3 1

0 1 64 1 0 0 0 0 0 0 1 1 2 1

0 1 58 0 0 0 0 0 0 0 1 1 0 1

0 1 69 0 0 0 0 0 0 0 1 1 0 1

0 1 56 1 0 0 0 0 0 0 1 1 0 1

0 1 54 0 0 0 0 0 0 0 1 1 0 1

0 1 58 0 0 0 0 0 0 0 1 1 0 1

0 1 46 0 0 0 0 0 0 0 1 1 0 1

0 1 55 0 0 0 0 0 0 0 1 1 0 1

0 1 46 1 0 0 0 0 0 0 1 1 0 1

0 1 54 0 0 0 0 0 0 0 1 1 0 1

0 1 51 0 0 0 0 0 0 0 1 1 4 1

0 2 31 1 0 0 0 0 0 0 1 1 4 1

0 2 59 0 0 0 0 0 0 0 1 1 3 1

0 2 64 0 0 0 0 0 0 0 1 1 0 1

0 2 63 0 0 0 0 0 0 0 1 1 3 1

0 2 51 0 0 0 0 0 0 0 1 1 4 1

0 2 48 1 0 0 0 0 0 0 1 1 3 1

0 2 43 0 0 0 0 0 0 0 1 1 2 1

0 2 70 0 0 0 0 0 0 0 1 1 0 1

0 2 61 1 0 0 0 0 0 0 1 1 0 1

0 2 40 0 0 0 0 0 0 0 1 1 3 1

0 2 62 1 0 0 0 0 0 0 1 1 0 1

0 2 71 0 0 0 0 0 0 0 1 1 0 1

0 2 45 1 0 0 0 0 0 0 1 1 2 1

0 2 35 0 0 0 1 0 0 0 1 1 2 1

0 2 41 0 0 0 0 0 0 0 1 1 4 1

0 2 56 1 0 0 0 0 0 0 1 1 2 1

0 2 57 0 0 0 0 0 0 0 1 1 0 1

0 2 60 0 0 0 0 0 0 0 1 1 2 1

0 2 39 0 0 0 0 0 0 0 1 1 3 1

0 2 75 0 0 0 0 0 0 0 1 1 0 1

0 2 67 0 0 0 0 0 0 0 1 1 0 1

0 2 74 0 0 0 1 0 0 0 1 1 3 1

0 2 48 0 0 0 0 0 0 0 1 1 4 1

0 2 64 0 0 0 0 0 0 0 1 1 0 1

0 2 46 0 0 0 0 0 0 0 1 1 0 1

0 2 33 0 0 0 0 0 0 0 1 1 4 1

0 2 43 1 0 0 0 0 0 0 1 1 0 1

0 2 49 0 0 0 0 0 0 0 1 1 3 1

0 2 65 0 0 0 0 0 0 0 1 1 0 1

0 2 71 0 0 0 0 0 0 0 1 1 4 1

0 2 49 1 0 0 0 0 0 0 1 1 3 1

0 2 48 0 0 0 0 0 0 0 1 1 0 1

0 2 50 0 0 0 0 0 0 0 1 1 0 1

0 2 55 0 0 0 0 0 0 0 1 1 0 1

0 1 78 0 0 1 0 0 0 0 0 1 2 1

0 2 48 1 0 1 0 0 0 0 0 1 4 1

0 2 53 1 0 1 0 0 0 0 0 1 0 1

1 1 52 0 0 1 1 0 0 0 0 1 3 1

1 1 45 0 0 1 0 0 0 0 0 1 2 1

1 2 72 0 0 1 0 0 0 0 0 1 0 1

1 2 49 0 0 1 0 0 0 0 0 1 1 1

1 2 79 0 0 1 0 0 0 0 0 1 0 1

1 2 53 0 0 1 0 0 0 0 0 1 0 1

1 2 45 0 0 1 0 0 0 0 0 1 2 1

1 2 47 0 0 1 0 0 0 0 0 1 4 1

1 2 63 0 0 1 0 0 0 0 0 1 0 1

0 1 39 0 0 1 0 0 0 0 0 1 0 1

0 1 69 0 0 1 0 0 0 0 0 1 3 1

0 1 88 0 0 1 0 0 0 0 0 1 0 1

0 2 55 0 0 1 0 0 0 0 0 1 0 1

0 2 79 0 0 1 0 0 0 0 0 1 2 1

0 2 46 0 0 1 0 0 0 0 0 1 0 1

0 2 58 0 0 1 0 0 0 0 0 1 4 1

0 2 82 0 0 1 0 0 0 0 0 1 0 1

0 2 53 0 0 1 0 0 0 0 0 1 2 1

0 2 66 0 0 1 0 0 0 0 0 1 0 1

0 2 58 0 0 1 0 0 0 0 0 1 3 1

0 2 69 0 0 1 0 0 0 0 0 1 4 1

1 1 77 1 0 1 0 0 0 0 0 1 2 1

1 2 85 1 0 1 0 0 0 0 0 1 0 1

1 2 88 1 0 1 0 0 0 0 0 1 4 1

0 1 61 1 0 1 0 0 0 0 0 1 0 1

0 2 52 1 0 1 0 0 0 0 0 1 0 1

0 2 79 1 0 1 0 0 0 0 0 1 0 1

1 1 61 0 0 1 0 0 0 0 0 1 0 1

1 1 66 0 0 1 0 0 0 0 0 1 0 1

1 1 57 0 0 1 0 0 0 0 0 1 0 1

1 1 50 0 0 1 0 0 0 0 0 1 3 1

1 1 67 0 0 1 0 0 0 0 0 1 0 1

1 1 78 0 0 1 0 0 0 0 0 1 3 1

1 1 57 0 0 1 0 0 0 0 0 1 2 1

1 1 59 0 0 1 0 0 0 0 0 1 3 1

1 2 49 0 0 1 0 0 0 0 0 1 0 1

1 2 69 0 0 1 0 0 0 0 0 1 0 1

1 2 76 0 0 1 0 0 0 0 0 1 0 1

1 2 80 0 0 1 0 0 0 0 0 1 0 1

1 2 48 0 0 1 0 0 0 0 0 1 0 1

1 2 61 0 0 1 0 0 0 0 0 1 0 1

1 2 65 0 0 1 0 0 0 0 0 1 3 1

1 2 62 0 0 1 0 0 0 0 0 1 0 1

0 1 35 0 0 1 0 0 0 0 0 1 0 1

0 1 75 0 0 1 0 0 0 0 0 1 0 1

0 1 74 0 0 1 0 0 0 0 0 1 0 1

0 1 59 0 0 1 0 0 0 0 0 1 0 1

0 1 52 0 0 1 0 0 0 0 0 1 0 1

0 1 76 0 0 1 0 0 0 0 0 1 2 1

0 1 31 0 0 1 0 0 0 0 0 1 2 1

0 1 86 0 0 1 0 0 0 0 0 1 0 1

0 1 81 0 0 1 0 0 0 0 0 1 0 1

0 1 69 0 0 1 0 0 0 0 0 1 0 1

0 1 77 0 0 1 0 0 0 0 0 1 0 1

0 2 55 0 0 1 0 0 0 0 0 1 3 1

0 2 56 0 0 1 0 0 0 0 0 1 4 1

0 2 80 0 0 1 0 0 0 0 0 1 0 1

0 2 70 0 0 1 0 0 0 0 0 1 1 1

0 2 71 0 0 1 0 0 0 0 0 1 3 1

0 2 69 0 0 1 0 0 0 0 0 1 0 1

0 2 52 0 0 1 0 0 0 0 0 1 0 1

0 2 41 0 0 1 0 0 0 0 0 1 0 1

0 2 56 0 0 1 0 0 0 0 0 1 0 1

0 2 59 0 0 1 0 0 0 0 0 1 0 1

0 2 48 0 0 1 0 0 0 0 0 1 0 1

0 2 62 0 0 1 0 0 0 0 0 1 0 1

0 2 49 0 0 1 0 0 0 0 0 1 3 1

0 2 57 0 0 1 0 0 0 0 0 1 0 1

0 2 47 0 0 1 0 0 0 0 0 1 0 1

0 2 59 0 0 1 0 0 0 0 0 1 0 1

0 2 57 0 0 1 0 0 0 0 0 1 0 1

0 2 53 0 0 1 0 0 0 0 0 1 3 1

0 2 54 0 0 1 0 0 0 0 0 1 2 1

0 2 56 0 0 1 1 0 0 0 0 1 4 1

0 2 73 0 0 1 0 0 0 0 0 1 0 1

0 2 48 0 0 1 0 0 0 0 0 1 0 1

0 2 82 0 0 1 0 0 0 0 0 1 3 1

0 2 81 0 0 1 0 0 0 0 0 1 0 1

0 2 77 0 0 1 0 0 0 0 0 1 2 1

0 2 57 0 0 1 0 0 0 0 0 1 0 1

0 2 63 0 0 1 0 0 0 0 0 1 2 1

0 2 66 0 0 1 0 0 0 0 0 1 0 1

0 2 75 0 0 1 0 0 0 0 0 1 0 1

0 2 70 0 0 1 0 0 0 0 0 1 0 1

1 1 76 1 0 0 0 0 0 0 0 0 0 1

1 1 80 1 0 0 0 0 0 0 0 0 4 1

1 1 41 1 0 0 0 0 0 0 0 0 0 1

1 1 83 1 0 0 0 0 0 0 0 0 0 1

1 1 60 1 0 0 0 0 0 0 0 0 0 1

1 1 72 1 0 0 0 0 0 0 0 0 0 1

1 1 74 1 0 0 0 0 0 0 0 0 0 1

1 2 76 1 0 0 0 0 0 0 0 0 1 1

1 2 78 1 0 0 0 0 0 0 0 0 2 1

0 1 74 1 0 0 1 0 0 0 0 0 3 1

0 1 38 1 0 0 0 0 0 0 0 0 0 1

0 1 85 1 0 0 0 0 0 0 0 0 3 1

0 1 82 1 0 0 0 0 0 0 0 0 3 1

0 1 45 1 0 0 0 0 0 0 0 0 4 1

0 1 75 1 0 0 0 0 0 0 0 0 0 1

0 1 65 1 0 0 0 0 0 0 0 0 0 1

0 2 55 1 0 0 0 0 0 0 0 0 0 1

0 2 77 1 0 0 0 0 0 0 0 0 0 1

0 2 58 1 0 0 0 0 0 0 0 0 4 1

0 2 68 1 0 0 0 0 0 0 0 0 0 1

0 2 72 1 0 0 0 0 0 0 0 0 2 1

0 2 59 1 0 0 0 0 0 0 0 0 0 1

0 2 82 1 0 0 0 0 0 0 0 0 2 1

1 1 86 0 0 0 0 0 0 0 0 0 2 1

1 1 84 0 0 0 0 0 0 0 0 0 0 1

1 1 76 0 0 0 0 0 0 0 0 0 0 1

1 1 80 0 0 0 0 0 0 0 0 0 0 1

1 1 48 0 0 0 0 0 0 0 0 0 2 1

1 1 73 0 0 0 1 0 0 0 0 0 0 1

1 1 75 0 0 0 0 0 0 0 0 0 4 1

1 1 67 0 0 0 0 0 0 0 0 0 0 1

1 1 71 0 0 0 0 0 0 0 0 0 2 1

1 1 56 0 0 0 0 0 0 0 0 0 4 1

1 1 75 0 0 0 0 0 0 0 0 0 0 1

1 1 71 0 0 0 0 0 0 0 0 0 0 1

1 1 60 0 0 0 0 0 0 0 0 0 2 1

1 1 72 0 0 0 0 0 0 0 0 0 0 1

1 1 81 0 0 0 0 0 0 0 0 0 3 1

1 1 76 0 0 0 0 0 0 0 0 0 3 1

1 1 68 0 0 0 0 0 0 0 0 0 3 1

1 1 69 0 0 0 0 0 0 0 0 0 0 1

1 1 74 0 0 0 0 0 0 0 0 0 3 1

1 1 75 0 0 0 0 0 0 0 0 0 3 1

1 1 76 0 0 0 0 0 0 0 0 0 0 1

1 2 44 0 0 0 0 0 0 0 0 0 0 1

1 2 76 0 0 0 0 0 0 0 0 0 2 1

1 2 79 0 0 0 0 0 0 0 0 0 0 1

1 2 64 0 0 0 0 0 0 0 0 0 3 1

1 2 62 0 0 0 0 0 0 0 0 0 0 1

1 2 75 0 0 0 0 0 0 0 0 0 2 1

1 2 78 0 0 0 0 0 0 0 0 0 0 1

1 2 89 0 0 0 0 0 0 0 0 0 3 1

1 2 82 0 0 0 0 0 0 0 0 0 3 1

1 2 83 0 0 0 0 0 0 0 0 0 3 1

1 2 61 0 0 0 0 0 0 0 0 0 4 1

1 2 53 0 0 0 0 0 0 0 0 0 0 1

1 2 82 0 0 0 0 0 0 0 0 0 3 1

1 2 84 0 0 0 0 0 0 0 0 0 3 1

1 2 60 0 0 0 0 0 0 0 0 0 3 1

1 2 64 0 0 0 0 0 0 0 0 0 0 1

0 1 38 0 0 0 0 0 0 0 0 0 3 1

0 1 78 0 0 0 0 0 0 0 0 0 2 1

0 1 74 0 0 0 1 0 0 0 0 0 0 1

0 1 59 0 0 0 0 0 0 0 0 0 0 1

0 1 62 0 0 0 0 0 0 0 0 0 4 1

0 1 77 0 0 0 0 0 0 0 0 0 3 1

0 1 57 0 0 0 0 0 0 0 0 0 0 1

0 1 78 0 0 0 0 0 0 0 0 0 2 1

0 1 48 0 0 0 0 0 0 0 0 0 0 1

0 1 75 0 0 0 0 0 0 0 0 0 2 1

0 1 71 0 0 0 0 0 0 0 0 0 0 1

0 1 69 0 0 0 0 0 0 0 0 0 2 1

0 1 55 0 0 0 0 0 0 0 0 0 2 1

0 1 71 0 0 0 0 0 0 0 0 0 4 1

0 1 69 0 0 0 1 0 0 0 0 0 0 1

0 1 44 0 0 0 0 0 0 0 0 0 4 1

0 1 64 0 0 0 0 0 0 0 0 0 0 1

0 1 57 0 0 0 0 0 0 0 0 0 4 1

0 1 68 0 0 0 0 0 0 0 0 0 4 1

0 1 76 0 0 0 0 0 0 0 0 0 2 1

0 1 40 0 0 0 0 0 0 0 0 0 0 1

0 1 71 0 0 0 0 0 0 0 0 0 0 1

0 1 55 0 0 0 0 0 0 0 0 0 0 1

0 1 72 0 0 0 0 0 0 0 0 0 0 1

0 1 48 0 0 0 0 0 0 0 0 0 0 1

0 1 80 0 0 0 0 0 0 0 0 0 0 1

0 1 79 0 0 0 0 0 0 0 0 0 0 1

0 1 65 0 0 0 0 0 0 0 0 0 0 1

0 1 82 0 0 0 0 0 0 0 0 0 0 1

0 1 77 0 0 0 0 0 0 0 0 0 3 1

0 1 63 0 0 0 0 0 0 0 0 0 0 1

0 1 44 0 0 0 0 0 0 0 0 0 0 1

0 1 70 0 0 0 0 0 0 0 0 0 0 1

0 1 75 0 0 0 0 0 0 0 0 0 0 1

0 1 73 0 0 0 0 0 0 0 0 0 0 1

0 1 75 0 0 0 0 0 0 0 0 0 0 1

0 1 53 0 0 0 0 0 0 0 0 0 0 1

0 1 52 0 0 0 0 0 0 0 0 0 0 1

0 1 76 0 0 0 0 0 0 0 0 0 0 1

0 1 81 0 0 0 0 0 0 0 0 0 3 1

0 1 65 0 0 0 0 0 0 0 0 0 0 1

0 1 65 0 0 0 1 0 0 0 0 0 0 1

0 1 57 0 0 0 0 0 0 0 0 0 3 1

0 1 72 0 0 0 0 0 0 0 0 0 0 1

0 1 58 0 0 0 1 0 0 0 0 0 0 1

0 1 54 0 0 0 0 0 0 0 0 0 0 1

0 1 73 0 0 0 0 0 0 0 0 0 0 1

0 1 93 0 0 0 0 0 0 0 0 0 0 1

0 1 71 0 0 0 0 0 0 0 0 0 0 1

0 1 51 0 0 0 0 0 0 0 0 0 0 1

0 1 61 0 0 0 1 0 0 0 0 0 4 1

0 1 64 0 0 0 0 0 0 0 0 0 4 1

0 1 89 0 0 0 0 0 0 0 0 0 0 1

0 1 72 0 0 0 0 0 0 0 0 0 2 1

0 1 50 0 0 0 0 0 0 0 0 0 0 1

0 1 72 0 0 0 0 0 0 0 0 0 0 1

0 1 62 0 0 0 0 0 0 0 0 0 4 1

0 1 62 0 0 0 0 0 0 0 0 0 3 1

0 1 77 0 0 0 0 0 0 0 0 0 2 1

0 1 67 0 0 0 0 0 0 0 0 0 3 1

0 1 73 0 0 0 0 0 0 0 0 0 0 1

0 1 67 0 0 0 0 0 0 0 0 0 0 1

0 1 68 0 0 0 1 0 0 0 0 0 0 1

0 1 70 0 0 0 0 0 0 0 0 0 0 1

0 1 69 0 0 0 0 0 0 0 0 0 4 1

0 1 81 0 0 0 0 0 0 0 0 0 0 1

0 1 82 0 0 0 0 0 0 0 0 0 0 1

0 1 66 0 0 0 0 0 0 0 0 0 0 1

0 1 47 0 0 0 0 0 0 0 0 0 4 1

0 1 79 0 0 0 0 0 0 0 0 0 0 1

0 1 71 0 0 0 0 0 0 0 0 0 3 1

0 1 74 0 0 0 0 0 0 0 0 0 4 1

0 1 74 0 0 0 0 0 0 0 0 0 0 1

0 1 68 0 0 0 0 0 0 0 0 0 0 1

0 1 72 0 0 0 0 0 0 0 0 0 3 1

0 1 84 0 0 0 0 0 0 0 0 0 0 1

0 1 53 0 0 0 0 0 0 0 0 0 3 1

0 1 69 0 0 0 0 0 0 0 0 0 4 1

0 1 65 0 0 0 0 0 0 0 0 0 2 1

0 1 62 0 0 0 0 0 0 0 0 0 0 1

0 1 67 0 0 0 0 0 0 0 0 0 4 1

0 1 78 0 0 0 0 0 0 0 0 0 3 1

0 1 84 0 0 0 0 0 0 0 0 0 3 1

0 1 53 0 0 0 0 0 0 0 0 0 0 1

0 1 79 0 0 0 0 0 0 0 0 0 0 1

0 1 62 0 0 0 0 0 0 0 0 0 0 1

0 2 80 0 0 0 0 0 0 0 0 0 0 1

0 2 61 0 0 0 0 0 0 0 0 0 0 1

0 2 60 0 0 0 0 0 0 0 0 0 0 1

0 2 72 0 0 0 0 0 0 0 0 0 0 1

0 2 69 0 0 0 0 0 0 0 0 0 0 1

0 2 69 0 0 0 0 0 0 0 0 0 3 1

0 2 37 0 0 0 0 0 0 0 0 0 0 1

0 2 32 0 0 0 0 0 0 0 0 0 1 1

0 2 75 0 0 0 0 0 0 0 0 0 3 1

0 2 44 0 0 0 0 0 0 0 0 0 2 1

0 2 66 0 0 0 0 0 0 0 0 0 0 1

0 2 80 0 0 0 0 0 0 0 0 0 0 1

0 2 76 0 0 0 0 0 0 0 0 0 0 1

0 2 76 0 0 0 0 0 0 0 0 0 2 1

0 2 82 0 0 0 0 0 0 0 0 0 0 1

0 2 69 0 0 0 0 0 0 0 0 0 0 1

0 2 86 0 0 0 0 0 0 0 0 0 4 1

0 2 77 0 0 0 0 0 0 0 0 0 4 1

0 2 70 0 0 0 0 0 0 0 0 0 4 1

0 2 59 0 0 0 0 0 0 0 0 0 0 1

0 2 77 0 0 0 0 0 0 0 0 0 3 1

0 2 77 0 0 0 0 0 0 0 0 0 0 1

0 2 78 0 0 0 0 0 0 0 0 0 4 1

0 2 72 0 0 0 0 0 0 0 0 0 2 1

0 2 59 0 0 0 0 0 0 0 0 0 0 1

0 2 56 0 0 0 0 0 0 0 0 0 0 1

0 2 74 0 0 0 0 0 0 0 0 0 0 1

0 2 68 0 0 0 0 0 0 0 0 0 0 1

0 2 77 0 0 0 0 0 0 0 0 0 1 1

0 2 71 0 0 0 0 0 0 0 0 0 0 1

0 2 79 0 0 0 0 0 0 0 0 0 0 1

0 2 65 0 0 0 0 0 0 0 0 0 3 1

0 2 80 0 0 0 0 0 0 0 0 0 0 1

0 2 65 0 0 0 0 0 0 0 0 0 4 1

0 2 64 0 0 0 0 0 0 0 0 0 4 1

0 2 80 0 0 0 0 0 0 0 0 0 4 1

0 2 81 0 0 0 0 0 0 0 0 0 2 1

0 2 83 0 0 0 0 0 0 0 0 0 4 1

0 2 60 0 0 0 0 0 0 0 0 0 2 1

0 2 69 0 0 0 0 0 0 0 0 0 4 1

0 2 91 0 0 0 0 0 0 0 0 0 3 1

0 2 68 0 0 0 0 0 0 0 0 0 0 1

0 2 84 0 0 0 0 0 0 0 0 0 0 1

0 2 79 0 0 0 0 0 0 0 0 0 0 1

0 2 62 0 0 0 0 0 0 0 0 0 4 1

0 2 63 0 0 0 0 0 0 0 0 0 3 1

0 2 87 0 0 0 0 0 0 0 0 0 4 1

0 2 42 0 0 0 0 0 0 0 0 0 3 1

0 2 52 0 0 0 0 0 0 0 0 0 2 1

0 2 79 0 0 0 0 0 0 0 0 0 0 1

0 2 74 0 0 0 0 0 0 0 0 0 0 1

0 2 71 0 0 0 0 0 0 0 0 0 0 1

0 2 74 0 0 0 0 0 0 0 0 0 3 1

0 2 68 0 0 0 0 0 0 0 0 0 3 1

0 2 78 0 0 0 0 0 0 0 0 0 0 1

0 2 81 0 0 0 0 0 0 0 0 0 0 1

0 2 84 0 0 0 0 0 0 0 0 0 0 1

0 2 77 0 0 0 0 0 0 0 0 0 0 1

0 2 62 0 0 0 0 0 0 0 0 0 1 1

0 2 76 0 0 0 0 0 0 0 0 0 0 1

0 2 58 0 0 0 0 0 0 0 0 0 0 1

0 2 73 0 0 0 0 0 0 0 0 0 3 1

0 2 92 0 0 0 0 0 0 0 0 0 3 1

0 2 67 0 0 0 0 0 0 0 0 0 3 1

0 2 61 0 0 0 0 0 0 0 0 0 0 1

0 2 80 0 0 0 0 0 0 0 0 0 3 1

0 2 61 0 0 0 0 0 0 0 0 0 4 1

0 2 85 0 0 0 0 0 0 0 0 0 4 1

0 2 93 0 0 0 0 0 0 0 0 0 0 1

1 1 71 1 0 0 0 0 0 0 0 0 0 1

1 1 30 1 0 0 0 0 0 0 0 0 0 1

1 1 89 1 0 0 0 0 0 0 0 0 0 1

1 1 87 1 0 0 0 0 0 0 0 0 2 1

1 1 73 1 0 0 0 0 0 0 0 0 0 1

1 1 75 1 0 0 0 0 0 0 0 0 2 1

1 1 71 1 0 0 0 0 0 0 0 0 4 1

1 1 68 1 0 0 0 0 0 0 0 0 3 1

1 1 77 1 0 0 0 0 0 0 0 0 0 1

1 1 64 1 0 0 0 0 0 0 0 0 2 1

1 1 89 1 0 0 0 0 0 0 0 0 0 1

1 1 68 1 0 0 0 0 0 0 0 0 2 1

1 1 71 1 0 0 0 0 0 0 0 0 2 1

1 1 68 1 0 0 0 0 0 0 0 0 0 1

1 1 57 1 0 0 0 0 0 0 0 0 3 1

1 1 74 1 0 0 0 0 0 0 0 0 2 1

1 1 87 1 0 0 0 0 0 0 0 0 2 1

1 1 76 1 0 0 0 0 0 0 0 0 3 1

1 1 71 1 0 0 0 0 0 0 0 0 4 1

1 1 63 1 0 0 0 0 0 0 0 0 0 1

1 1 83 1 0 0 0 0 0 0 0 0 0 1

1 1 60 1 0 0 0 0 0 0 0 0 2 1

1 1 66 1 0 0 0 0 0 0 0 0 0 1

1 1 70 1 0 0 0 0 0 0 0 0 0 1

1 1 67 1 0 0 0 0 0 0 0 0 0 1

1 1 84 1 0 0 0 0 0 0 0 0 4 1

1 1 68 1 0 0 0 0 0 0 0 0 2 1

1 1 67 1 0 0 0 0 0 0 0 0 4 1

1 2 88 1 0 0 0 0 0 0 0 0 0 1

1 2 71 1 0 0 0 0 0 0 0 0 0 1

1 2 76 1 0 0 0 0 0 0 0 0 0 1

1 2 87 1 0 0 0 0 0 0 0 0 0 1

1 2 85 1 0 0 0 0 0 0 0 0 3 1

1 2 75 1 0 0 0 0 0 0 0 0 4 1

1 2 69 1 0 0 0 0 0 0 0 0 2 1

1 2 76 1 0 0 0 0 0 0 0 0 2 1

1 2 73 1 0 0 0 0 0 0 0 0 3 1

1 2 78 1 0 0 0 0 0 0 0 0 3 1

1 2 91 1 0 0 0 0 0 0 0 0 3 1

1 2 84 1 0 0 0 0 0 0 0 0 4 1

1 2 77 1 0 0 0 0 0 0 0 0 3 1

1 2 58 1 0 0 0 0 0 0 0 0 0 1

1 2 74 1 0 0 0 0 0 0 0 0 3 1

1 2 78 1 0 0 0 0 0 0 0 0 0 1

1 2 69 1 0 0 0 0 0 0 0 0 0 1

1 2 90 1 0 0 0 0 0 0 0 0 4 1

1 2 73 1 0 0 0 0 0 0 0 0 3 1

1 2 65 1 0 0 0 0 0 0 0 0 0 1

1 2 81 1 0 0 0 0 0 0 0 0 0 1

0 1 55 1 0 0 0 0 0 0 0 0 3 1

0 1 37 1 0 0 0 0 0 0 0 0 0 1

0 1 69 1 0 0 0 0 0 0 0 0 0 1

0 1 32 1 0 0 0 0 0 0 0 0 4 1

0 1 80 1 0 0 0 0 0 0 0 0 3 1

0 1 77 1 0 0 0 0 0 0 0 0 3 1

0 1 71 1 0 0 0 0 0 0 0 0 4 1

0 1 80 1 0 0 0 0 0 0 0 0 0 1

0 1 79 1 0 0 0 0 0 0 0 0 4 1

0 1 66 1 0 0 0 0 0 0 0 0 0 1

0 1 73 1 0 0 0 0 0 0 0 0 4 1

0 1 80 1 0 0 0 0 0 0 0 0 0 1

0 1 54 1 0 0 0 0 0 0 0 0 0 1

0 1 68 1 0 0 0 0 0 0 0 0 0 1

0 1 71 1 0 0 0 0 0 0 0 0 0 1

0 1 85 1 0 0 0 0 0 0 0 0 3 1

0 1 74 1 0 0 0 0 0 0 0 0 4 1

0 1 59 1 0 0 0 0 0 0 0 0 0 1

0 1 62 1 0 0 0 0 0 0 0 0 0 1

0 1 67 1 0 0 0 0 0 0 0 0 3 1

0 1 75 1 0 0 0 0 0 0 0 0 3 1

0 1 65 1 0 0 0 0 0 0 0 0 0 1

0 1 61 1 0 0 0 0 0 0 0 0 0 1

0 1 70 1 0 0 0 0 0 0 0 0 1 1

0 1 76 1 0 0 0 0 0 0 0 0 0 1

0 1 63 1 0 0 1 0 0 0 0 0 4 1

0 1 77 1 0 0 0 0 0 0 0 0 0 1

0 1 71 1 0 0 0 0 0 0 0 0 3 1

0 1 47 1 0 0 0 0 0 0 0 0 0 1

0 1 67 1 0 0 0 0 0 0 0 0 0 1

0 1 72 1 0 0 0 0 0 0 0 0 0 1

0 1 78 1 0 0 0 0 0 0 0 0 4 1

0 1 70 1 0 0 0 0 0 0 0 0 0 1

0 1 68 1 0 0 0 0 0 0 0 0 4 1

0 1 82 1 0 0 0 0 0 0 0 0 4 1

0 1 72 1 0 0 0 0 0 0 0 0 0 1

0 1 82 1 0 0 0 0 0 0 0 0 3 1

0 1 70 1 0 0 0 0 0 0 0 0 0 1

0 1 66 1 0 0 0 0 0 0 0 0 2 1

0 1 81 1 0 0 0 0 0 0 0 0 4 1

0 1 73 1 0 0 0 0 0 0 0 0 2 1

0 1 66 1 0 0 0 0 0 0 0 0 3 1

0 1 66 1 0 0 0 0 0 0 0 0 4 1

0 1 68 1 0 0 0 0 0 0 0 0 1 1

0 1 77 1 0 0 0 0 0 0 0 0 2 1

0 1 89 1 0 0 1 0 0 0 0 0 2 1

0 1 75 1 0 0 0 0 0 0 0 0 3 1

0 1 68 1 0 0 1 0 0 0 0 0 0 1

0 1 62 1 0 0 0 0 0 0 0 0 0 1

0 1 72 1 0 0 0 0 0 0 0 0 2 1

0 1 80 1 0 0 0 0 0 0 0 0 3 1

0 1 79 1 0 0 0 0 0 0 0 0 0 1

0 1 78 1 0 0 0 0 0 0 0 0 0 1

0 1 60 1 0 0 0 0 0 0 0 0 0 1

0 1 67 1 0 0 0 0 0 0 0 0 3 1

0 1 57 1 0 0 0 0 0 0 0 0 4 1

0 1 65 1 0 0 0 0 0 0 0 0 0 1

0 1 67 1 0 0 0 0 0 0 0 0 0 1

0 1 58 1 0 0 0 0 0 0 0 0 2 1

0 1 76 1 0 0 0 0 0 0 0 0 3 1

0 1 68 1 0 0 0 0 0 0 0 0 4 1

0 1 70 1 0 0 0 0 0 0 0 0 0 1

0 1 66 1 0 0 0 0 0 0 0 0 3 1

0 1 67 1 0 0 0 0 0 0 0 0 0 1

0 1 62 1 0 0 0 0 0 0 0 0 0 1

0 1 83 1 0 0 0 0 0 0 0 0 2 1

0 1 45 1 0 0 0 0 0 0 0 0 2 1

0 1 45 1 0 0 0 0 0 0 0 0 0 1

0 1 61 1 0 0 0 0 0 0 0 0 0 1

0 1 66 1 0 0 0 0 0 0 0 0 0 1

0 1 61 1 0 0 0 0 0 0 0 0 3 1

0 1 66 1 0 0 0 0 0 0 0 0 1 1

0 1 79 1 0 0 0 0 0 0 0 0 0 1

0 1 65 1 0 0 0 0 0 0 0 0 0 1

0 1 77 1 0 0 0 0 0 0 0 0 3 1

0 1 82 1 0 0 0 0 0 0 0 0 0 1

0 1 62 1 0 0 0 0 0 0 0 0 4 1

0 1 71 1 0 0 0 0 0 0 0 0 0 1

0 1 80 1 0 0 0 0 0 0 0 0 3 1

0 1 77 1 0 0 0 0 0 0 0 0 0 1

0 2 52 1 0 0 0 0 0 0 0 0 0 1

0 2 72 1 0 0 0 0 0 0 0 0 0 1

0 2 69 1 0 0 0 0 0 0 0 0 1 1

0 2 72 1 0 0 0 0 0 0 0 0 3 1

0 2 65 1 0 0 0 0 0 0 0 0 0 1

0 2 82 1 0 0 0 0 0 0 0 0 0 1

0 2 80 1 0 0 0 0 0 0 0 0 0 1

0 2 84 1 0 0 0 0 0 0 0 0 0 1

0 2 67 1 0 0 0 0 0 0 0 0 0 1

0 2 82 1 0 0 0 0 0 0 0 0 4 1

0 2 77 1 0 0 0 0 0 0 0 0 4 1

0 2 76 1 0 0 0 0 0 0 0 0 3 1

0 2 79 1 0 0 0 0 0 0 0 0 0 1

0 2 77 1 0 0 0 0 0 0 0 0 0 1

0 2 76 1 0 0 0 0 0 0 0 0 0 1

0 2 69 1 0 0 0 0 0 0 0 0 0 1

0 2 78 1 0 0 0 0 0 0 0 0 0 1

0 2 54 1 0 0 0 0 0 0 0 0 2 1

0 2 77 1 0 0 0 0 0 0 0 0 0 1

0 2 76 1 0 0 0 0 0 0 0 0 0 1

0 2 77 1 0 0 0 0 0 0 0 0 4 1

0 2 61 1 0 0 0 0 0 0 0 0 0 1

0 2 85 1 0 0 0 0 0 0 0 0 4 1

0 2 68 1 0 0 0 0 0 0 0 0 3 1

0 2 75 1 0 0 0 0 0 0 0 0 0 1

0 2 78 1 0 0 0 0 0 0 0 0 3 1

0 2 80 1 0 0 0 0 0 0 0 0 4 1

0 2 73 1 0 0 0 0 0 0 0 0 0 1

0 2 62 1 0 0 0 0 0 0 0 0 3 1

0 2 63 1 0 0 0 0 0 0 0 0 0 1

0 2 99 1 0 0 0 0 0 0 0 0 3 1

0 2 80 1 0 0 0 0 0 0 0 0 0 1

0 2 76 1 0 0 0 0 0 0 0 0 3 1

0 2 66 1 0 0 0 0 0 0 0 0 0 1

0 2 74 1 0 0 0 0 0 0 0 0 0 1

0 2 63 1 0 0 0 0 0 0 0 0 0 1

0 2 82 1 0 0 0 0 0 0 0 0 0 1

0 2 61 1 0 0 0 0 0 0 0 0 3 1

0 2 71 1 0 0 0 0 0 0 0 0 0 1

0 2 78 1 0 0 0 0 0 0 0 0 0 1

0 2 72 1 0 0 0 0 0 0 0 0 0 1

0 2 81 1 0 0 0 0 0 0 0 0 0 1

0 2 47 1 0 0 0 0 0 0 0 0 0 1

0 2 68 1 0 0 1 0 0 0 0 0 4 1

0 2 78 1 0 0 0 0 0 0 0 0 3 1

0 2 77 1 0 0 0 0 0 0 0 0 0 1

0 2 78 1 0 0 0 0 0 0 0 0 0 1

0 2 74 1 0 0 0 0 0 0 0 0 0 1

0 2 78 1 0 0 0 0 0 0 0 0 3 1

0 2 59 1 0 0 0 0 0 0 0 0 0 1

0 2 79 1 0 0 0 0 0 0 0 0 0 1

0 2 80 1 0 0 0 0 0 0 0 0 0 1

1 1 68 0 0 0 0 0 0 0 0 0 0 1

1 1 55 0 0 0 0 0 0 0 0 0 0 1

1 1 86 0 0 0 0 0 0 0 0 0 0 1

1 1 68 0 0 0 0 0 0 0 0 0 3 1

1 1 62 0 0 0 0 0 0 0 0 0 3 1

1 1 84 0 0 0 0 0 0 0 0 0 0 1

1 1 73 0 0 0 0 0 0 0 0 0 0 1

1 1 35 0 0 0 1 0 0 0 0 0 0 1

1 1 61 0 0 0 0 0 0 0 0 0 0 1

1 1 72 0 0 0 0 0 0 0 0 0 3 1

1 1 56 0 0 0 0 0 0 0 0 0 0 1

1 1 54 0 0 0 0 0 0 0 0 0 4 1

1 1 53 0 0 0 0 0 0 0 0 0 0 1

1 1 66 0 0 0 0 0 0 0 0 0 0 1

1 1 62 0 0 0 0 0 0 0 0 0 0 1

1 1 71 0 0 0 0 0 0 0 0 0 0 1

1 1 60 0 0 0 0 0 0 0 0 0 0 1

1 1 55 0 0 0 0 0 0 0 0 0 0 1

1 1 83 0 0 0 0 0 0 0 0 0 3 1

1 1 63 0 0 0 0 0 0 0 0 0 2 1

1 1 77 0 0 0 0 0 0 0 0 0 0 1

1 1 70 0 0 0 0 0 0 0 0 0 0 1

1 1 50 0 0 0 0 0 0 0 0 0 0 1

1 1 50 0 0 0 0 0 0 0 0 0 0 1

1 1 62 0 0 0 0 0 0 0 0 0 0 1

1 1 60 0 0 0 0 0 0 0 0 0 0 1

1 1 68 0 0 0 0 0 0 0 0 0 0 1

1 1 50 0 0 0 0 0 0 0 0 0 0 1

1 1 76 0 0 0 0 0 0 0 0 0 3 1

1 1 60 0 0 0 0 0 0 0 0 0 2 1

1 1 75 0 0 0 0 0 0 0 0 0 2 1

1 1 61 0 0 0 0 0 0 0 0 0 3 1

1 1 65 0 0 0 1 0 0 0 0 0 0 1

1 1 80 0 0 0 0 0 0 0 0 0 3 1

1 1 65 0 0 0 0 0 0 0 0 0 0 1

1 1 51 0 0 0 0 0 0 0 0 0 0 1

1 1 60 0 0 0 0 0 0 0 0 0 4 1

1 1 53 0 0 0 0 0 0 0 0 0 0 1

1 1 68 0 0 0 0 0 0 0 0 0 3 1

1 2 80 0 0 0 0 0 0 0 0 0 4 1

1 2 56 0 0 0 0 0 0 0 0 0 2 1

1 2 81 0 0 0 0 0 0 0 0 0 0 1

1 2 78 0 0 0 0 0 0 0 0 0 0 1

1 2 73 0 0 0 0 0 0 0 0 0 4 1

1 2 74 0 0 0 0 0 0 0 0 0 3 1

1 2 77 0 0 0 0 0 0 0 0 0 2 1

1 2 76 0 0 0 0 0 0 0 0 0 4 1

1 2 65 0 0 0 0 0 0 0 0 0 2 1

1 2 65 0 0 0 0 0 0 0 0 0 0 1

1 2 47 0 0 0 0 0 0 0 0 0 0 1

1 2 89 0 0 0 0 0 0 0 0 0 0 1

1 2 48 0 0 0 0 0 0 0 0 0 0 1

1 2 43 0 0 0 0 0 0 0 0 0 0 1

1 2 54 0 0 0 0 0 0 0 0 0 0 1

1 2 55 0 0 0 0 0 0 0 0 0 0 1

1 2 56 0 0 0 1 0 0 0 0 0 0 1

1 2 72 0 0 0 0 0 0 0 0 0 4 1

1 2 76 0 0 0 0 0 0 0 0 0 0 1

1 2 75 0 0 0 0 0 0 0 0 0 3 1

1 2 74 0 0 0 0 0 0 0 0 0 0 1

1 2 41 0 0 0 0 0 0 0 0 0 4 1

1 2 60 0 0 0 0 0 0 0 0 0 4 1

1 2 62 0 0 0 0 0 0 0 0 0 4 1

1 2 86 0 0 0 0 0 0 0 0 0 0 1

1 2 46 0 0 0 0 0 0 0 0 0 0 1

1 2 57 0 0 0 0 0 0 0 0 0 0 1

1 2 53 0 0 0 0 0 0 0 0 0 0 1

1 2 59 0 0 0 0 0 0 0 0 0 3 1

1 2 75 0 0 0 0 0 0 0 0 0 1 1

1 2 65 0 0 0 0 0 0 0 0 0 2 1

1 2 65 0 0 0 0 0 0 0 0 0 0 1

1 2 57 0 0 0 0 0 0 0 0 0 0 1

1 2 93 0 0 0 0 0 0 0 0 0 0 1

1 2 48 0 0 0 0 0 0 0 0 0 4 1

1 2 79 0 0 0 0 0 0 0 0 0 0 1

1 2 56 0 0 0 0 0 0 0 0 0 0 1

0 1 61 0 0 0 0 0 0 0 0 0 3 1

0 1 62 0 0 0 0 0 0 0 0 0 0 1

0 1 86 0 0 0 0 0 0 0 0 0 0 1

0 1 78 0 0 0 0 0 0 0 0 0 0 1

0 1 46 0 0 0 0 0 0 0 0 0 2 1

0 1 59 0 0 0 0 0 0 0 0 0 0 1

0 1 80 0 0 0 0 0 0 0 0 0 4 1

0 1 59 0 0 0 1 0 0 0 0 0 0 1

0 1 83 0 0 0 0 0 0 0 0 0 0 1

0 1 72 0 0 0 0 0 0 0 0 0 0 1

0 1 68 0 0 0 0 0 0 0 0 0 0 1

0 1 82 0 0 0 0 0 0 0 0 0 3 1

0 1 75 0 0 0 0 0 0 0 0 0 0 1

0 1 68 0 0 0 1 0 0 0 0 0 0 1

0 1 14 0 0 0 0 0 0 0 0 0 0 1

0 1 43 0 0 0 0 0 0 0 0 0 3 1

0 1 54 0 0 0 0 0 0 0 0 0 3 1

0 1 60 0 0 0 0 0 0 0 0 0 0 1

0 1 73 0 0 0 0 0 0 0 0 0 0 1

0 1 69 0 0 0 0 0 0 0 0 0 3 1

0 1 78 0 0 0 0 0 0 0 0 0 0 1

0 1 81 0 0 0 0 0 0 0 0 0 2 1

0 1 67 0 0 0 0 0 0 0 0 0 2 1

0 1 27 0 0 0 0 0 0 0 0 0 0 1

0 1 56 0 0 0 0 0 0 0 0 0 0 1

0 1 57 0 0 0 0 0 0 0 0 0 3 1

0 1 58 0 0 0 0 0 0 0 0 0 0 1

0 1 72 0 0 0 0 0 0 0 0 0 2 1

0 1 75 0 0 0 0 0 0 0 0 0 4 1

0 1 57 0 0 0 0 0 0 0 0 0 3 1

0 1 76 0 0 0 0 0 0 0 0 0 4 1

0 1 79 0 0 0 0 0 0 0 0 0 3 1

0 1 75 0 0 0 0 0 0 0 0 0 0 1

0 1 52 0 0 0 0 0 0 0 0 0 0 1

0 1 61 0 0 0 0 0 0 0 0 0 2 1

0 1 58 0 0 0 0 0 0 0 0 0 3 1

0 1 48 0 0 0 0 0 0 0 0 0 4 1

0 1 68 0 0 0 0 0 0 0 0 0 3 1

0 1 71 0 0 0 0 0 0 0 0 0 4 1

0 1 73 0 0 0 0 0 0 0 0 0 2 1

0 1 51 0 0 0 0 0 0 0 0 0 3 1

0 1 71 0 0 0 0 0 0 0 0 0 0 1

0 1 67 0 0 0 0 0 0 0 0 0 3 1

0 1 68 0 0 0 0 0 0 0 0 0 0 1

0 1 75 0 0 0 0 0 0 0 0 0 0 1

0 1 75 0 0 0 0 0 0 0 0 0 4 1

0 1 78 0 0 0 0 0 0 0 0 0 2 1

0 1 78 0 0 0 0 0 0 0 0 0 3 1

0 1 63 0 0 0 0 0 0 0 0 0 2 1

0 1 55 0 0 0 0 0 0 0 0 0 3 1

0 1 64 0 0 0 1 0 0 0 0 0 0 1

0 1 43 0 0 0 0 0 0 0 0 0 4 1

0 1 57 0 0 0 0 0 0 0 0 0 0 1

0 1 94 0 0 0 0 0 0 0 0 0 4 1

0 1 66 0 0 0 0 0 0 0 0 0 3 1

0 1 65 0 0 0 0 0 0 0 0 0 0 1

0 1 73 0 0 0 1 0 0 0 0 0 0 1

0 1 58 0 0 0 0 0 0 0 0 0 3 1

0 1 61 0 0 0 0 0 0 0 0 0 0 1

0 1 61 0 0 0 0 0 0 0 0 0 1 1

0 1 76 0 0 0 0 0 0 0 0 0 0 1

0 1 72 0 0 0 0 0 0 0 0 0 0 1

0 1 65 0 0 0 0 0 0 0 0 0 0 1

0 1 59 0 0 0 0 0 0 0 0 0 2 1

0 1 60 0 0 0 0 0 0 0 0 0 0 1

0 1 80 0 0 0 0 0 0 0 0 0 3 1

0 1 67 0 0 0 0 0 0 0 0 0 0 1

0 1 48 0 0 0 0 0 0 0 0 0 2 1

0 1 37 0 0 0 0 0 0 0 0 0 4 1

0 1 57 0 0 0 1 0 0 0 0 0 0 1

0 1 80 0 0 0 0 0 0 0 0 0 0 1

0 1 76 0 0 0 0 0 0 0 0 0 2 1

0 1 62 0 0 0 0 0 0 0 0 0 0 1

0 1 76 0 0 0 0 0 0 0 0 0 0 1

0 1 45 0 0 0 0 0 0 0 0 0 3 1

0 1 72 0 0 0 0 0 0 0 0 0 0 1

0 1 76 0 0 0 0 0 0 0 0 0 0 1

0 1 62 0 0 0 0 0 0 0 0 0 4 1

0 1 65 0 0 0 1 0 0 0 0 0 0 1

0 1 73 0 0 0 0 0 0 0 0 0 0 1

0 1 72 0 0 0 0 0 0 0 0 0 0 1

0 1 65 0 0 0 0 0 0 0 0 0 2 1

0 1 32 0 0 0 0 0 0 0 0 0 0 1

0 1 33 0 0 0 0 0 0 0 0 0 0 1

0 1 72 0 0 0 0 0 0 0 0 0 2 1

0 1 53 0 0 0 0 0 0 0 0 0 0 1

0 1 64 0 0 0 0 0 0 0 0 0 0 1

0 1 80 0 0 0 0 0 0 0 0 0 0 1

0 1 38 0 0 0 0 0 0 0 0 0 0 1

0 1 76 0 0 0 0 0 0 0 0 0 0 1

0 1 45 0 0 0 0 0 0 0 0 0 0 1

0 1 71 0 0 0 0 0 0 0 0 0 0 1

0 1 62 0 0 0 0 0 0 0 0 0 0 1

0 1 48 0 0 0 0 0 0 0 0 0 0 1

0 1 66 0 0 0 0 0 0 0 0 0 0 1

0 1 76 0 0 0 0 0 0 0 0 0 1 1

0 1 53 0 0 0 0 0 0 0 0 0 2 1

0 1 61 0 0 0 0 0 0 0 0 0 2 1

0 1 48 0 0 0 0 0 0 0 0 0 0 1

0 1 66 0 0 0 0 0 0 0 0 0 0 1

0 1 56 0 0 0 0 0 0 0 0 0 0 1

0 1 76 0 0 0 0 0 0 0 0 0 0 1

0 1 59 0 0 0 0 0 0 0 0 0 4 1

0 1 88 0 0 0 0 0 0 0 0 0 0 1

0 1 47 0 0 0 0 0 0 0 0 0 2 1

0 1 65 0 0 0 0 0 0 0 0 0 2 1

0 1 60 0 0 0 0 0 0 0 0 0 0 1

0 1 86 0 0 0 0 0 0 0 0 0 4 1

0 1 60 0 0 0 0 0 0 0 0 0 0 1

0 1 61 0 0 0 0 0 0 0 0 0 0 1

0 1 29 0 0 0 0 0 0 0 0 0 2 1

0 1 82 0 0 0 0 0 0 0 0 0 0 1

0 1 72 0 0 0 0 0 0 0 0 0 0 1

0 1 76 0 0 0 0 0 0 0 0 0 0 1

0 1 30 0 0 0 0 0 0 0 0 0 0 1

0 1 45 0 0 0 0 0 0 0 0 0 0 1

0 1 88 0 0 0 0 0 0 0 0 0 0 1

0 1 77 0 0 0 0 0 0 0 0 0 0 1

0 1 61 0 0 0 0 0 0 0 0 0 0 1

0 1 62 0 0 0 0 0 0 0 0 0 0 1

0 2 61 0 0 0 0 0 0 0 0 0 4 1

0 2 80 0 0 0 0 0 0 0 0 0 2 1

0 2 74 0 0 0 0 0 0 0 0 0 0 1

0 2 81 0 0 0 0 0 0 0 0 0 0 1

0 2 48 0 0 0 0 0 0 0 0 0 0 1

0 2 70 0 0 0 0 0 0 0 0 0 0 1

0 2 83 0 0 0 0 0 0 0 0 0 0 1

0 2 72 0 0 0 0 0 0 0 0 0 0 1

0 2 15 0 0 0 0 0 0 0 0 0 0 1

0 2 49 0 0 0 0 0 0 0 0 0 2 1

0 2 62 0 0 0 0 0 0 0 0 0 0 1

0 2 66 0 0 0 0 0 0 0 0 0 0 1

0 2 63 0 0 0 0 0 0 0 0 0 2 1

0 2 65 0 0 0 0 0 0 0 0 0 3 1

0 2 37 0 0 0 0 0 0 0 0 0 3 1

0 2 57 0 0 0 1 0 0 0 0 0 0 1

0 2 61 0 0 0 0 0 0 0 0 0 3 1

0 2 76 0 0 0 0 0 0 0 0 0 2 1

0 2 61 0 0 0 0 0 0 0 0 0 0 1

0 2 79 0 0 0 1 0 0 0 0 0 3 1

0 2 57 0 0 0 0 0 0 0 0 0 2 1

0 2 41 0 0 0 0 0 0 0 0 0 0 1

0 2 38 0 0 0 0 0 0 0 0 0 3 1

0 2 41 0 0 0 0 0 0 0 0 0 0 1

0 2 70 0 0 0 0 0 0 0 0 0 0 1

0 2 45 0 0 0 0 0 0 0 0 0 3 1

0 2 65 0 0 0 0 0 0 0 0 0 2 1

0 2 72 0 0 0 0 0 0 0 0 0 4 1

0 2 64 0 0 0 0 0 0 0 0 0 4 1

0 2 55 0 0 0 0 0 0 0 0 0 0 1

0 2 72 0 0 0 1 0 0 0 0 0 0 1

0 2 94 0 0 0 0 0 0 0 0 0 0 1

0 2 46 0 0 0 0 0 0 0 0 0 2 1

0 2 76 0 0 0 0 0 0 0 0 0 3 1

0 2 56 0 0 0 0 0 0 0 0 0 0 1

0 2 59 0 0 0 1 0 0 0 0 0 0 1

0 2 80 0 0 0 0 0 0 0 0 0 0 1

0 2 57 0 0 0 0 0 0 0 0 0 0 1

0 2 55 0 0 0 0 0 0 0 0 0 0 1

0 2 65 0 0 0 0 0 0 0 0 0 0 1

0 2 65 0 0 0 0 0 0 0 0 0 3 1

0 2 49 0 0 0 0 0 0 0 0 0 0 1

0 2 65 0 0 0 0 0 0 0 0 0 0 1

0 2 65 0 0 0 0 0 0 0 0 0 3 1

0 2 60 0 0 0 0 0 0 0 0 0 0 1

0 2 61 0 0 0 0 0 0 0 0 0 0 1

0 2 52 0 0 0 0 0 0 0 0 0 0 1

0 2 55 0 0 0 0 0 0 0 0 0 0 1

0 2 74 0 0 0 0 0 0 0 0 0 0 1

0 2 78 0 0 0 0 0 0 0 0 0 0 1

0 2 62 0 0 0 0 0 0 0 0 0 0 1

0 2 72 0 0 0 0 0 0 0 0 0 0 1

0 2 52 0 0 0 0 0 0 0 0 0 3 1

0 2 65 0 0 0 1 0 0 0 0 0 0 1

0 2 70 0 0 0 0 0 0 0 0 0 0 1

0 2 49 0 0 0 0 0 0 0 0 0 2 1

0 2 48 0 0 0 0 0 0 0 0 0 0 1

0 2 57 0 0 0 1 0 0 0 0 0 0 1

0 2 55 0 0 0 0 0 0 0 0 0 0 1

0 2 57 0 0 0 0 0 0 0 0 0 4 1

0 2 47 0 0 0 0 0 0 0 0 0 0 1

0 2 76 0 0 0 0 0 0 0 0 0 2 1

0 2 80 0 0 0 0 0 0 0 0 0 0 1
